# Supplementary material for: The Arabidopsis phosphatase PP2C12 negatively regulates LRX-RALF-FER-mediated cell wall integrity sensing
Source: EMBO J. 2025 Nov 17;45(1):243–60. doi: 10.1038/s44318-025-00614-x (PMC12759080; doi:10.1038/s44318-025-00614-x)
Supplement: Supplementary file 1 — Appendix [file 44318_2025_614_MOESM1_ESM.pdf]

Appendix

Appendix for “The Arabidopsis phosphatase PP2C12 negatively regulates LRX-RALF-FER-mediated cell wall integrity sensing” by Hou et al.

Table of Contents:

Appendix Figure S1………… page 2

Appendix Figure S2………… page 4

Appendix Figure S3………… page 5

Appendix Figure S4………… page 7

Appendix Figure S5………… page 8

Appendix Figure S6………… page 9

Appendix Figure S7………… page 10

Appendix Table S1………….. page 11

Appendix Table S2………….. page 12

Appendix Table S3………… page 13

Appendix

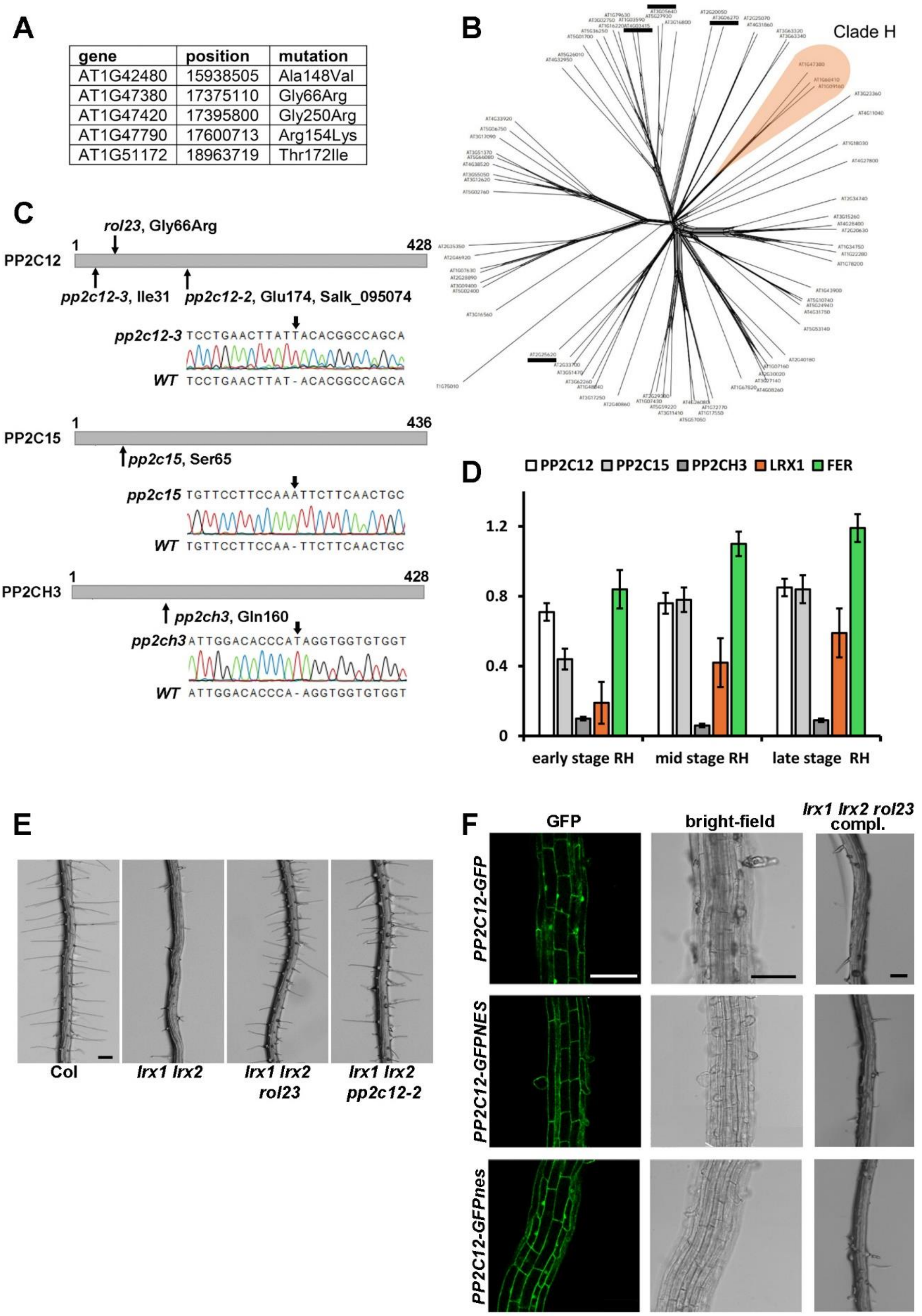
A
gene | position | mutation
AT1G42480 | 15938505 | Ala148Val
AT1G47380 | 17375110 | Gly66Arg
AT1G47420 | 17395800 | Gly250Arg
AT1G47790 | 17600713 | Arg154Lys
AT1G51172 | 18963719 | Thr172Ile
B
Clade H
C
rol23, Gly66Arg
1
428
PP2C12
pp2c12-3, Ile31
pp2c12-2, Glu174, Salk_095074
pp2c12-3 TCCTGAACTTATTACACGGCCAGCA
WT TCCTGAACTTAT-ACACGGCCAGCA
1
436
PP2C15
pp2c15, Ser65
pp2c15 TGTTCCTTCCAAATTCTTCAACTGC
WT TGTTCCTTCCAA-TTCTTCAACTGC
1
428
PP2CH3
pp2ch3, Gln160
pp2ch3 ATTGGACACCCATAGGTGGTGTGGT
WT ATTGGACACCCA-AGGTGGTGTGGT
D
PP2C12
PP2C15
PP2CH3
LRX1
FER
1.2
0.8
0.4
0
early stage RH
mid stage RH
late stage RH
E
Col
lrx1 lrx2
lrx1 lrx2 rol23
lrx1 lrx2 pp2c12-2
F
GFP
bright-field
lrx1 lrx2 rol23 compl.
PP2C12-GFP
PP2C12-GFPNES
PP2C12-GFPnes

Appendix

Appendix Figure S1 *ROL23* codes for PP2C12.

(A) Whole-genome sequencing revealed several SNPs on chromosome 1 linked to the *rol23* phenotype. (B) Phylogenetic network of the PP2C family of phosphatases of *Arabidopsis thaliana*. Clade H PP2Cs are highlighted. PP2Cs of other clades used in this study (see also Table 1) are marked with a black bar. The analysis was performed using: https://uni-tuebingen.de/fakultaeten/mathematisch-naturwissenschaftliche-fakultaet/fachbereiche/informatik/lehrstuehle/algorithms-in-bioinformatics/software/splitstree/

(C) Schematic representation of the clade H PP2C family, the arrows indicating the CRISPR/Cas9-induced 1 bp insertions in all the mutants. The shifts in the ORF cause premature stop codon after encoding 16, 12, and 10 aberrant amino acids in *PP2C12*, *PP2C15*, and *PP2CH3*, respectively. (D) Comparison of expression levels based on single-cell RNA sequencing data (Ryu et al Ref) of the *PP2CH* family, *LRX1*, and *FER* throughout root hair development shows comparable levels of *PP2C12* and *PP2C15* while *PP2CH3* shows lower expression. (E) The *lrx1 lrx2* double mutant develops an enhanced *lrx1* root hair phenotype, which is also suppressed by *rol23*. Scale bar = 300 μm. (F) *PP2C12::PP2C12-GFP* is ubiquitously expressed in Arabidopsis roots. GFP fluorescence and bright-field images are shown (left and middle panels), and the root hair phenotype of *lrx1 lrx2 rol23* mutants complemented with the constructs (right panels). Scale bar = 300 μm.

Appendix

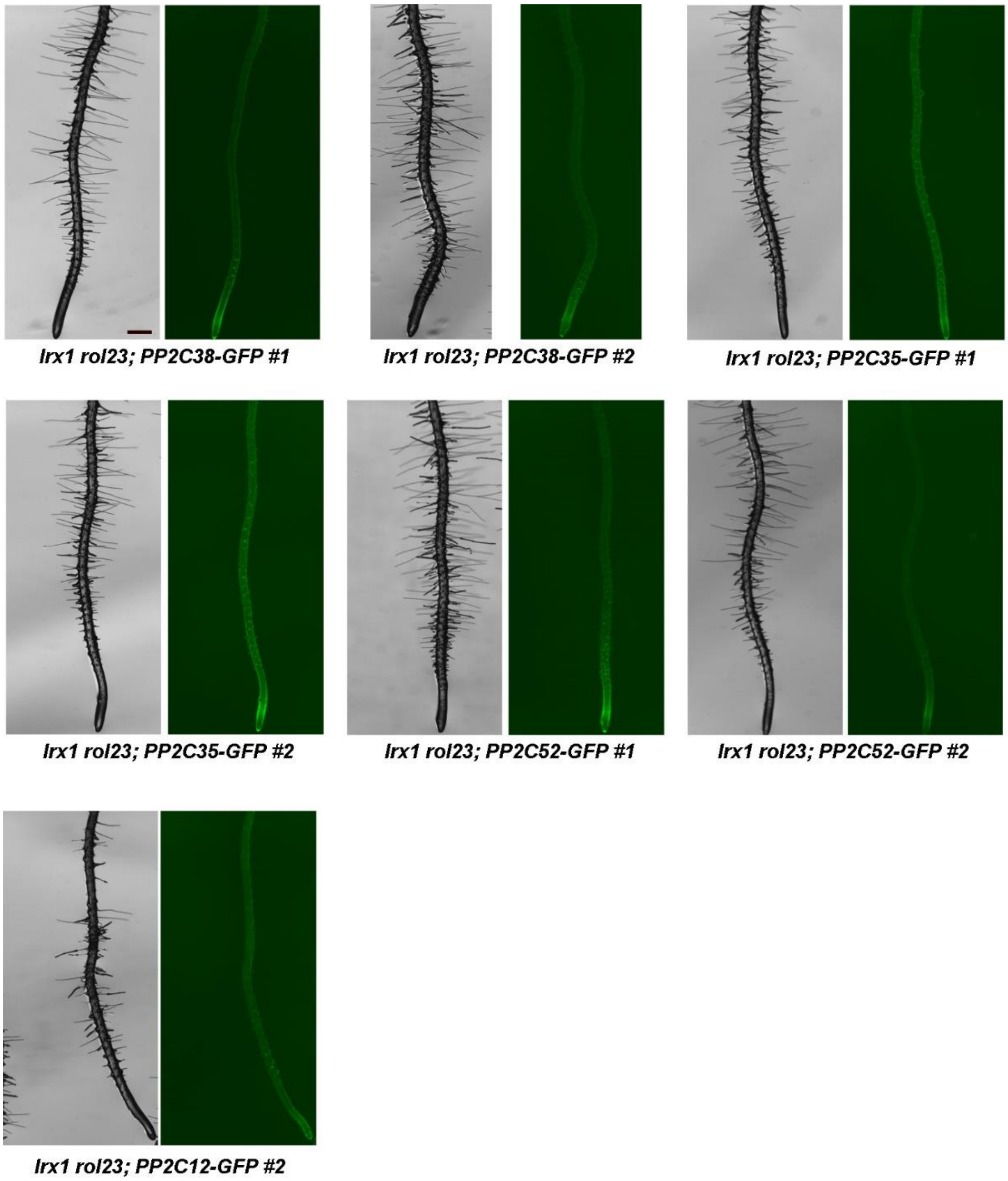

Appendix Figure S2 Specific function of clade H PP2Cs.

Representative images of 5-days-old Arabidopsis seedlings grown vertically. Different *PP2C12::PP2Cs-GFP* constructs in the *lrx1 rol23* mutant induce comparable expression levels, i.e. GFP fluorescence, as in *PP2C12::PP2C12-GFP* yet do not complement the *rol23* phenotype. Scale bar = 500 µm.

Appendix

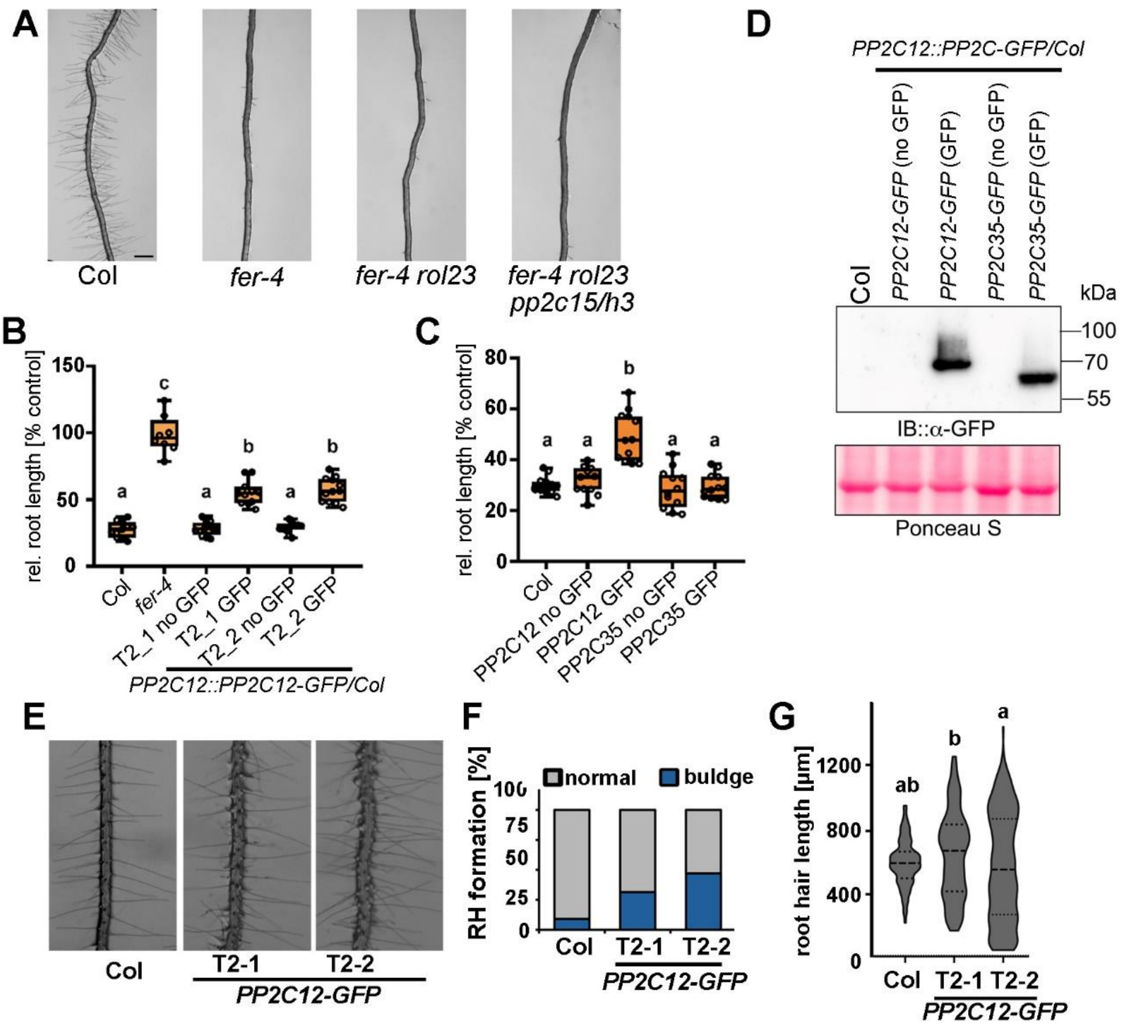

Appendix Figure S3 PP2C12 affects RALF/FER-related processes.

(A) Roots of 5-d-old Arabidopsis seedlings grown in a vertical direction. Neither *rol23* nor *rol23 pp2c15 pp2ch3* (*rol23 pp2c15/h3*) have a significant effect on the *fer-4* knock-out root hair phenotype. Scale bar = 500 µm. (B,C) Quantification of primary root length of 7-d-old seedlings grown in the absence (mock) or presence of 2 M RALF1 peptide. The primary root length is expressed as relative to mock data for each genotype. Strong expression of *PP2C12::PP2C12-GFP* causes reduced sensitivity to RALF1, as revealed by reduced root growth inhibition. GFP-less seedlings of a population segregating for the transgene show wild type Col-like response to RALF1, but GFP positive lines respond less. This effect is not seen with the non-H-clade *PP2C35*. Different letters indicate significant differences. (one-way ANOVA with Tukey's unequal N-HSD post hoc test, P< 0.01). Similar results with at least two independent experiments were obtained. (D) Immunoblot with an anti(α)-GFP antibody on total extracts of seedlings shown in (B) and (C). Blot stained with Ponceau S

shows comparable loading. Comparable accumulation of the PP2C12-GFP and PP2C35-GFP were detected, whereas no detectable signals were shown in the GFP-less lines. (E) Two independent wild-type Col lines overexpressing *PP2C12-GFP* revealed frequently swollen root hair bases. Scale bar = 500 µm (F) Quantification of frequency of the aberrant root hair phenotype of plants shown in (E), average value of 5 seedlings are shown, >80 root hairs per seedlings were analyzed. (G) Quantification of the root hair length of plants shown in (E). While the mean value of root hair length is not significantly different in the transgenic lines from the wild type, there is a clearly greater heterogeneity as observed in the violin plots. The median is represented by the central dash line, and the 25% and 75% percentages are represented by the dotted lines. Genotypes with like letter designations are not statistically different (one-way ANOVA with Tukey's unequal N-HSD post hoc test, $P < 0.01$).

Appendix

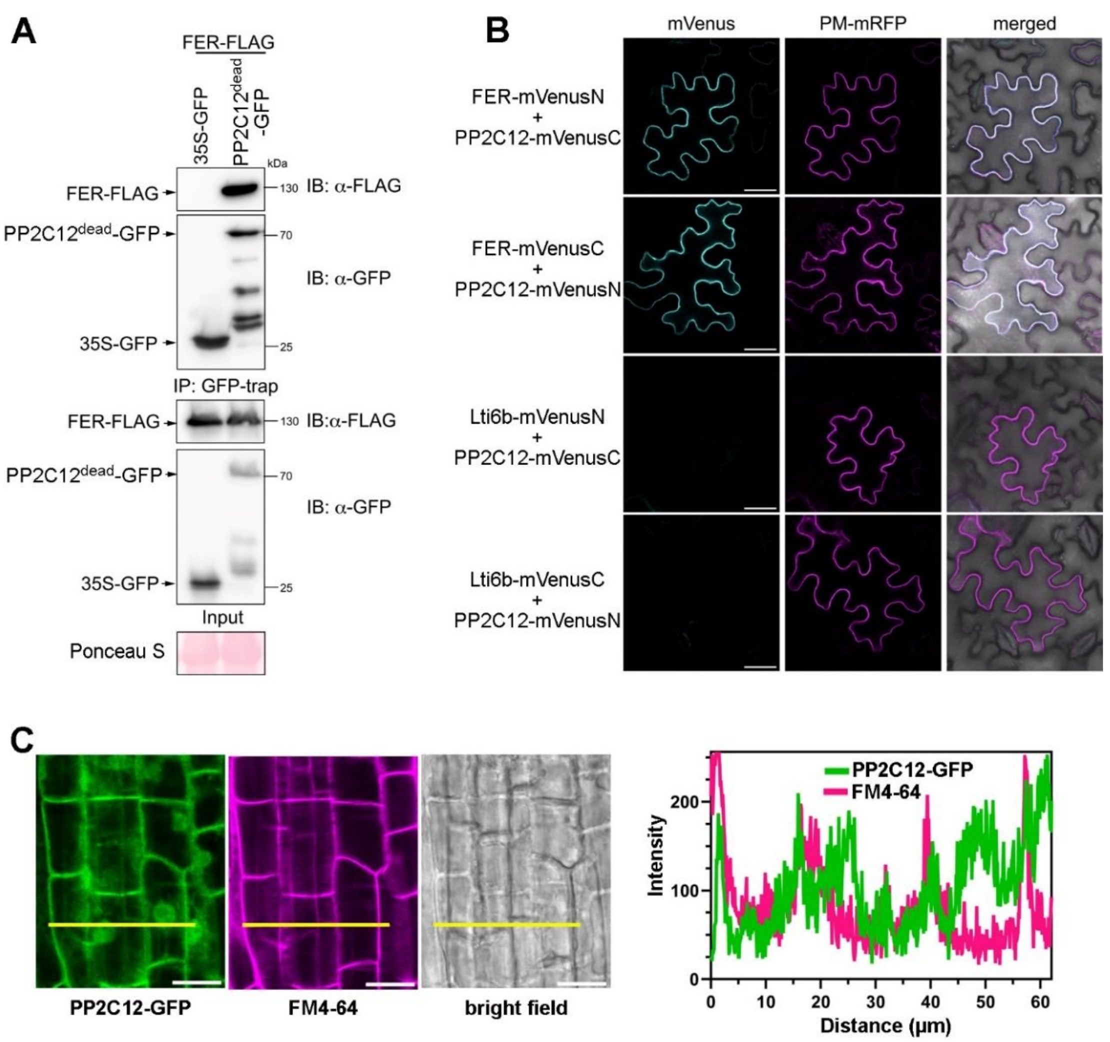

Appendix Figure S4 PP2C12 interacts with FER at the plasma membrane.

(A) FER interacts with *PP2C12*^{dead} in *N. benthamiana*. Co-IP experiment using overexpressed cytoplasmic GFP as the negative control (opposed to the membrane associated Lti6b-GFP shown in Fig 4). *FER-FLAG* was transiently co-expressed in *N. benthamiana* leaves with either *PP2C12*^{dead}*-GFP* or *GFP*. Immunoprecipitation of *PP2C12*^{dead}-GFP and GFP was done using the anti-GFP trap. FER-FLAG was detected with anti (α)-FLAG antibody when co-expressed with *PP2C12*^{dead}*-GFP* but not *GFP*. Blot stained with Ponceau S shows comparable loading. (B) BiFC assay in *N. benthamiana* as shown in Fig 4 with the addition of the PM marker REM 1.2-mRFP reveals interaction of FER and PP2C12 at the plasma membrane. Scale bar= 20 μm. (C) Section of root tissue of a *PP2C12-GFP* expressing line and co-localization with the membrane marker FM4-64. Panels on the left show GFP fluorescence, FM4-64 staining, and bright-field microscopy of the identical tissue covering three cells. The yellow line indicates position of the virtual section shown in the graph on the left. Overlapping signals of GFP and the peaks of FM4-64 signal at positions of the plasma membranes. Scale Bars = 20 μm.

Appendix

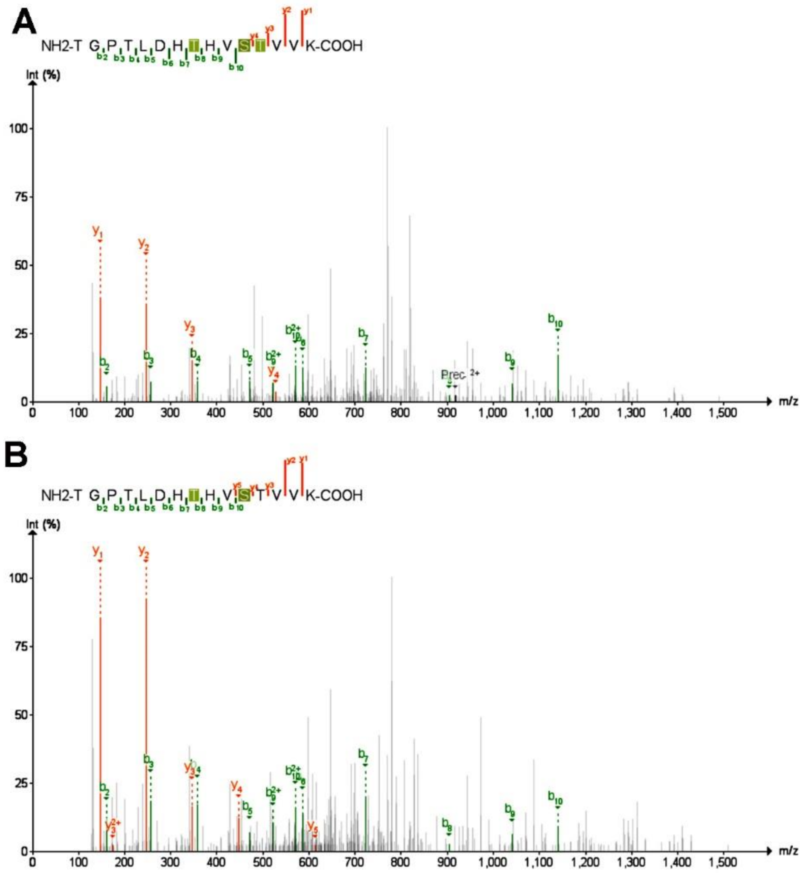

Appendix Figure S5 MS2 spectra of peptides identifying FER Thr696 as a dephosphorylation target of PP2C12.

A) The triply phosphorylated peptide carrying pT692, pS695, and pT696 decreased in abundance after treatment with PP2C12. B) The doubly phosphorylated peptide carrying pT692 and pS695 increased in abundance after treatment with PP2C12.

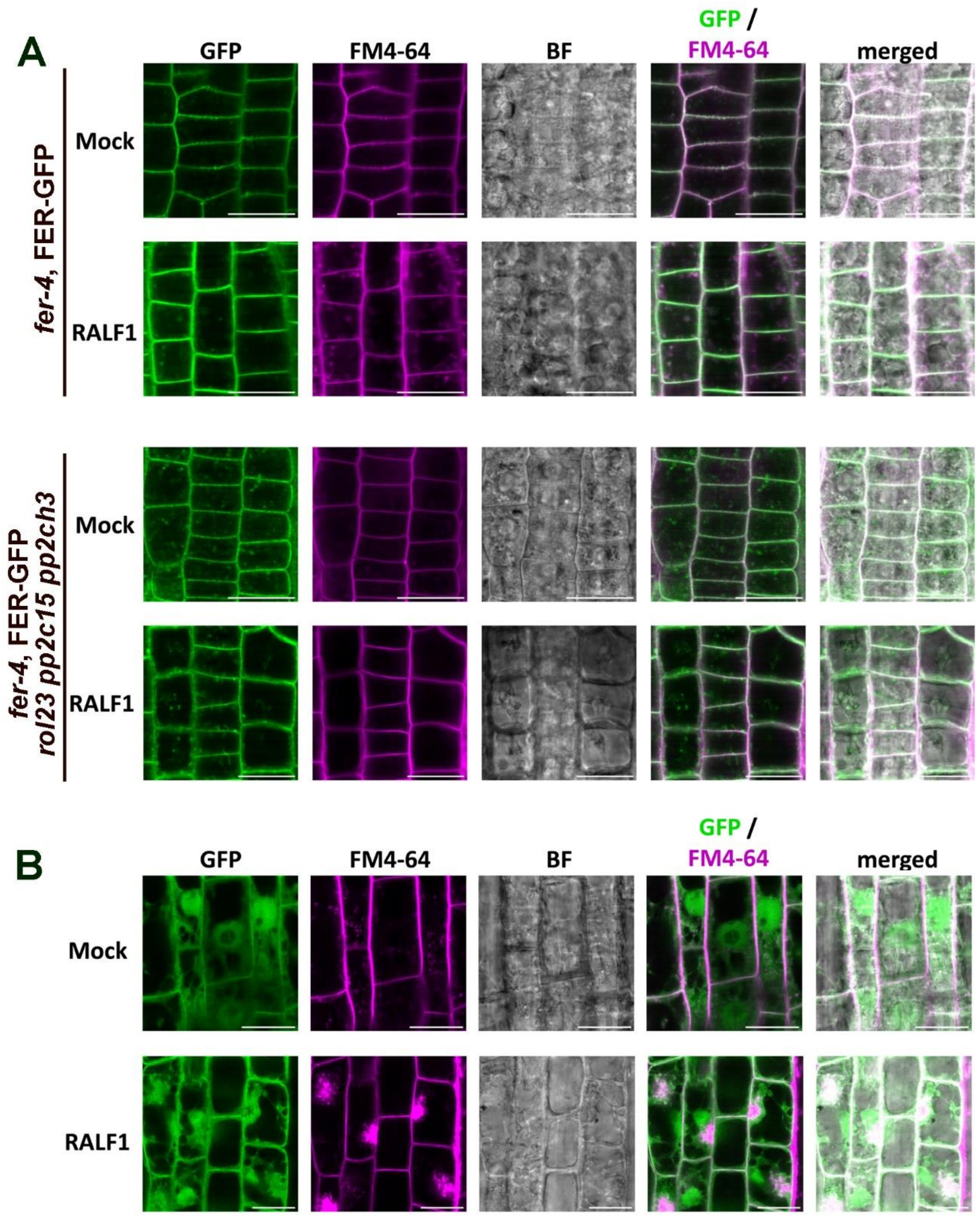

Appendix Figure S6 Dynamics of RALF1-induced FER-GFP and PP2C12-GFP localization.

(A) Endocytosis of FER-GFP is induced by RALF1 mainly in the *fer-4*; *FER-GFP* line, but not in the *fer-4 rol23 pp2c15 pp2ch3*, *FER-GFP*, which shows a steady-state level of FER-GFP endocytosis. These lines show no difference in FM4-64 stained intracellular bodies, pointing at increased endocytosis of FER-GFP. (B) RALF1-induces invaginations can be observed, which are surrounded by PP2C12-GFP fluorescence in the cytoplasm. Other major changes in PP2C12-GFP abundance or localization upon RALF1 treatment were not observed. Scale bars = 20 µm.

Appendix

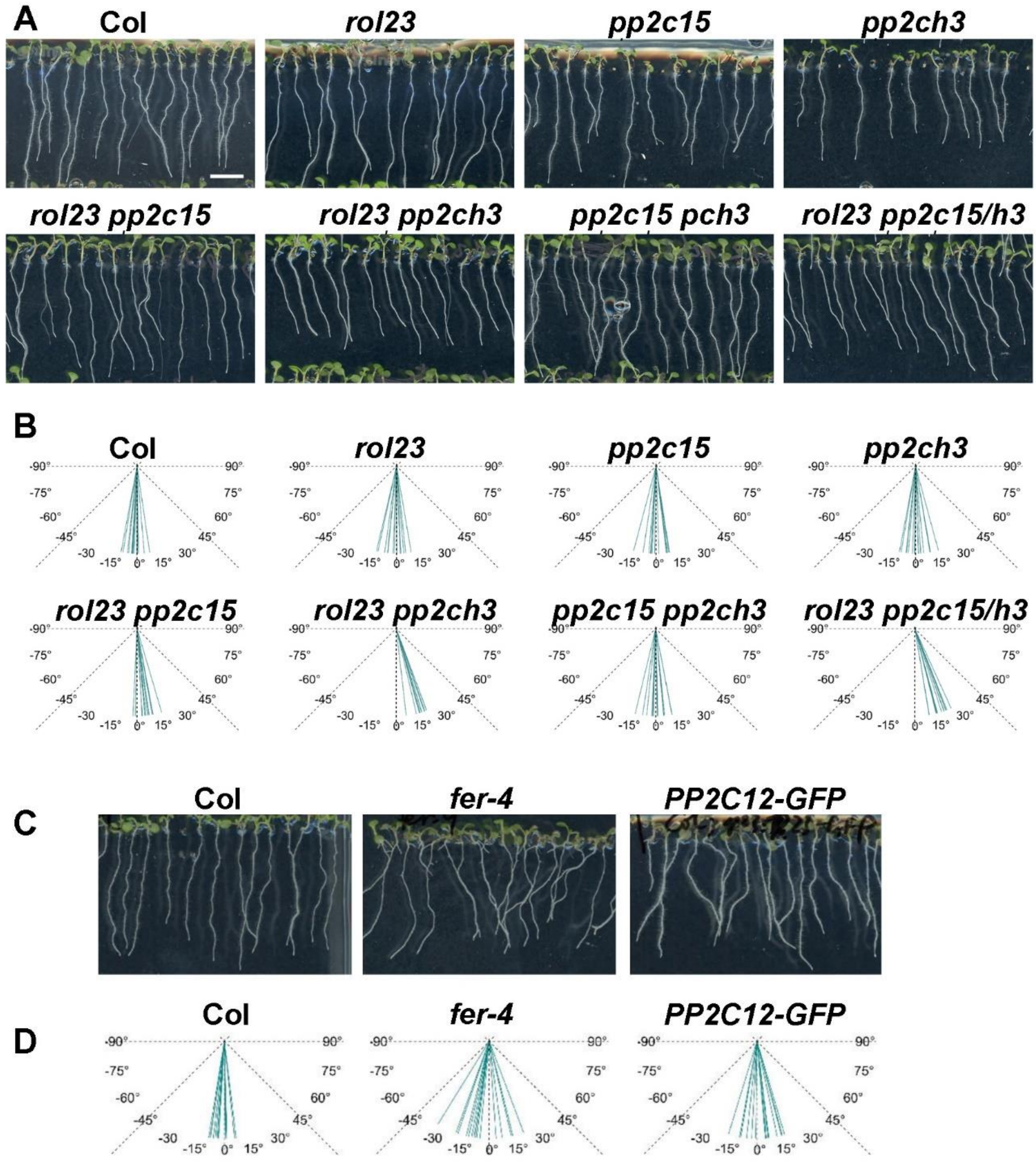

Appendix Figure S7 Clade H PP2C members redundantly regulate the root skewing.

(A) Images of 7-d-old Arabidopsis seedlings of Col and clade H *pp2c* mutants grown in a vertical orientation are shown. The primary root skewing is observed in *rol23 pp2c15*, *rol23 pp2ch3*, and *rol23 pp2c15 pp2ch3* (labelled in panel A and B as *rol23 pp2c15/h3*). (B) Quantification of the deviation from the gravitropic vector of seedlings shown in (A). (C) Images of 7-d-old Arabidopsis seedlings of Col, *fer-4* mutant, and a strongly expressing *PP2C12-GFP* line show reduced gravitropism in the latter two lines. (D) Quantification of the

deviation from the gravitropic vector of seedlings shown in (C). Bending angles of 15 seedlings per line were measured.

Appendix Table S1

Table S1. Phosphorylation sites and diagnostic peptides identified on the recombinant FER cytoplasmic domain by LC-MS/MS. Only phosphorylation sites identified by a localization score of >0.75 were included.

Site	Phosphopeptides(s)[a]	Best Localization Score
Thr508	TN**t**TGSYASSLPSNLCR;TN**tt**GSYASSLPSNLCR	0.9557
Thr509	TNT**t**GSYASSLPSNLCR;TN**tt**GSYASSLPSNLCR	0.9479
Ser525	HF**s**FAEIK;HF**s**FAEIKAATK	1
Thr533	AA**t**KNFDE**s**RVLGVGGFGK	1
Ser539	AATKNFDE**s**RVLGVGGFGK;AA**t**KNFDE**s**RVLGVGGFGK;NFDE**s**RVLGVGGFGK	1
Thr559	VYRGEIDGG**t**TK	0.9444
Thr560	VYRGEIDGGT**t**K	0.823
Ser571	GNPM**s**EQGVHEFQTEIEMLSK	0.892
Thr580	GNPMSEQGVHEFQ**t**EIEMLSK;GNPMSEQGVHEFQ**t**EIEML**s**KLR	0.9342
Ser586	GNPMSEQGVHEFQtEIEML**s**KLR	0.9337
Thr625	EHLYK**t**QNPSLPWK	0.8804
Ser629	TQNP**s**LPWK	0.9522
Tyr648	GLH**y**LHTGAKHTIIHR	0.8224
Thr651	GLHYLH**t**GAKHTIIHR	0.9234
Thr656	GLHYLHTGAKH**t**IIHR	0.7871
Thr664	DVK**t**TNILLDEK	0.9574
Thr665	DVKT**t**NILLDEK;DVKT**t**NILLDEKWVAK	0.9391
Ser683	VSDFGL**s**K;VSDFGL**s**KTGPTLDHTHVSTVVK	0.9511
Thr685	VSDFGLSK**t**GP**t**LDHTHVSTVVK	0.8953
Thr688	TGP**t**LDHTHVSTVVK;VSDFGLSKTGP**t**LDHtHVSTVVK;VSDFGLSK**t**GP**t**LDHTHVSTVVK	0.8953
Thr692	TGPTLDH**t**HVSTVVK;TGPTLDH**t**HV**s**TVVK;TGPTLDH**t**HV**st**VVK;TGPTLDH**t**HV**st**VVKGSFGYLDPEYFR;VSDFGLSKTGP**t**LDH**t**HVSTVVK	0.9601
Ser695	TGPTLDHTHV**s**TVVK;TGPTLDHTHV**st**VVKGSFGYLDPEYFR;TGPTLDHTHV**st**VVKG**s**FGYLDPEYFR;TGPTLDH**t**HV**s**TVVK;TGPTLDH**t**HV**st**VVK;TGPTLDH**t**HV**st**VVKGSFGYLDPEYFR	0.9602
Thr696[b]	TGPTLDHTHVS**t**VVKGSFGYLDPEYFR;TGPTLDHTHV**st**VVKGSFGYLDPEYFR;TGPTLDHTHV**st**VVKG**s**FGYLDPEYFR;TGPTLDH**t**HV**st**VVK;TGPTLDH**t**HV**st**VVKGSFGYLDPEYFR	0.9602
Ser701	G**s**FGYLDPEYFR;TGPTLDHTHVSTVVKG**s**FGYLDPEYFR;TGPTLDHTHV**st**VVKG**s**FGYLDPEYFR	0.963
Ser748	EQV**s**LAEWAPYCYK;EQV**s**LAEWAPYCYKK	0.9652
Thr785	FAE**t**AMK	1
Ser858	NDKSSDVYEGNVTD**s**R	0.7828
Ser866	SSGIDM**s**IGGR	0.9408

a, Phosphorylated residues are indicated in bold. Some peptides appear in multiple rows where doubly and triply phosphorylated peptides were identified.

b, Dephosphorylated by ROL23.

Appendix

Appendix Table S2

Primers used

Primers used for cloning		
ROL23PromF	ATGACTAGTGTGTTAATCTTCTTTGACACATC	
ROL23PromR	TGAGGCGCGCCCTTCCTCTAAGCTGCGTCTAG	
ROL23CDSF	GGCGCGCCATGTCAACAAAGGGAGAACATC	
ROL23CDSR	GGCGCGCCTGTCGCTTTCACTACTATGTC	
EGR_F	GACGCAGCTTAGAGGAAGGGCGCGCATGGGACATTTCTCTTCCATG	
EGR_R	CTTCTCCTTTACTCATGGCGCGCCATAGAGATGGCGACGACGATG	
PP2C28_F	GACGCAGCTTAGAGGAAGGGCGCGCATGGTATCATCGGCAACTATATTG	
PP2C28_R	CTTCTCCTTTACTCATGGCGCGCCAAGTAGAAGGTCCAGCTAAATC	
PP2C52_F	GACGCAGCTTAGAGGAAGGGCGCGCATGGGGGGTTGTGTGTCGACTAG	
PP2C52_R	CTTCTCCTTTACTCATGGCGCGCCAAGTCTTCGATTTCTCTTCAG	
PP2C35_F	GACGCAGCTTAGAGGAAGGGCGCGCATGGGTTGTGTTCAATGCAAATG	
PP2C35_R	CTTCTCCTTTACTCATGGCGCGCCATTCATTAGACAGCTTTTTAATC	
PP2C15_F	TCAGGCGCGCCATGGCGTCTAGAGAAGGAAAG	
PP2C15_R	TCAGGCGCGCCAGATGACTTTAACTCCACTTG	
PP2CH3_F	GTTCCAGCCAATGCTGAAGGCGCGCATGAGCGTGTCAAAAGCATCG	
PP2CH3_R	CTTCTCCTTTACTCATGGCGCGCCAAGTAGTCACTGAACCTTTGC	
GFPNESnes_F	CATTACCTGTCCACACAATCTG	
NES_R	GATCGGGGAAATTCGAGCTCTCACTTGTTAATATCAAGTCCAGCCAACTTAAGAGC	
nes_R	CTCCAGCTGCCTTAAGAGCAAGCTCGTTGTGGTGGTGGTGGTGGTGT	
pET28a(+)-FER CD_F	CGCGGATCCGAATTCGCTTACCGCAGACGTAAGCG	
pET28a(+)-FER CD_R	CGCAAGCTTGTCGACCTAACGTCCCTTTGGATTCATGA	
pMALc4e-MBP_ PP2C12_F	ATCCTCTAGAGTCGACATGTCAACAAAGGGAGAACATC	
pMALc4e-MBP_ PP2C12_R	GGCCAGTGCCAAGCTTCTAGTCGCTTTCACTACTATGT	
pDEST-Cterm tag-FER FL_F	GGGGACAAGTTTGTACAAAAAAGCAGGCTATGAAGATCACAGAGGGACG	
pDEST-Cterm tag-FER FL_R	GGGGACCACTTTGTACAAGAAAGCTGGGTCACGTCCCTTTGGATTCATGA	
pDEST-Nterm tag-PP2C12_F	GGGGACAAGTTTGTACAAAAAAGCAGGCTGGATGTCAACAAAGGGAGAACA	
pDEST-Nterm tag-PP2C12_R	GGGGACCACTTTGTACAAGAAAGCTGGGTCTAGTCGCTTTCACTACTAT	
pDEST-Cterm tag-lti1.3_F	GGGGACAAGTTTGTACAAAAAAGCAGGCTATGAGTACAGCCACTTTCGTAG	
pDEST-Cterm tag-lti1.3_R	GGGGACCACTTTGTACAAGAAAGCTGGGTCCTTGGTGATGATATAAAGAGCG	
FER_R4	TGGTCTAGTGTAGGACCAGTC	
FERT696A_F	CACACACGTAAGCgctGTTG	mutated bases are in lower case
FERT696D_F	CACACACGTAAGCgatGTTG	mutated bases are in lower case
FERT696E_F	CACACACGTAAGCgaaGTTG	mutated bases are in lower case
rol23 CAPS marker		
ROL23_F1	GACTAACTTGGATTTCAAGTTTCAG	
SG1	AGAGAAAGAAGGCAAATAATAGGCC	mutated bases to introduce StuI site in *rol23*
Crispr genotyping		
pp2c12-3		
ROL23_F1	GACTAACTTGGATTTCAAGTTTCAG	
ROL23S182_R	CTTCACCACCACTTGCAGTGAC	
pp2c15		
410_F5	CATCAGAGTACTAAGTTAGTTTC	
410_R1	CTAGGAAGAGCACTAATAACATG	
pp2ch3		
160_F1	CTAAGGAACATCTTCTGGAAAAC	
160_R1	TTGCATTGGCATAATCAAAGCAC	
Crispr/CAS9 gRNAs		
ROL23 gRNA_2F	attgGACAATCCTGAACTTATACA	
ROL23 gRNA_2R	aaacTGTATAAGTTCAGGATTGTC	
PP2C15 gRNA_1F	attgAAAGCAGTTGAAGAATTGGA	
PP2C15 gRNA_1R	aaacTCCAATTCTTCAACTGCTTT	
PP2CH3 gRNA_1F	attgTGCATATTGGACACCCAAGG	
PP2CH3 gRNA_1R	aaacCCTTGGGTGTCCAATATGCA	

Appendix

Appendix Table S3

Detailed p values of statistical analyses

Tukey's multiple comparisons tests	Adjusted P Value
Figure 1C	
Col vs. lrx1	*<0.0001*
Col vs. lrx1 rol23	*0.9995*
Col vs. lrx1 pch1-2	*0.9647*
Col vs. lrx1 pch1-1	*0.8014*
lrx1 vs. *lrx1 rol23*	*<0.0001*
lrx1 vs. *lrx1 pch1-2*	*<0.0001*
lrx1 vs. *lrx1 pch1-1*	*<0.0001*
lrx1 rol23 vs. *lrx1 pch1-2*	*0.9932*
lrx1 rol23 vs. *lrx1 pch1-1*	*0.703*
lrx1 pch1-2 vs. *lrx1 pch1-1*	*0.423*
Figure 2C	
Col vs. lrx1	*<0.0001*
Col vs. lrx1 rol23	*0.0071*
Col vs. PP2C12-GFP #1	*<0.0001*
Col vs. PP2C12-GFP #2	*<0.0001*
Col vs. PP2C12-GFP #3	*<0.0001*
Col vs. PP2C15-GFP #1	*<0.0001*
Col vs. PP2C15-GFP #2	*<0.0001*
Col vs. PCH3-GFP #1	*<0.0001*
Col vs. PCH3-GFP #2	*<0.0001*
Col vs. PP2C38-GFP #1	*<0.0001*
Col vs. PP2C38-GFP #2	*<0.0001*
Col vs. PP2C35-GFP #1	*<0.0001*
Col vs. PP2C35-GFP #2	*<0.0001*
Col vs. PP2C52-GFP #1	*<0.0001*
Col vs. PP2C52-GFP #2	*<0.0001*
Col vs. EGR1-GFP	*<0.0001*
lrx1 vs. *lrx1 rol23*	*<0.0001*
lrx1 vs. PP2C12-GFP #1	*>0.9999*
lrx1 vs. PP2C12-GFP #2	*0.2772*
lrx1 vs. PP2C12-GFP #3	*0.9371*
lrx1 vs. PP2C15-GFP #1	*<0.0001*
lrx1 vs. PP2C15-GFP #2	*0.002*
lrx1 vs. PCH3-GFP #1	*0.1951*
lrx1 vs. PCH3-GFP #2	*<0.0001*
lrx1 vs. *PP2C38-GFP #1*	*<0.0001*
lrx1 vs. *PP2C38-GFP #2*	*<0.0001*
lrx1 vs. *PP2C35-GFP #1*	*<0.0001*
lrx1 vs. *PP2C35-GFP #2*	*<0.0001*
lrx1 vs. *PP2C52-GFP #1*	*<0.0001*

Appendix

lrx1 vs. *PP2C52-GFP #2*	<0.0001
lrx1 vs. *EGR1-GFP*	<0.0001
lrx1 rol23 vs. PP2C12-GFP #1	<0.0001
lrx1 rol23 vs. PP2C12-GFP #2	<0.0001
lrx1 rol23 vs. PP2C12-GFP #3	<0.0001
lrx1 rol23 vs. PP2C15-GFP #1	<0.0001
lrx1 rol23 vs. PP2C15-GFP #2	<0.0001
lrx1 rol23 vs. PCH3-GFP #1	<0.0001
lrx1 rol23 vs. PCH3-GFP #2	<0.0001
lrx1 rol23 vs. *PP2C38-GFP #1*	0.1223
lrx1 rol23 vs. *PP2C38-GFP #2*	0.9664
lrx1 rol23 vs. *PP2C35-GFP #1*	0.6219
lrx1 rol23 vs. *PP2C35-GFP #2*	<0.0001
lrx1 rol23 vs. *PP2C52-GFP #1*	<0.0001
lrx1 rol23 vs. *PP2C52-GFP #2*	<0.0001
lrx1 rol23 vs. *EGR1-GFP*	0.7837
PP2C12-GFP #1 vs. PP2C12-GFP #2	0.2247
PP2C12-GFP #1 vs. PP2C12-GFP #3	0.8817
PP2C12-GFP #1 vs. PP2C15-GFP #1	<0.0001
PP2C12-GFP #1 vs. PP2C15-GFP #2	0.0006
PP2C12-GFP #1 vs. PCH3-GFP #1	0.1436
PP2C12-GFP #1 vs. PCH3-GFP #2	<0.0001
PP2C12-GFP #1 vs. PP2C38-GFP #1	<0.0001
PP2C12-GFP #1 vs. PP2C38-GFP #2	<0.0001
PP2C12-GFP #1 vs. PP2C35-GFP #1	<0.0001
PP2C12-GFP #1 vs. PP2C35-GFP #2	<0.0001
PP2C12-GFP #1 vs. PP2C52-GFP #1	<0.0001
PP2C12-GFP #1 vs. PP2C52-GFP #2	<0.0001
PP2C12-GFP #1 vs. EGR1-GFP	<0.0001
PP2C12-GFP #2 vs. PP2C12-GFP #3	0.0004
PP2C12-GFP #2 vs. PP2C15-GFP #1	<0.0001
PP2C12-GFP #2 vs. PP2C15-GFP #2	<0.0001
PP2C12-GFP #2 vs. PCH3-GFP #1	>0.9999
PP2C12-GFP #2 vs. PCH3-GFP #2	0.1621
PP2C12-GFP #2 vs. PP2C38-GFP #1	<0.0001
PP2C12-GFP #2 vs. PP2C38-GFP #2	<0.0001
PP2C12-GFP #2 vs. PP2C35-GFP #1	<0.0001
PP2C12-GFP #2 vs. PP2C35-GFP #2	<0.0001
PP2C12-GFP #2 vs. PP2C52-GFP #1	<0.0001
PP2C12-GFP #2 vs. PP2C52-GFP #2	0.0001
PP2C12-GFP #2 vs. EGR1-GFP	<0.0001
PP2C12-GFP #3 vs. PP2C15-GFP #1	0.0002
PP2C12-GFP #3 vs. PP2C15-GFP #2	0.3551
PP2C12-GFP #3 vs. PCH3-GFP #1	<0.0001
PP2C12-GFP #3 vs. PCH3-GFP #2	<0.0001

Appendix

PP2C12-GFP #3 vs. PP2C38-GFP #1	<0.0001
PP2C12-GFP #3 vs. PP2C38-GFP #2	<0.0001
PP2C12-GFP #3 vs. PP2C35-GFP #1	<0.0001
PP2C12-GFP #3 vs. PP2C35-GFP #2	<0.0001
PP2C12-GFP #3 vs. PP2C52-GFP #1	<0.0001
PP2C12-GFP #3 vs. PP2C52-GFP #2	<0.0001
PP2C12-GFP #3 vs. EGR1-GFP	<0.0001
PP2C15-GFP #1 vs. PP2C15-GFP #2	0.8957
PP2C15-GFP #1 vs. PCH3-GFP #1	<0.0001
PP2C15-GFP #1 vs. PCH3-GFP #2	<0.0001
PP2C15-GFP #1 vs. PP2C38-GFP #1	<0.0001
PP2C15-GFP #1 vs. PP2C38-GFP #2	<0.0001
PP2C15-GFP #1 vs. PP2C35-GFP #1	<0.0001
PP2C15-GFP #1 vs. PP2C35-GFP #2	<0.0001
PP2C15-GFP #1 vs. PP2C52-GFP #1	<0.0001
PP2C15-GFP #1 vs. PP2C52-GFP #2	<0.0001
PP2C15-GFP #1 vs. EGR1-GFP	<0.0001
PP2C15-GFP #2 vs. PCH3-GFP #1	<0.0001
PP2C15-GFP #2 vs. PCH3-GFP #2	<0.0001
PP2C15-GFP #2 vs. PP2C38-GFP #1	<0.0001
PP2C15-GFP #2 vs. PP2C38-GFP #2	<0.0001
PP2C15-GFP #2 vs. PP2C35-GFP #1	<0.0001
PP2C15-GFP #2 vs. PP2C35-GFP #2	<0.0001
PP2C15-GFP #2 vs. PP2C52-GFP #1	<0.0001
PP2C15-GFP #2 vs. PP2C52-GFP #2	<0.0001
PP2C15-GFP #2 vs. EGR1-GFP	<0.0001
PCH3-GFP #1 vs. PCH3-GFP #2	0.0693
PCH3-GFP #1 vs. PP2C38-GFP #1	<0.0001
PCH3-GFP #1 vs. PP2C38-GFP #2	<0.0001
PCH3-GFP #1 vs. PP2C35-GFP #1	<0.0001
PCH3-GFP #1 vs. PP2C35-GFP #2	<0.0001
PCH3-GFP #1 vs. PP2C52-GFP #1	<0.0001
PCH3-GFP #1 vs. PP2C52-GFP #2	<0.0001
PCH3-GFP #1 vs. EGR1-GFP	<0.0001
PCH3-GFP #2 vs. PP2C38-GFP #1	<0.0001
PCH3-GFP #2 vs. PP2C38-GFP #2	<0.0001
PCH3-GFP #2 vs. PP2C35-GFP #1	<0.0001
PCH3-GFP #2 vs. PP2C35-GFP #2	<0.0001
PCH3-GFP #2 vs. PP2C52-GFP #1	<0.0001
PCH3-GFP #2 vs. PP2C52-GFP #2	0.7545
PCH3-GFP #2 vs. EGR1-GFP	<0.0001
PP2C38-GFP #1 vs. PP2C38-GFP #2	0.9969
PP2C38-GFP #1 vs. PP2C35-GFP #1	>0.9999
PP2C38-GFP #1 vs. PP2C35-GFP #2	0.8322
PP2C38-GFP #1 vs. PP2C52-GFP #1	0.6834

Appendix

PP2C38-GFP #1 vs. PP2C52-GFP #2	<0.0001
PP2C38-GFP #1 vs. EGR1-GFP	>0.9999
PP2C38-GFP #2 vs. PP2C35-GFP #1	>0.9999
PP2C38-GFP #2 vs. PP2C35-GFP #2	0.116
PP2C38-GFP #2 vs. PP2C52-GFP #1	0.0679
PP2C38-GFP #2 vs. PP2C52-GFP #2	<0.0001
PP2C38-GFP #2 vs. EGR1-GFP	>0.9999
PP2C35-GFP #1 vs. PP2C35-GFP #2	0.1497
PP2C35-GFP #1 vs. PP2C52-GFP #1	0.0863
PP2C35-GFP #1 vs. PP2C52-GFP #2	<0.0001
PP2C35-GFP #1 vs. EGR1-GFP	>0.9999
PP2C35-GFP #2 vs. PP2C52-GFP #1	>0.9999
PP2C35-GFP #2 vs. PP2C52-GFP #2	0.022
PP2C35-GFP #2 vs. EGR1-GFP	0.2268
PP2C52-GFP #1 vs. PP2C52-GFP #2	0.0725
PP2C52-GFP #1 vs. EGR1-GFP	0.1395
PP2C52-GFP #2 vs. EGR1-GFP	<0.0001
Figure 3B	
Col vs. fer-5	<0.0001
Col vs. fer-5 rol23	<0.0001
fer-5 vs. fer-5 rol23	<0.0001
Figure 3C	
Col vs. lrx1	<0.0001
Col vs. lrx1 bak1-4	<0.0001
Col vs. lrx1 bak1-5	<0.0001
Col vs. lrx1 rol23	0.0037
Col vs. lrx1 rol23 bak1-4	<0.0001
Col vs. lrx1 rol23 bak1-5	0.1921
lrx1 vs. lrx1 bak1-4	<0.0001
lrx1 vs. lrx1 bak1-5	0.9996
lrx1 vs. lrx1 rol23	<0.0001
lrx1 vs. lrx1 rol23 bak1-4	<0.0001
lrx1 vs. lrx1 rol23 bak1-5	<0.0001
lrx1 bak1-4 vs. lrx1 bak1-5	0.0006
lrx1 bak1-4 vs. lrx1 rol23	<0.0001
lrx1 bak1-4 vs. lrx1 rol23 bak1-4	<0.0001
lrx1 bak1-4 vs. lrx1 rol23 bak1-5	<0.0001
lrx1 bak1-5 vs. lrx1 rol23	<0.0001
lrx1 bak1-5 vs. lrx1 rol23 bak1-4	<0.0001
lrx1 bak1-5 vs. lrx1 rol23 bak1-5	<0.0001
lrx1 rol23 vs. lrx1 rol23 bak1-4	>0.9999
lrx1 rol23 vs. lrx1 rol23 bak1-5	0.4872
lrx1 rol23 bak1-4 vs. lrx1 rol23 bak1-5	0.1041

Appendix

Figure 3D	
Col vs. rol23	*0.972*
Col vs. pch1-1	*>0.9999*
Col vs. rol23 pch2 pch3	*0.9991*
Col vs. Col; pPCH1:PCH1-GFP	*<0.0001*
Col vs. fer-5	*<0.0001*
Col vs. fer-5 rol23	*<0.0001*
Col vs. fer-4	*<0.0001*
Col vs. fer-4 rol23	*<0.0001*
rol23 vs. *pch1-1*	*0.9951*
rol23 vs. *rol23 pch2 pch3*	*0.7099*
rol23 vs. *Col; pPCH1:PCH1-GFP*	*<0.0001*
rol23 vs. *fer-5*	*<0.0001*
rol23 vs. *fer-5 rol23*	*<0.0001*
rol23 vs. *fer-4*	*<0.0001*
rol23 vs. *fer-4 rol23*	*<0.0001*
pch1-1 vs. *rol23 pch2 pch3*	*0.9674*
pch1-1 vs. *Col; pPCH1:PCH1-GFP*	*<0.0001*
pch1-1 vs. *fer-5*	*<0.0001*
pch1-1 vs. *fer-5 rol23*	*<0.0001*
pch1-1 vs. *fer-4*	*<0.0001*
pch1-1 vs. *fer-4 rol23*	*<0.0001*
rol23 pch2 pch3 vs. *Col; pPCH1:PCH1-GFP*	*<0.0001*
rol23 pch2 pch3 vs. *fer-5*	*<0.0001*
rol23 pch2 pch3 vs. *fer-5 rol23*	*<0.0001*
rol23 pch2 pch3 vs. *fer-4*	*<0.0001*
rol23 pch2 pch3 vs. *fer-4 rol23*	*<0.0001*
Col; pPCH1:PCH1-GFP vs. *fer-5*	*0.6619*
Col; pPCH1:PCH1-GFP vs. *fer-5 rol23*	*0.0996*
Col; pPCH1:PCH1-GFP vs. *fer-4*	*<0.0001*
Col; pPCH1:PCH1-GFP vs. *fer-4 rol23*	*<0.0001*
fer-5 vs. *fer-5 rol23*	*0.967*
fer-5 vs. *fer-4*	*<0.0001*
fer-5 vs. *fer-4 rol23*	*0.0094*
fer-5 rol23 vs. *fer-4*	*<0.0001*
fer-5 rol23 vs. *fer-4 rol23*	*0.1652*
fer-4 vs. *fer-4 rol23*	*0.2319*

Figure 3E	
Col vs. pch1-2 pch2 pch3	*0.0009*
Col vs. rol23 pch2 pch3	*0.8283*
Col vs. fer-4	*<0.0001*
Col vs. fer-4 rol23 pch2 pch3	*<0.0001*
Col vs. fer-5	*<0.0001*

Appendix

Col vs. fer-5 rol23 pch2 pch3	*0.042*
pch1-2 pch2 pch3 vs. *rol23 pch2 pch3*	*<0.0001*
pch1-2 pch2 pch3 vs. *fer-4*	*<0.0001*
pch1-2 pch2 pch3 vs. *fer-4 rol23 pch2 pch3*	*<0.0001*
pch1-2 pch2 pch3 vs. *fer-5*	*<0.0001*
pch1-2 pch2 pch3 vs. *fer-5 rol23 pch2 pch3*	*<0.0001*
rol23 pch2 pch3 vs. *fer-4*	*<0.0001*
rol23 pch2 pch3 vs. *fer-4 rol23 pch2 pch3*	*<0.0001*
rol23 pch2 pch3 vs. *fer-5*	*<0.0001*
rol23 pch2 pch3 vs. *fer-5 rol23 pch2 pch3*	*0.5865*
fer-4 vs. *fer-4 rol23 pch2 pch3*	*>0.9999*
fer-4 vs. *fer-5*	*<0.0001*
fer-4 vs. *fer-5 rol23 pch2 pch3*	*<0.0001*
fer-4 rol23 pch2 pch3 vs. *fer-5*	*<0.0001*
fer-4 rol23 pch2 pch3 vs. *fer-5 rol23 pch2 pch3*	*<0.0001*
fer-5 vs. *fer-5 rol23 pch2 pch3*	*<0.0001*
Figure 4D	
Col vs. lrx1	*<0.0001*
Col vs. lrx1 rol23	*<0.0001*
Col vs. lrx1 rol23; PP2C12-GFP T2_1	*<0.0001*
Col vs. lrx1 rol23; PP2C12-GFP T2_2	*<0.0001*
Col vs. lrx1 rol23; PP2C12-GFP T2_4	*<0.0001*
Col vs. lrx1 rol23; PP2C12dead-GFP T2_1	*0.9939*
Col vs. lrx1 rol23; PP2C12dead-GFP T2_2	*>0.9999*
lrx1 vs. lrx1 rol23	*<0.0001*
lrx1 vs. lrx1 rol23; PP2C12-GFP T2_1	*>0.9999*
lrx1 vs. lrx1 rol23; PP2C12-GFP T2_2	*0.0357*
lrx1 vs. lrx1 rol23; PP2C12-GFP T2_4	*>0.9999*
lrx1 vs. lrx1 rol23; PP2C12dead-GFP T2_1	*<0.0001*
lrx1 vs. lrx1 rol23; PP2C12dead-GFP T2_2	*<0.0001*
lrx1 rol23 vs. *lrx1 rol23; PP2C12-GFP T2_1*	*<0.0001*
lrx1 rol23 vs. *lrx1 rol23; PP2C12-GFP T2_2*	*<0.0001*
lrx1 rol23 vs. *lrx1 rol23; PP2C12-GFP T2_4*	*<0.0001*
lrx1 rol23 vs. lrx1 rol23; PP2C12dead-GFP T2_1	*<0.0001*
lrx1 rol23 vs. lrx1 rol23; PP2C12dead-GFP T2_2	*<0.0001*
lrx1 rol23; PP2C12-GFP T2_1 vs. *lrx1 rol23; PP2C12-GFP T2_2*	*0.0412*
lrx1 rol23; PP2C12-GFP T2_1 vs. *lrx1 rol23; PP2C12-GFP T2_4*	*>0.9999*
lrx1 rol23; PP2C12-GFP T2_1 vs. *lrx1 rol23; PP2C12dead-GFP T2_1*	*<0.0001*
lrx1 rol23; PP2C12-GFP T2_1 vs. *lrx1 rol23; PP2C12dead-GFP T2_2*	*<0.0001*
lrx1 rol23; PP2C12-GFP T2_2 vs. *lrx1 rol23; PP2C12-GFP T2_4*	*0.0843*
lrx1 rol23; PP2C12-GFP T2_2 vs. *lrx1 rol23; PP2C12dead-GFP T2_1*	*<0.0001*
lrx1 rol23; PP2C12-GFP T2_2 vs. *lrx1 rol23; PP2C12dead-GFP T2_2*	*<0.0001*
lrx1 rol23; PP2C12-GFP T2_4 vs. *lrx1 rol23; PP2C12dead-GFP T2_1*	*<0.0001*
lrx1 rol23; PP2C12-GFP T2_4 vs. *lrx1 rol23; PP2C12dead-GFP T2_2*	*<0.0001*

lrx1 rol23; PP2C12dead-GFP T2_1 vs. *lrx1 rol23; PP2C12dead-GFP T2_1*	*0.9965*
Figure 7D	
Col vs. lrx1	*<0.0001*
Col vs. lrx1 fer-4	*<0.0001*
Col vs. lrx1fer-4, FER T696_1	*<0.0001*
Col vs. lrx1fer-4, FER T696_2	*<0.0001*
Col vs. lrx1fer-4, FER T696_7	*<0.0001*
Col vs. lrx1fer-4, FER T696_9	*<0.0001*
Col vs. lrx1fer-4, FER T696A_1	*<0.0001*
Col vs. lrx1fer-4, FER T696A_2	*<0.0001*
Col vs. lrx1fer-4, FER T696A_3	*<0.0001*
Col vs. lrx1fer-4, FER T696D_1	*<0.0001*
Col vs. lrx1fer-4, FER T696D_2	*<0.0001*
Col vs. lrx1fer-4, FER T696D_3	*<0.0001*
Col vs. lrx1fer-4, FER T696E_1	*<0.0001*
Col vs. lrx1fer-4, FER T696E_2	*<0.0001*
Col vs. lrx1fer-4, FER T696E_3	*<0.0001*
lrx1 vs. lrx1 fer-4	*<0.0001*
lrx1 vs. lrx1fer-4, FER T696_1	*<0.0001*
lrx1 vs. lrx1fer-4, FER T696_2	*<0.0001*
lrx1 vs. lrx1fer-4, FER T696_7	*<0.0001*
lrx1 vs. lrx1fer-4, FER T696_9	*<0.0001*
lrx1 vs. lrx1fer-4, FER T696A_1	*<0.0001*
lrx1 vs. lrx1fer-4, FER T696A_2	*<0.0001*
lrx1 vs. lrx1fer-4, FER T696A_3	*<0.0001*
lrx1 vs. lrx1fer-4, FER T696D_1	*<0.0001*
lrx1 vs. lrx1fer-4, FER T696D_2	*<0.0001*
lrx1 vs. lrx1fer-4, FER T696D_3	*<0.0001*
lrx1 vs. lrx1fer-4, FER T696E_1	*<0.0001*
lrx1 vs. lrx1fer-4, FER T696E_2	*<0.0001*
lrx1 vs. lrx1fer-4, FER T696E_3	*<0.0001*
lrx1 fer-4 vs. lrx1fer-4, FER T696_1	*<0.0001*
lrx1 fer-4 vs. lrx1fer-4, FER T696_2	*<0.0001*
lrx1 fer-4 vs. lrx1fer-4, FER T696_7	*<0.0001*
lrx1 fer-4 vs. lrx1fer-4, FER T696_9	*<0.0001*
lrx1 fer-4 vs. lrx1fer-4, FER T696A_1	*<0.0001*
lrx1 fer-4 vs. lrx1fer-4, FER T696A_2	*0.0025*
lrx1 fer-4 vs. lrx1fer-4, FER T696A_3	*<0.0001*
lrx1 fer-4 vs. lrx1fer-4, FER T696D_1	*<0.0001*
lrx1 fer-4 vs. lrx1fer-4, FER T696D_2	*<0.0001*
lrx1 fer-4 vs. lrx1fer-4, FER T696D_3	*<0.0001*
lrx1 fer-4 vs. lrx1fer-4, FER T696E_1	*<0.0001*
lrx1 fer-4 vs. lrx1fer-4, FER T696E_2	*<0.0001*
lrx1 fer-4 vs. lrx1fer-4, FER T696E_3	*<0.0001*

Appendix

lrx1fer-4, FER T696_1 vs. lrx1fer-4, FER T696_2	*0.3001*
lrx1fer-4, FER T696_1 vs. lrx1fer-4, FER T696_7	*<0.0001*
lrx1fer-4, FER T696_1 vs. lrx1fer-4, FER T696_9	*<0.0001*
lrx1fer-4, FER T696_1 vs. lrx1fer-4, FER T696A_1	*<0.0001*
lrx1fer-4, FER T696_1 vs. lrx1fer-4, FER T696A_2	*<0.0001*
lrx1fer-4, FER T696_1 vs. lrx1fer-4, FER T696A_3	*<0.0001*
lrx1fer-4, FER T696_1 vs. lrx1fer-4, FER T696D_1	*0.8785*
lrx1fer-4, FER T696_1 vs. lrx1fer-4, FER T696D_2	*<0.0001*
lrx1fer-4, FER T696_1 vs. lrx1fer-4, FER T696D_3	*<0.0001*
lrx1fer-4, FER T696_1 vs. lrx1fer-4, FER T696E_1	*0.0003*
lrx1fer-4, FER T696_1 vs. lrx1fer-4, FER T696E_2	*0.0001*
lrx1fer-4, FER T696_1 vs. lrx1fer-4, FER T696E_3	*0.0156*
lrx1fer-4, FER T696_2 vs. lrx1fer-4, FER T696_7	*0.5191*
lrx1fer-4, FER T696_2 vs. lrx1fer-4, FER T696_9	*0.1692*
lrx1fer-4, FER T696_2 vs. lrx1fer-4, FER T696A_1	*0.0002*
lrx1fer-4, FER T696_2 vs. lrx1fer-4, FER T696A_2	*<0.0001*
lrx1fer-4, FER T696_2 vs. lrx1fer-4, FER T696A_3	*<0.0001*
lrx1fer-4, FER T696_2 vs. lrx1fer-4, FER T696D_1	*>0.9999*
lrx1fer-4, FER T696_2 vs. lrx1fer-4, FER T696D_2	*<0.0001*
lrx1fer-4, FER T696_2 vs. lrx1fer-4, FER T696D_3	*0.0023*
lrx1fer-4, FER T696_2 vs. lrx1fer-4, FER T696E_1	*0.8591*
lrx1fer-4, FER T696_2 vs. lrx1fer-4, FER T696E_2	*0.7265*
lrx1fer-4, FER T696_2 vs. lrx1fer-4, FER T696E_3	*0.9999*
lrx1fer-4, FER T696_7 vs. lrx1fer-4, FER T696_9	*>0.9999*
lrx1fer-4, FER T696_7 vs. lrx1fer-4, FER T696A_1	*0.5731*
lrx1fer-4, FER T696_7 vs. lrx1fer-4, FER T696A_2	*0.0003*
lrx1fer-4, FER T696_7 vs. lrx1fer-4, FER T696A_3	*0.4558*
lrx1fer-4, FER T696_7 vs. lrx1fer-4, FER T696D_1	*0.1947*
lrx1fer-4, FER T696_7 vs. lrx1fer-4, FER T696D_2	*0.2803*
lrx1fer-4, FER T696_7 vs. lrx1fer-4, FER T696D_3	*0.8505*
lrx1fer-4, FER T696_7 vs. lrx1fer-4, FER T696E_1	*>0.9999*
lrx1fer-4, FER T696_7 vs. lrx1fer-4, FER T696E_2	*>0.9999*
lrx1fer-4, FER T696_7 vs. lrx1fer-4, FER T696E_3	*0.9801*
lrx1fer-4, FER T696_9 vs. lrx1fer-4, FER T696A_1	*0.918*
lrx1fer-4, FER T696_9 vs. lrx1fer-4, FER T696A_2	*0.003*
lrx1fer-4, FER T696_9 vs. lrx1fer-4, FER T696A_3	*0.8514*
lrx1fer-4, FER T696_9 vs. lrx1fer-4, FER T696D_1	*0.0449*
lrx1fer-4, FER T696_9 vs. lrx1fer-4, FER T696D_2	*0.6767*
lrx1fer-4, FER T696_9 vs. lrx1fer-4, FER T696D_3	*0.99*
lrx1fer-4, FER T696_9 vs. lrx1fer-4, FER T696E_1	*0.9992*
lrx1fer-4, FER T696_9 vs. lrx1fer-4, FER T696E_2	*>0.9999*
lrx1fer-4, FER T696_9 vs. lrx1fer-4, FER T696E_3	*0.7647*
lrx1fer-4, FER T696A_1 vs. lrx1fer-4, FER T696A_2	*0.4756*
lrx1fer-4, FER T696A_1 vs. lrx1fer-4, FER T696A_3	*>0.9999*
lrx1fer-4, FER T696A_1 vs. lrx1fer-4, FER T696D_1	*<0.0001*

lrx1fer-4, FER T696A_1 vs. lrx1fer-4, FER T696D_2	>0.9999
lrx1fer-4, FER T696A_1 vs. lrx1fer-4, FER T696D_3	>0.9999
lrx1fer-4, FER T696A_1 vs. lrx1fer-4, FER T696E_1	0.1823
lrx1fer-4, FER T696A_1 vs. lrx1fer-4, FER T696E_2	0.36
lrx1fer-4, FER T696A_1 vs. lrx1fer-4, FER T696E_3	0.0075
lrx1fer-4, FER T696A_2 vs. lrx1fer-4, FER T696A_3	0.5916
lrx1fer-4, FER T696A_2 vs. lrx1fer-4, FER T696D_1	<0.0001
lrx1fer-4, FER T696A_2 vs. lrx1fer-4, FER T696D_2	0.8544
lrx1fer-4, FER T696A_2 vs. lrx1fer-4, FER T696D_3	0.4004
lrx1fer-4, FER T696A_2 vs. lrx1fer-4, FER T696E_1	<0.0001
lrx1fer-4, FER T696A_2 vs. lrx1fer-4, FER T696E_2	<0.0001
lrx1fer-4, FER T696A_2 vs. lrx1fer-4, FER T696E_3	<0.0001
lrx1fer-4, FER T696A_3 vs. lrx1fer-4, FER T696D_1	<0.0001
lrx1fer-4, FER T696A_3 vs. lrx1fer-4, FER T696D_2	>0.9999
lrx1fer-4, FER T696A_3 vs. lrx1fer-4, FER T696D_3	>0.9999
lrx1fer-4, FER T696A_3 vs. lrx1fer-4, FER T696E_1	0.1215
lrx1fer-4, FER T696A_3 vs. lrx1fer-4, FER T696E_2	0.2632
lrx1fer-4, FER T696A_3 vs. lrx1fer-4, FER T696E_3	0.004
lrx1fer-4, FER T696D_1 vs. lrx1fer-4, FER T696D_2	<0.0001
lrx1fer-4, FER T696D_1 vs. lrx1fer-4, FER T696D_3	0.0004
lrx1fer-4, FER T696D_1 vs. lrx1fer-4, FER T696E_1	0.4828
lrx1fer-4, FER T696D_1 vs. lrx1fer-4, FER T696E_2	0.3404
lrx1fer-4, FER T696D_1 vs. lrx1fer-4, FER T696E_3	0.9679
lrx1fer-4, FER T696D_2 vs. lrx1fer-4, FER T696D_3	>0.9999
lrx1fer-4, FER T696D_2 vs. lrx1fer-4, FER T696E_1	0.0598
lrx1fer-4, FER T696D_2 vs. lrx1fer-4, FER T696E_2	0.1446
lrx1fer-4, FER T696D_2 vs. lrx1fer-4, FER T696E_3	0.0016
lrx1fer-4, FER T696D_3 vs. lrx1fer-4, FER T696E_1	0.4661
lrx1fer-4, FER T696D_3 vs. lrx1fer-4, FER T696E_2	0.6802
lrx1fer-4, FER T696D_3 vs. lrx1fer-4, FER T696E_3	0.049
lrx1fer-4, FER T696E_1 vs. lrx1fer-4, FER T696E_2	>0.9999
lrx1fer-4, FER T696E_1 vs. lrx1fer-4, FER T696E_3	0.9998
lrx1fer-4, FER T696E_2 vs. lrx1fer-4, FER T696E_3	0.9977
**Figure 7F**	
Col vs. lrx1	<0.0001
Col vs. lrx1 fer-4	<0.0001
Col vs. lrx1fer-4, FER_WT_1 cntrl	<0.0001
Col vs. lrx1fer-4, FER_WT_1 pch1_2	<0.0001
Col vs. lrx1fer-4, FER_TA_1 cntrl	<0.0001
Col vs. lrx1fer-4, FER_TA_1 pch1_2	<0.0001
Col vs. lrx1fer-4, FER_TA_2 cntrl	<0.0001
Col vs. lrx1fer-4, FER_TA_2 pch1_1	<0.0001
lrx1 vs. lrx1 fer-4	<0.0001
lrx1 vs. lrx1fer-4, FER_WT_1 cntrl	0.935

lrx1 vs. lrx1fer-4, FER_WT_1 pch1_2	*<0.0001*
lrx1 vs. lrx1fer-4, FER_TA_1 cntrl	*0.008*
lrx1 vs. lrx1fer-4, FER_TA_1 pch1_2	*0.8651*
lrx1 vs. lrx1fer-4, FER_TA_2 cntrl	*0.755*
lrx1 vs. lrx1fer-4, FER_TA_2 pch1_1	*0.0673*
lrx1 fer-4 vs. lrx1fer-4, FER_WT_1 cntrl	*<0.0001*
lrx1 fer-4 vs. lrx1fer-4, FER_WT_1 pch1_2	*<0.0001*
lrx1 fer-4 vs. lrx1fer-4, FER_TA_1 cntrl	*0.001*
lrx1 fer-4 vs. lrx1fer-4, FER_TA_1 pch1_2	*<0.0001*
lrx1 fer-4 vs. lrx1fer-4, FER_TA_2 cntrl	*<0.0001*
lrx1 fer-4 vs. lrx1fer-4, FER_TA_2 pch1_1	*<0.0001*
lrx1fer-4, FER_WT_1 cntrl vs. lrx1fer-4, FER_WT_1 pch1_2	*<0.0001*
lrx1fer-4, FER_WT_1 cntrl vs. lrx1fer-4, FER_TA_1 cntrl	*0.2594*
lrx1fer-4, FER_WT_1 cntrl vs. lrx1fer-4, FER_TA_1 pch1_2	*>0.9999*
lrx1fer-4, FER_WT_1 cntrl vs. lrx1fer-4, FER_TA_2 cntrl	*>0.9999*
lrx1fer-4, FER_WT_1 cntrl vs. lrx1fer-4, FER_TA_2 pch1_1	*0.0006*
lrx1fer-4, FER_WT_1 pch1_2 vs. lrx1fer-4, FER_TA_1 cntrl	*<0.0001*
lrx1fer-4, FER_WT_1 pch1_2 vs. lrx1fer-4, FER_TA_1 pch1_2	*<0.0001*
lrx1fer-4, FER_WT_1 pch1_2 vs. lrx1fer-4, FER_TA_2 cntrl	*<0.0001*
lrx1fer-4, FER_WT_1 pch1_2 vs. lrx1fer-4, FER_TA_2 pch1_1	*<0.0001*
lrx1fer-4, FER_TA_1 cntrl vs. lrx1fer-4, FER_TA_1 pch1_2	*0.4052*
lrx1fer-4, FER_TA_2 cntrl vs. lrx1fer-4, FER_TA_2 cntrl	*0.5753*
lrx1fer-4, FER_TA_2 cntrl vs. lrx1fer-4, FER_TA_2 pch1_1	*<0.0001*
lrx1fer-4, FER_TA_2 pch1_2 vs. lrx1fer-4, FER_TA_2 cntrl	*>0.9999*
lrx1fer-4, FER_TA_2 pch1_2 vs. lrx1fer-4, FER_TA_2 pch1_1	*0.0003*
lrx1fer-4, FER_TA_2 cntrl vs. lrx1fer-4, FER_TA_2 pch1_1	0.0001
**Figure 8B**	
> 0.10 µm2	
fer-4 FER-GFP vs. *fer-4 FER-GFP rol23 pch2 pch3*	*<0.0001*
fer-4 FER-GFP vs. *fer-4 FER-GFP rol23 pch2 pch3*, 50 mM EGCG	*0.7006*
fer-4 FER-GFP rol23 pch2 pch3 vs. *fer-4 FER-GFP rol23 pch2 pch3*, 50 mM EGCG	*<0.0001*
> 0.25 µm2	
fer-4 FER-GFP vs. *fer-4 FER-GFP rol23 pch2 pch3*	*<0.0001*
fer-4 FER-GFP vs. *fer-4 FER-GFP rol23 pch2 pch3*, 50 mM EGCG	*0.7419*
fer-4 FER-GFP rol23 pch2 pch3 vs. *fer-4 FER-GFP rol23 pch2 pch3*, 50 mM EGCG	*<0.0001*
> 0.5 µm2	
fer-4 FER-GFP vs. *fer-4 FER-GFP rol23 pch2 pch3*	*<0.0001*
fer-4 FER-GFP vs. *fer-4 FER-GFP rol23 pch2 pch3*, 50 mM EGCG	*0.5267*
fer-4 FER-GFP rol23 pch2 pch3 vs. *fer-4 FER-GFP rol23 pch2 pch3*, 50 mM EGCG	*<0.0001*
