# Supplementary figures and images for: The Arabidopsis phosphatase PP2C12 negatively regulates LRX-RALF-FER-mediated cell wall integrity sensing (part 1 of 2)
Source: EMBO J. 2025 Nov 17;45(1):243–60. doi: 10.1038/s44318-025-00614-x (PMC12759080; doi:10.1038/s44318-025-00614-x)

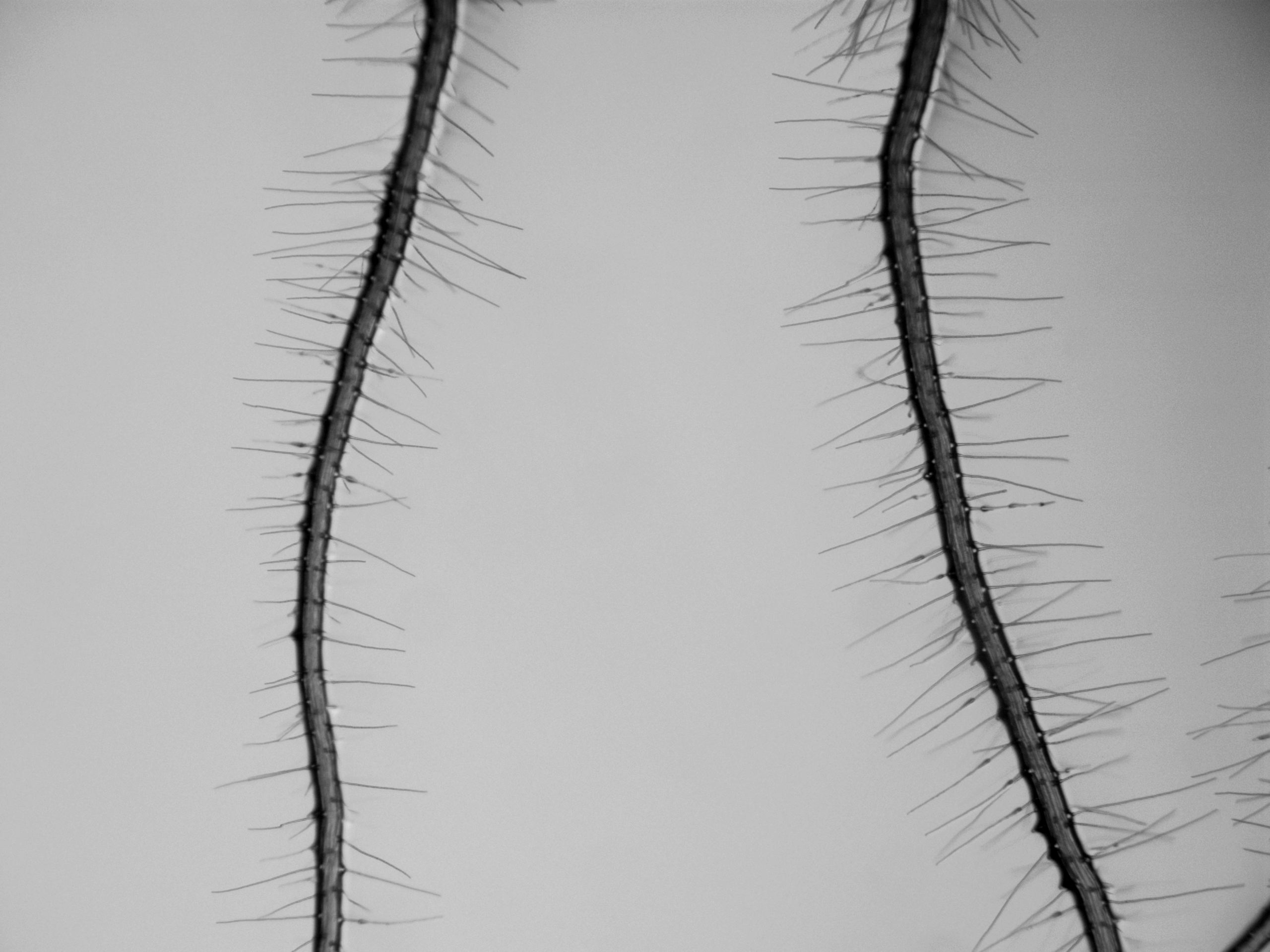

Supplement: Supplementary file 3 — Source data Fig. 1 [file 44318_2025_614_MOESM3_ESM.zip › Fig 1/Fig 1A/Col.tif]

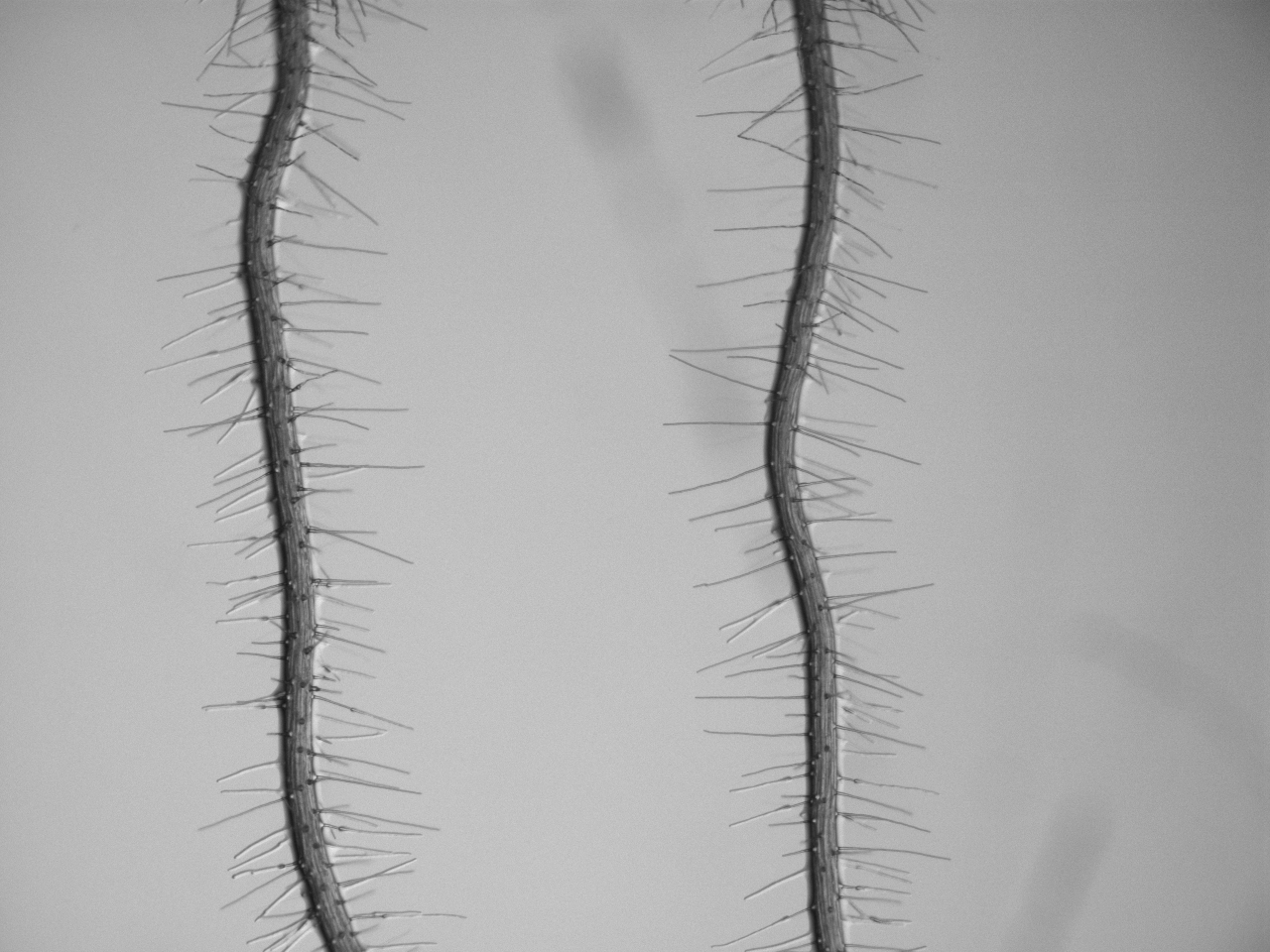

Supplement: Supplementary file 3 — Source data Fig. 1 [file 44318_2025_614_MOESM3_ESM.zip › Fig 1/Fig 1A/lrx1 pp2c12_2.tif]

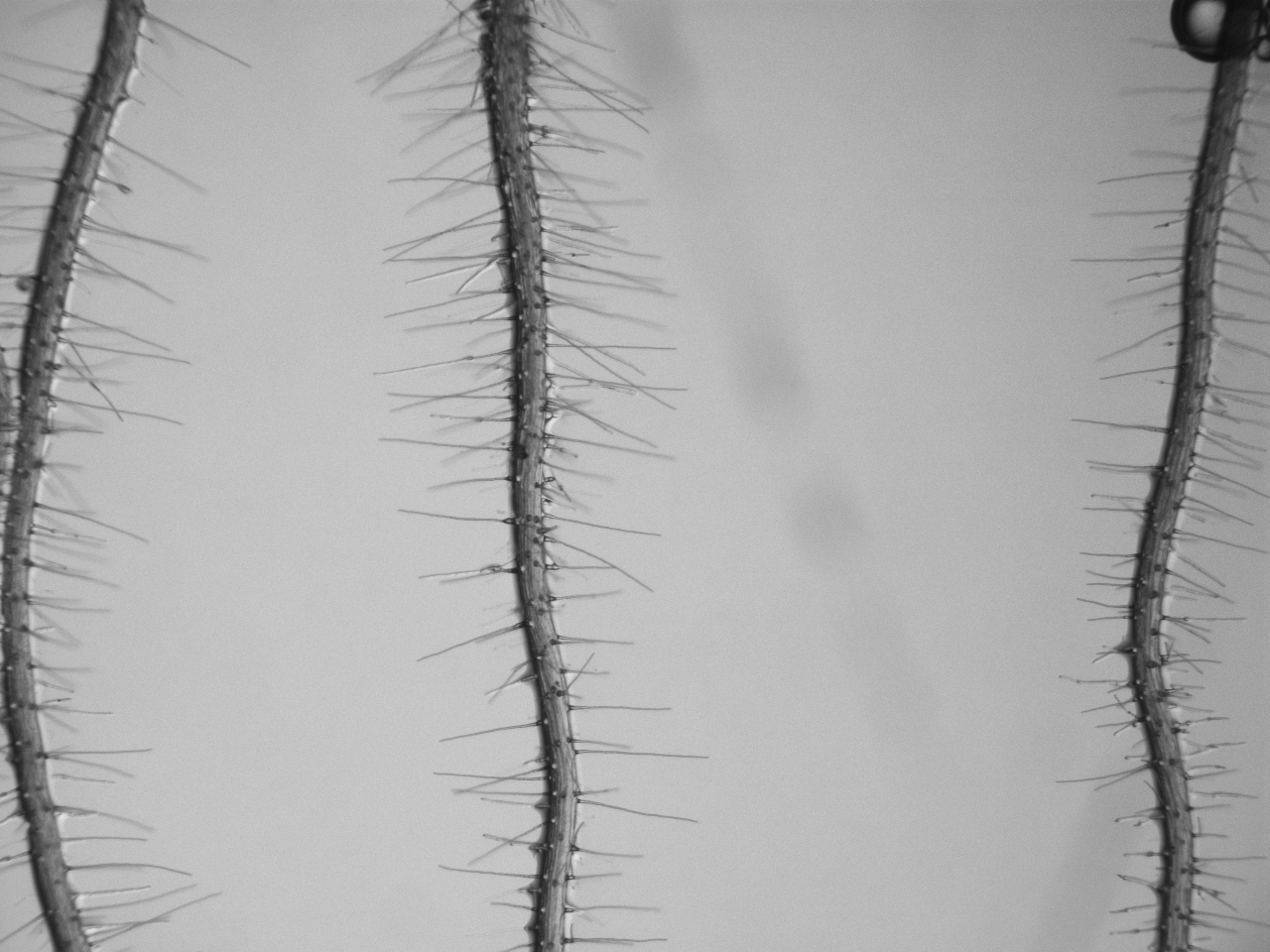

Supplement: Supplementary file 3 — Source data Fig. 1 [file 44318_2025_614_MOESM3_ESM.zip › Fig 1/Fig 1A/lrx1 pp2c12_3.tif]

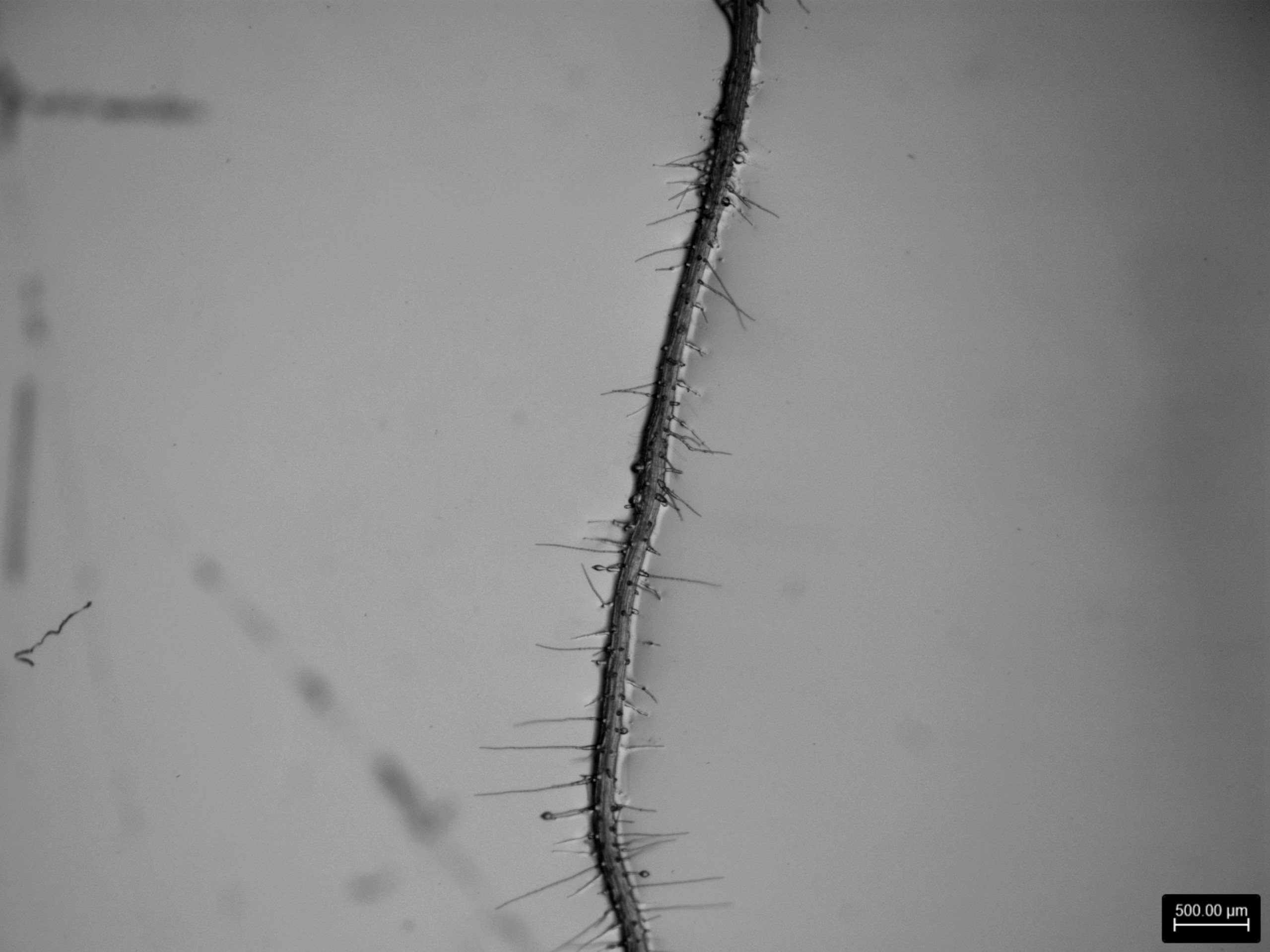

Supplement: Supplementary file 3 — Source data Fig. 1 [file 44318_2025_614_MOESM3_ESM.zip › Fig 1/Fig 1A/lrx1_6.jpg]

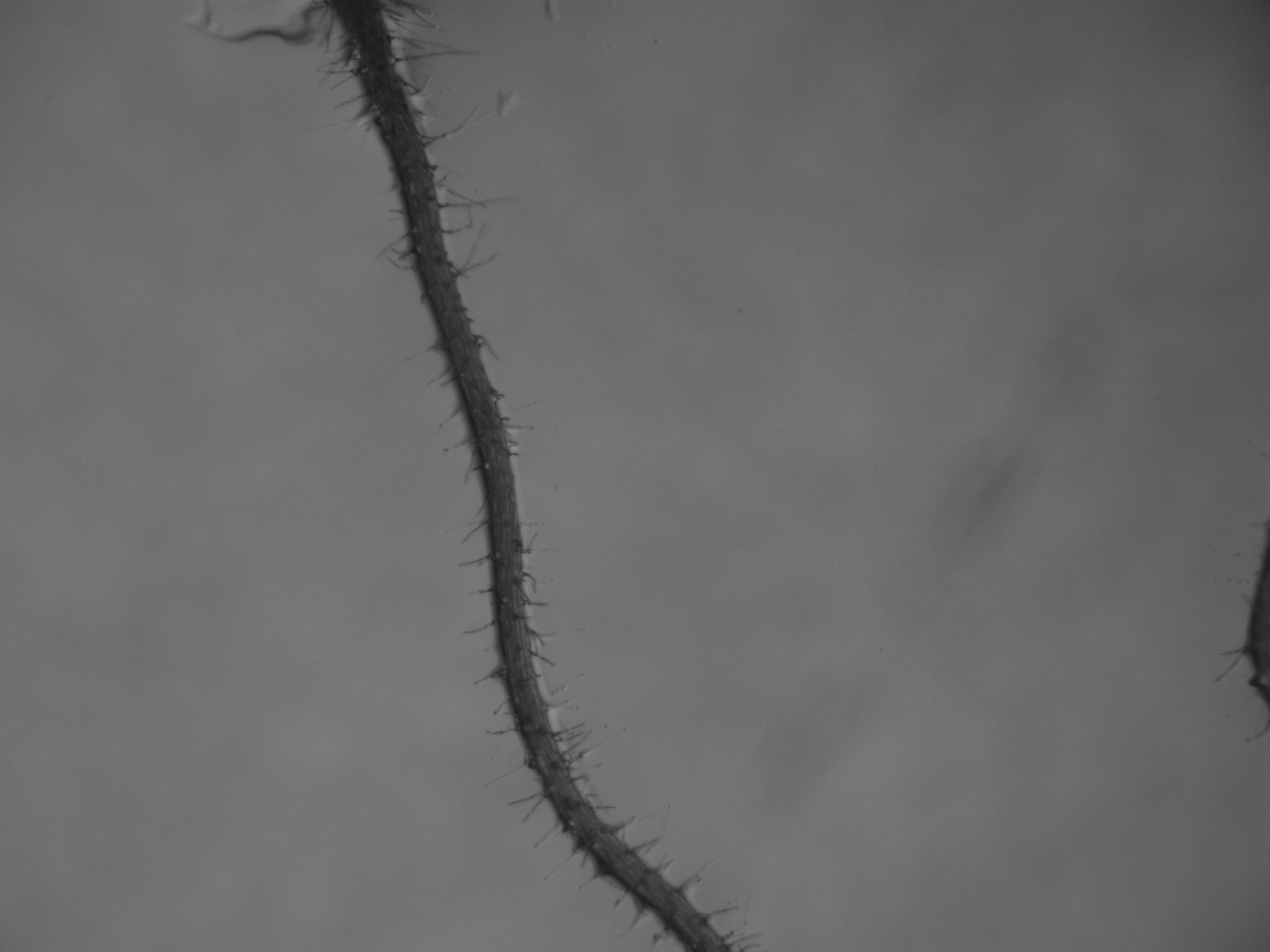

Supplement: Supplementary file 3 — Source data Fig. 1 [file 44318_2025_614_MOESM3_ESM.zip › Fig 1/Fig 1A/lrx1rol23 x lrx1 F1.tif]

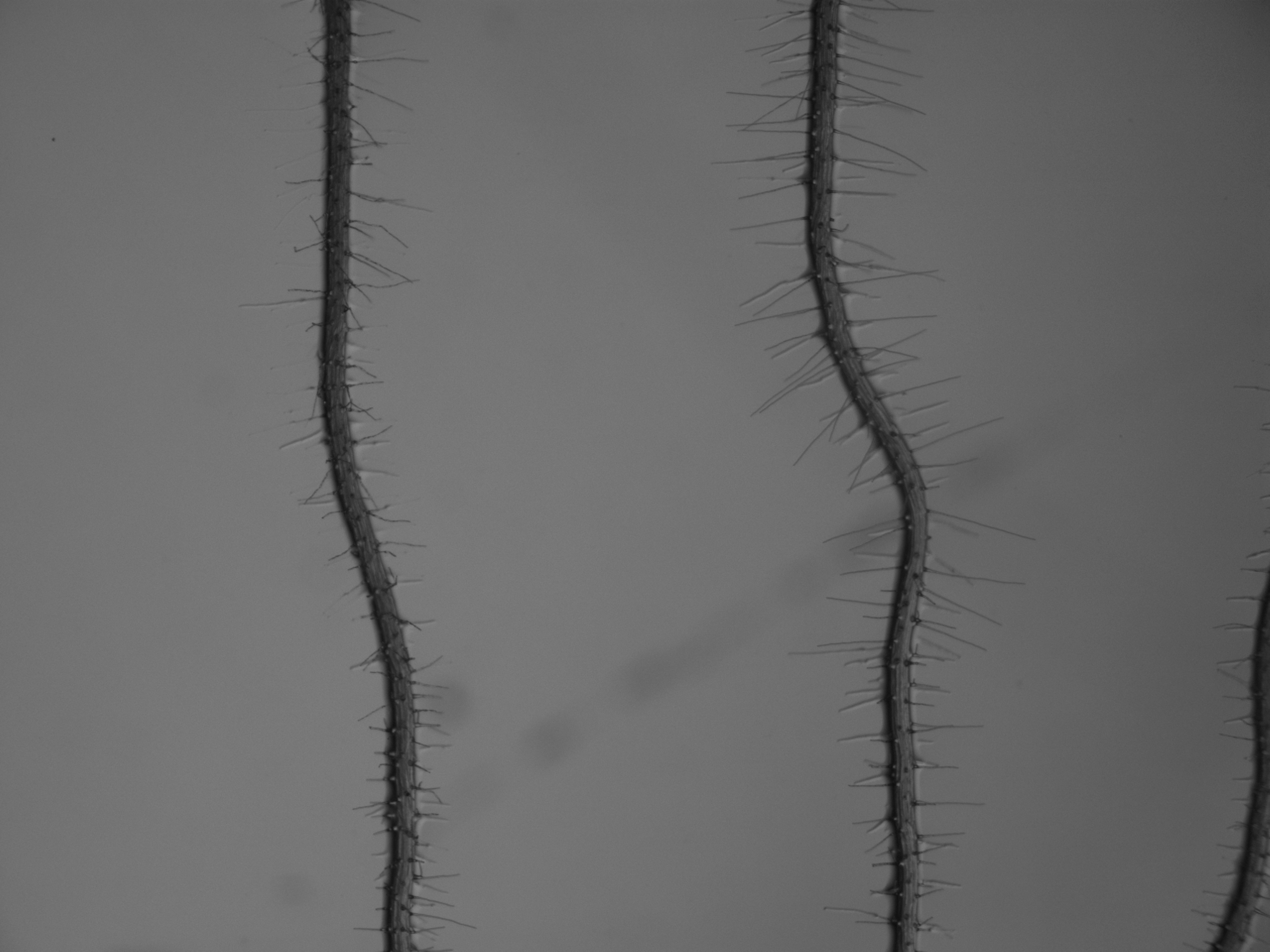

Supplement: Supplementary file 3 — Source data Fig. 1 [file 44318_2025_614_MOESM3_ESM.zip › Fig 1/Fig 1A/lrx1rol23_2.tif]

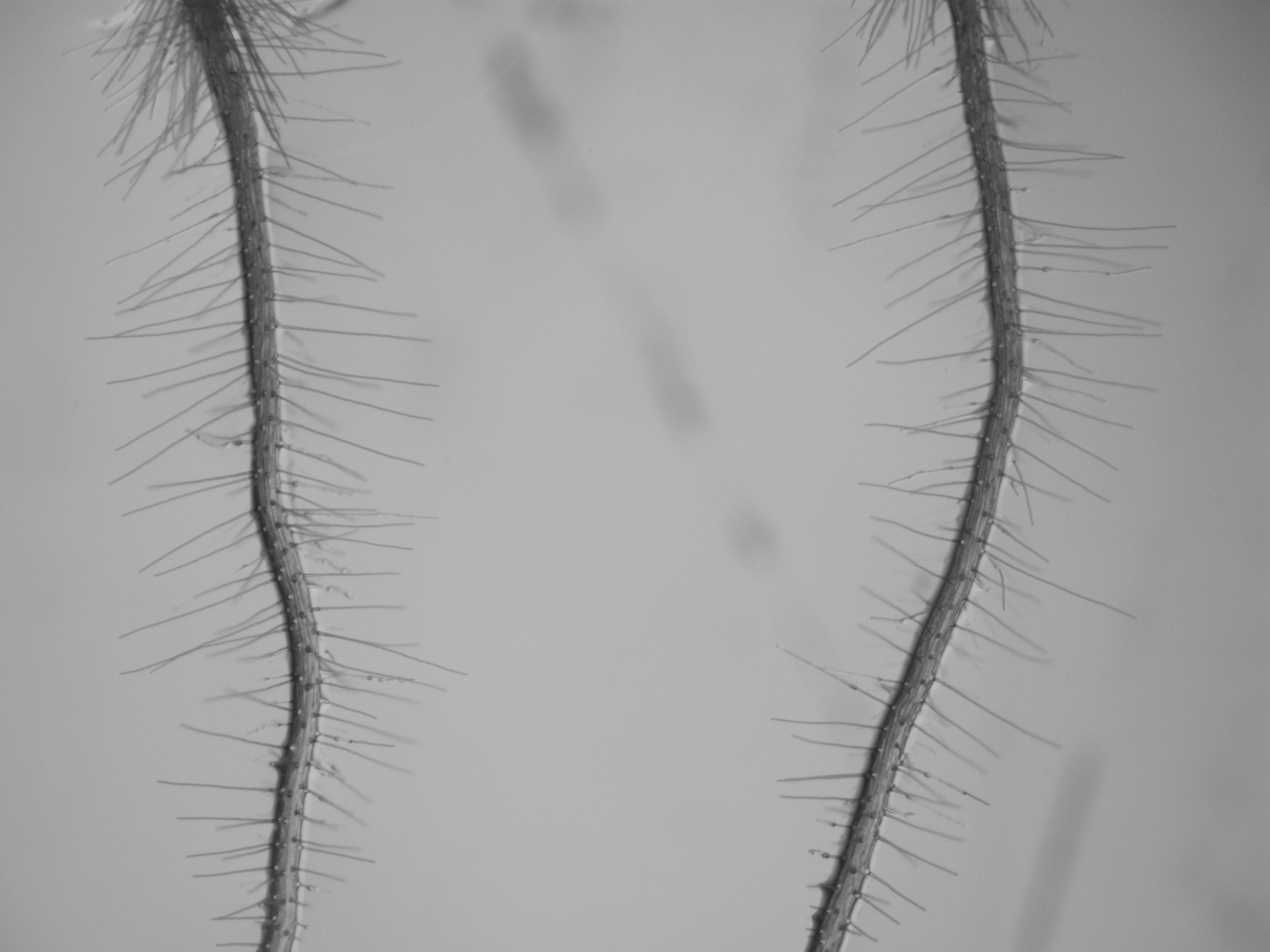

Supplement: Supplementary file 4 — Source data Fig. 2 [file 44318_2025_614_MOESM4_ESM.zip › Fig 2/Fig 2A/Col.tif]

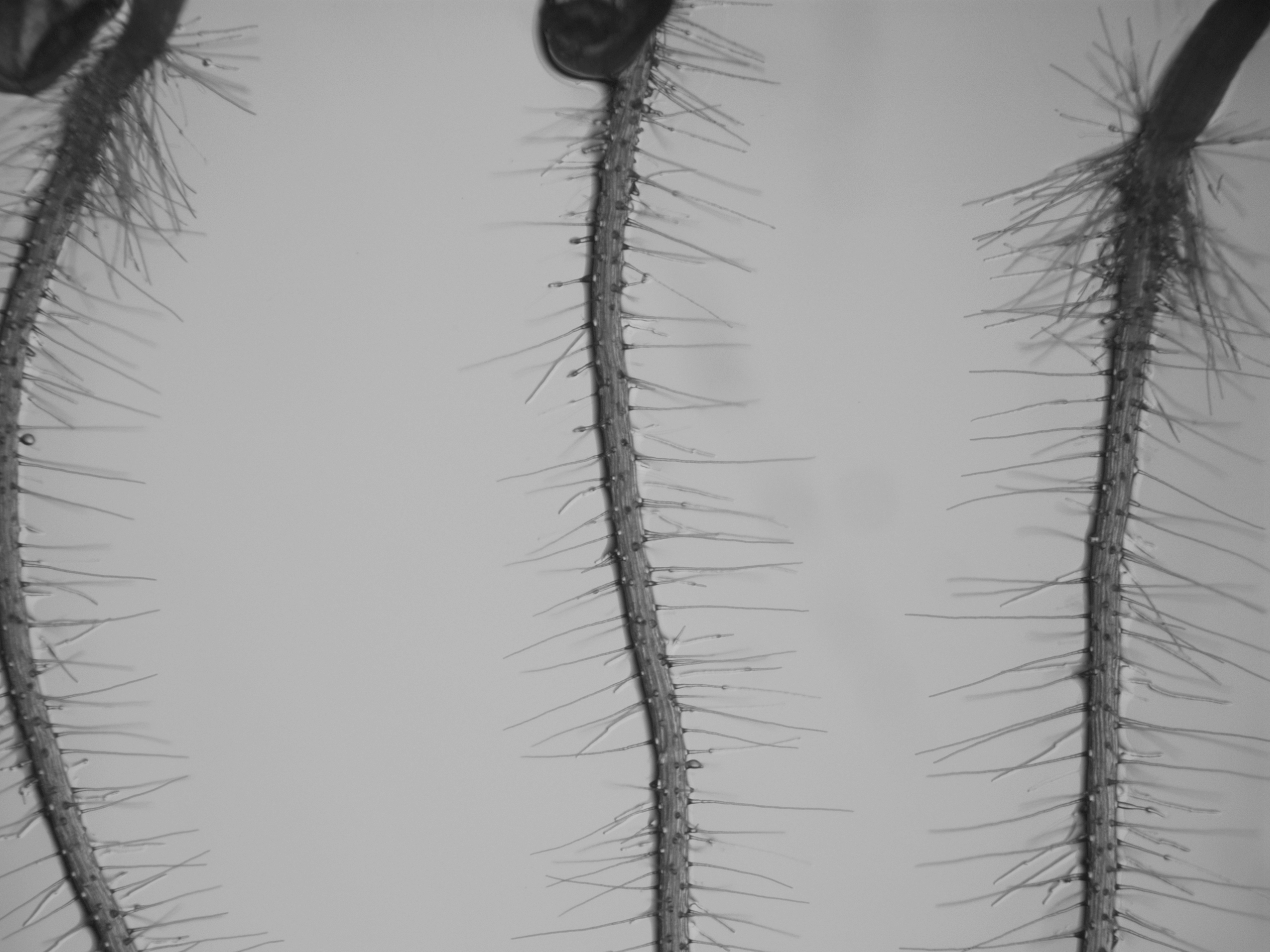

Supplement: Supplementary file 4 — Source data Fig. 2 [file 44318_2025_614_MOESM4_ESM.zip › Fig 2/Fig 2A/lrx1 rol23, EGR1-GFP.tif]

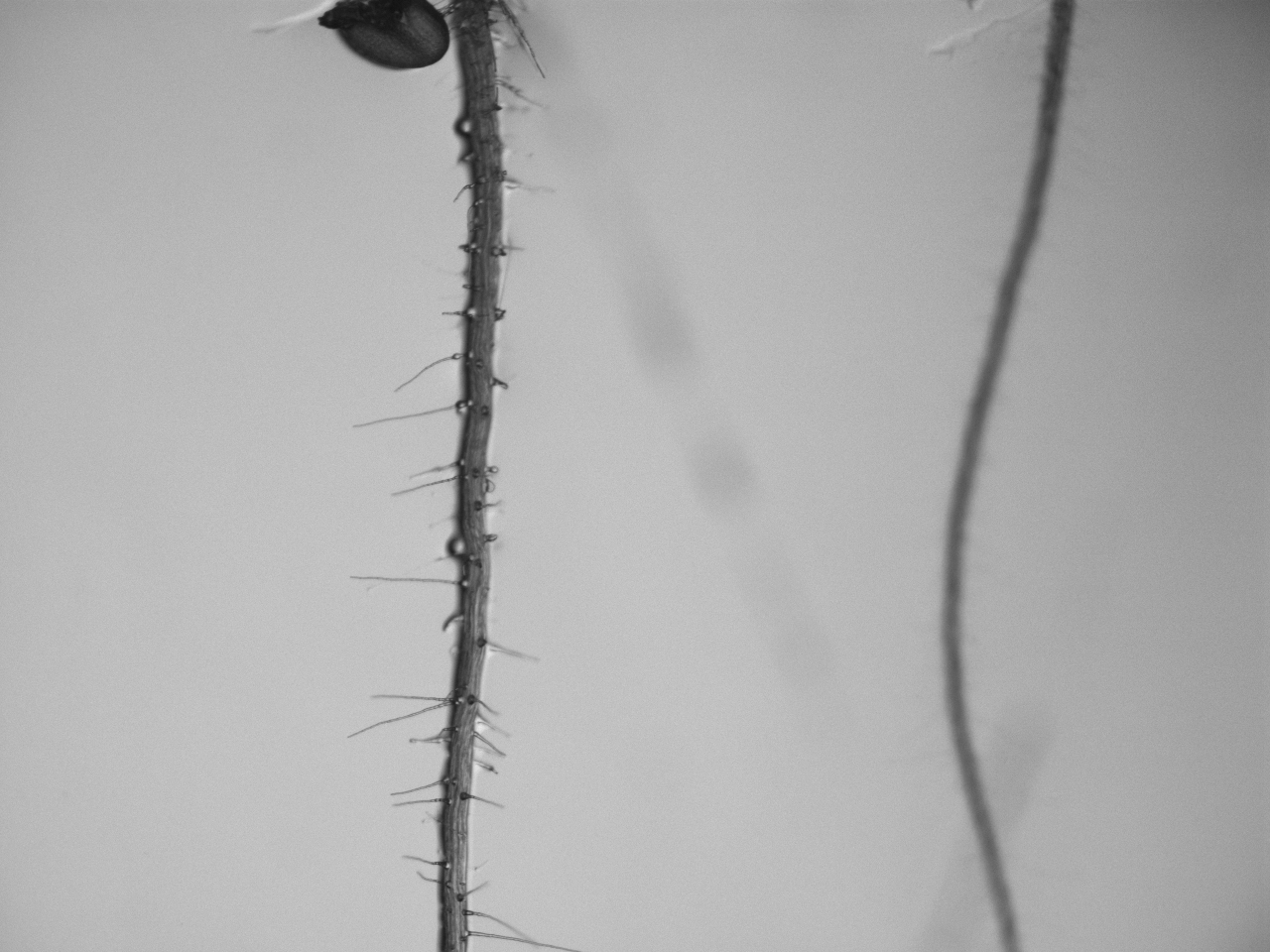

Supplement: Supplementary file 4 — Source data Fig. 2 [file 44318_2025_614_MOESM4_ESM.zip › Fig 2/Fig 2A/lrx1 rol23, PP2C12-GFP_T2_1.tif]

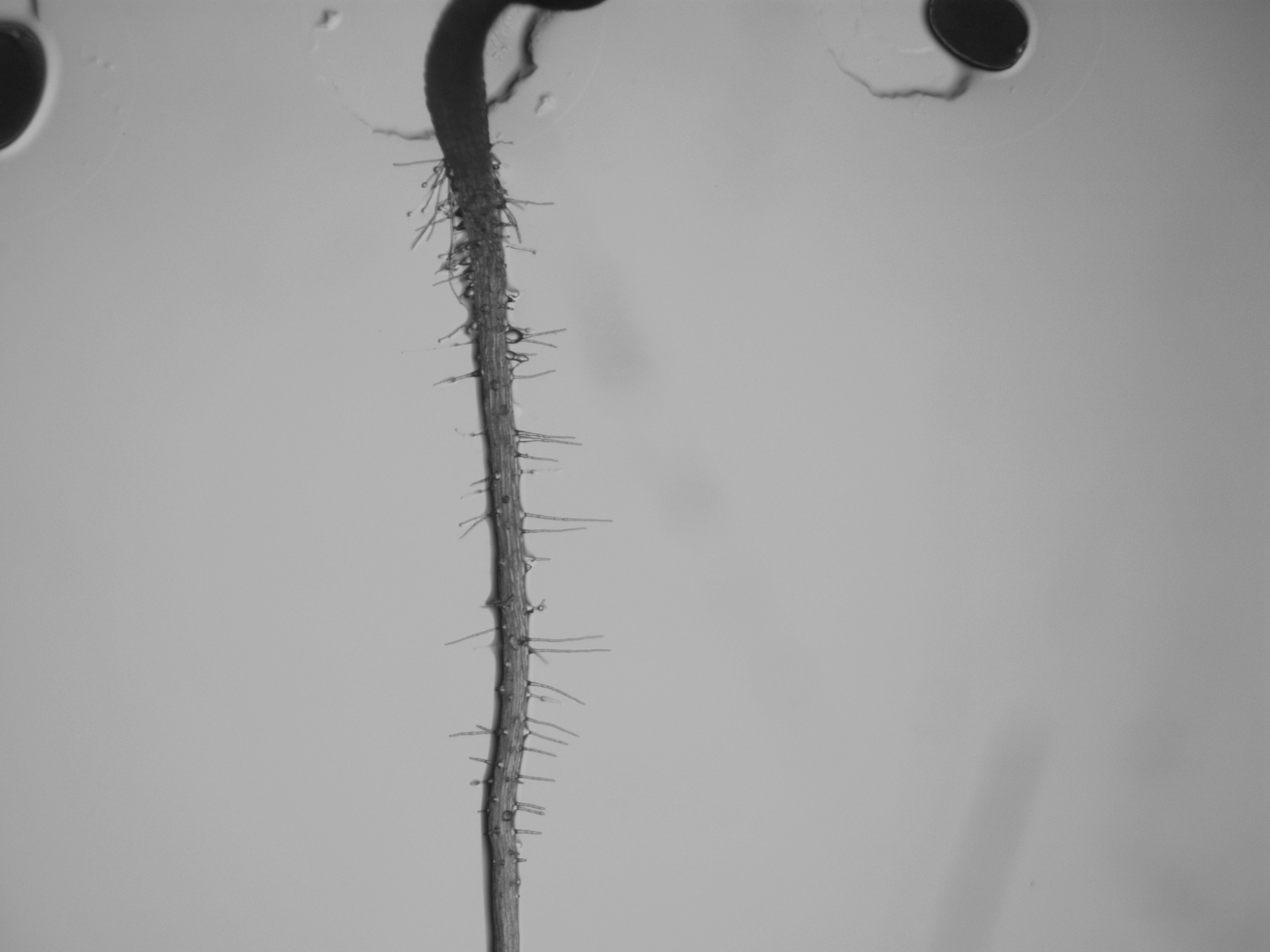

Supplement: Supplementary file 4 — Source data Fig. 2 [file 44318_2025_614_MOESM4_ESM.zip › Fig 2/Fig 2A/lrx1 rol23, PP2C12-GFP_T2_2.tif]

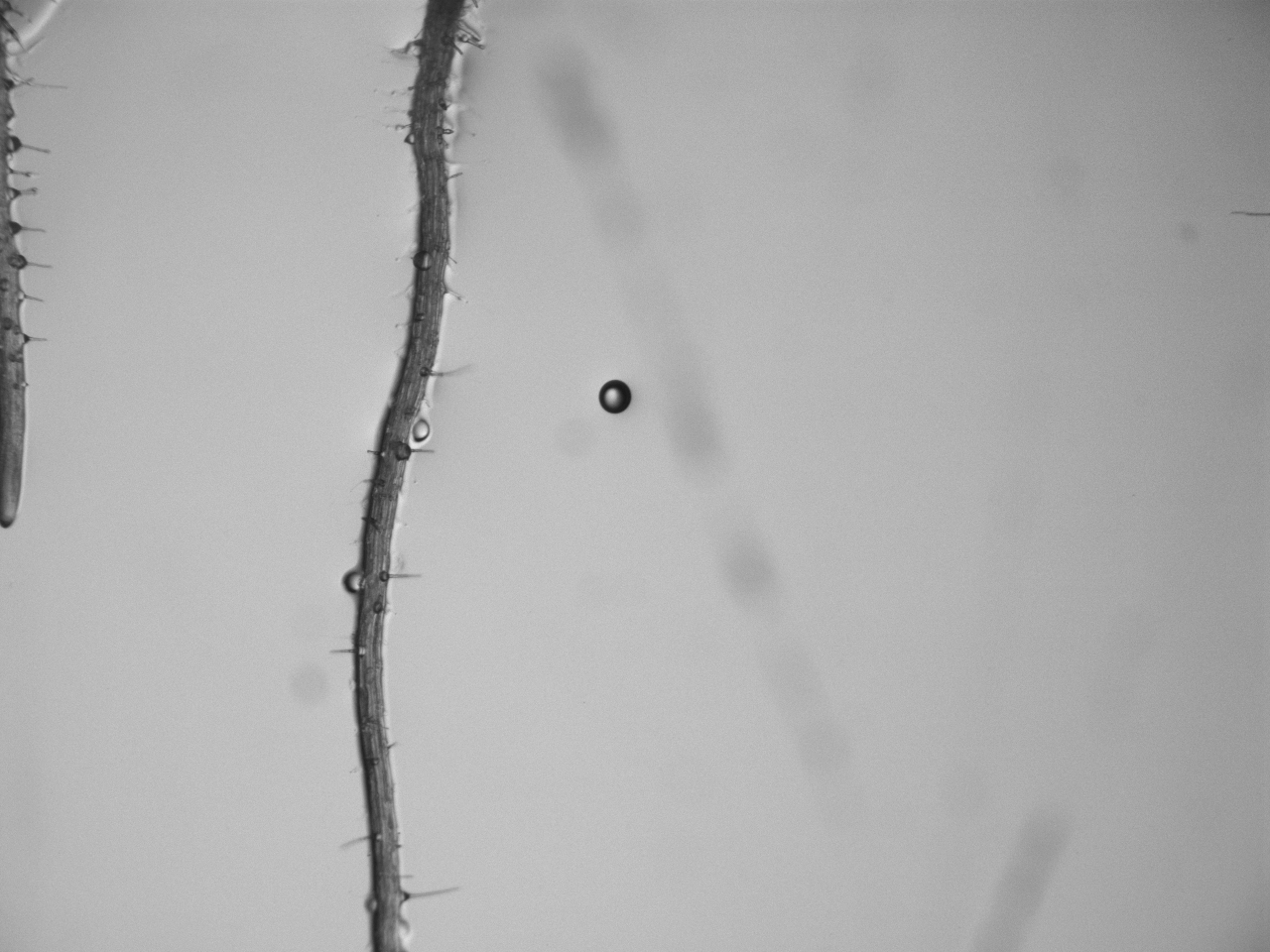

Supplement: Supplementary file 4 — Source data Fig. 2 [file 44318_2025_614_MOESM4_ESM.zip › Fig 2/Fig 2A/lrx1 rol23, PP2C12-GFP_T2_3.tif]

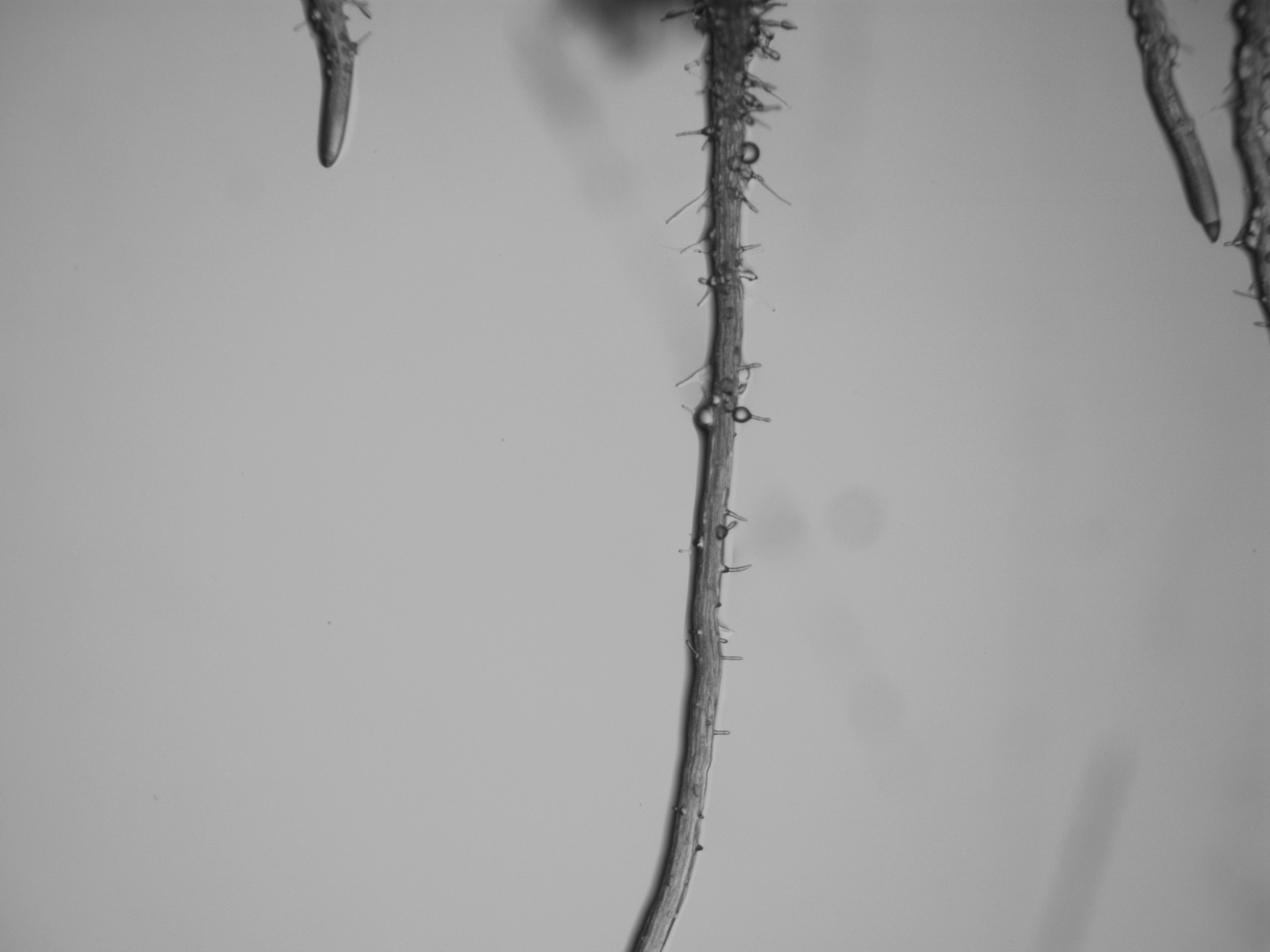

Supplement: Supplementary file 4 — Source data Fig. 2 [file 44318_2025_614_MOESM4_ESM.zip › Fig 2/Fig 2A/lrx1 rol23, PP2C15-GFP_T2_1.tif]

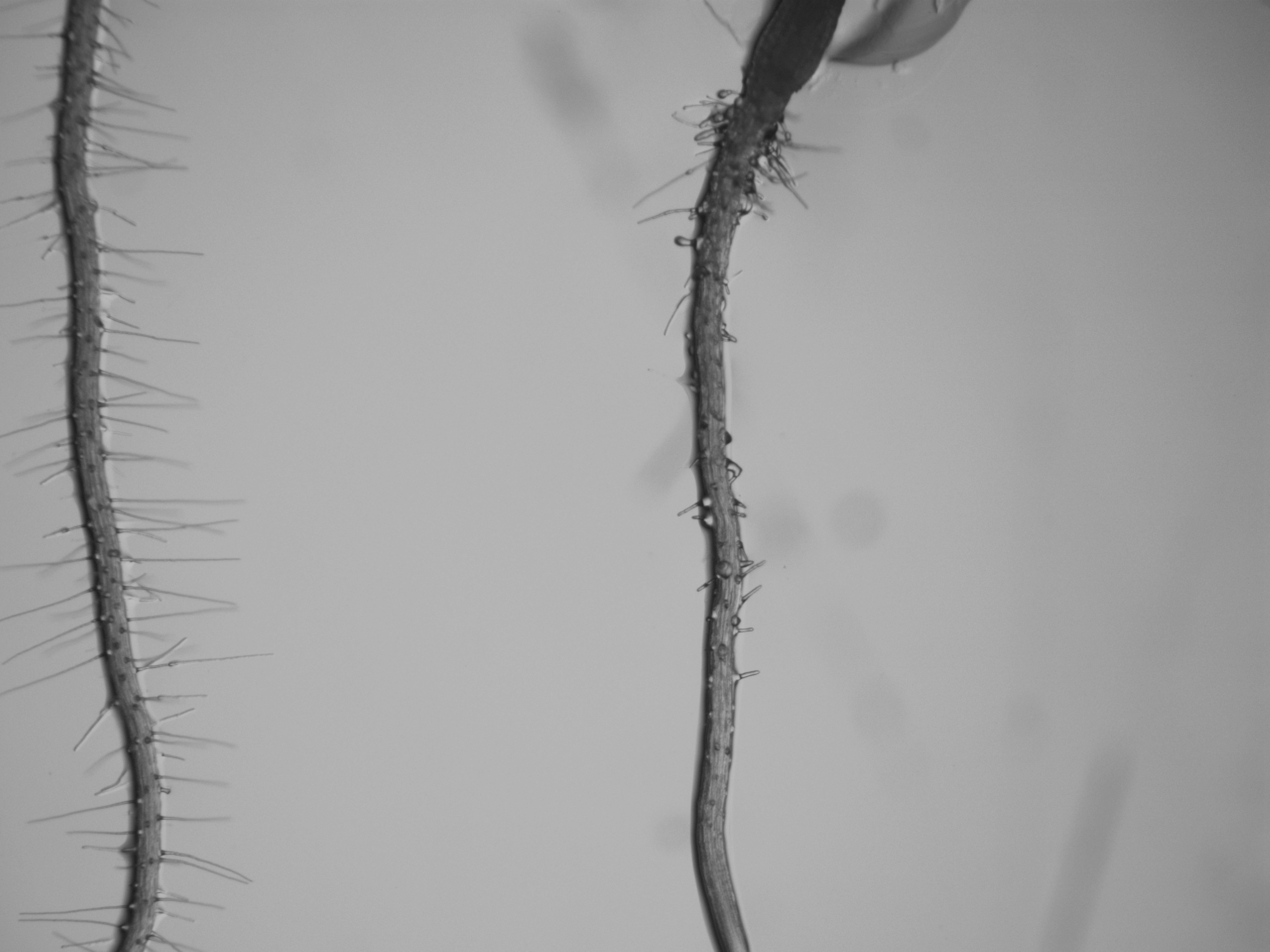

Supplement: Supplementary file 4 — Source data Fig. 2 [file 44318_2025_614_MOESM4_ESM.zip › Fig 2/Fig 2A/lrx1 rol23, PP2C15-GFP_T2_2.tif]

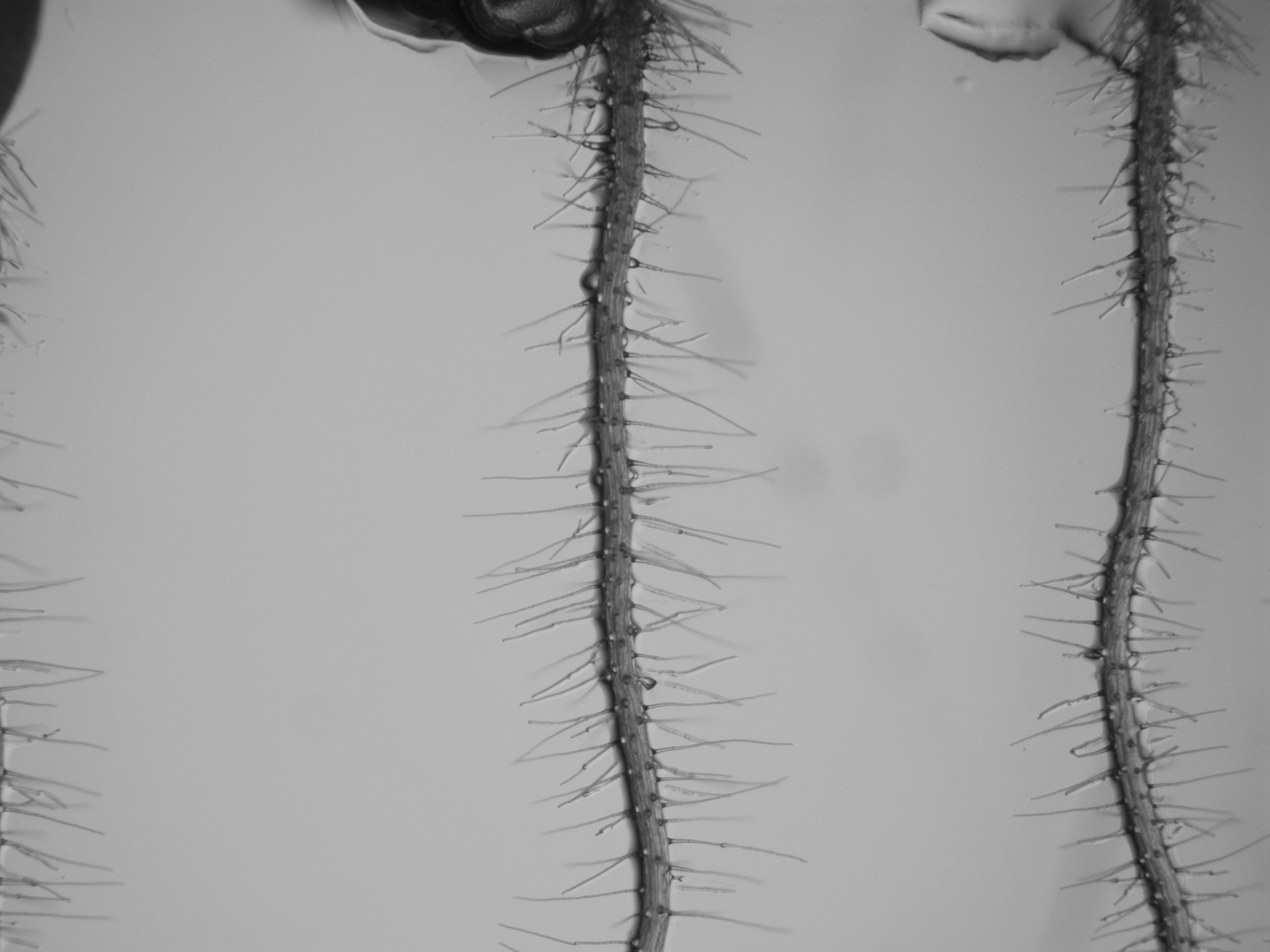

Supplement: Supplementary file 4 — Source data Fig. 2 [file 44318_2025_614_MOESM4_ESM.zip › Fig 2/Fig 2A/lrx1 rol23, PP2C35-GFP_T2_1.tif]

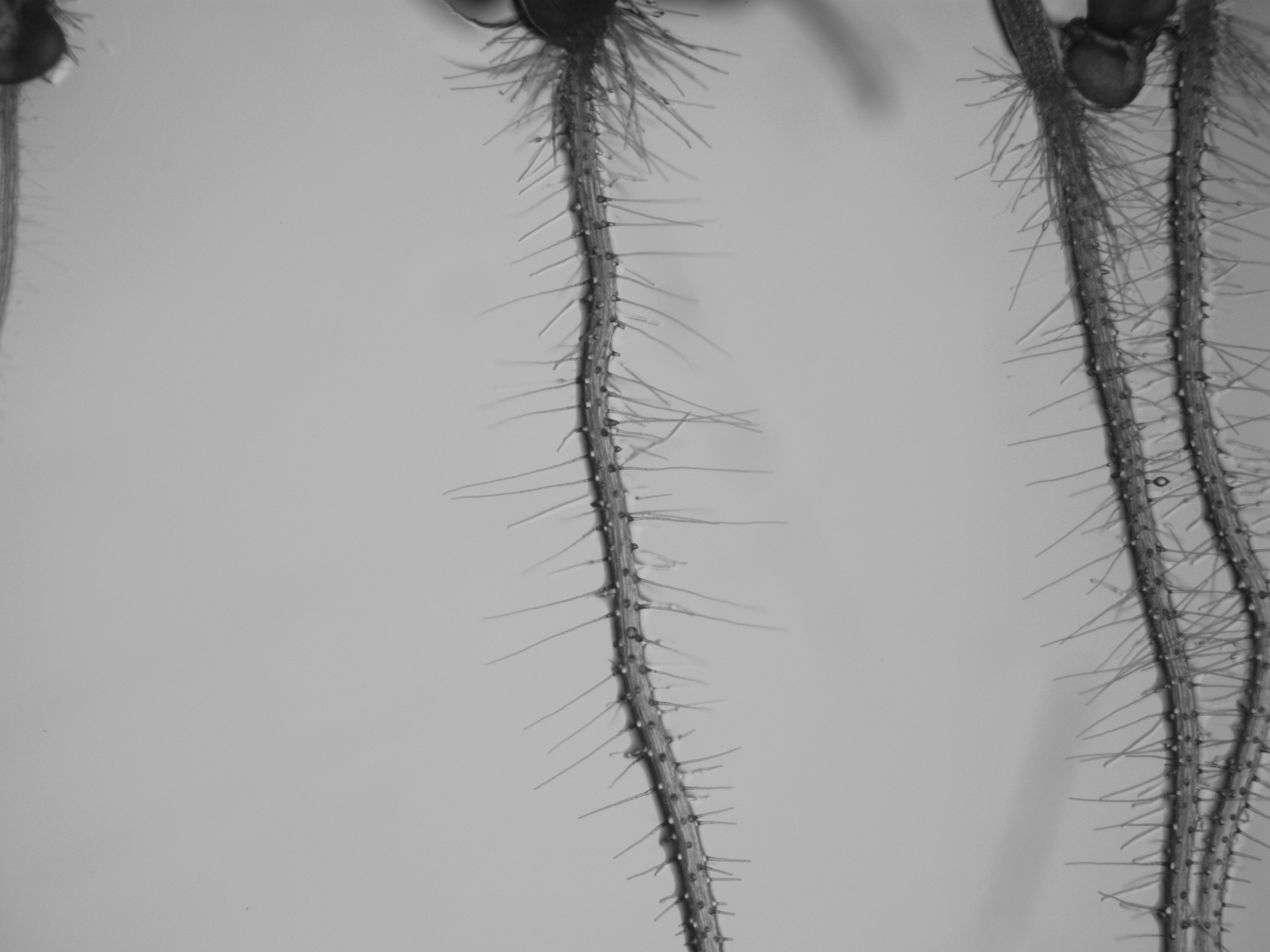

Supplement: Supplementary file 4 — Source data Fig. 2 [file 44318_2025_614_MOESM4_ESM.zip › Fig 2/Fig 2A/lrx1 rol23, PP2C38-GFP_T2_1.tif]

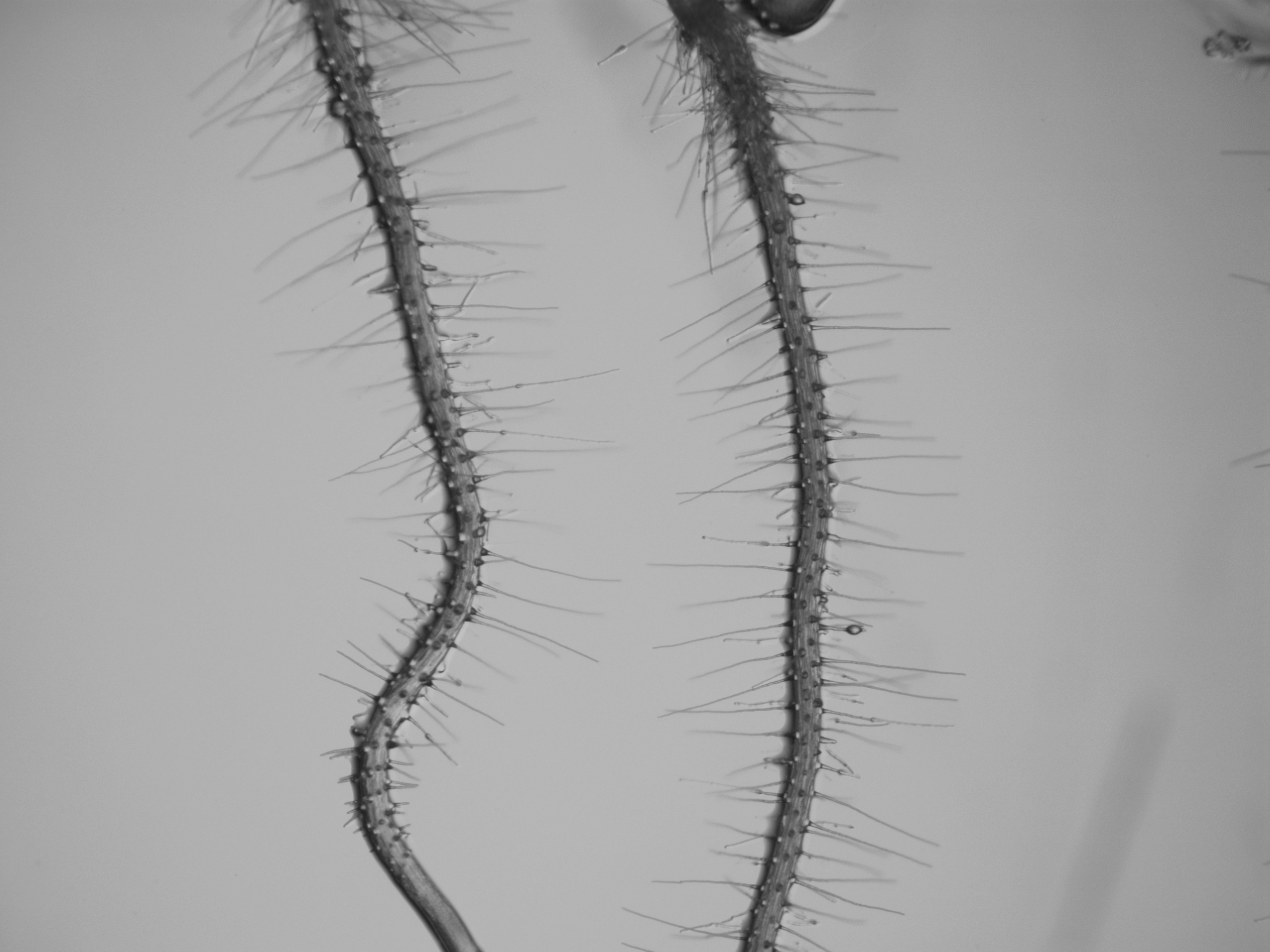

Supplement: Supplementary file 4 — Source data Fig. 2 [file 44318_2025_614_MOESM4_ESM.zip › Fig 2/Fig 2A/lrx1 rol23, PP2C38-GFP_T2_2.tif]

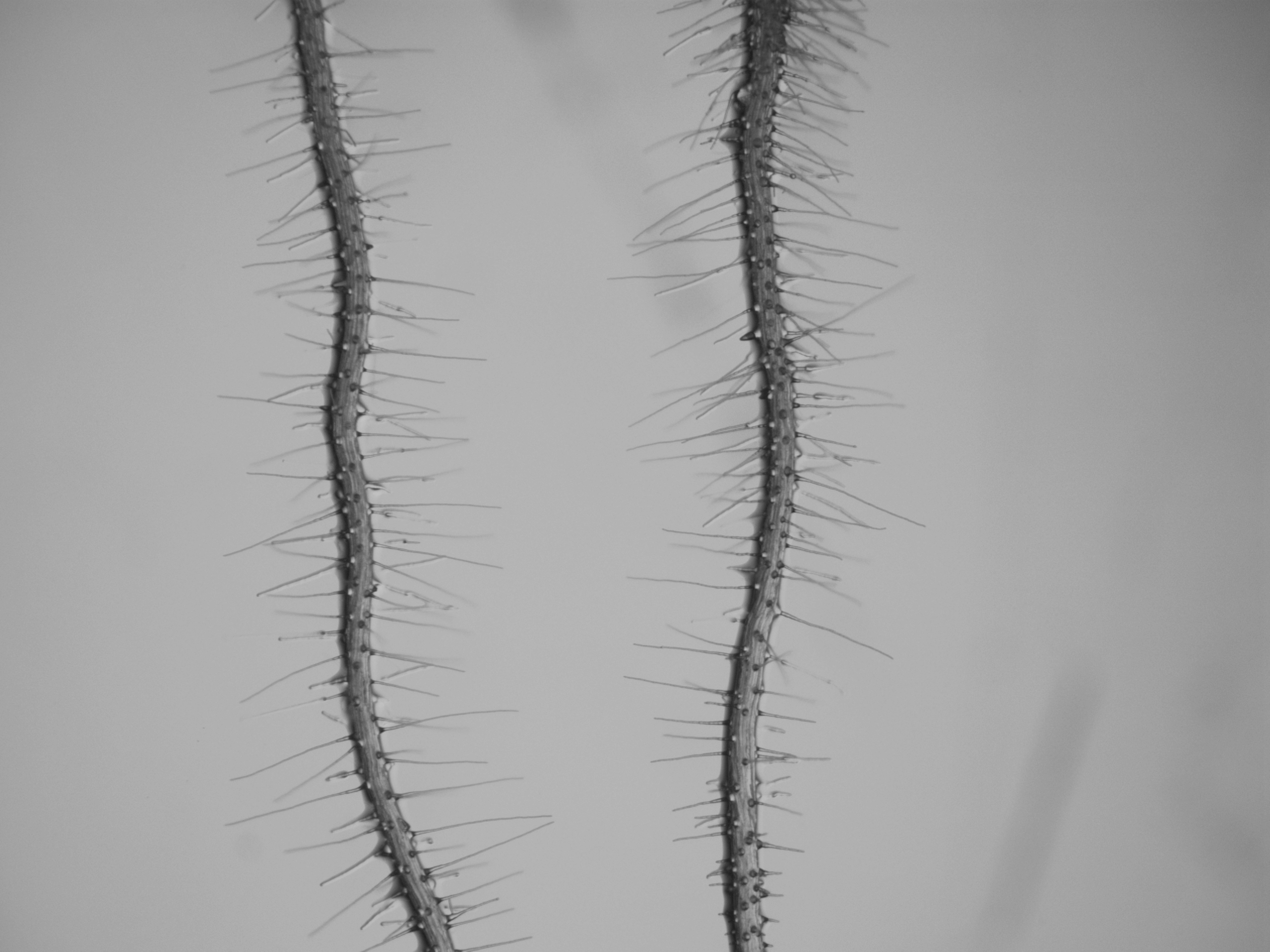

Supplement: Supplementary file 4 — Source data Fig. 2 [file 44318_2025_614_MOESM4_ESM.zip › Fig 2/Fig 2A/lrx1 rol23, PP2C52-GFP_T2_1.tif]

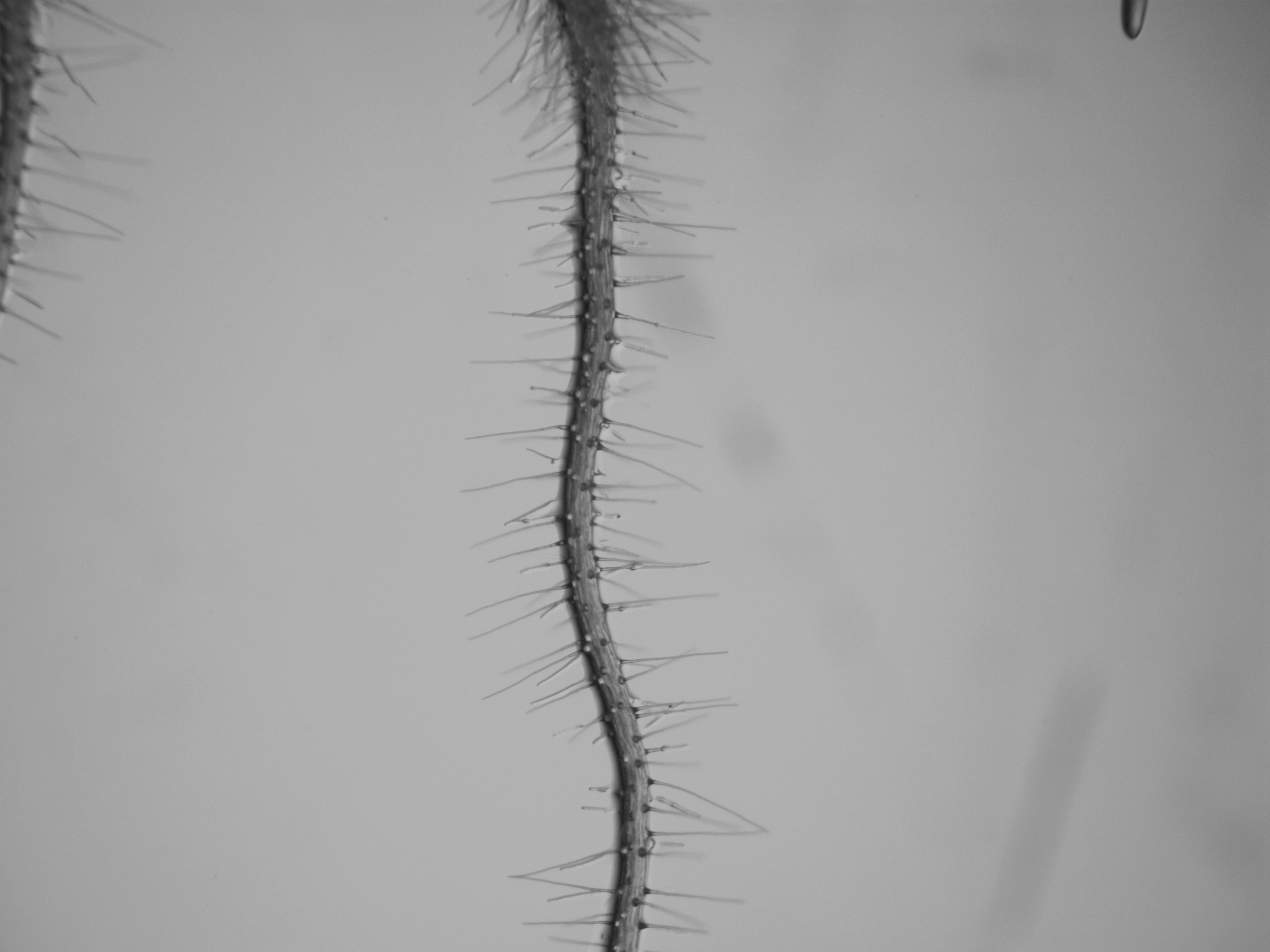

Supplement: Supplementary file 4 — Source data Fig. 2 [file 44318_2025_614_MOESM4_ESM.zip › Fig 2/Fig 2A/lrx1 rol23, PP2C52-GFP_T2_2.tif]

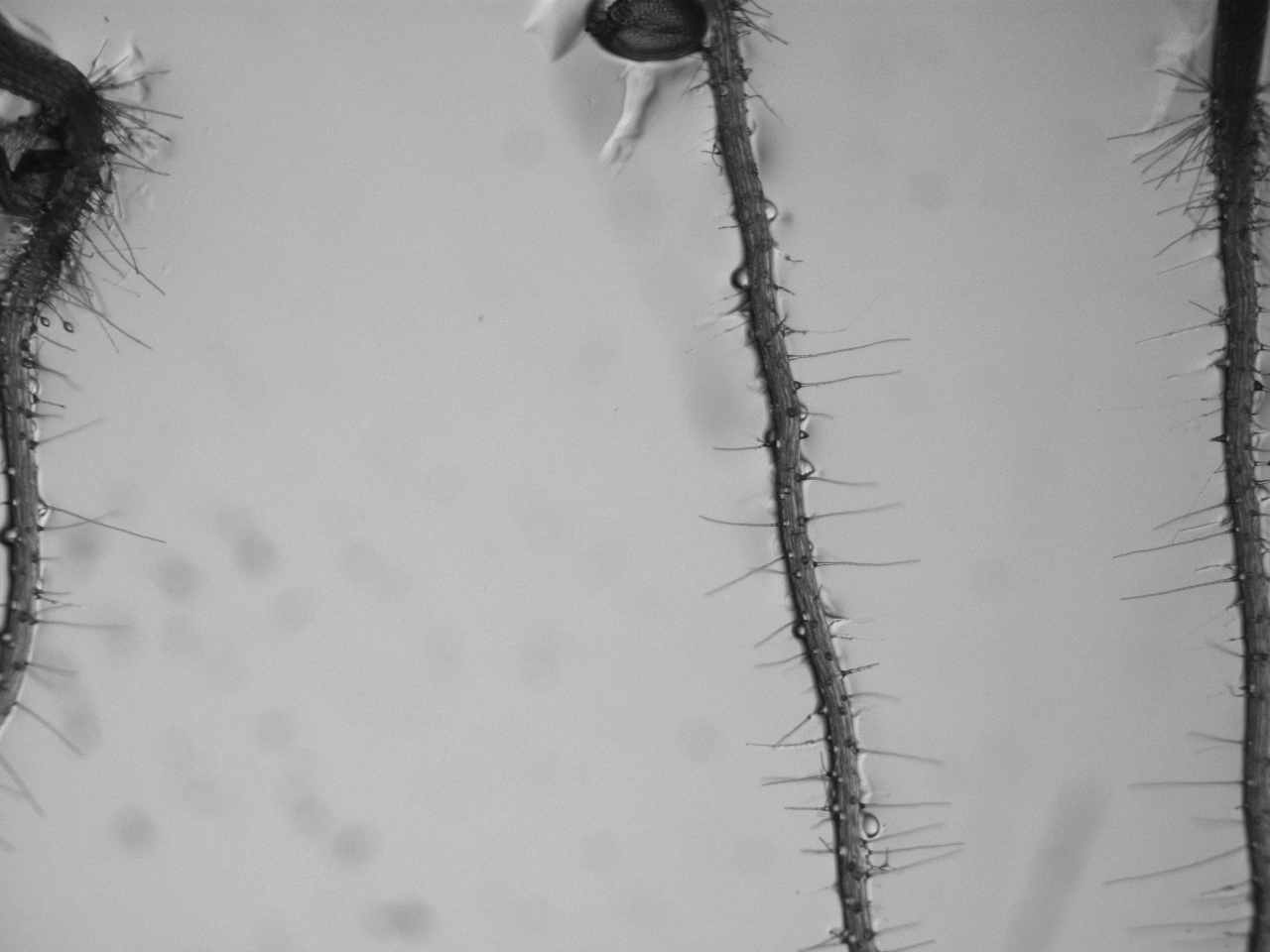

Supplement: Supplementary file 4 — Source data Fig. 2 [file 44318_2025_614_MOESM4_ESM.zip › Fig 2/Fig 2A/lrx1 rol23, PP2CH3-GFP_T2_1.tif]

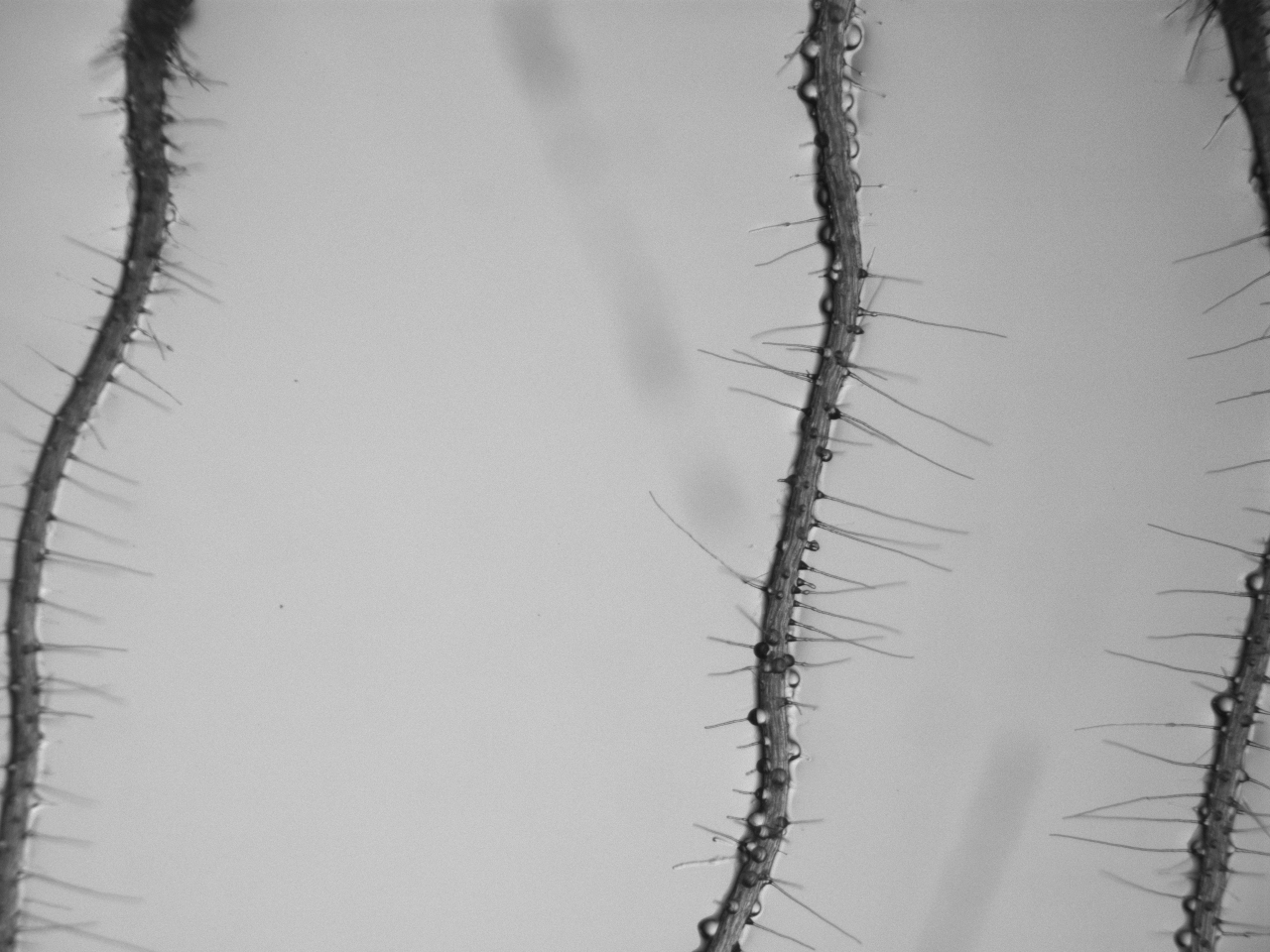

Supplement: Supplementary file 4 — Source data Fig. 2 [file 44318_2025_614_MOESM4_ESM.zip › Fig 2/Fig 2A/lrx1 rol23, PP2CH3-GFP_T2_2.tif]

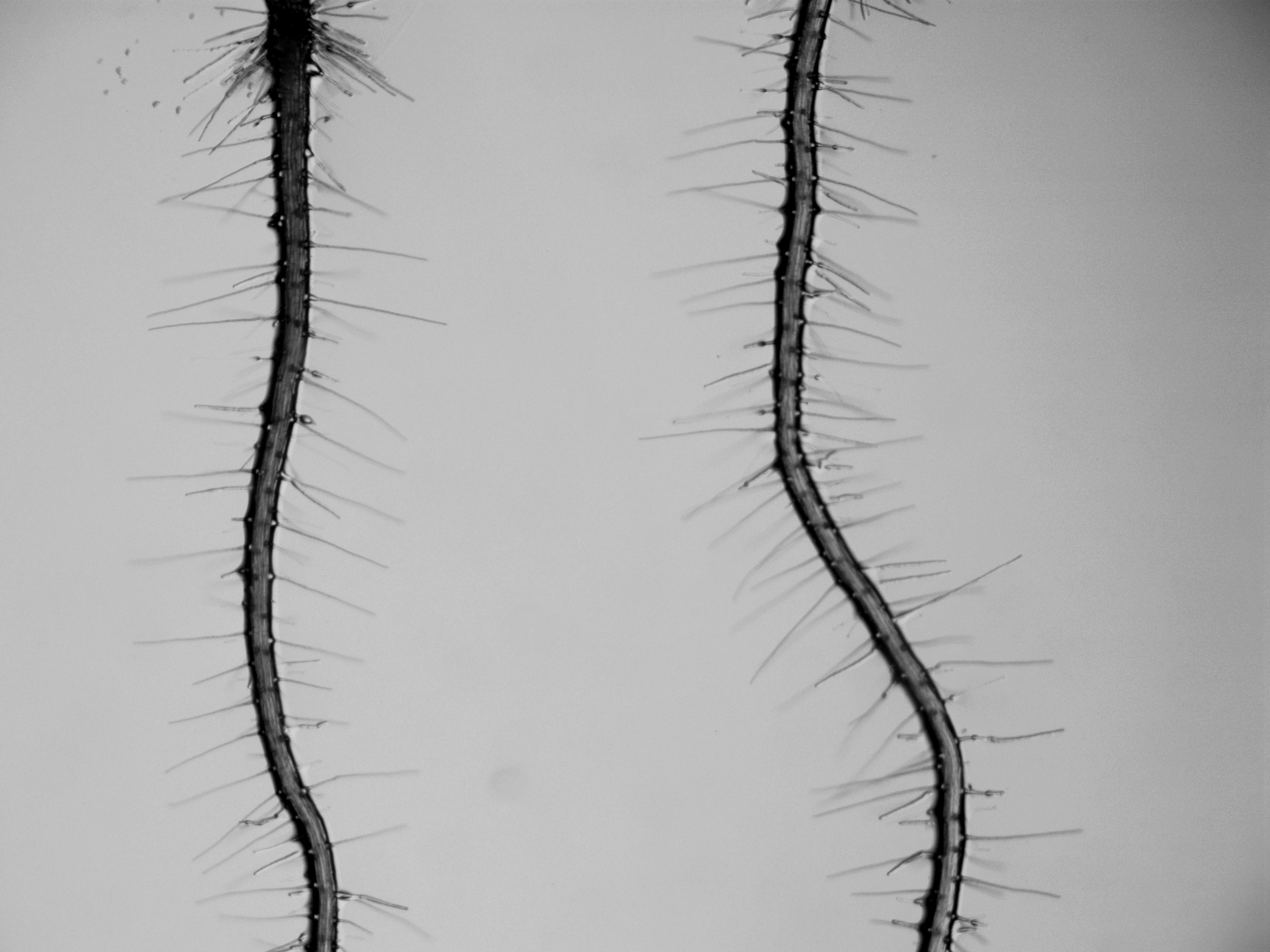

Supplement: Supplementary file 4 — Source data Fig. 2 [file 44318_2025_614_MOESM4_ESM.zip › Fig 2/Fig 2A/lrx1 rol23.tif]

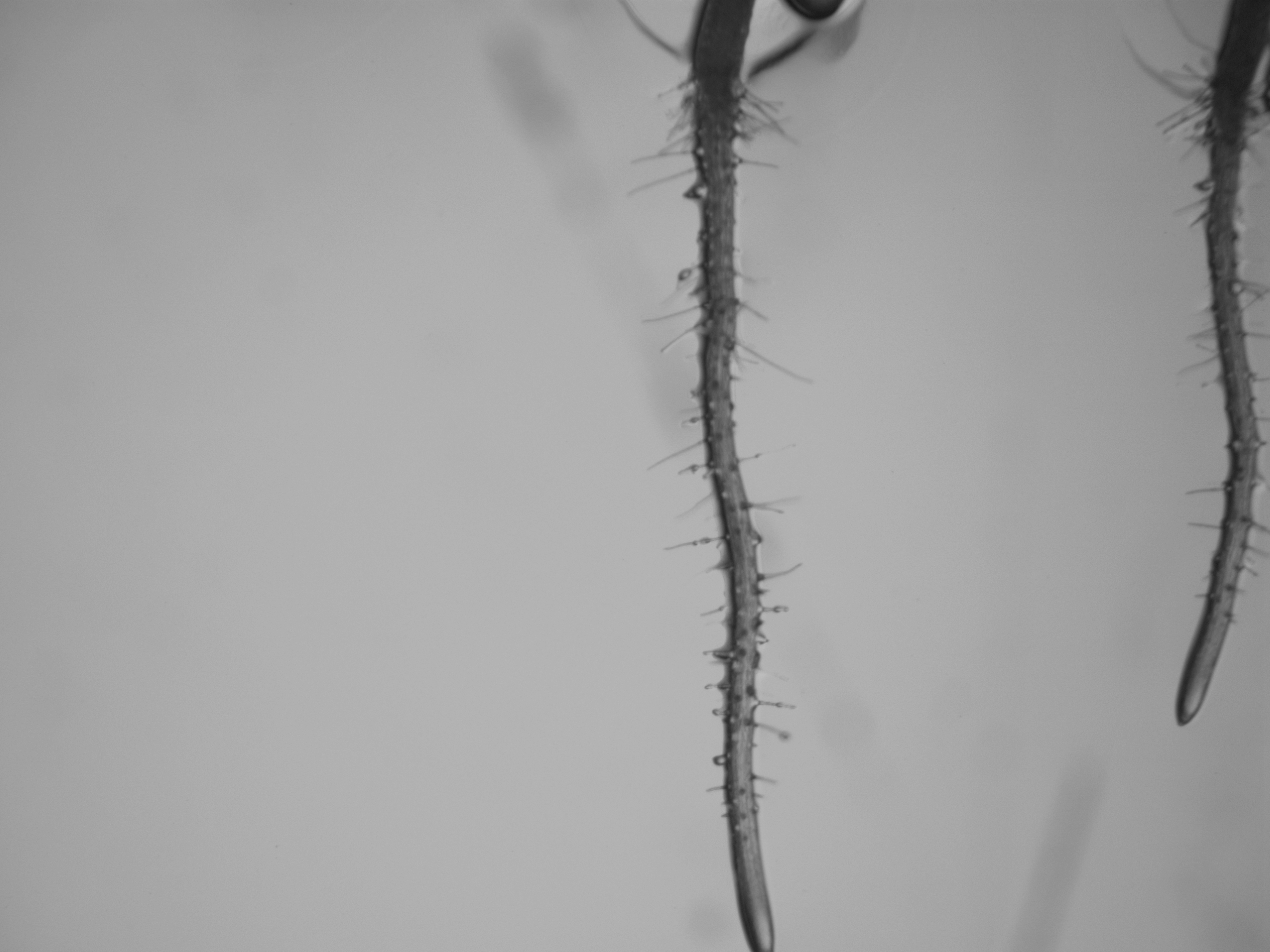

Supplement: Supplementary file 4 — Source data Fig. 2 [file 44318_2025_614_MOESM4_ESM.zip › Fig 2/Fig 2A/lrx1.tif]

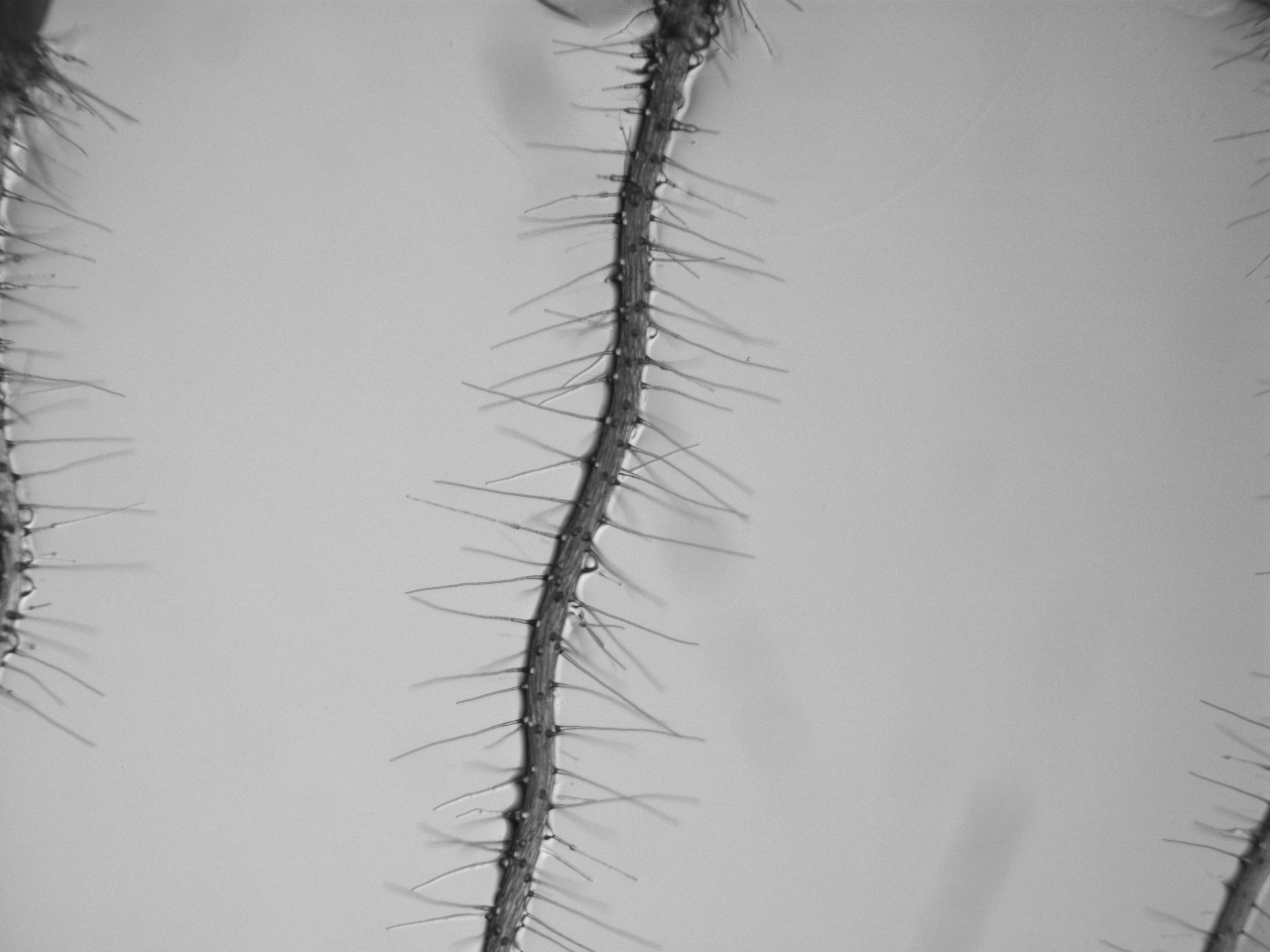

Supplement: Supplementary file 4 — Source data Fig. 2 [file 44318_2025_614_MOESM4_ESM.zip › Fig 2/Fig 2A/PP2C35-GFP_T2_2.tif]

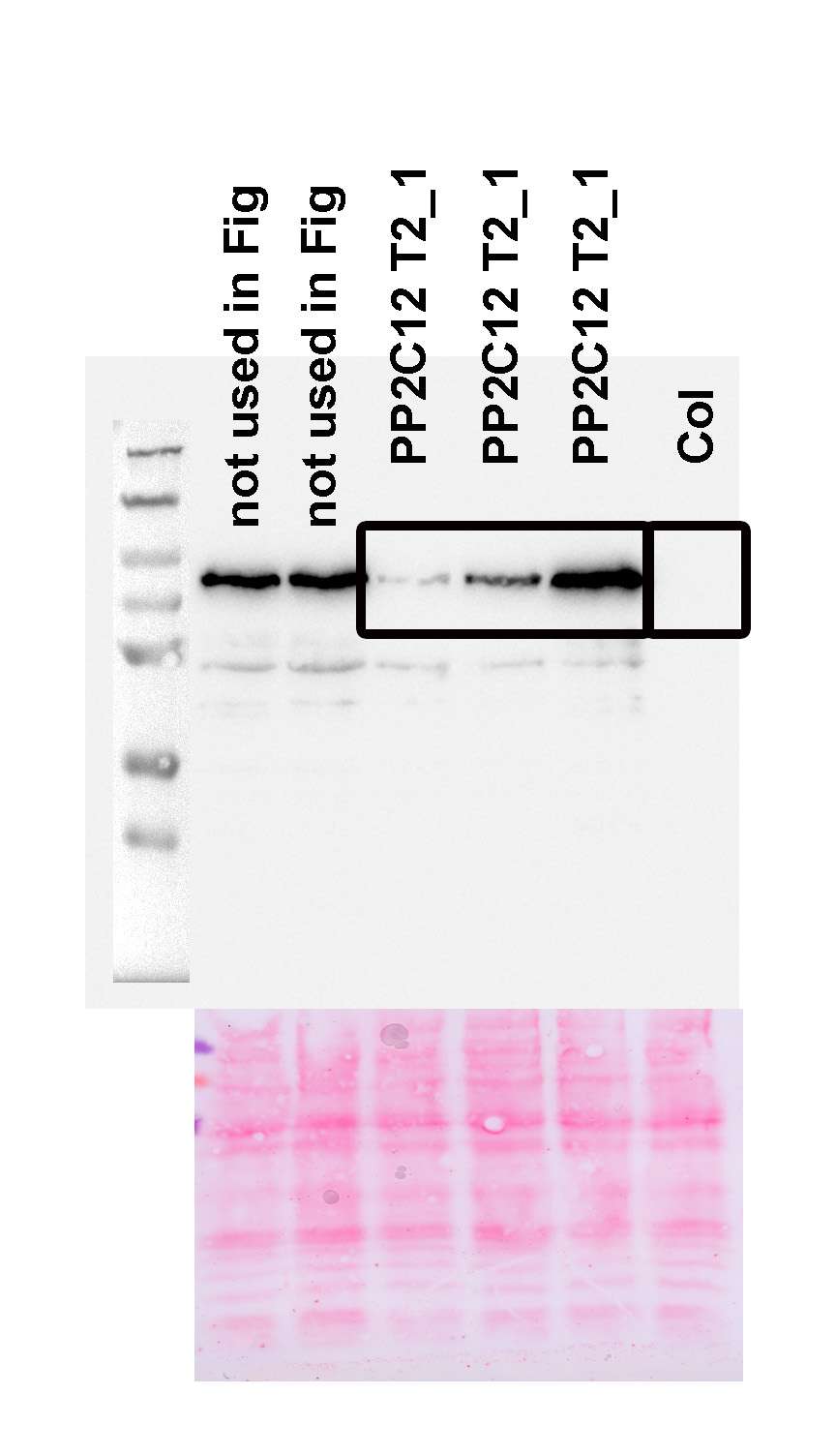

Supplement: Supplementary file 4 — Source data Fig. 2 [file 44318_2025_614_MOESM4_ESM.zip › Fig 2/Fig 2B2.jpg]

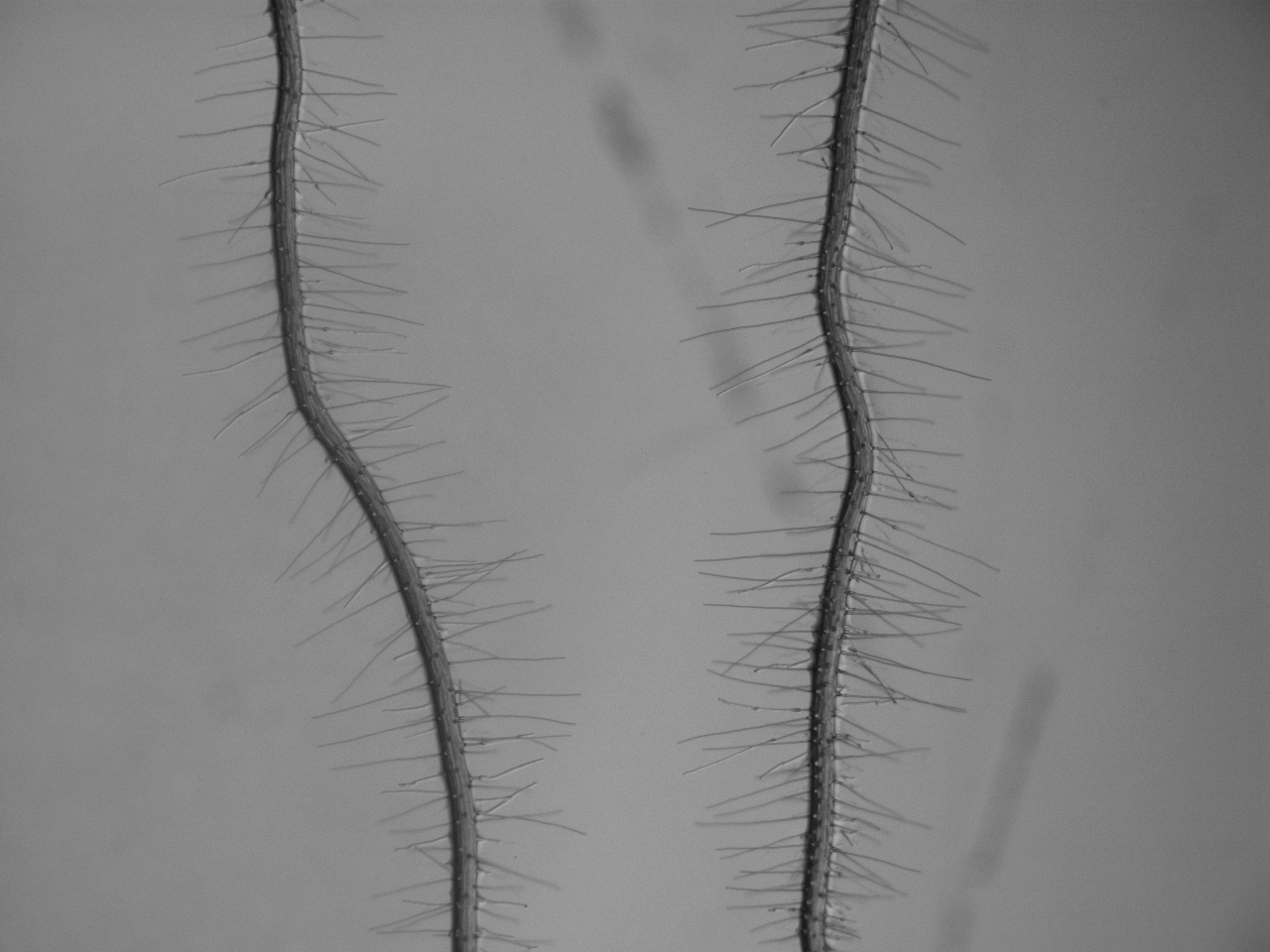

Supplement: Supplementary file 5 — Source data Fig. 3 [file 44318_2025_614_MOESM5_ESM.zip › Fig 3/Fig 3A/Col.tif]

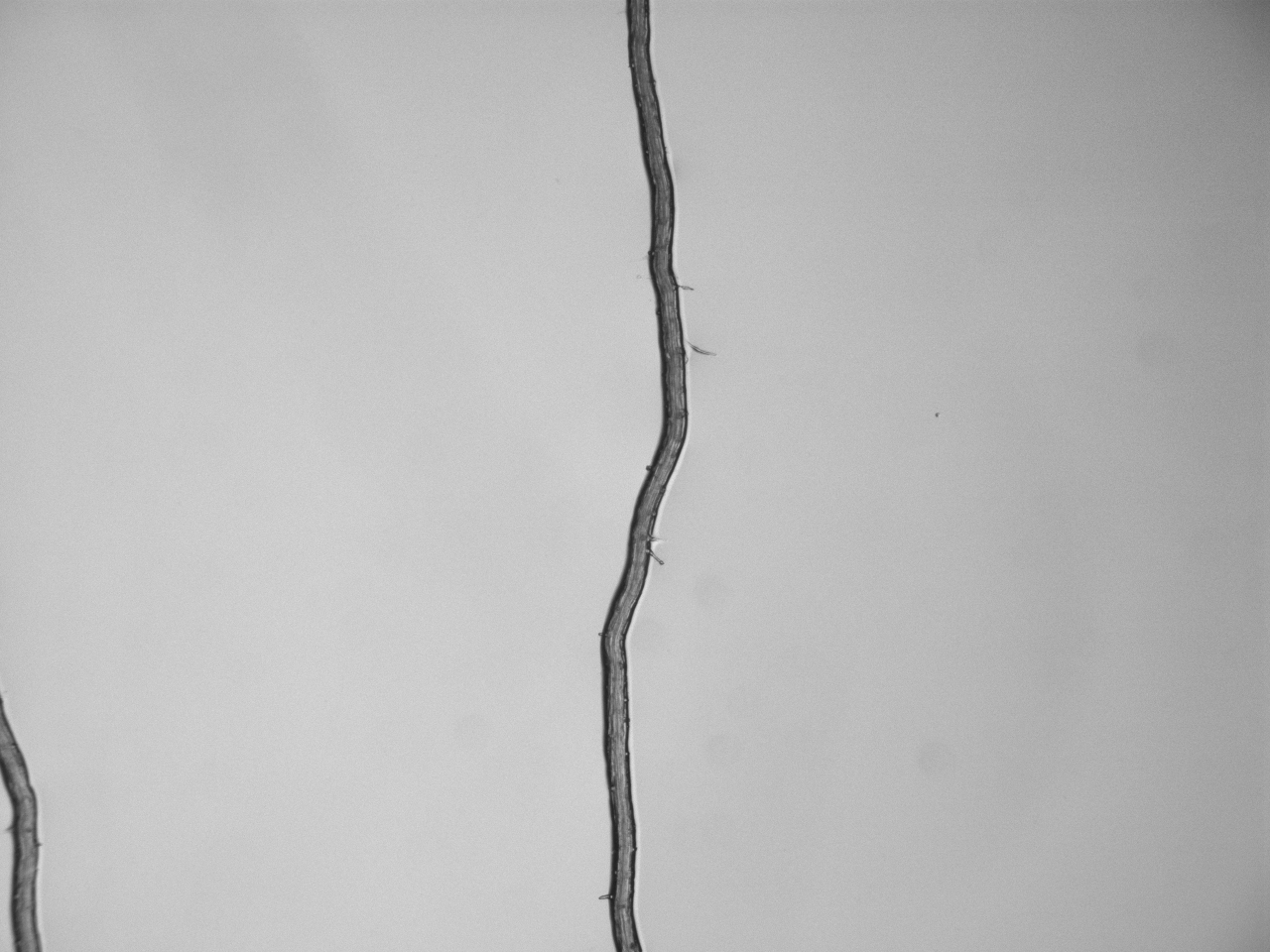

Supplement: Supplementary file 5 — Source data Fig. 3 [file 44318_2025_614_MOESM5_ESM.zip › Fig 3/Fig 3A/fer-4 rol23.tif]

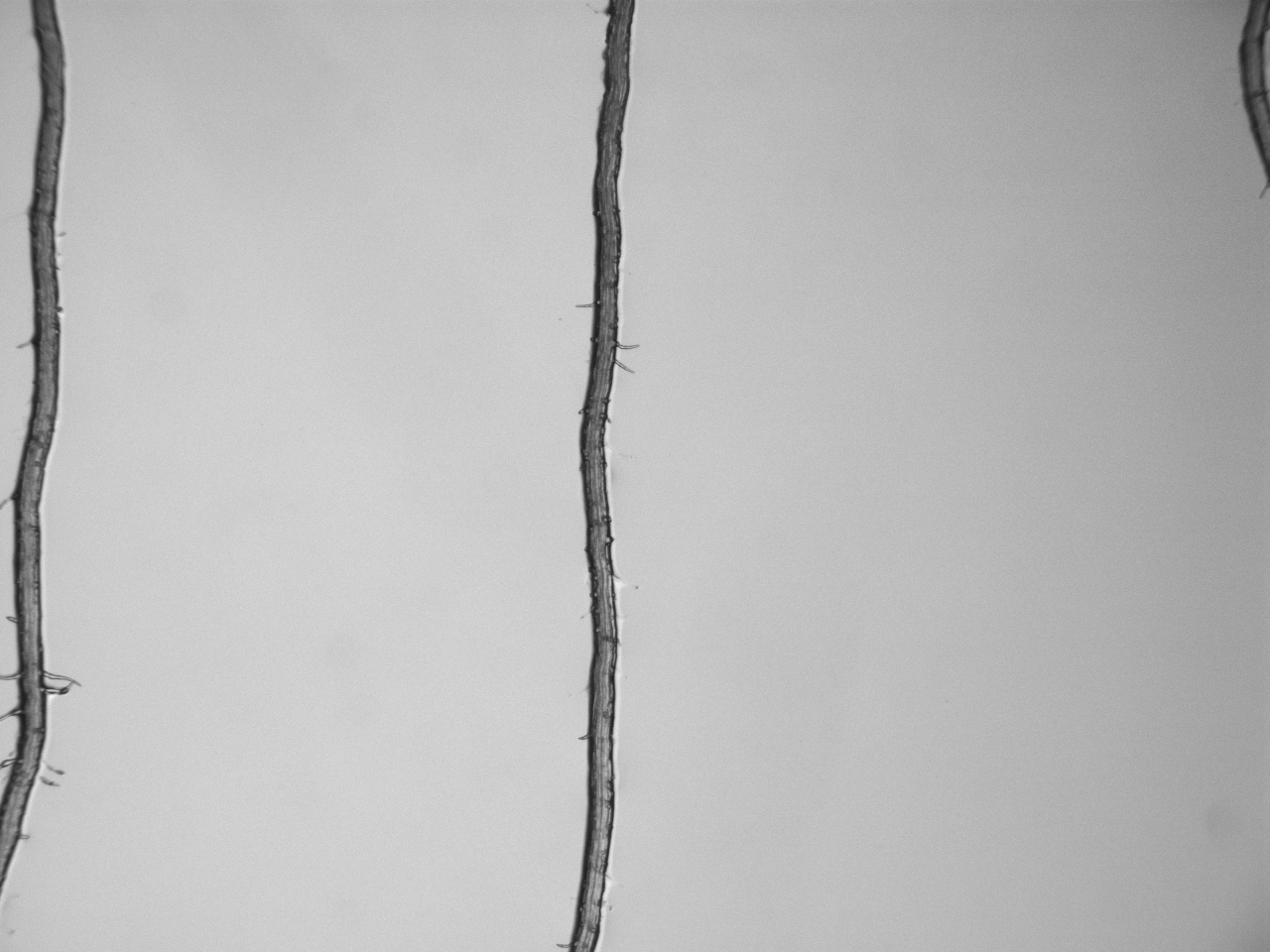

Supplement: Supplementary file 5 — Source data Fig. 3 [file 44318_2025_614_MOESM5_ESM.zip › Fig 3/Fig 3A/fer-4.tif]

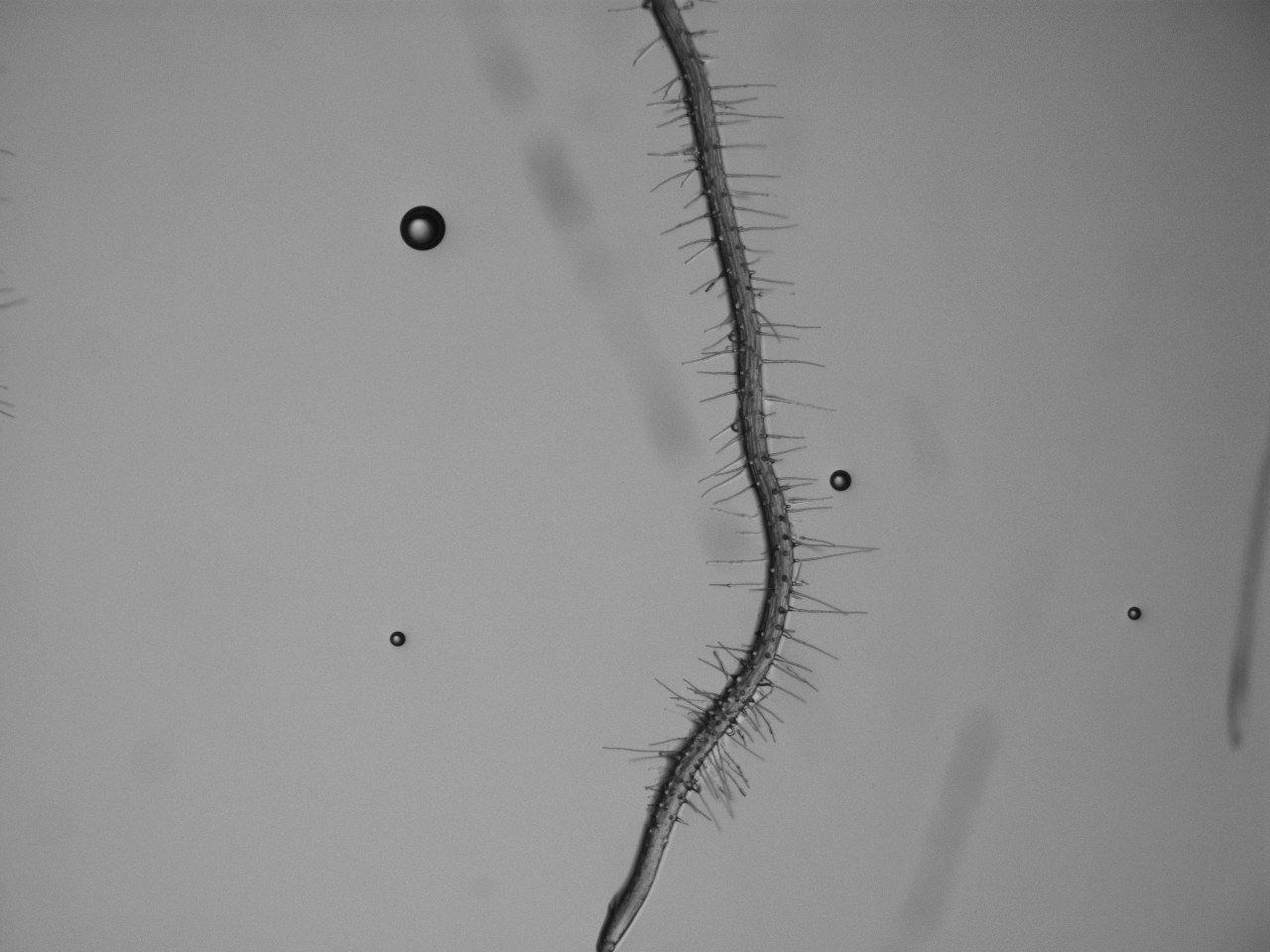

Supplement: Supplementary file 5 — Source data Fig. 3 [file 44318_2025_614_MOESM5_ESM.zip › Fig 3/Fig 3A/fer-5 rol23.tif]

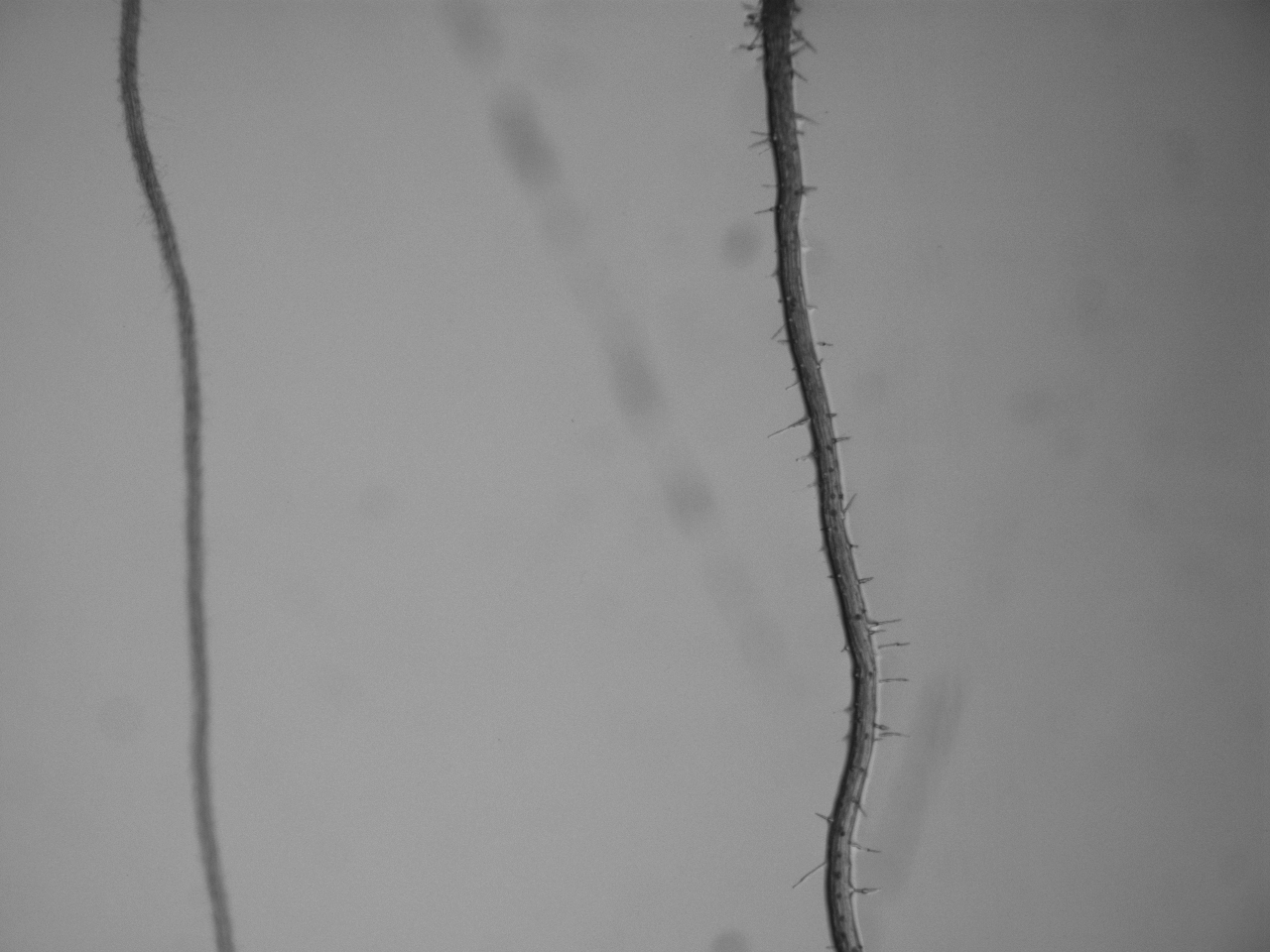

Supplement: Supplementary file 5 — Source data Fig. 3 [file 44318_2025_614_MOESM5_ESM.zip › Fig 3/Fig 3A/fer-5.tif]

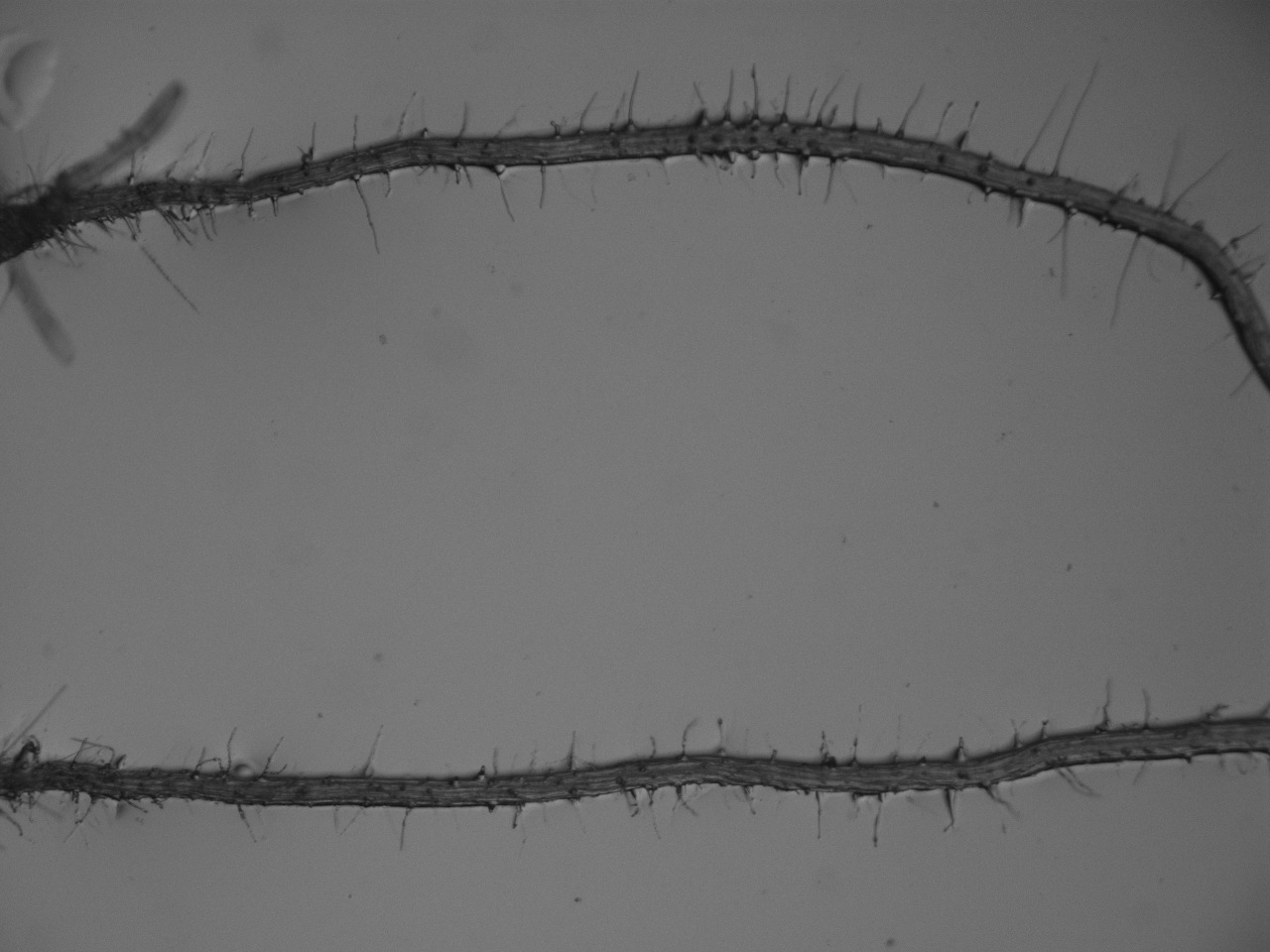

Supplement: Supplementary file 5 — Source data Fig. 3 [file 44318_2025_614_MOESM5_ESM.zip › Fig 3/Fig 3A/lrx1 bak1-5_F3_2_2.tif]

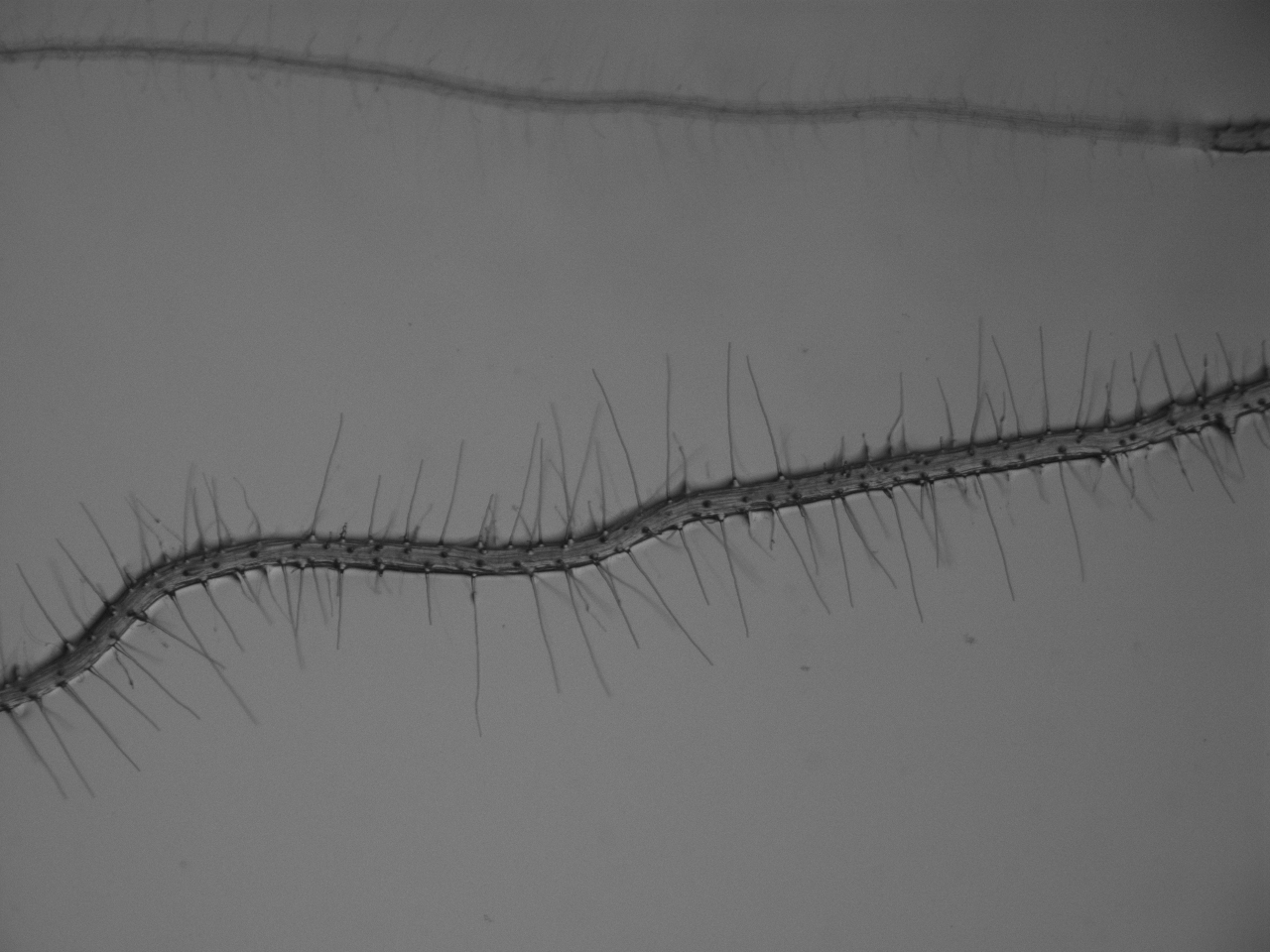

Supplement: Supplementary file 5 — Source data Fig. 3 [file 44318_2025_614_MOESM5_ESM.zip › Fig 3/Fig 3A/lrx1 rol23 bak1-4 F3_14_2.tif]

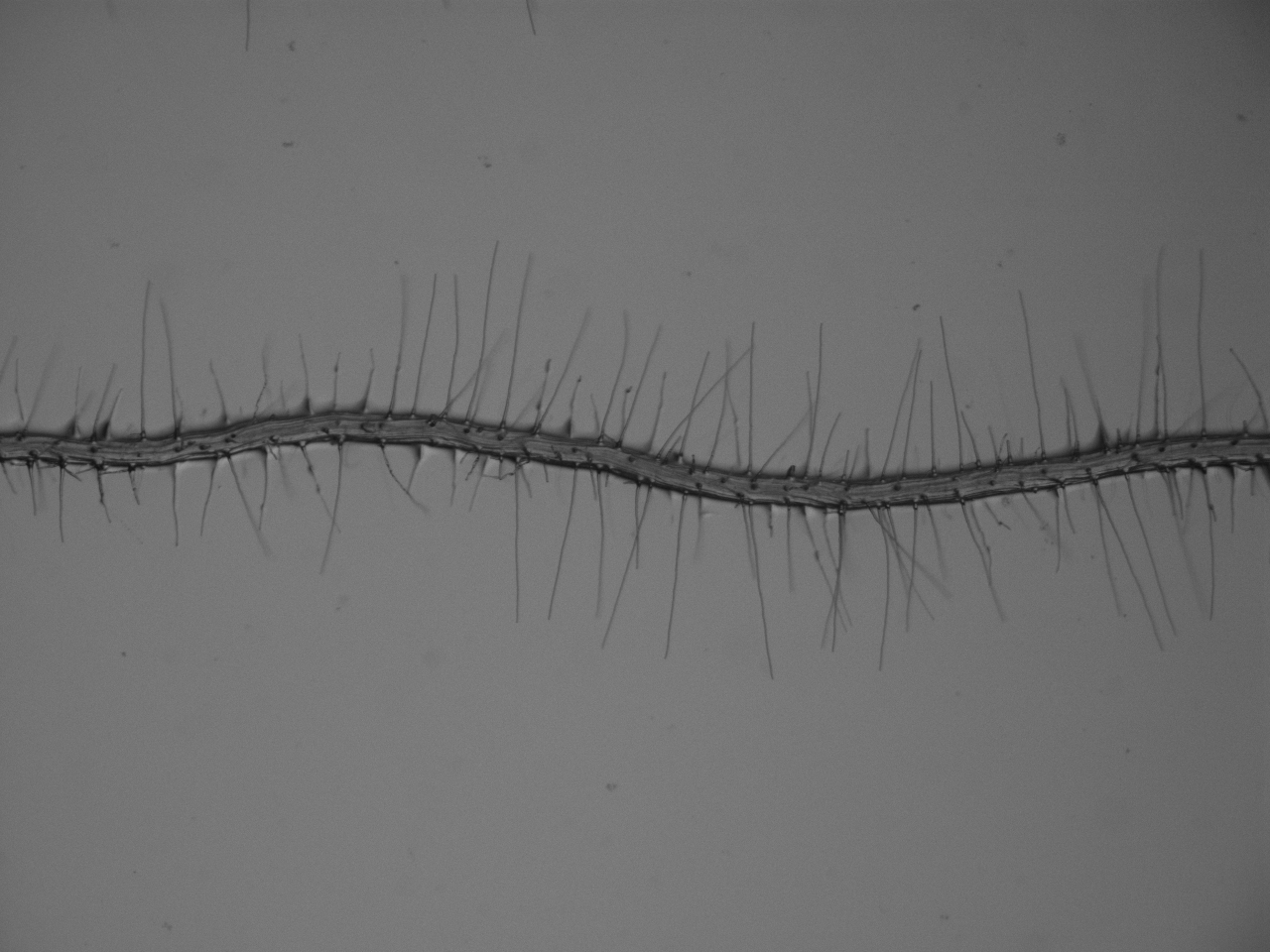

Supplement: Supplementary file 5 — Source data Fig. 3 [file 44318_2025_614_MOESM5_ESM.zip › Fig 3/Fig 3A/lrx1 rol23 bak1-5_F3_10_3.tif]

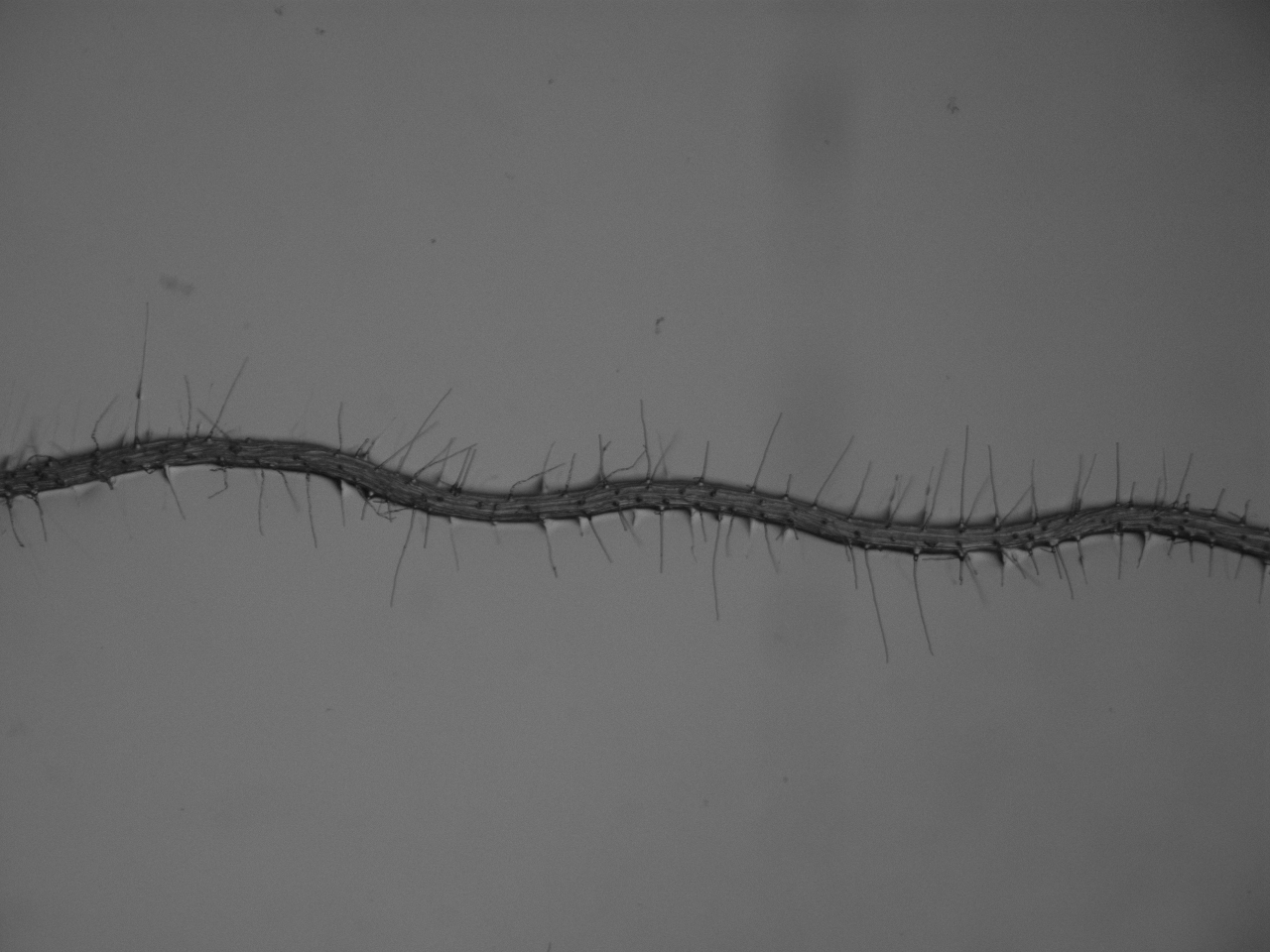

Supplement: Supplementary file 5 — Source data Fig. 3 [file 44318_2025_614_MOESM5_ESM.zip › Fig 3/Fig 3A/lrx1 rol23_5.tif]

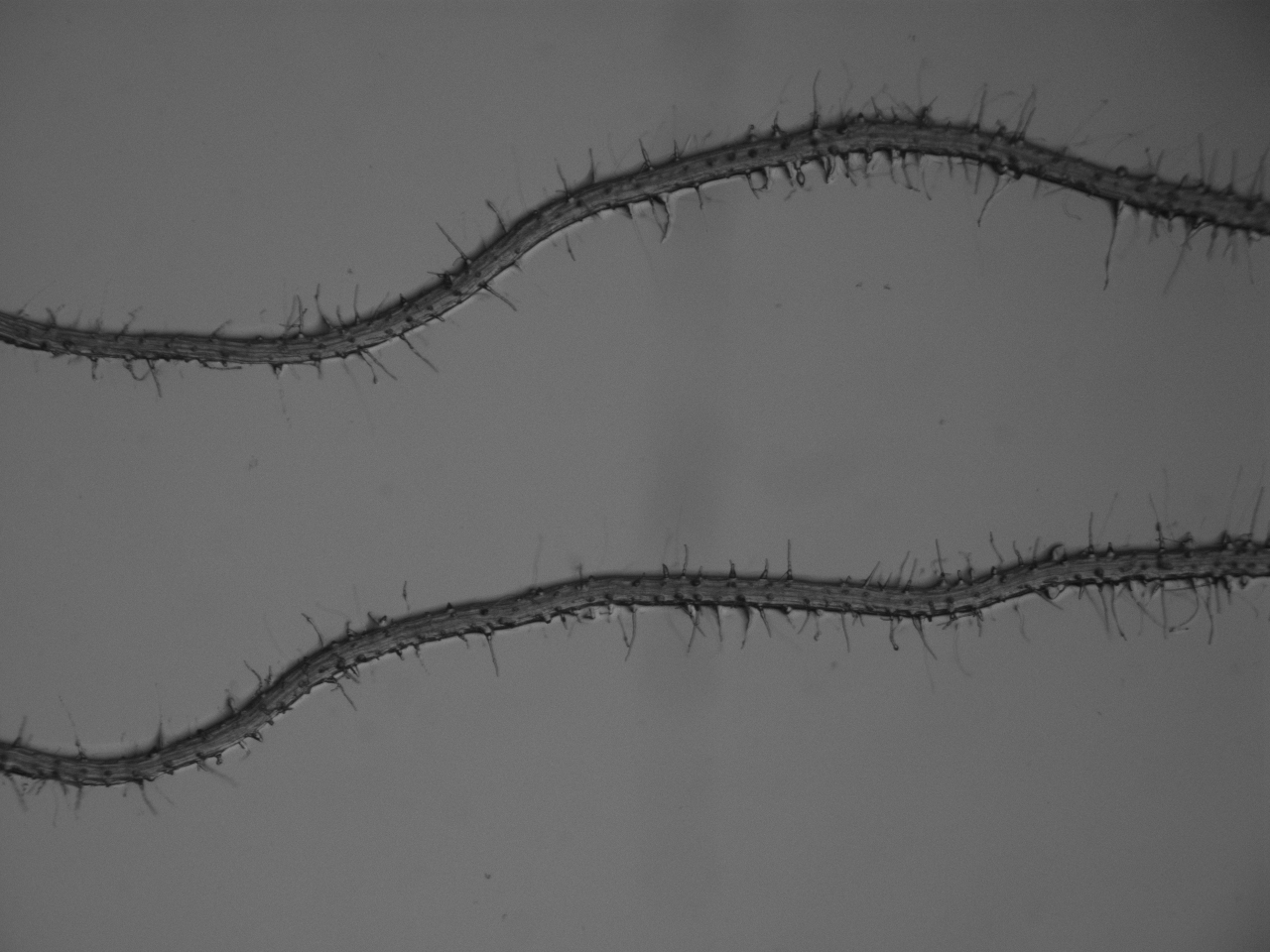

Supplement: Supplementary file 5 — Source data Fig. 3 [file 44318_2025_614_MOESM5_ESM.zip › Fig 3/Fig 3A/lrx1_4.tif]

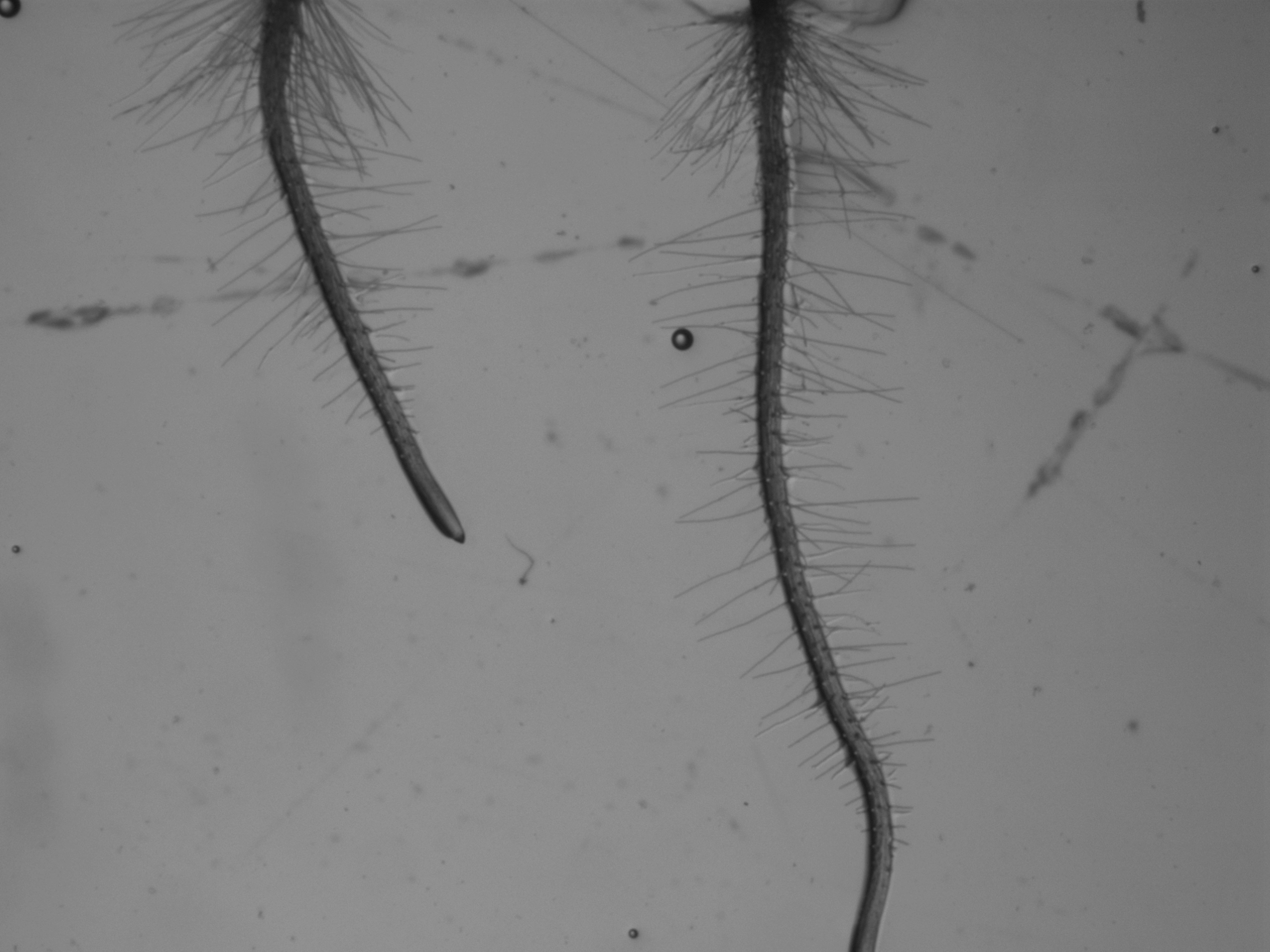

Supplement: Supplementary file 6 — Source data Fig. 4 [file 44318_2025_614_MOESM6_ESM.zip › Fig 4/Fig 4B/Col_2.png]

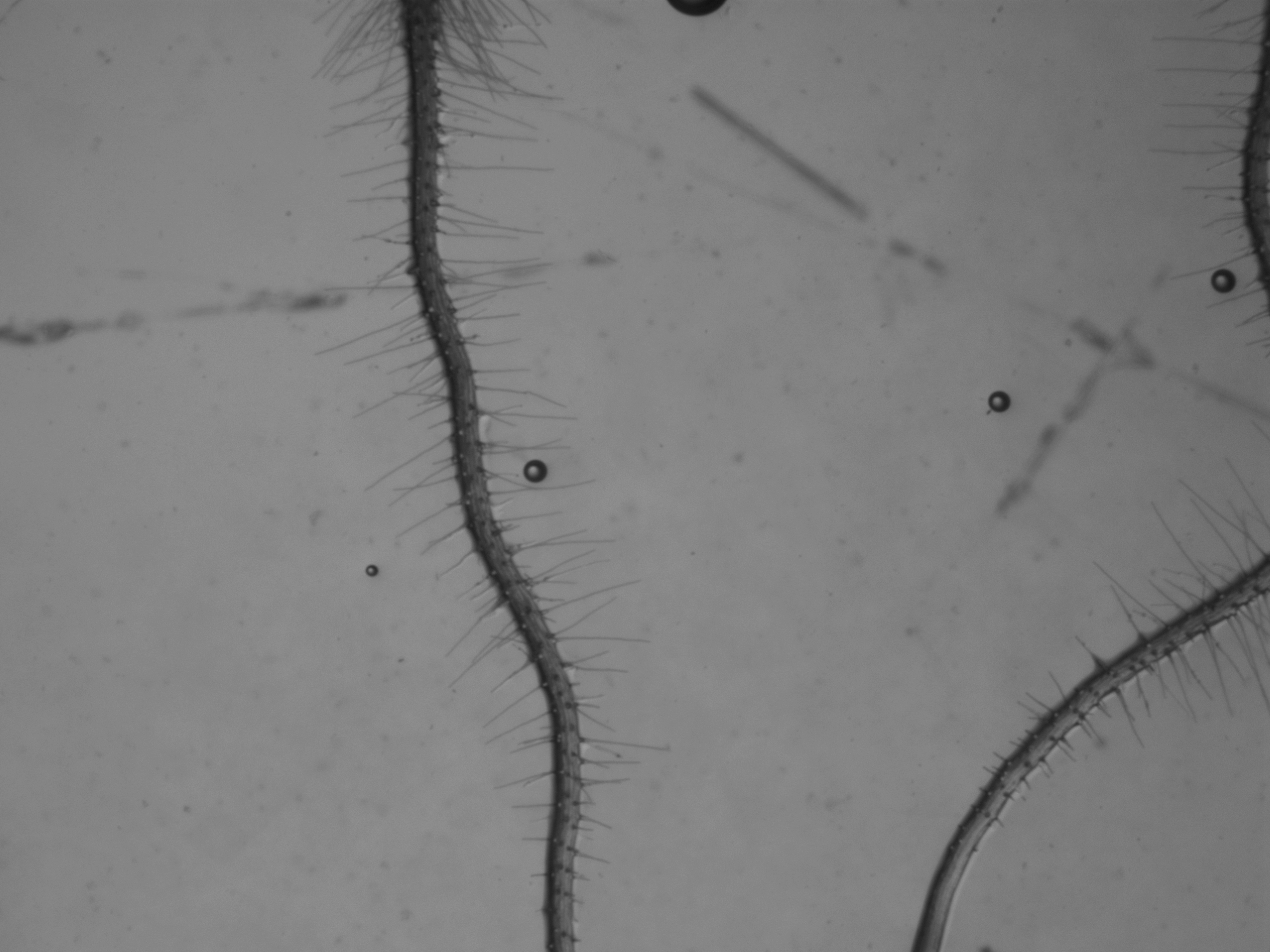

Supplement: Supplementary file 6 — Source data Fig. 4 [file 44318_2025_614_MOESM6_ESM.zip › Fig 4/Fig 4B/lrx1 rol23, PP2C12dead-GFP T2_1.tif]

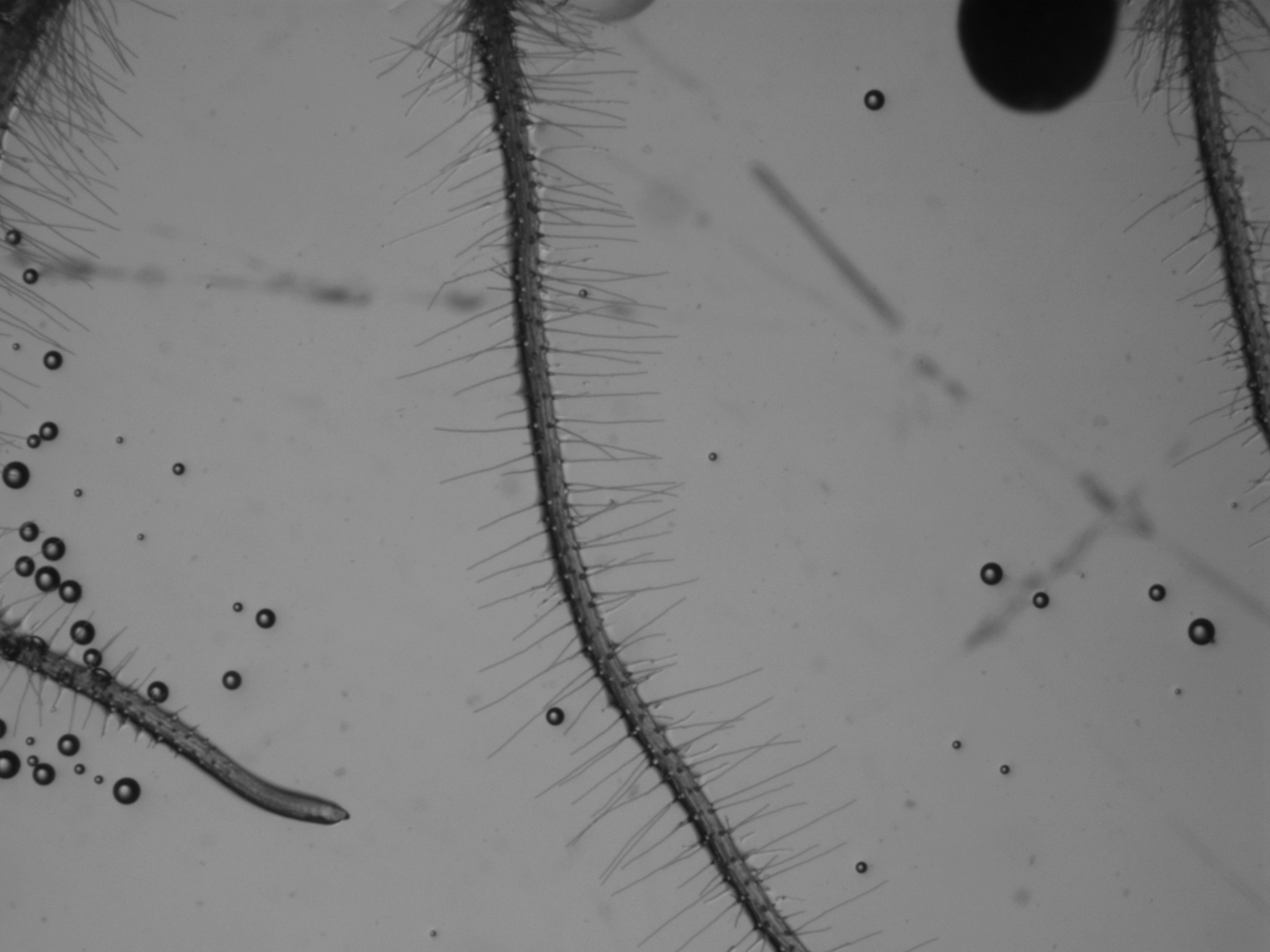

Supplement: Supplementary file 6 — Source data Fig. 4 [file 44318_2025_614_MOESM6_ESM.zip › Fig 4/Fig 4B/lrx1 rol23, PP2C12dead-GFP T2_2.tif]

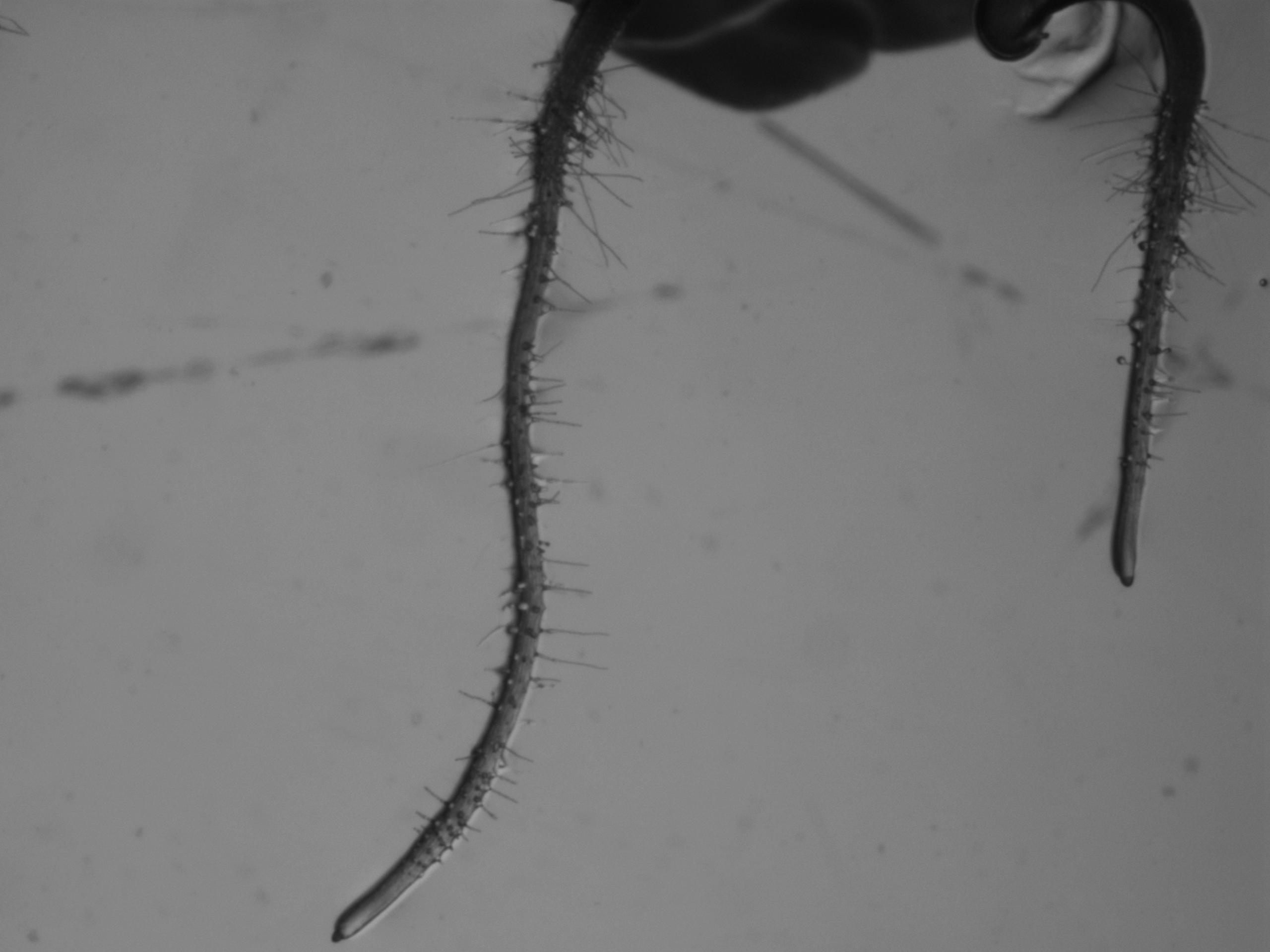

Supplement: Supplementary file 6 — Source data Fig. 4 [file 44318_2025_614_MOESM6_ESM.zip › Fig 4/Fig 4B/lrx1 rol23, PP2C12-GFP T2_1.png]

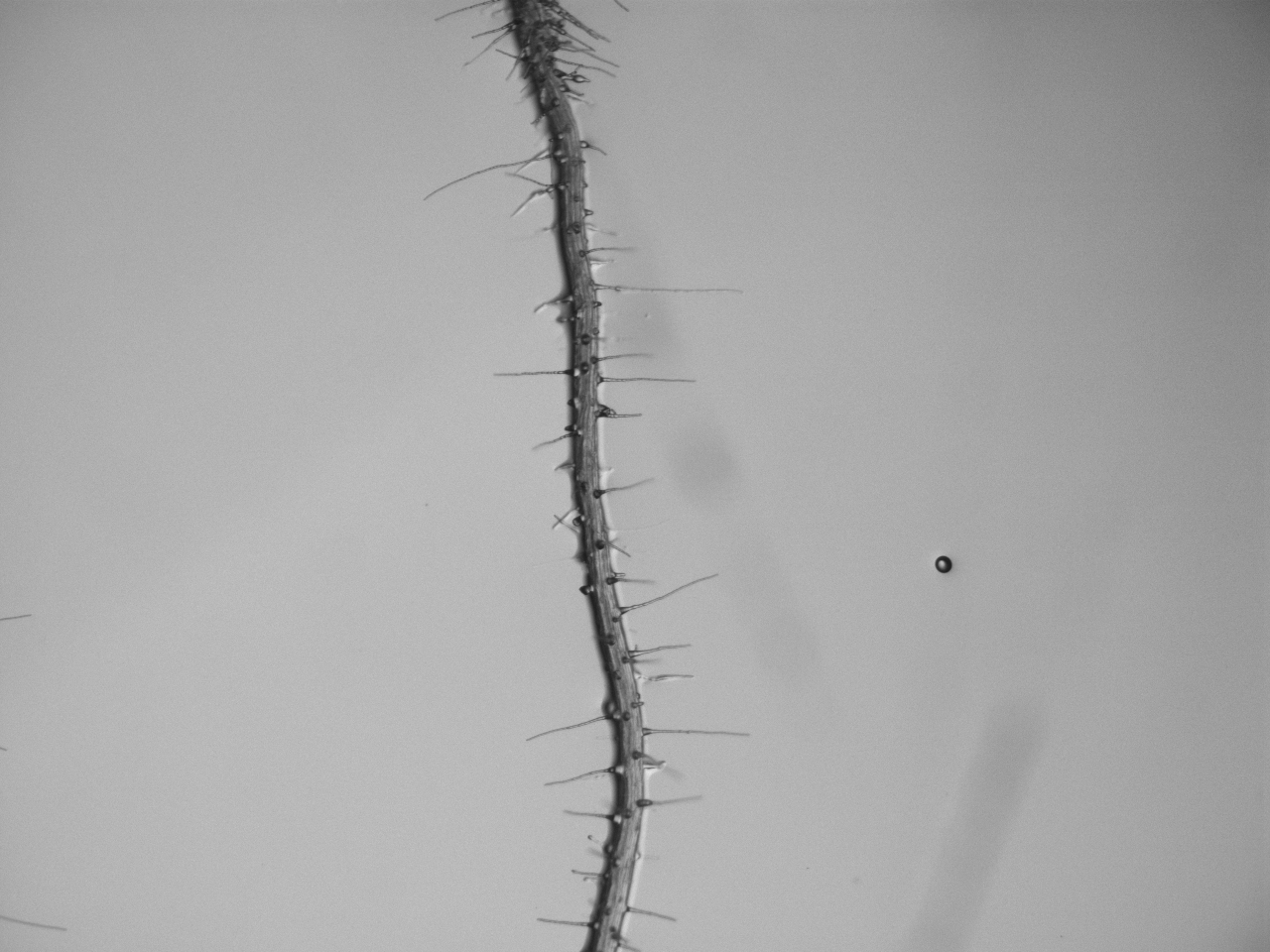

Supplement: Supplementary file 6 — Source data Fig. 4 [file 44318_2025_614_MOESM6_ESM.zip › Fig 4/Fig 4B/lrx1 rol23, PP2C12-GFP T2_4.tif]

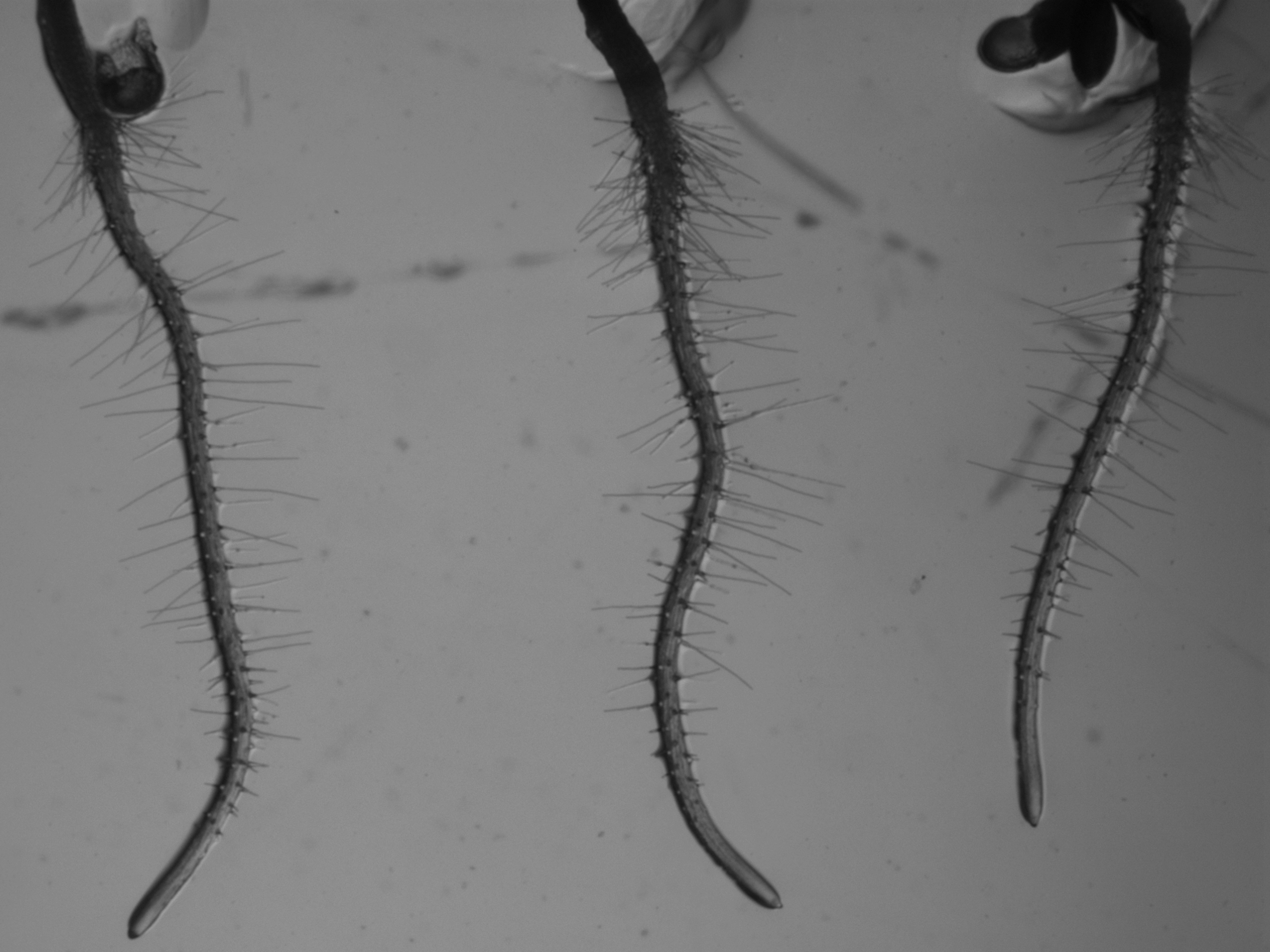

Supplement: Supplementary file 6 — Source data Fig. 4 [file 44318_2025_614_MOESM6_ESM.zip › Fig 4/Fig 4B/lrx1 rol23_2.tif]

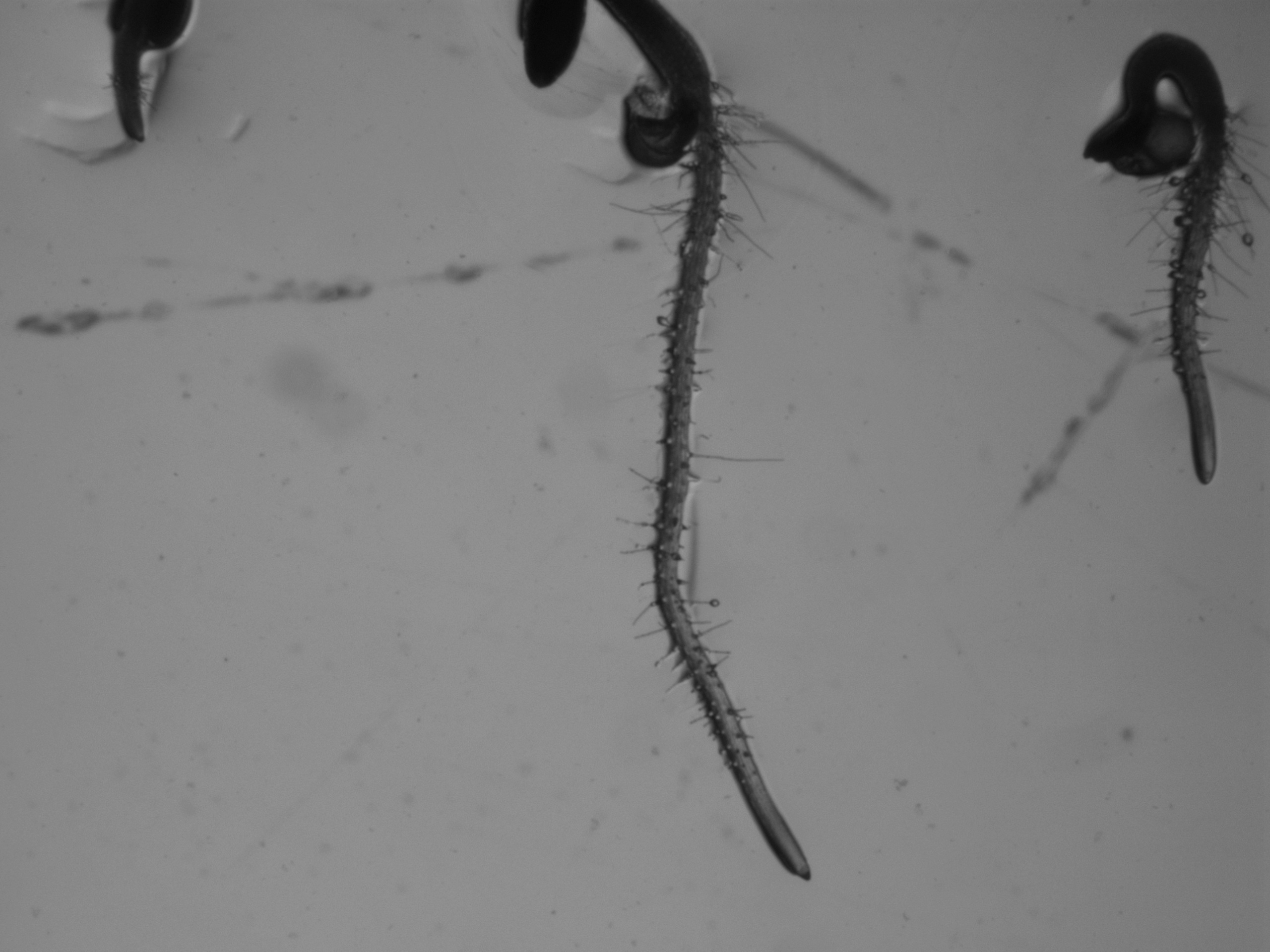

Supplement: Supplementary file 6 — Source data Fig. 4 [file 44318_2025_614_MOESM6_ESM.zip › Fig 4/Fig 4B/lrx1_3.tif]

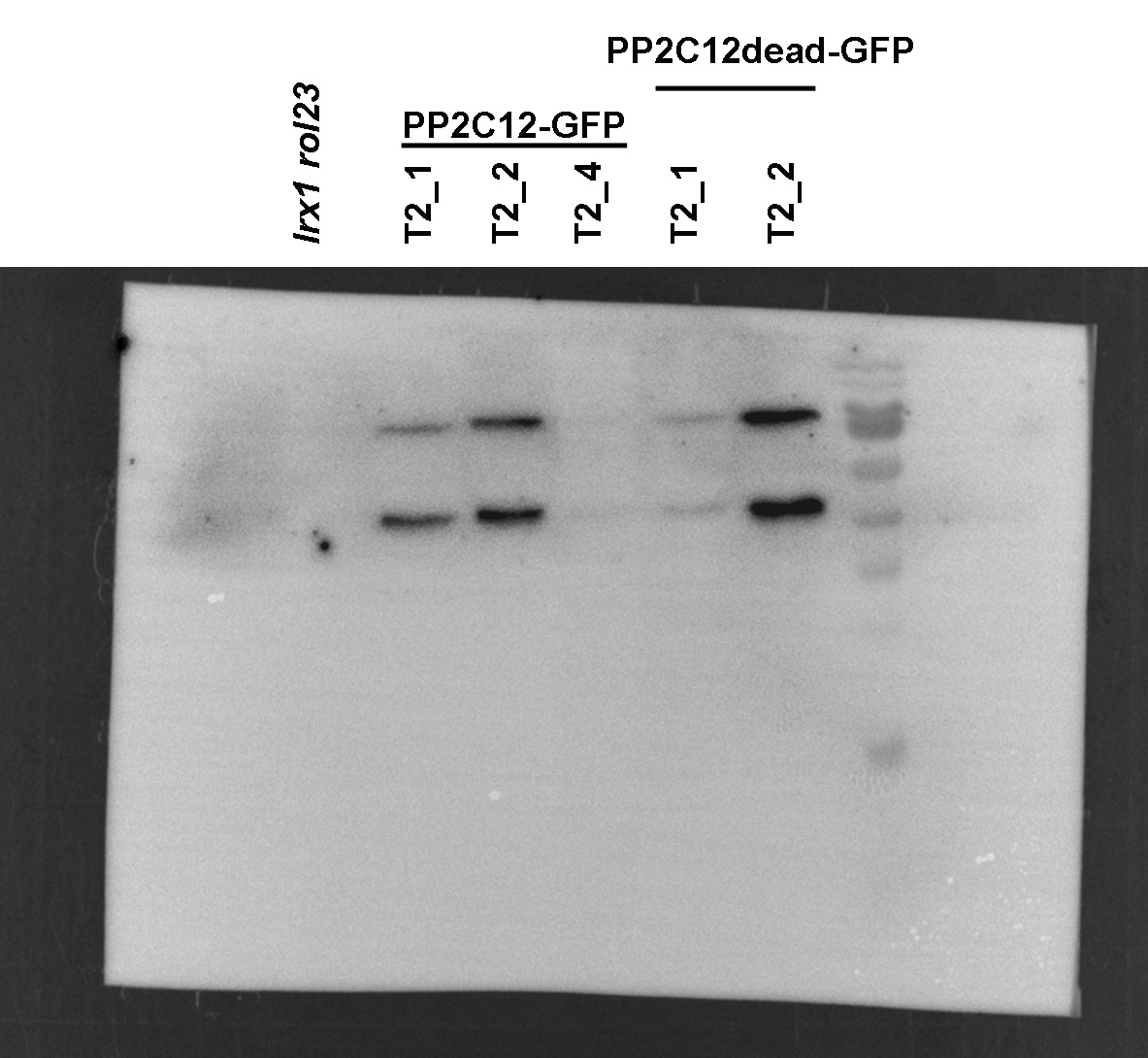

Supplement: Supplementary file 6 — Source data Fig. 4 [file 44318_2025_614_MOESM6_ESM.zip › Fig 4/Fig 4C.jpg]

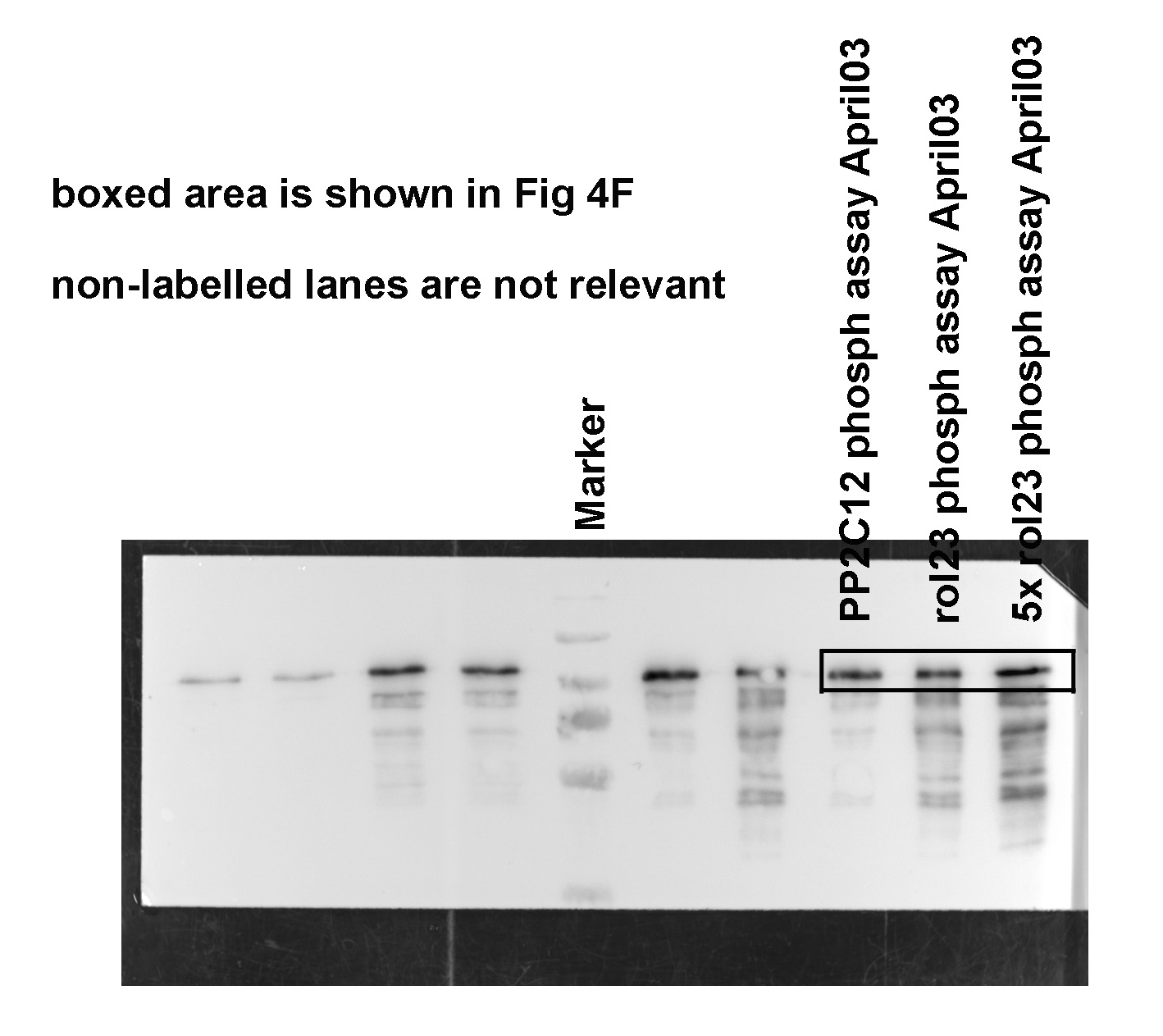

Supplement: Supplementary file 6 — Source data Fig. 4 [file 44318_2025_614_MOESM6_ESM.zip › Fig 4/Fig 4F immunoblot.jpg]

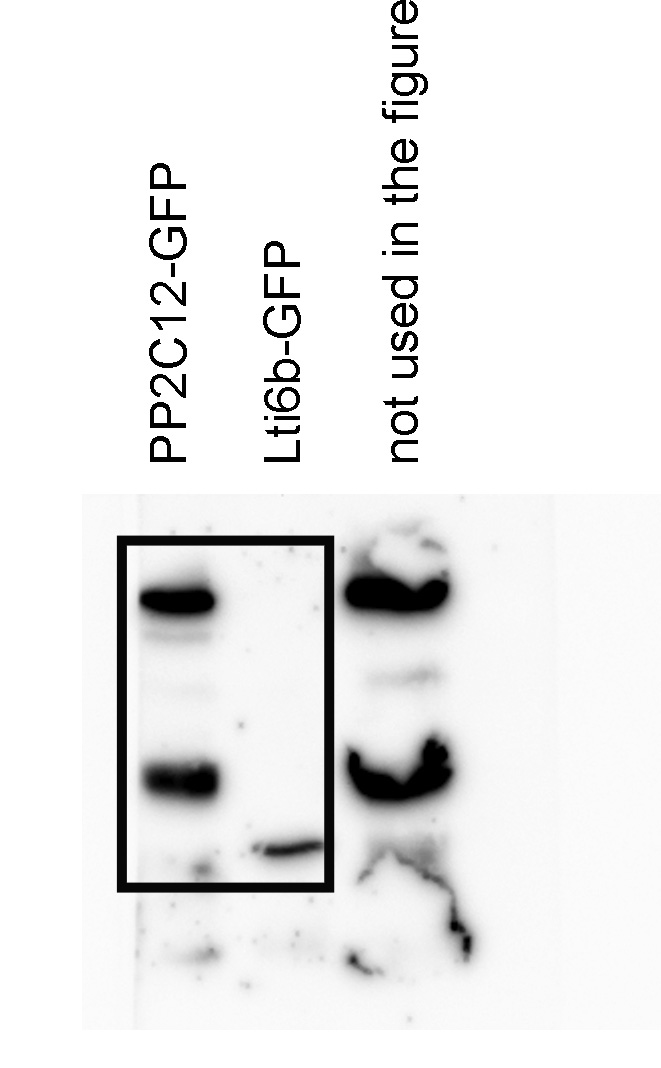

Supplement: Supplementary file 7 — Source data Fig. 5 [file 44318_2025_614_MOESM7_ESM.zip › Fig 5/Fig 5A/Arab anti-GFP lower panel.jpg]

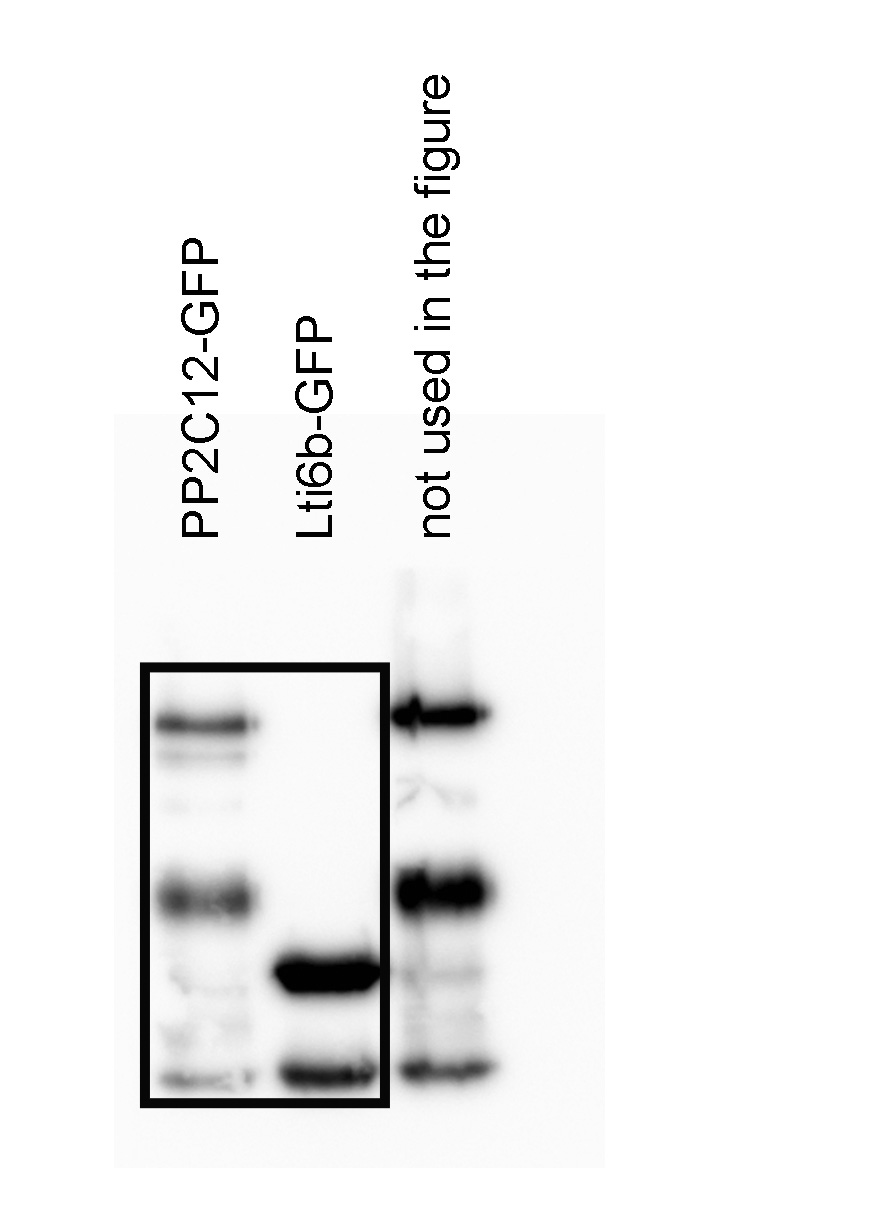

Supplement: Supplementary file 7 — Source data Fig. 5 [file 44318_2025_614_MOESM7_ESM.zip › Fig 5/Fig 5A/Arab anti-GFP top panel.jpg]

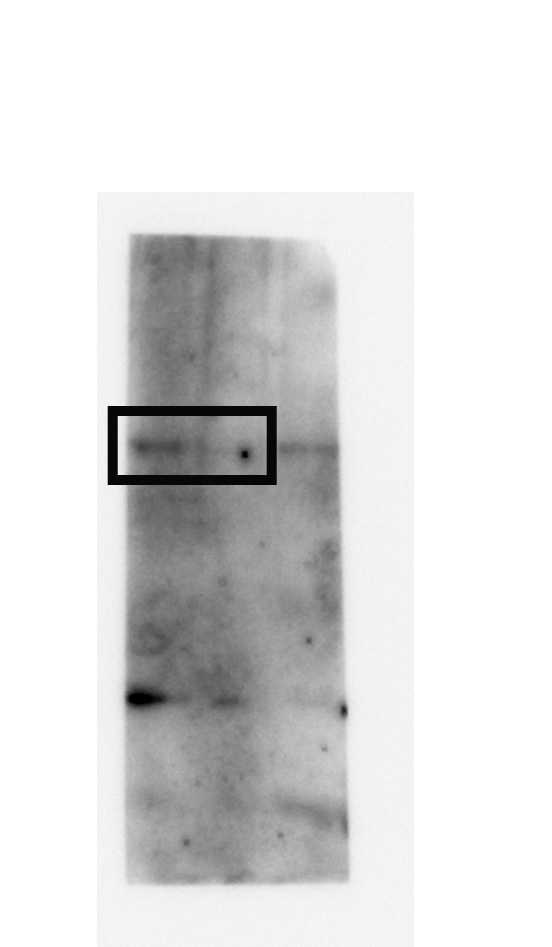

Supplement: Supplementary file 7 — Source data Fig. 5 [file 44318_2025_614_MOESM7_ESM.zip › Fig 5/Fig 5A/Arab FLAG top panel copy.jpg]

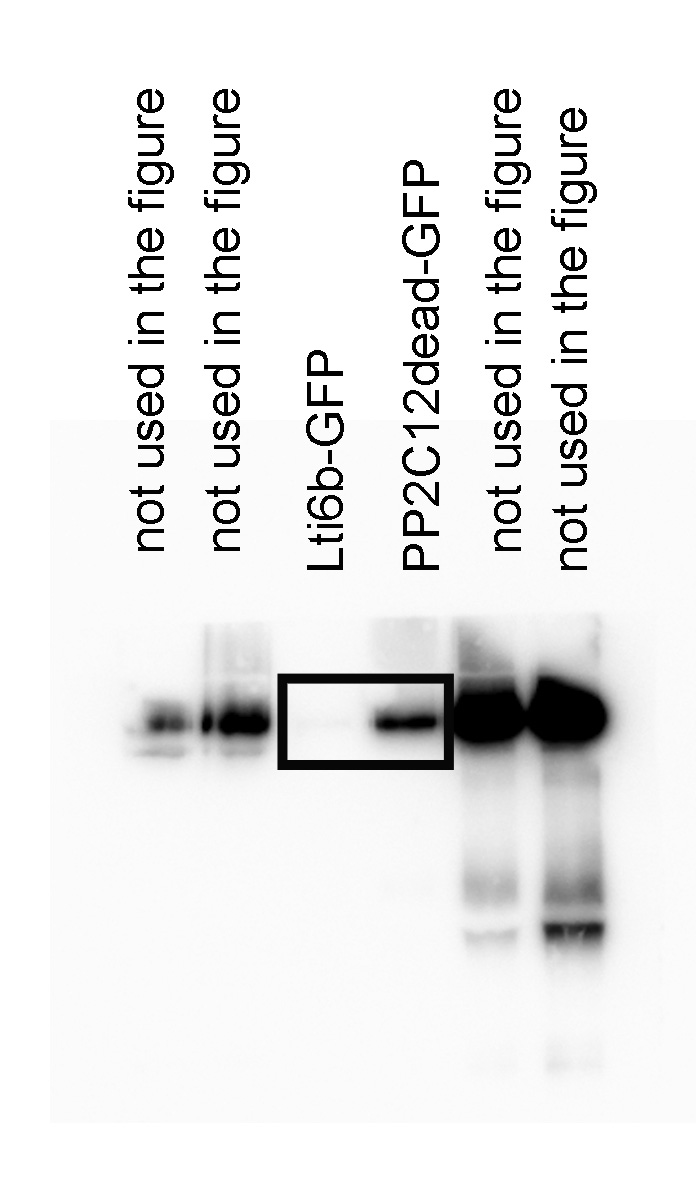

Supplement: Supplementary file 7 — Source data Fig. 5 [file 44318_2025_614_MOESM7_ESM.zip › Fig 5/Fig 5B/N benth anti-FLAG top panel.jpg]

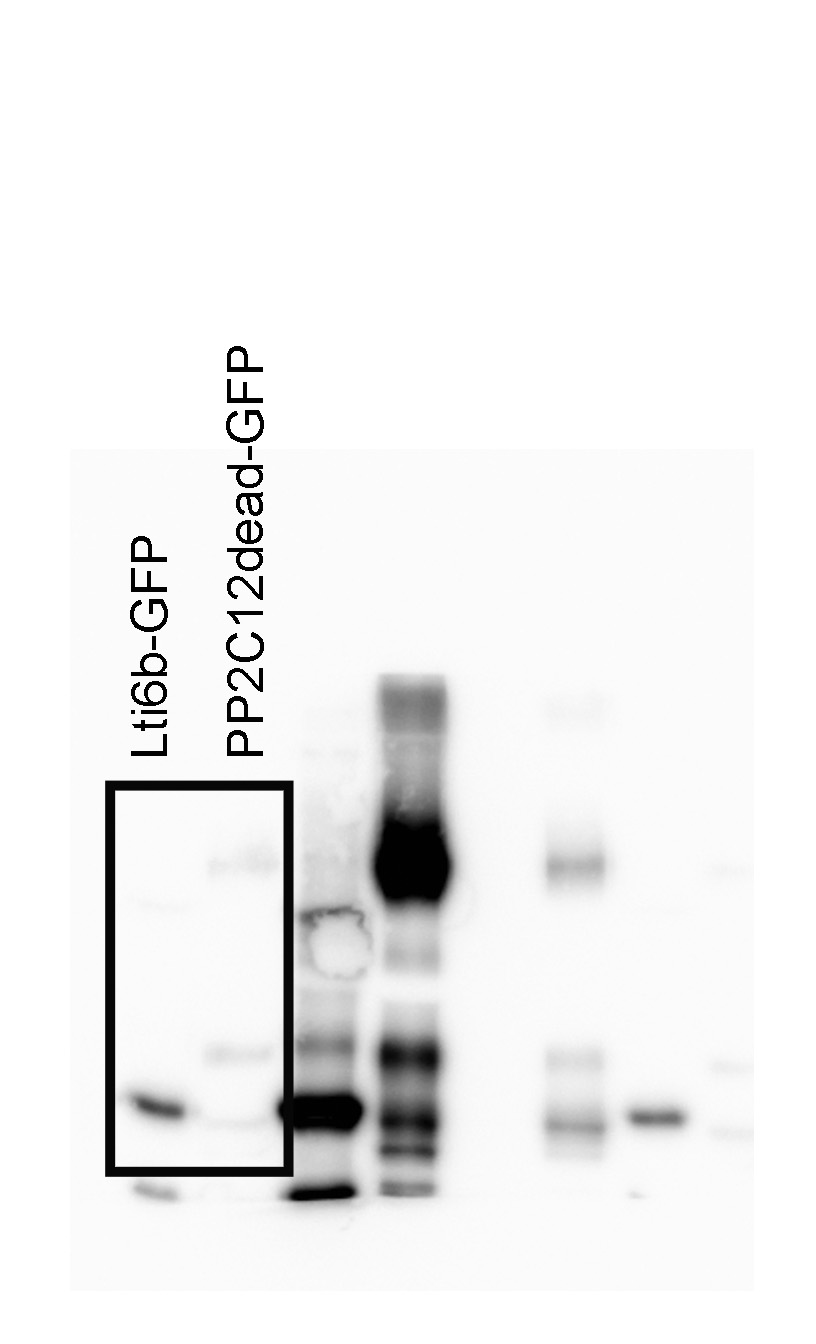

Supplement: Supplementary file 7 — Source data Fig. 5 [file 44318_2025_614_MOESM7_ESM.zip › Fig 5/Fig 5B/N benth anti-GFP lower panel.jpg]

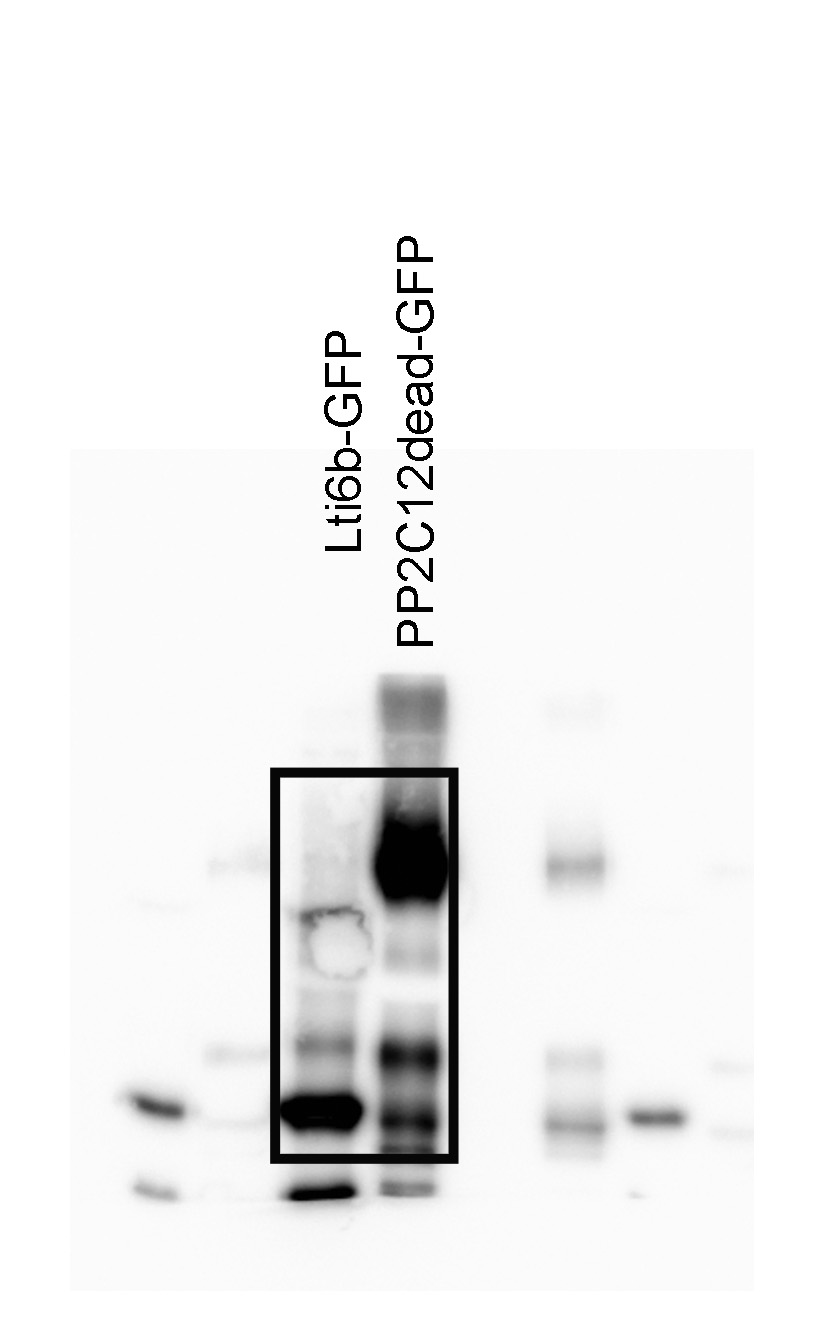

Supplement: Supplementary file 7 — Source data Fig. 5 [file 44318_2025_614_MOESM7_ESM.zip › Fig 5/Fig 5B/N benth anti-GFP top panel.jpg]

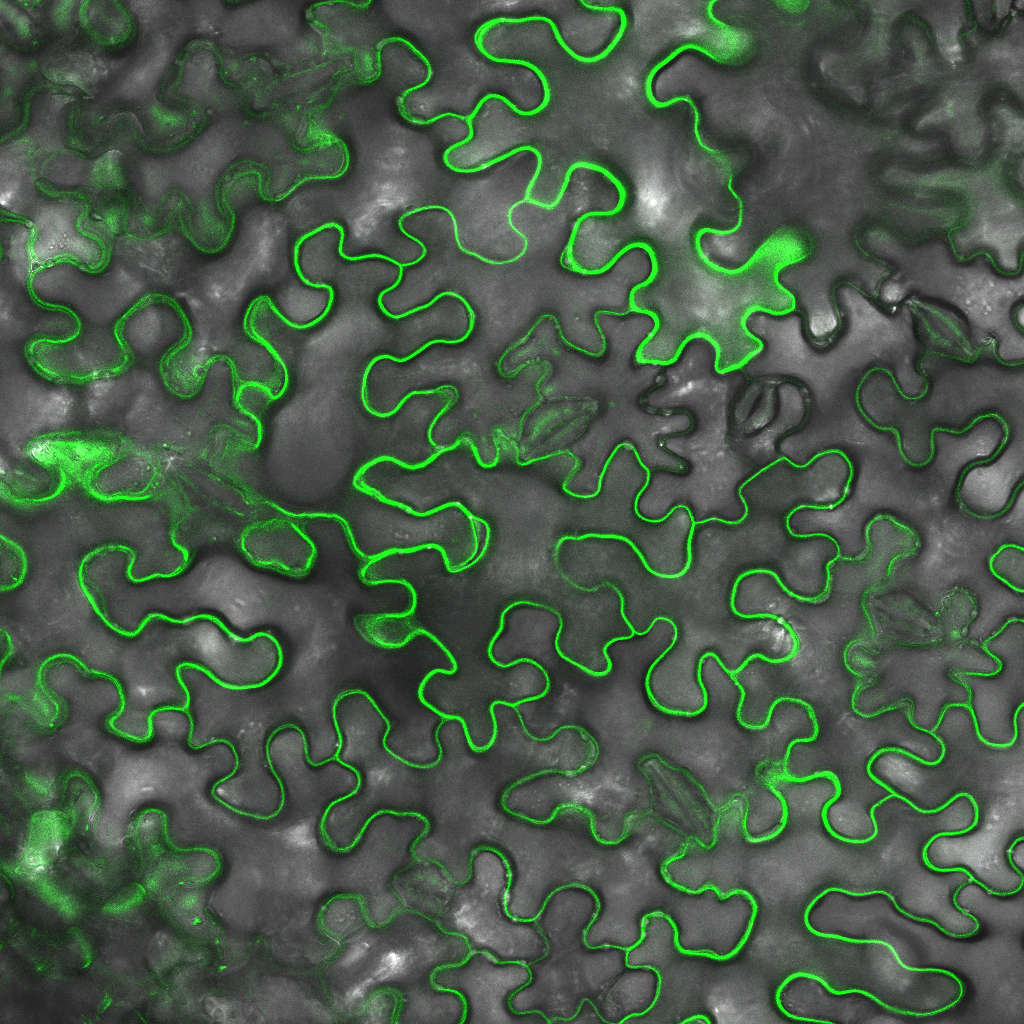

Supplement: Supplementary file 7 — Source data Fig. 5 [file 44318_2025_614_MOESM7_ESM.zip › Fig 5/Fig 5C/1.2_FER YCE+ROL23 YNE/Image002_000.tif]

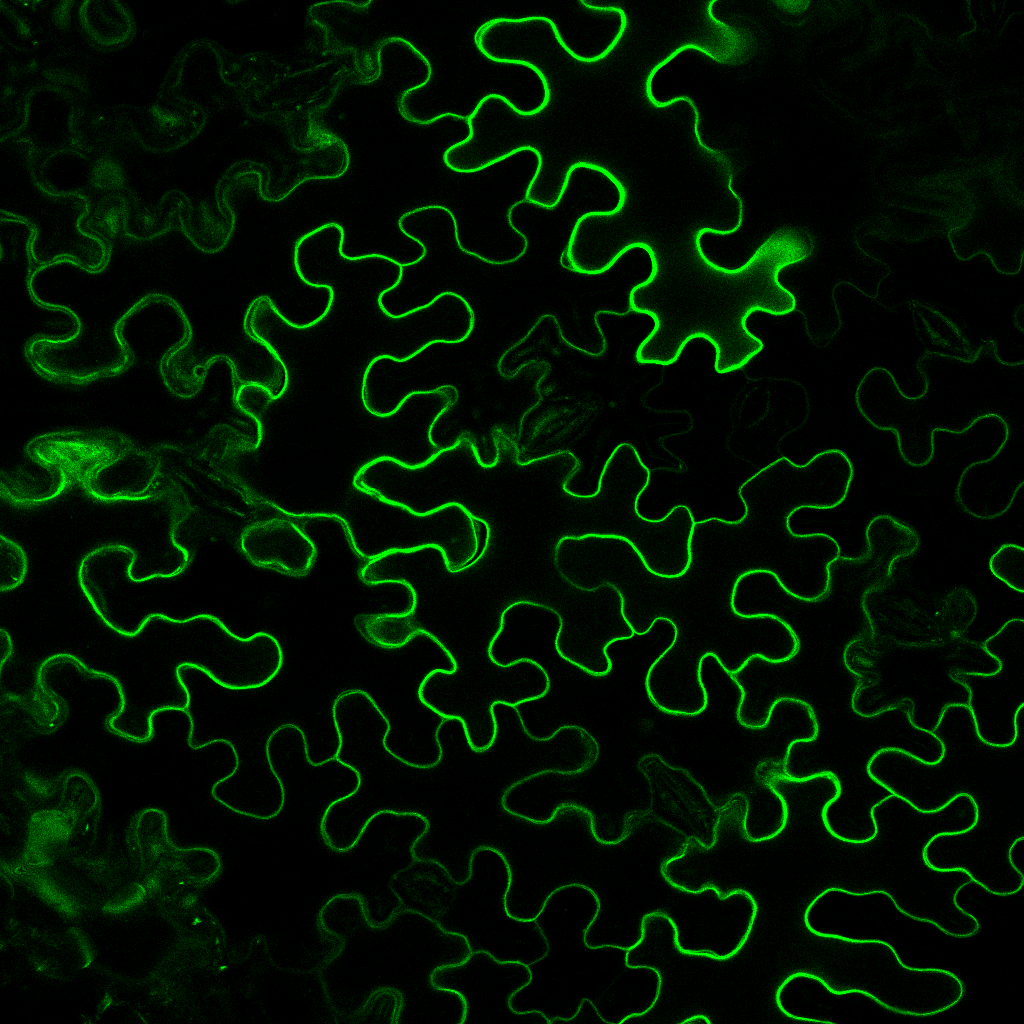

Supplement: Supplementary file 7 — Source data Fig. 5 [file 44318_2025_614_MOESM7_ESM.zip › Fig 5/Fig 5C/1.2_FER YCE+ROL23 YNE/Image002_000_ch00.tif]

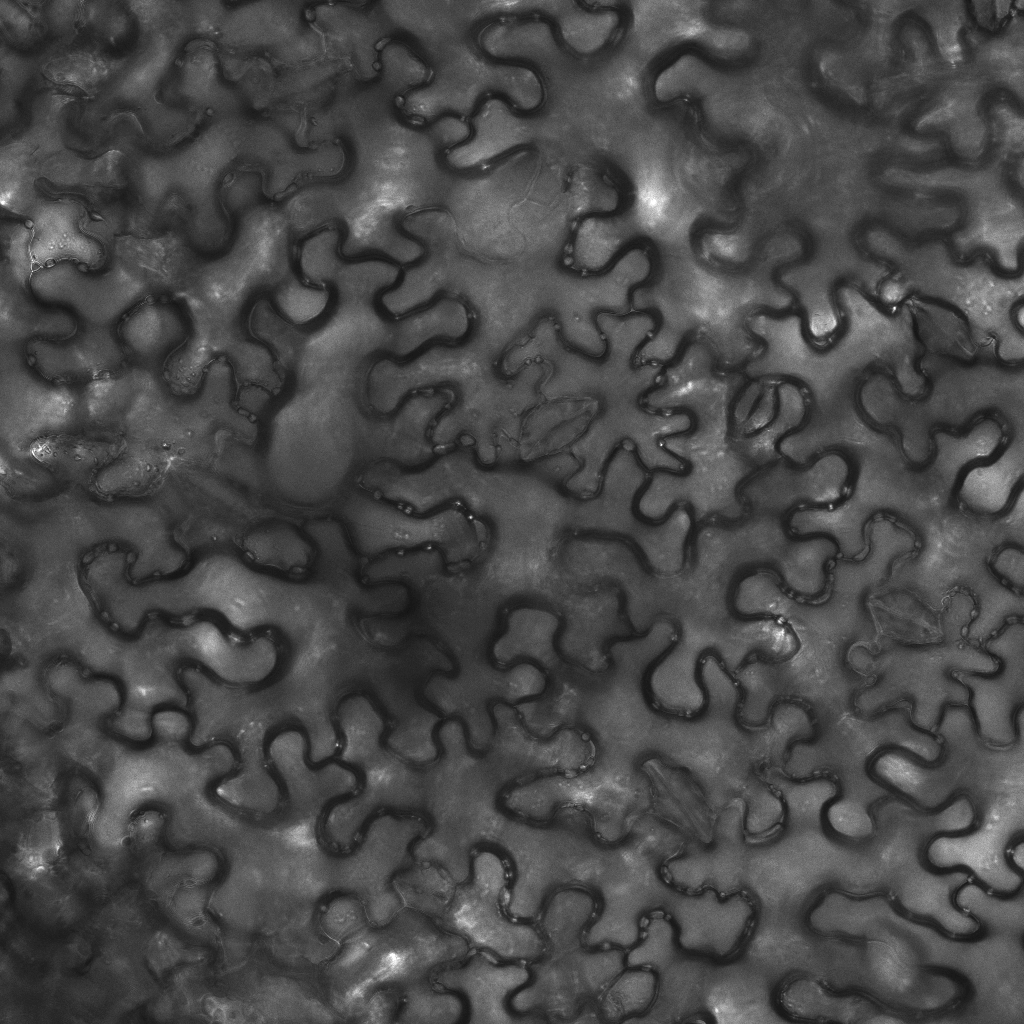

Supplement: Supplementary file 7 — Source data Fig. 5 [file 44318_2025_614_MOESM7_ESM.zip › Fig 5/Fig 5C/1.2_FER YCE+ROL23 YNE/Image002_000_ch01.tif]

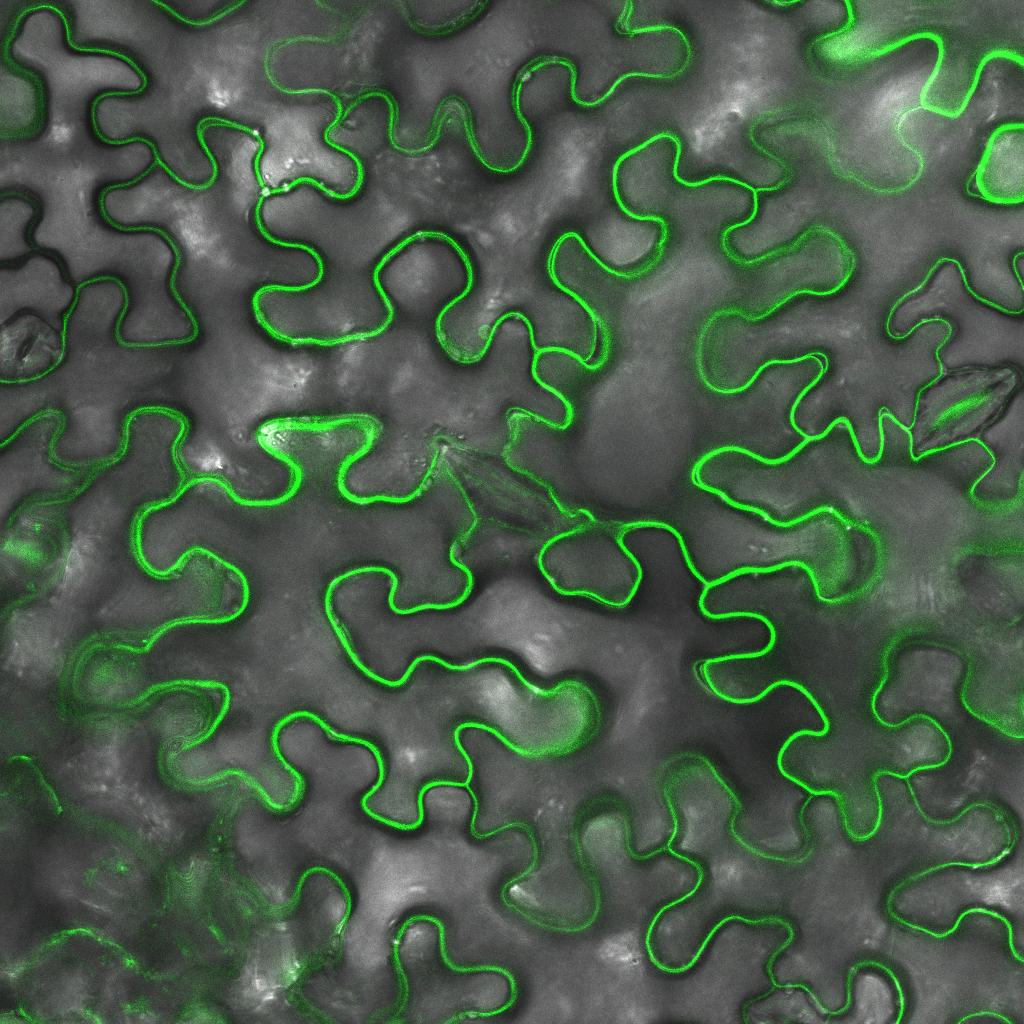

Supplement: Supplementary file 7 — Source data Fig. 5 [file 44318_2025_614_MOESM7_ESM.zip › Fig 5/Fig 5C/1.2_FER YCE+ROL23 YNE/Image004.tif]

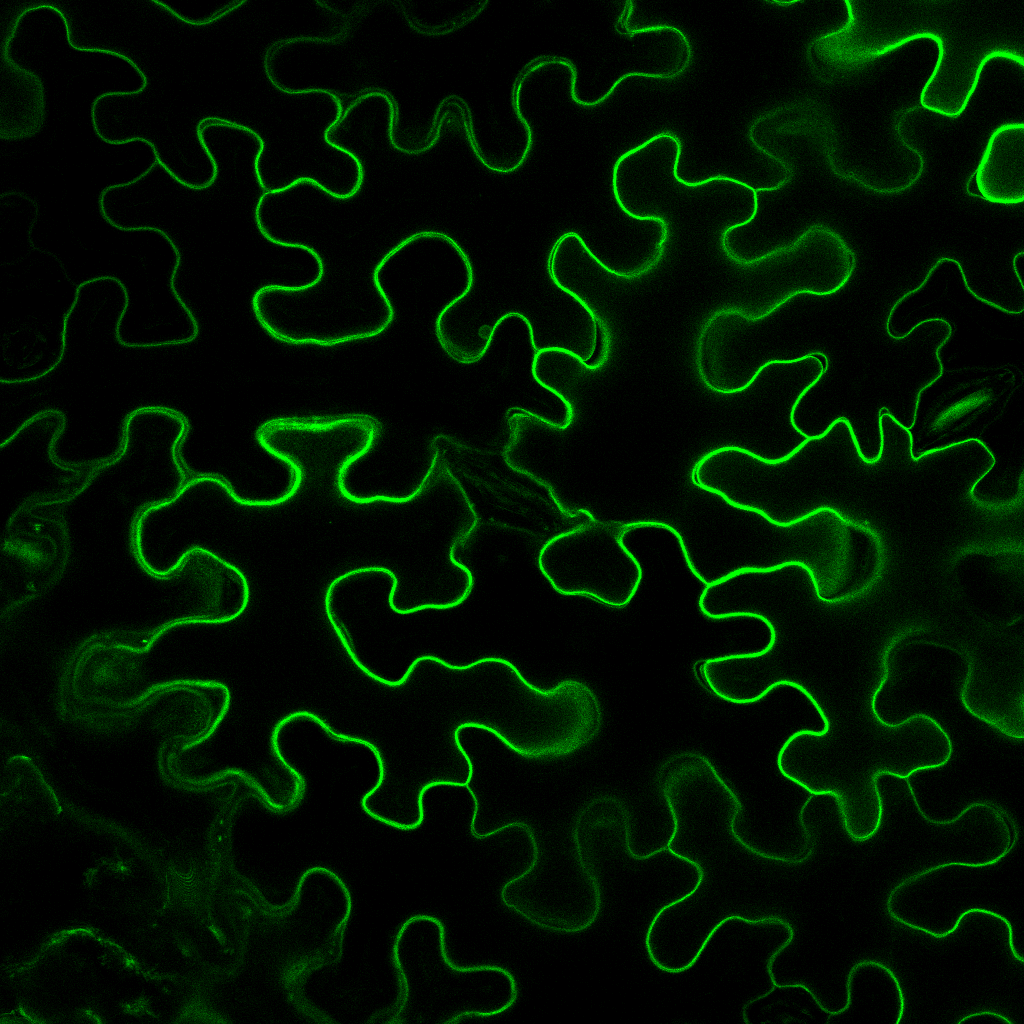

Supplement: Supplementary file 7 — Source data Fig. 5 [file 44318_2025_614_MOESM7_ESM.zip › Fig 5/Fig 5C/1.2_FER YCE+ROL23 YNE/Image004_ch00.tif]

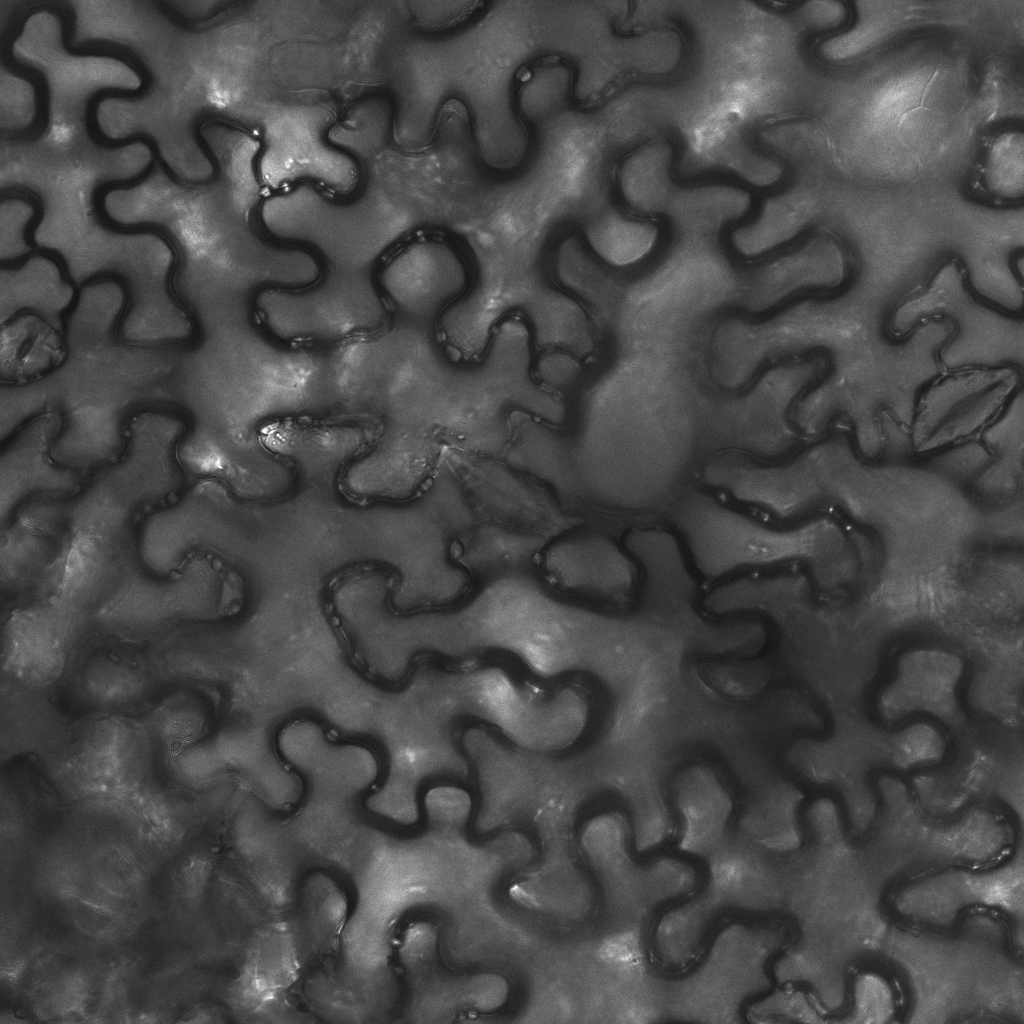

Supplement: Supplementary file 7 — Source data Fig. 5 [file 44318_2025_614_MOESM7_ESM.zip › Fig 5/Fig 5C/1.2_FER YCE+ROL23 YNE/Image004_ch01.tif]

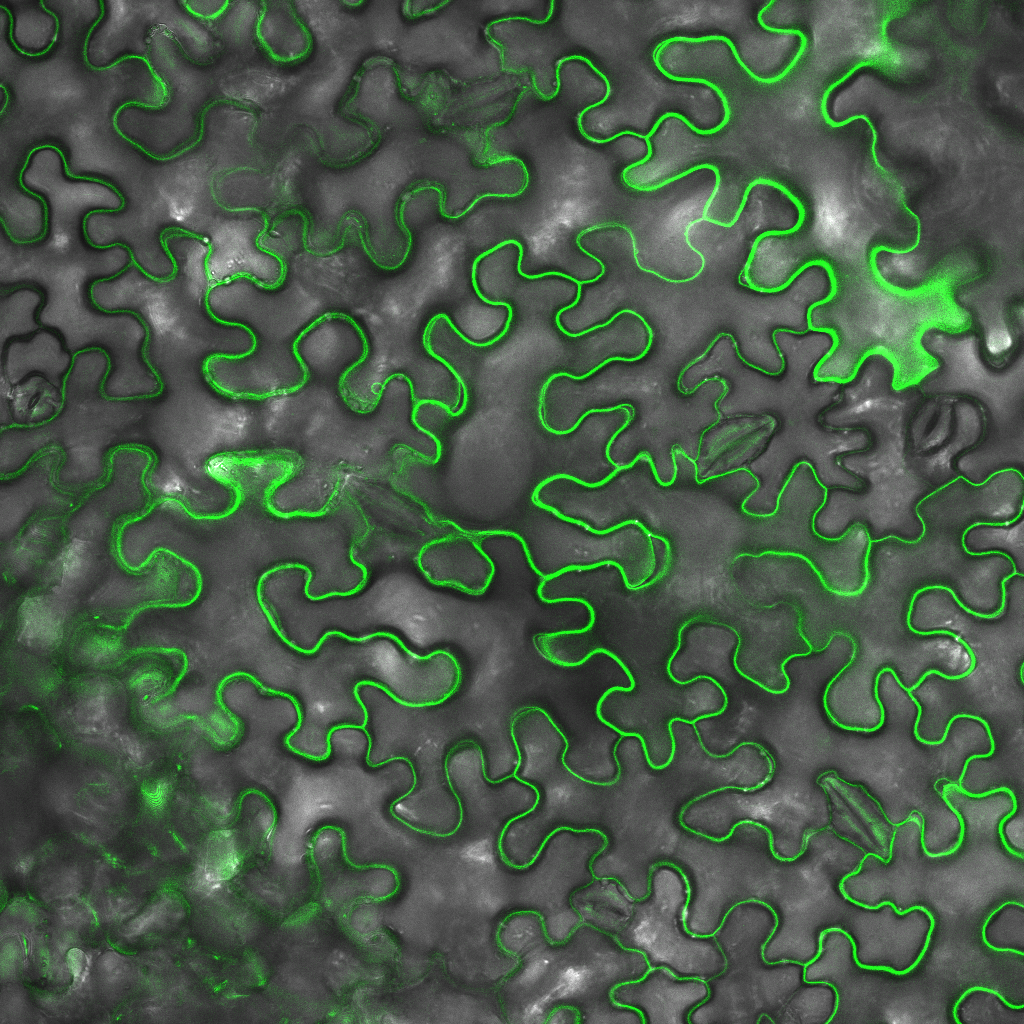

Supplement: Supplementary file 7 — Source data Fig. 5 [file 44318_2025_614_MOESM7_ESM.zip › Fig 5/Fig 5C/1.2_FER YCE+ROL23 YNE/Image005.tif]

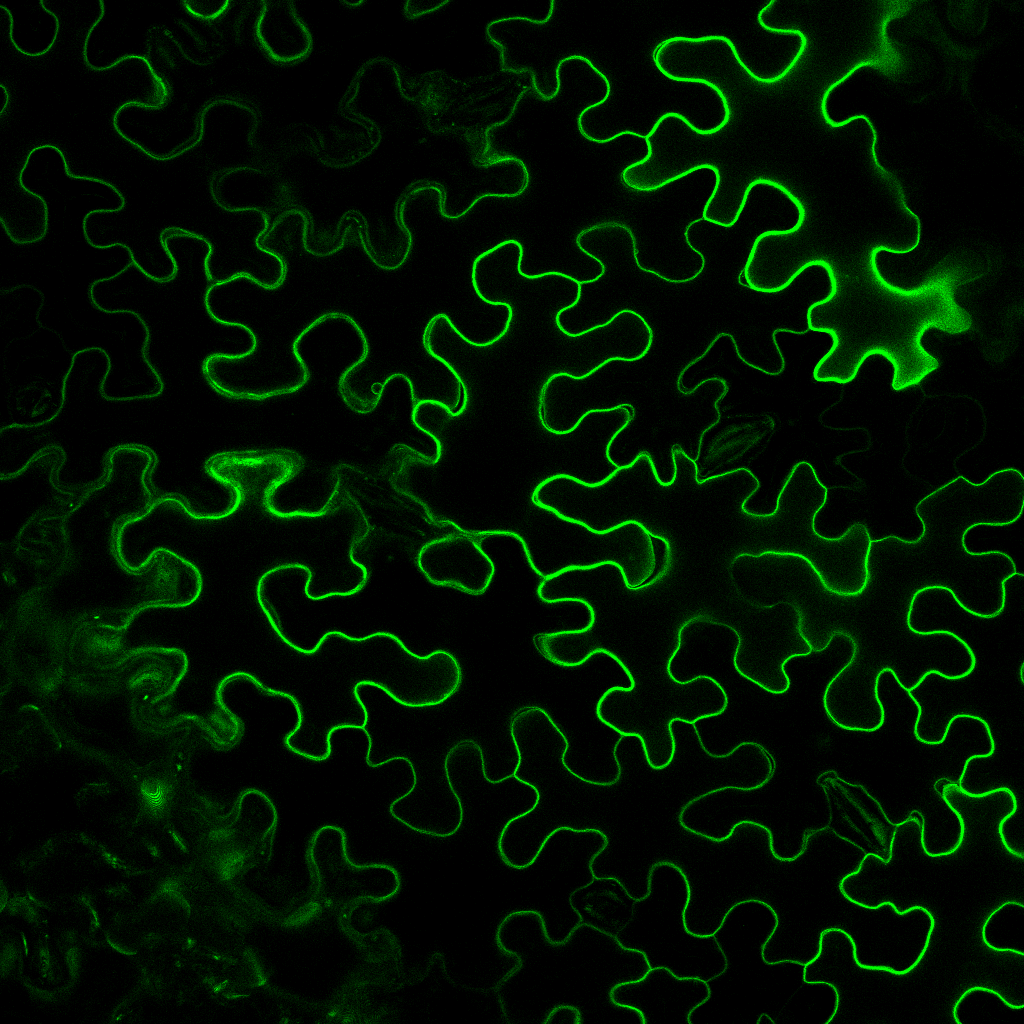

Supplement: Supplementary file 7 — Source data Fig. 5 [file 44318_2025_614_MOESM7_ESM.zip › Fig 5/Fig 5C/1.2_FER YCE+ROL23 YNE/Image005_ch00.tif]

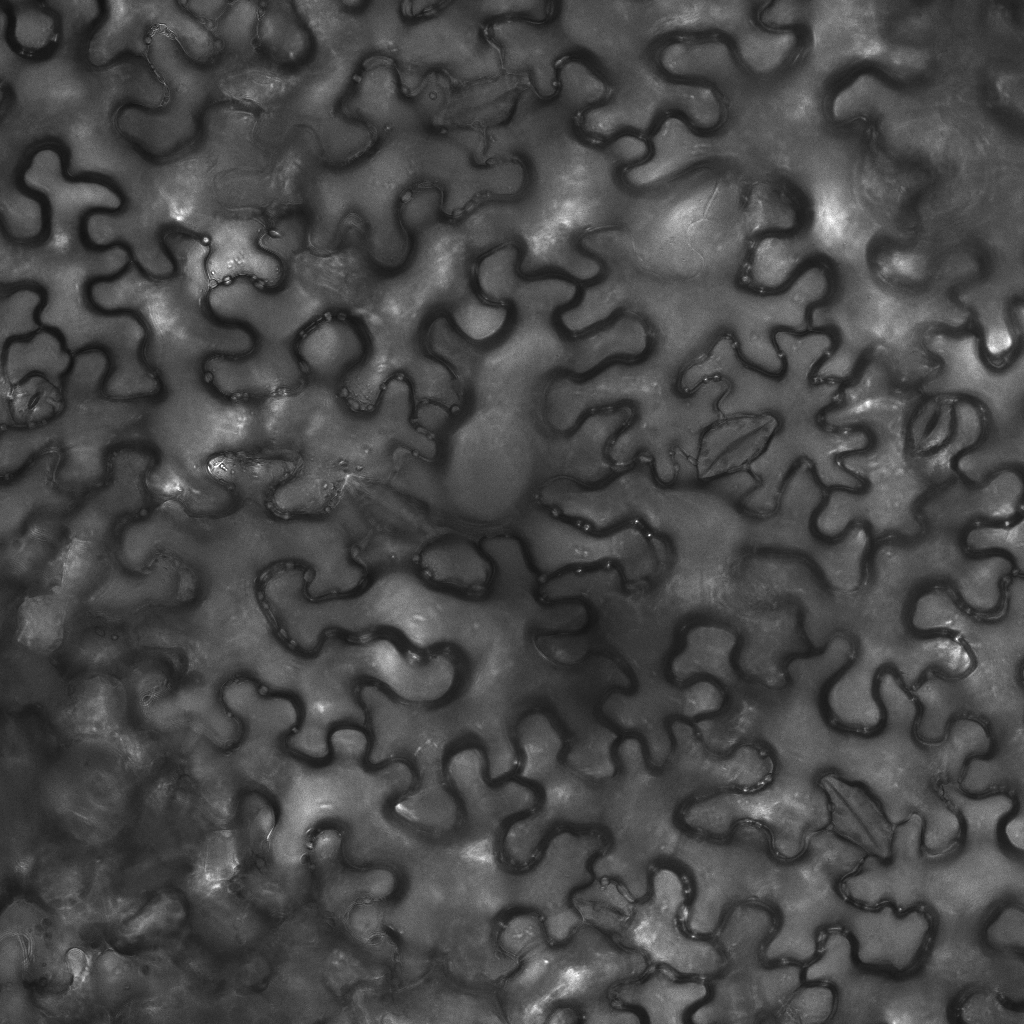

Supplement: Supplementary file 7 — Source data Fig. 5 [file 44318_2025_614_MOESM7_ESM.zip › Fig 5/Fig 5C/1.2_FER YCE+ROL23 YNE/Image005_ch01.tif]

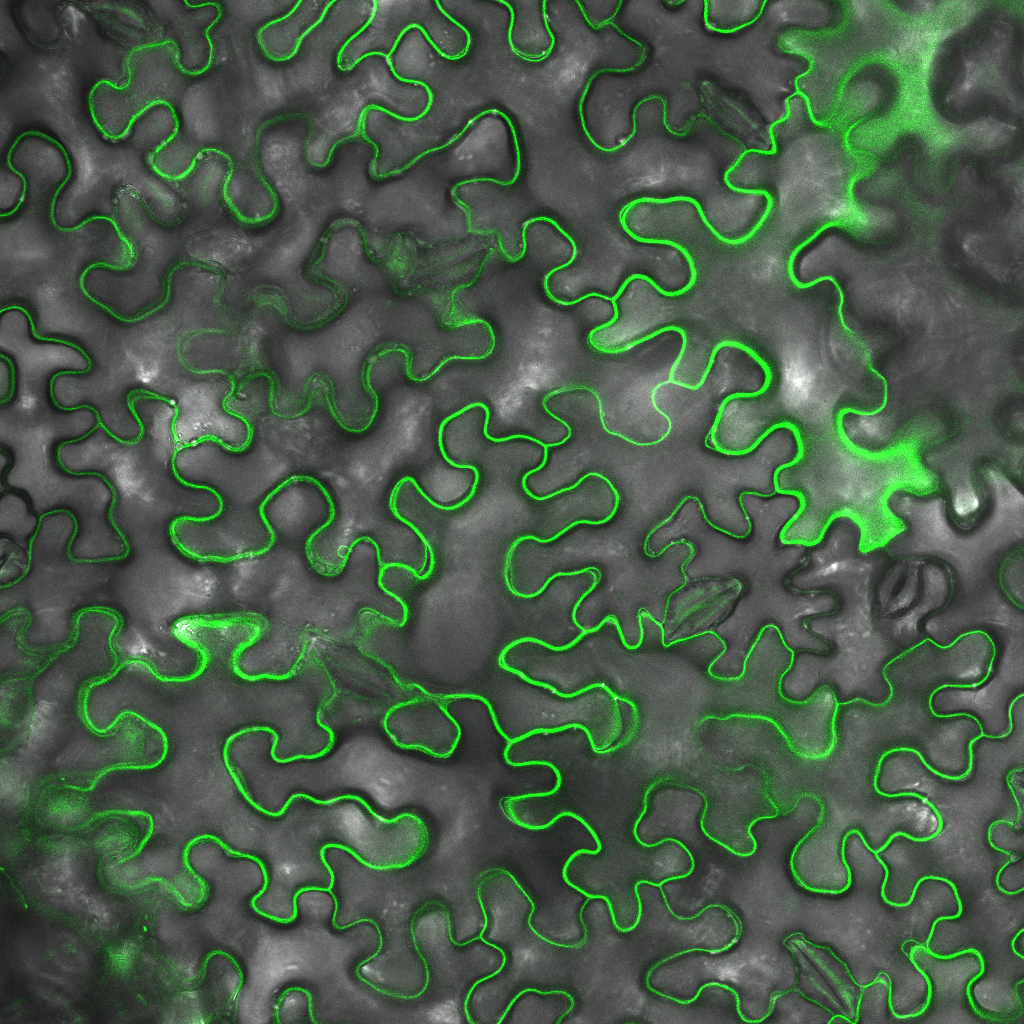

Supplement: Supplementary file 7 — Source data Fig. 5 [file 44318_2025_614_MOESM7_ESM.zip › Fig 5/Fig 5C/1.2_FER YCE+ROL23 YNE/Image006.tif]

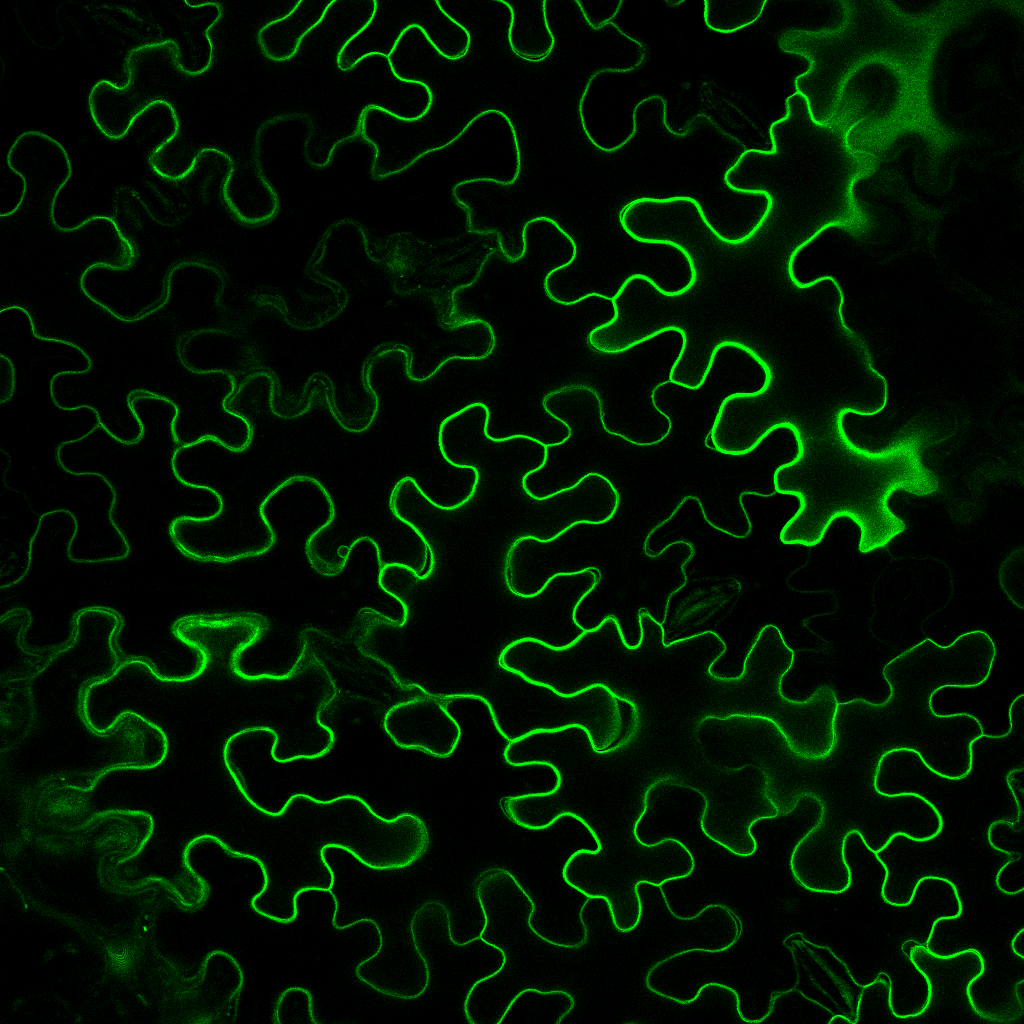

Supplement: Supplementary file 7 — Source data Fig. 5 [file 44318_2025_614_MOESM7_ESM.zip › Fig 5/Fig 5C/1.2_FER YCE+ROL23 YNE/Image006_ch00.tif]

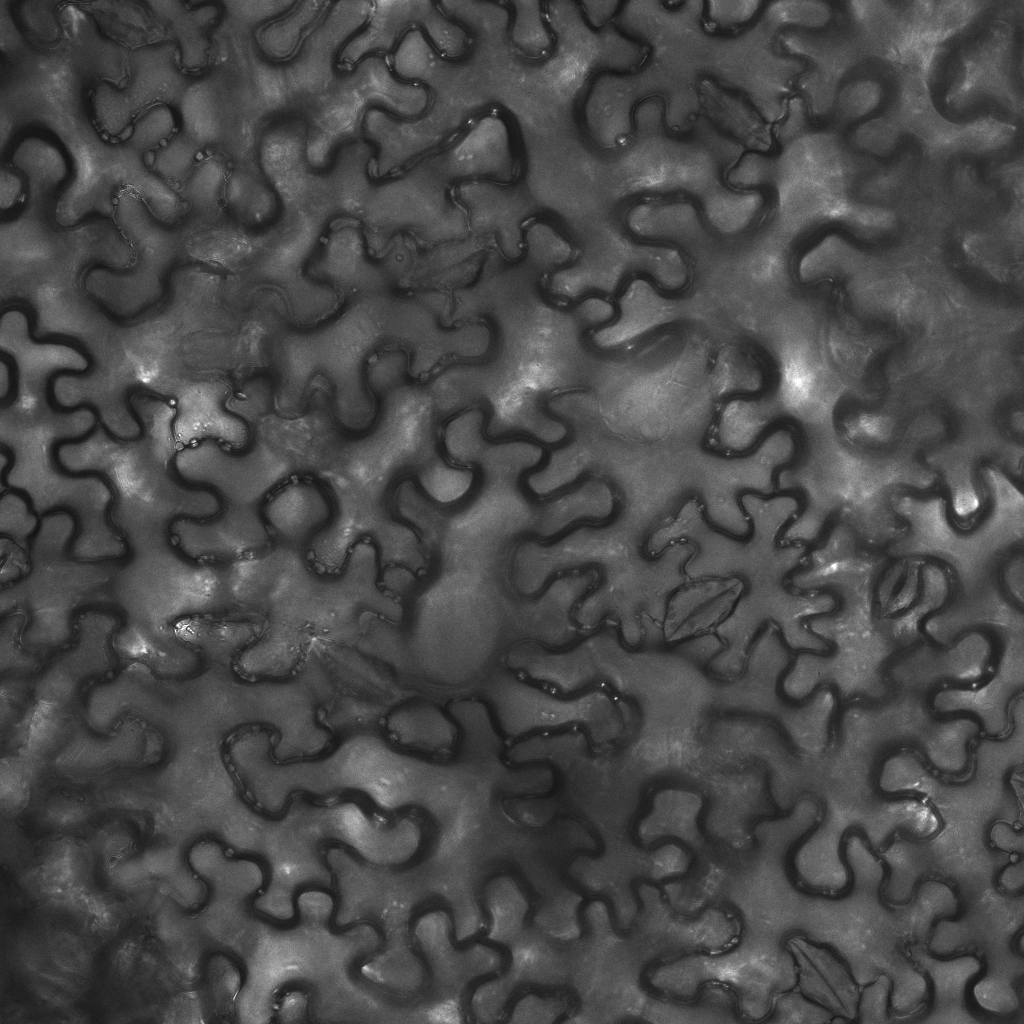

Supplement: Supplementary file 7 — Source data Fig. 5 [file 44318_2025_614_MOESM7_ESM.zip › Fig 5/Fig 5C/1.2_FER YCE+ROL23 YNE/Image006_ch01.tif]

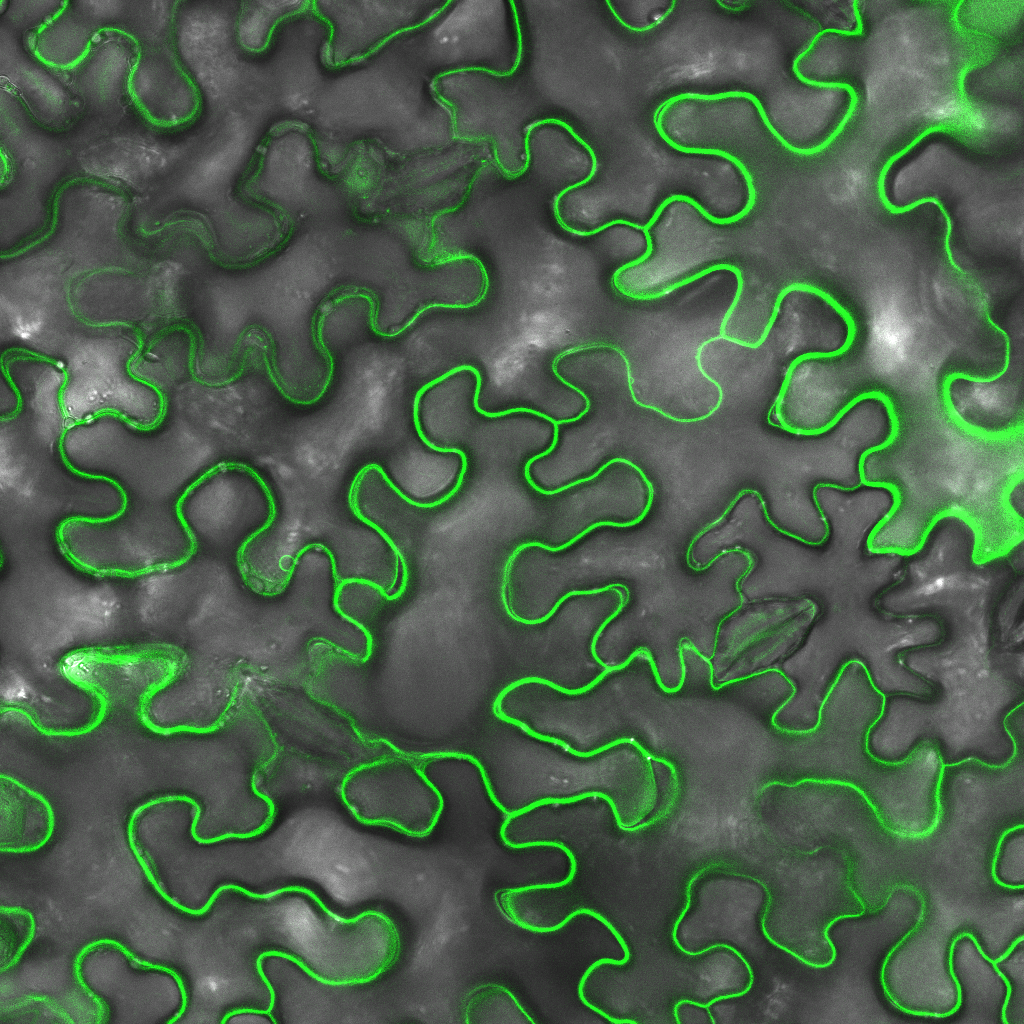

Supplement: Supplementary file 7 — Source data Fig. 5 [file 44318_2025_614_MOESM7_ESM.zip › Fig 5/Fig 5C/1.2_FER YCE+ROL23 YNE/Image007.tif]

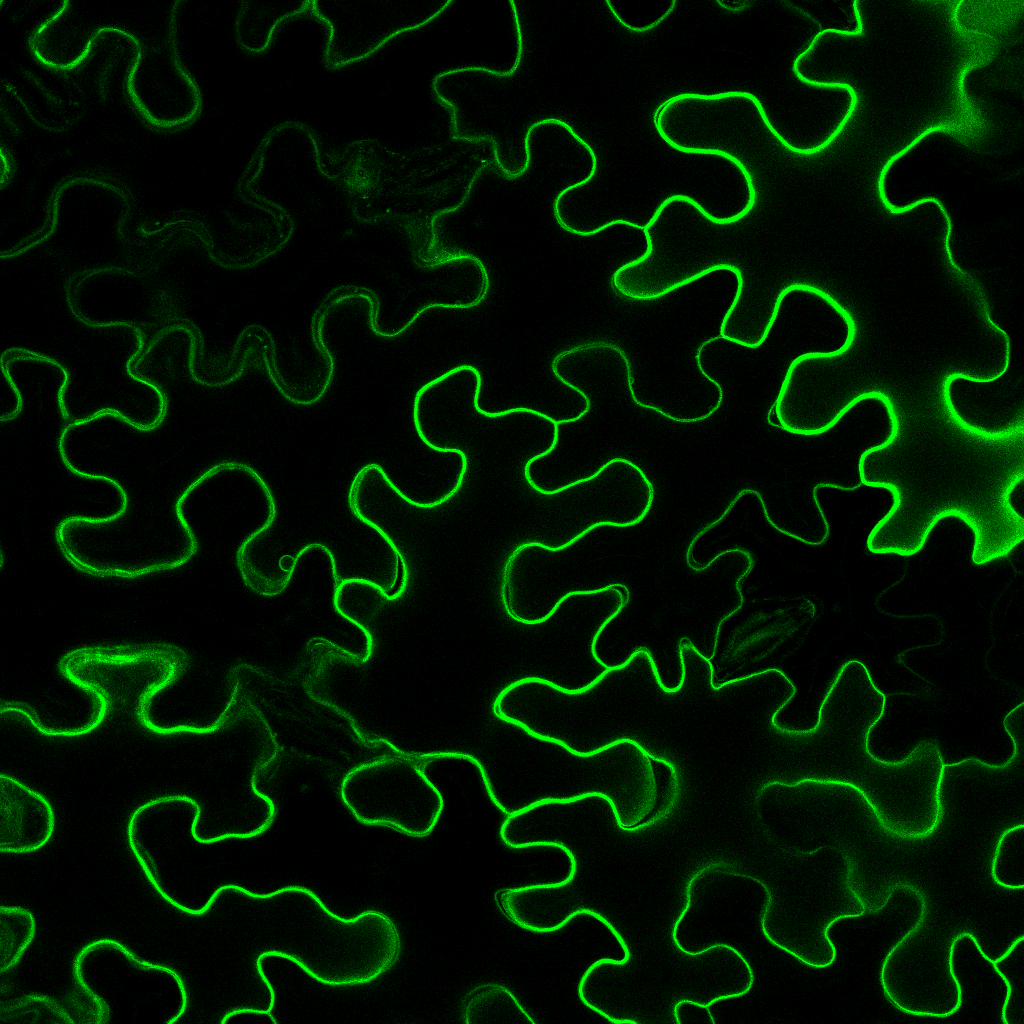

Supplement: Supplementary file 7 — Source data Fig. 5 [file 44318_2025_614_MOESM7_ESM.zip › Fig 5/Fig 5C/1.2_FER YCE+ROL23 YNE/Image007_ch00.tif]

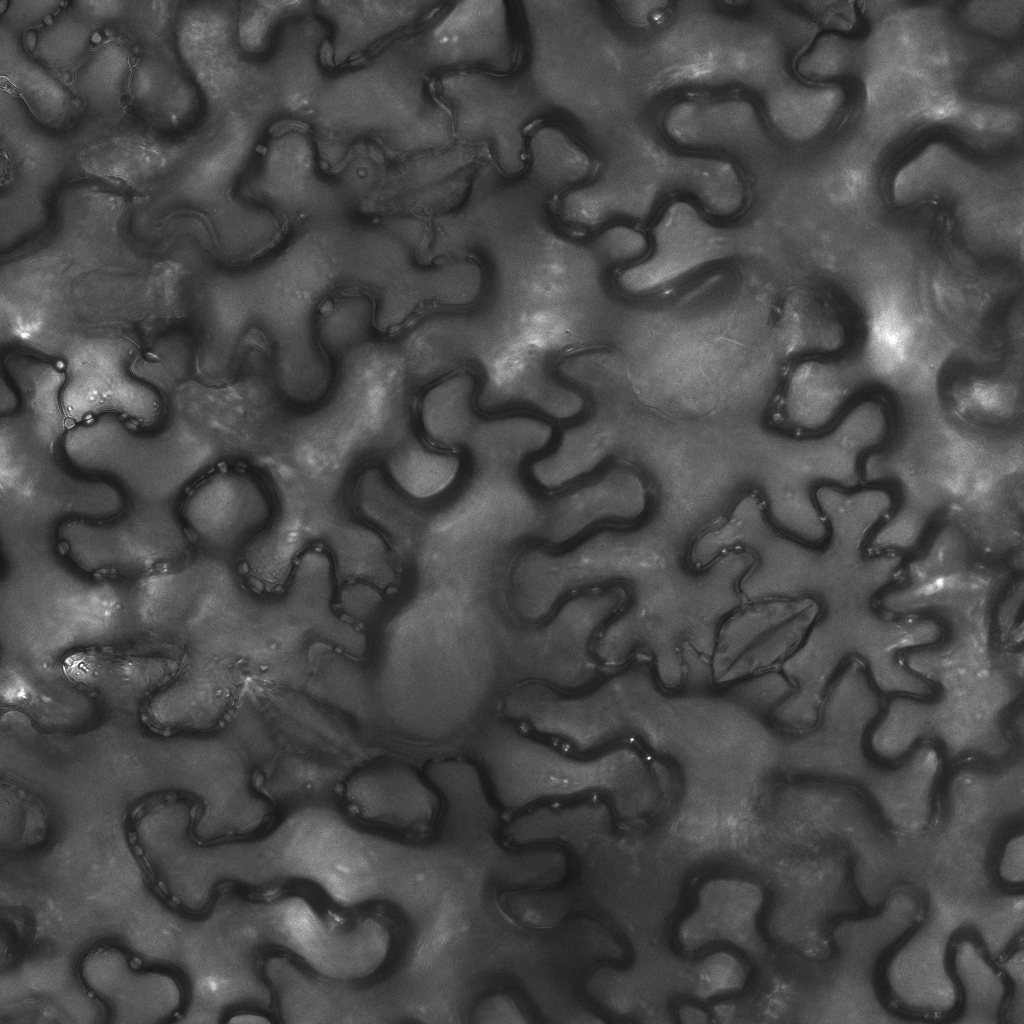

Supplement: Supplementary file 7 — Source data Fig. 5 [file 44318_2025_614_MOESM7_ESM.zip › Fig 5/Fig 5C/1.2_FER YCE+ROL23 YNE/Image007_ch01.tif]

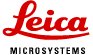

Supplement: Supplementary file 7 — Source data Fig. 5 [file 44318_2025_614_MOESM7_ESM.zip › Fig 5/Fig 5C/1.2_FER YCE+ROL23 YNE/MetaData/LeicaLogo.jpg]

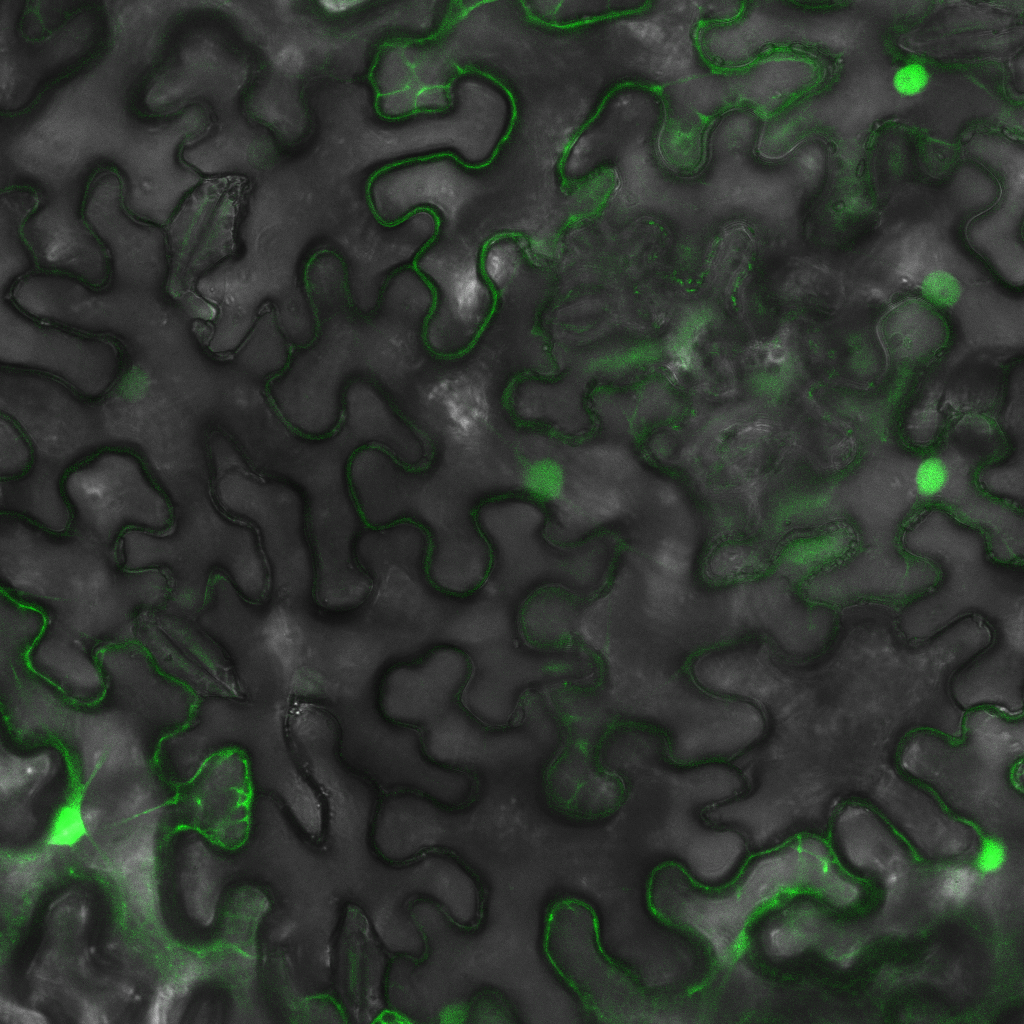

Supplement: Supplementary file 7 — Source data Fig. 5 [file 44318_2025_614_MOESM7_ESM.zip › Fig 5/Fig 5C/2.1_Iti6b YNE+ROL23 YCE/11.tif]

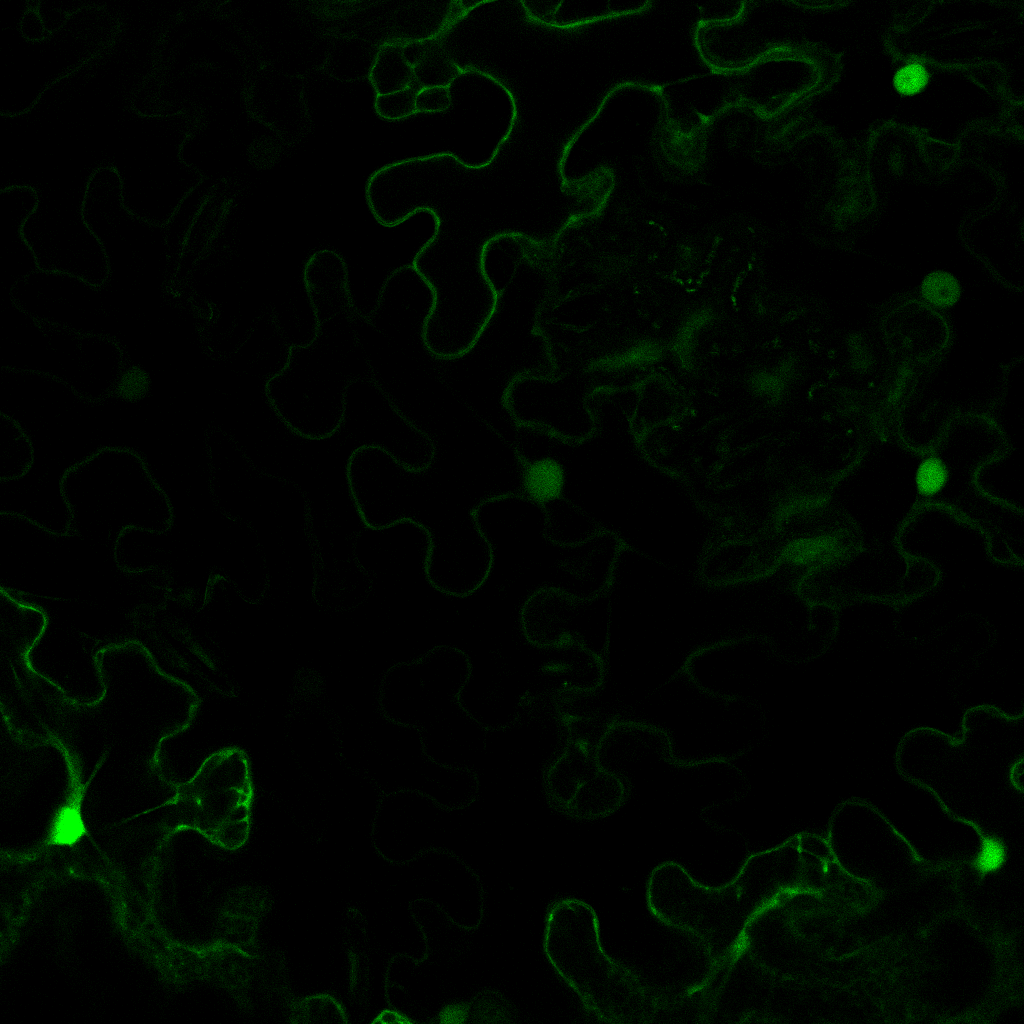

Supplement: Supplementary file 7 — Source data Fig. 5 [file 44318_2025_614_MOESM7_ESM.zip › Fig 5/Fig 5C/2.1_Iti6b YNE+ROL23 YCE/11_ch00.tif]

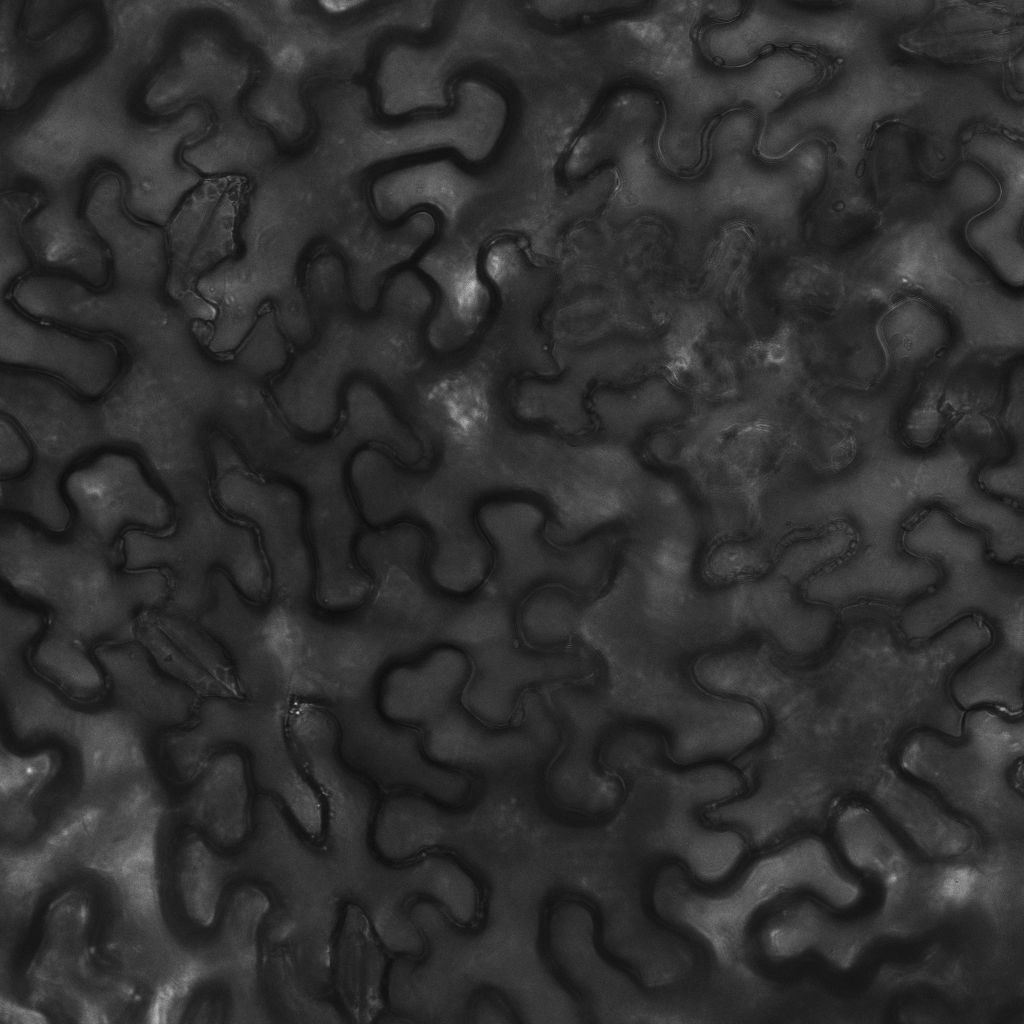

Supplement: Supplementary file 7 — Source data Fig. 5 [file 44318_2025_614_MOESM7_ESM.zip › Fig 5/Fig 5C/2.1_Iti6b YNE+ROL23 YCE/11_ch01.tif]

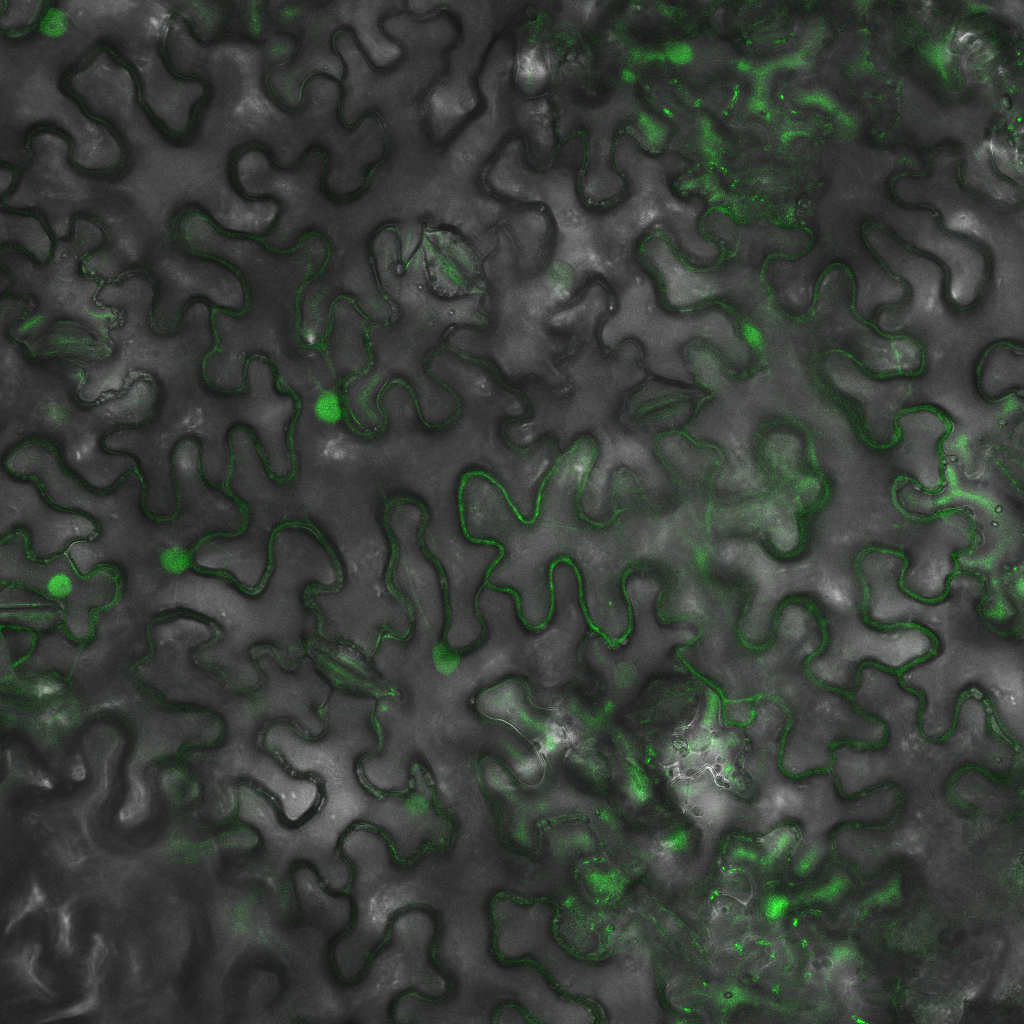

Supplement: Supplementary file 7 — Source data Fig. 5 [file 44318_2025_614_MOESM7_ESM.zip › Fig 5/Fig 5C/2.1_Iti6b YNE+ROL23 YCE/7.tif]

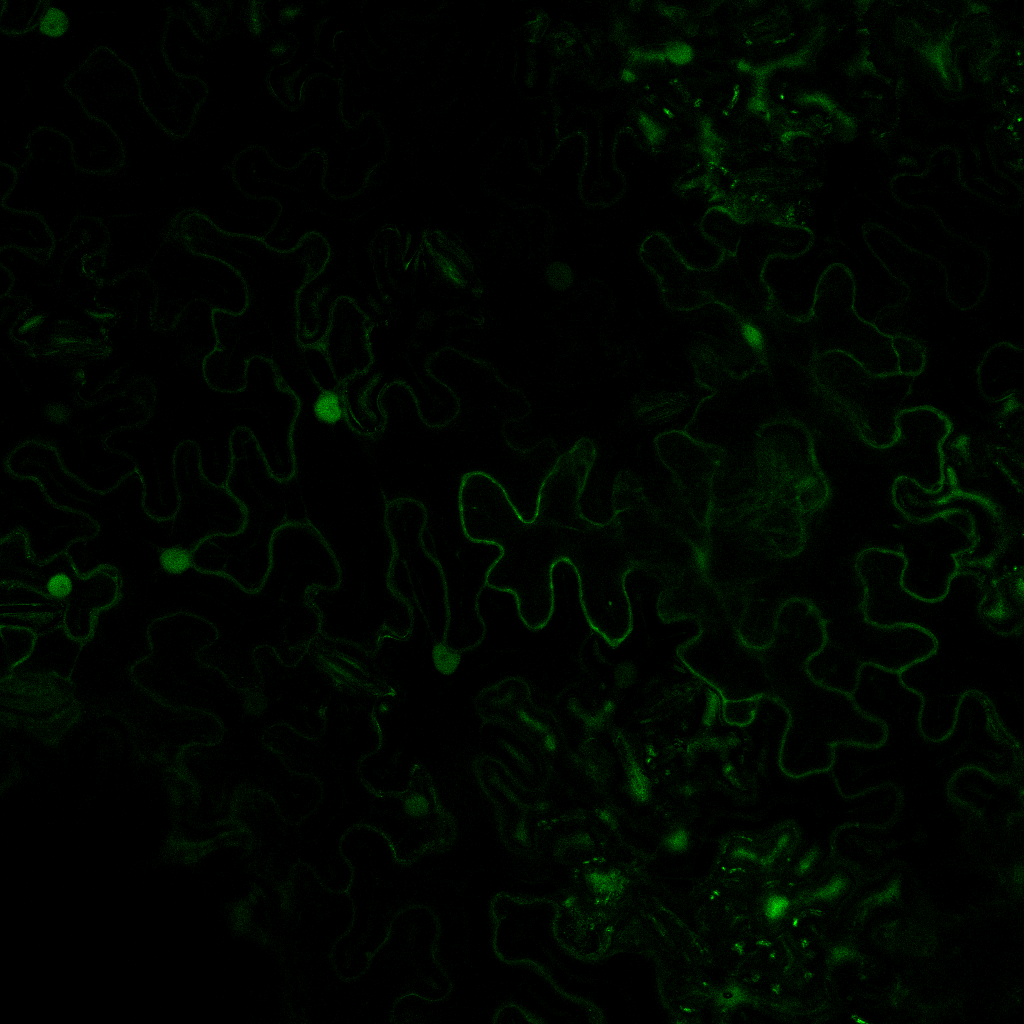

Supplement: Supplementary file 7 — Source data Fig. 5 [file 44318_2025_614_MOESM7_ESM.zip › Fig 5/Fig 5C/2.1_Iti6b YNE+ROL23 YCE/7_ch00.tif]

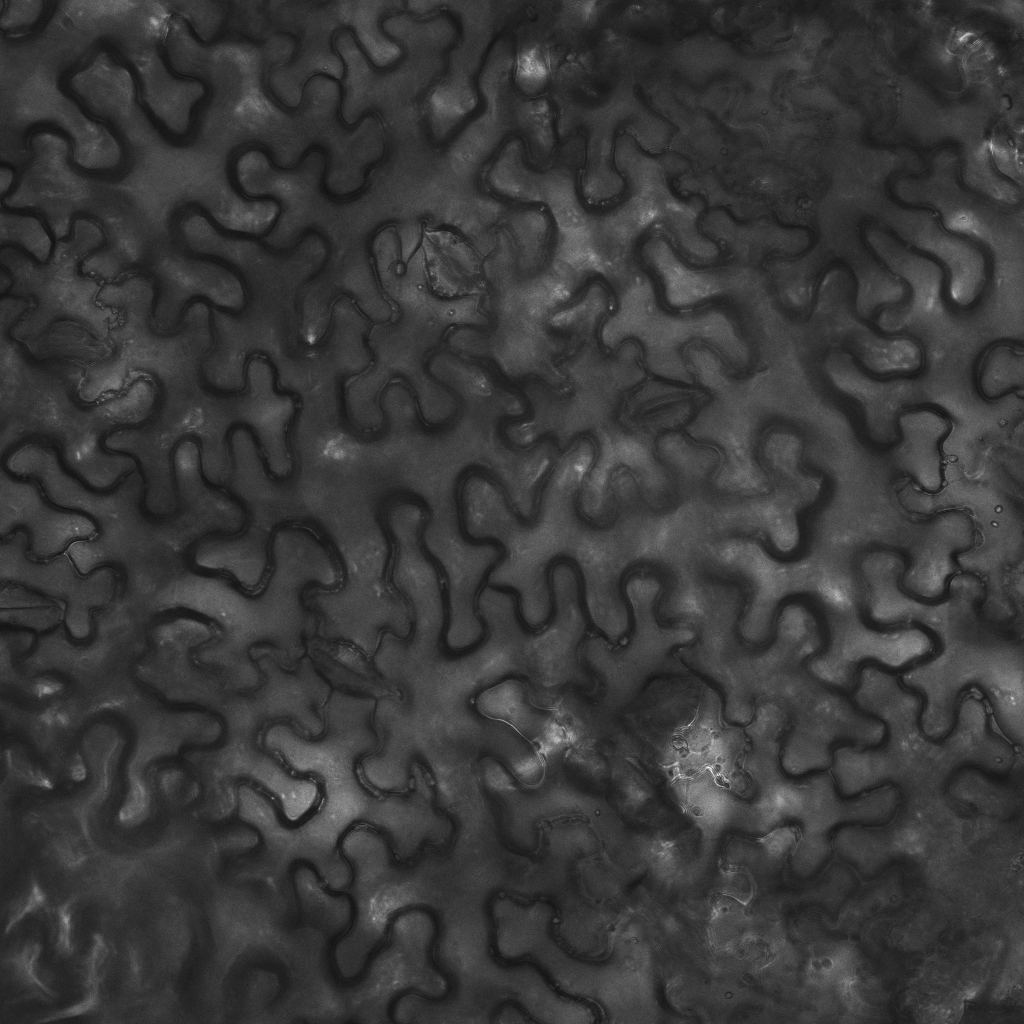

Supplement: Supplementary file 7 — Source data Fig. 5 [file 44318_2025_614_MOESM7_ESM.zip › Fig 5/Fig 5C/2.1_Iti6b YNE+ROL23 YCE/7_ch01.tif]

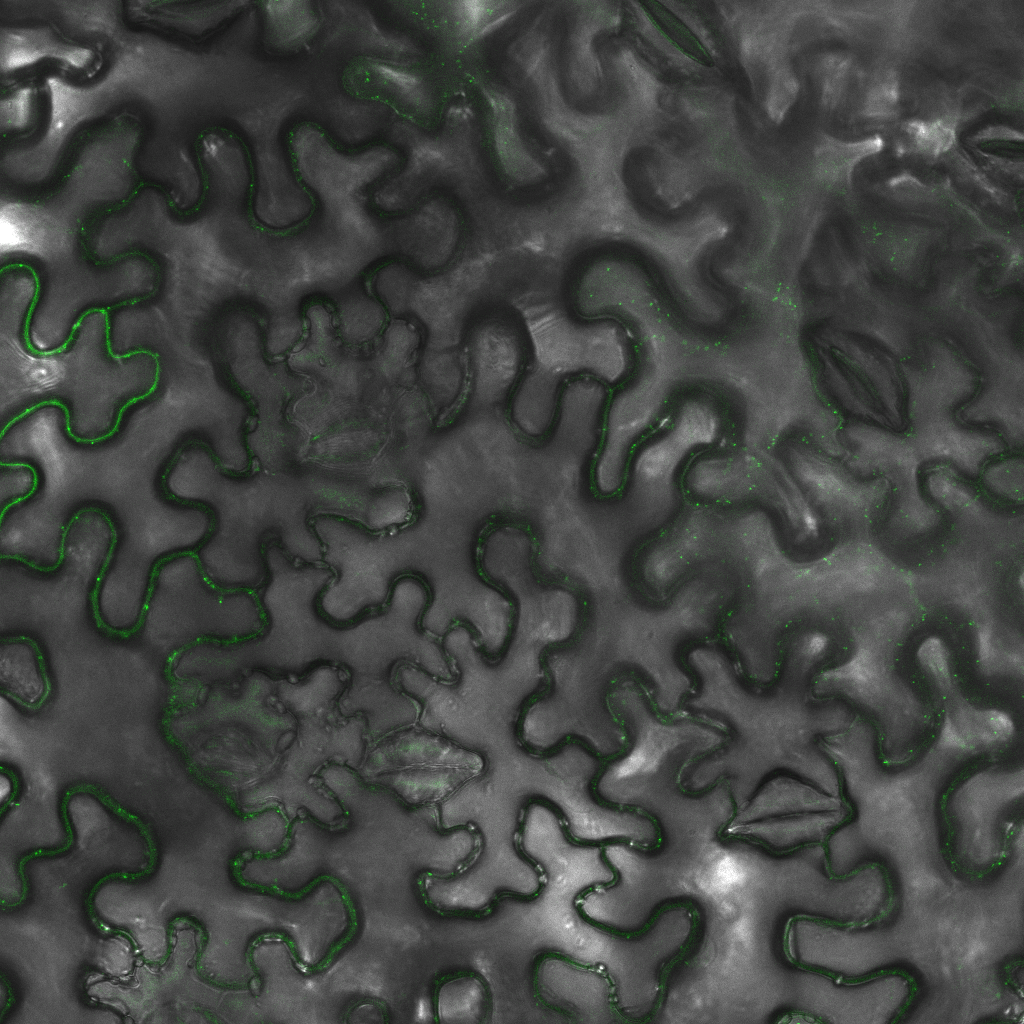

Supplement: Supplementary file 7 — Source data Fig. 5 [file 44318_2025_614_MOESM7_ESM.zip › Fig 5/Fig 5C/3.2_Iti6b YCE _ROL23 YNE/Figure3/Image002_000.tif]

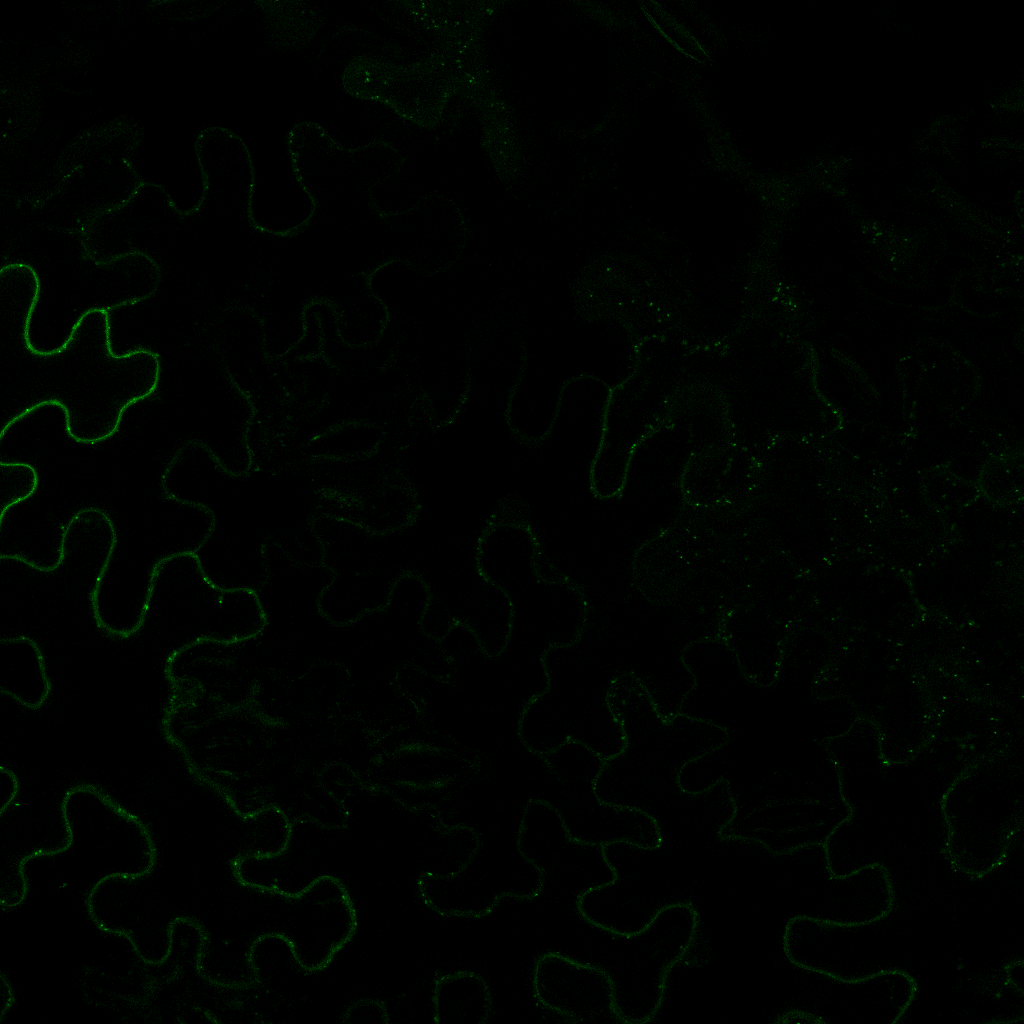

Supplement: Supplementary file 7 — Source data Fig. 5 [file 44318_2025_614_MOESM7_ESM.zip › Fig 5/Fig 5C/3.2_Iti6b YCE _ROL23 YNE/Figure3/Image002_000_ch00.tif]

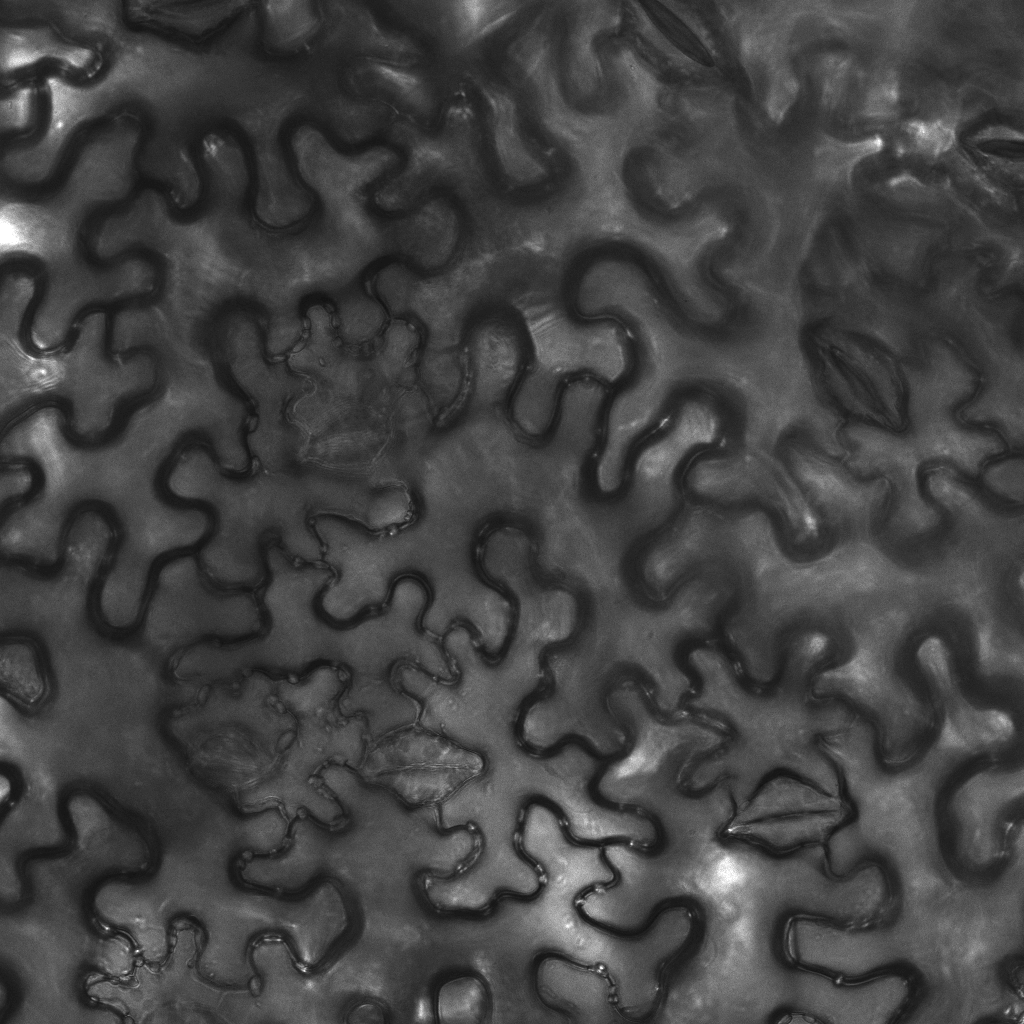

Supplement: Supplementary file 7 — Source data Fig. 5 [file 44318_2025_614_MOESM7_ESM.zip › Fig 5/Fig 5C/3.2_Iti6b YCE _ROL23 YNE/Figure3/Image002_000_ch01.tif]

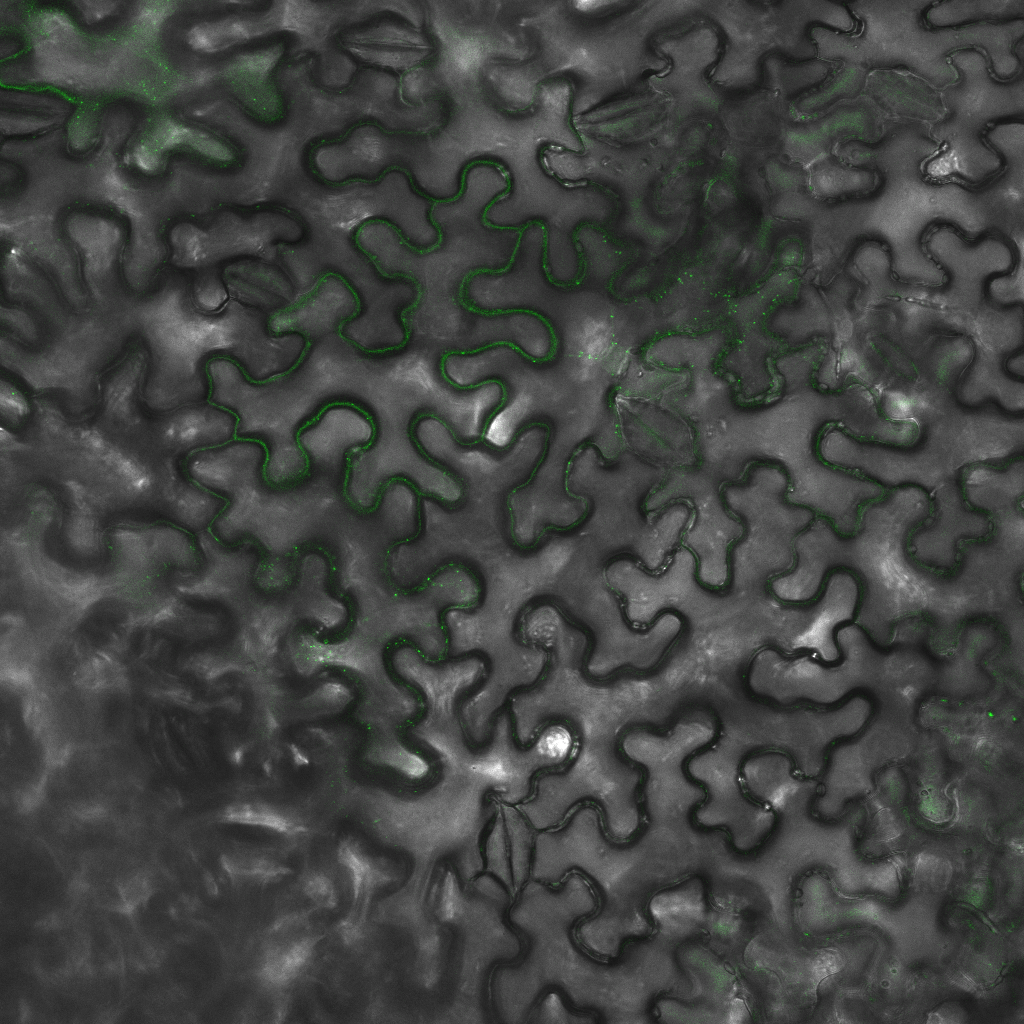

Supplement: Supplementary file 7 — Source data Fig. 5 [file 44318_2025_614_MOESM7_ESM.zip › Fig 5/Fig 5C/3.2_Iti6b YCE _ROL23 YNE/Image001.tif]

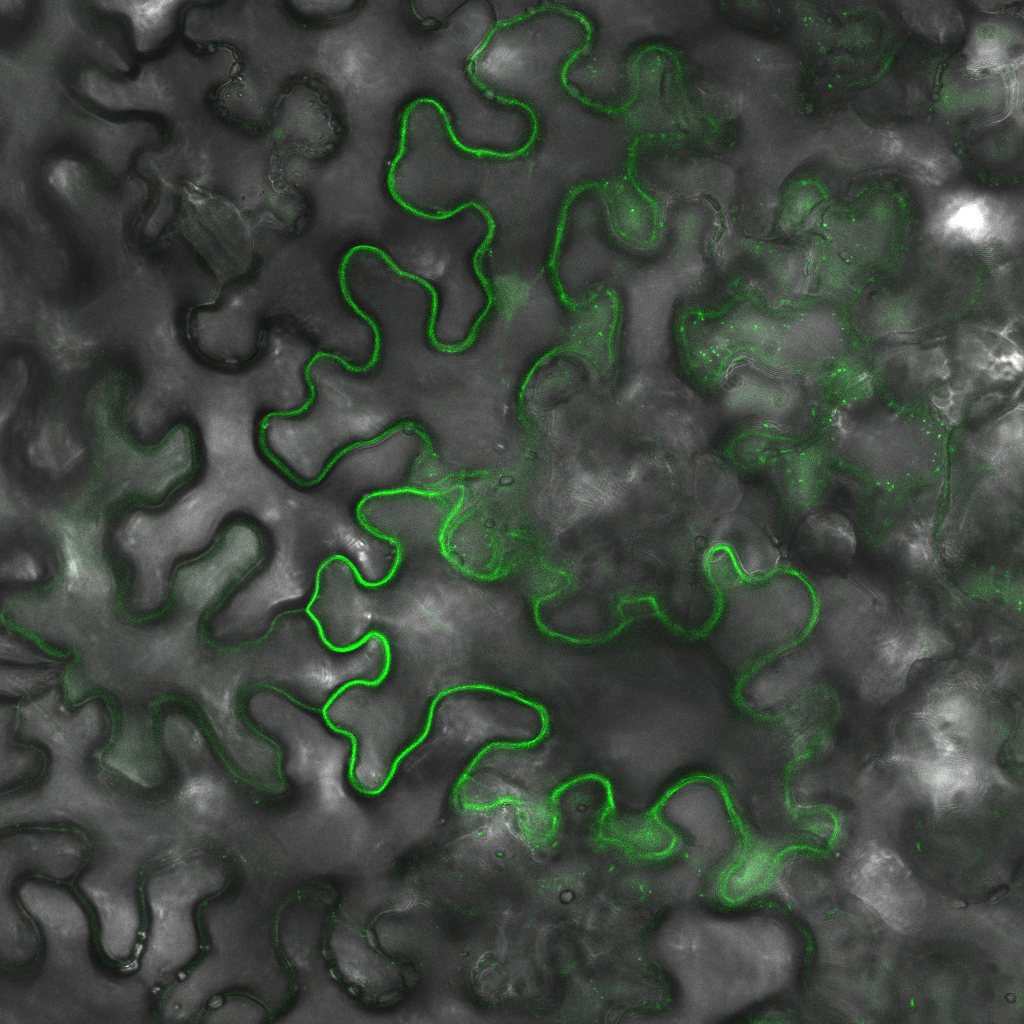

Supplement: Supplementary file 7 — Source data Fig. 5 [file 44318_2025_614_MOESM7_ESM.zip › Fig 5/Fig 5C/3.2_Iti6b YCE _ROL23 YNE/Image001_000.tif]

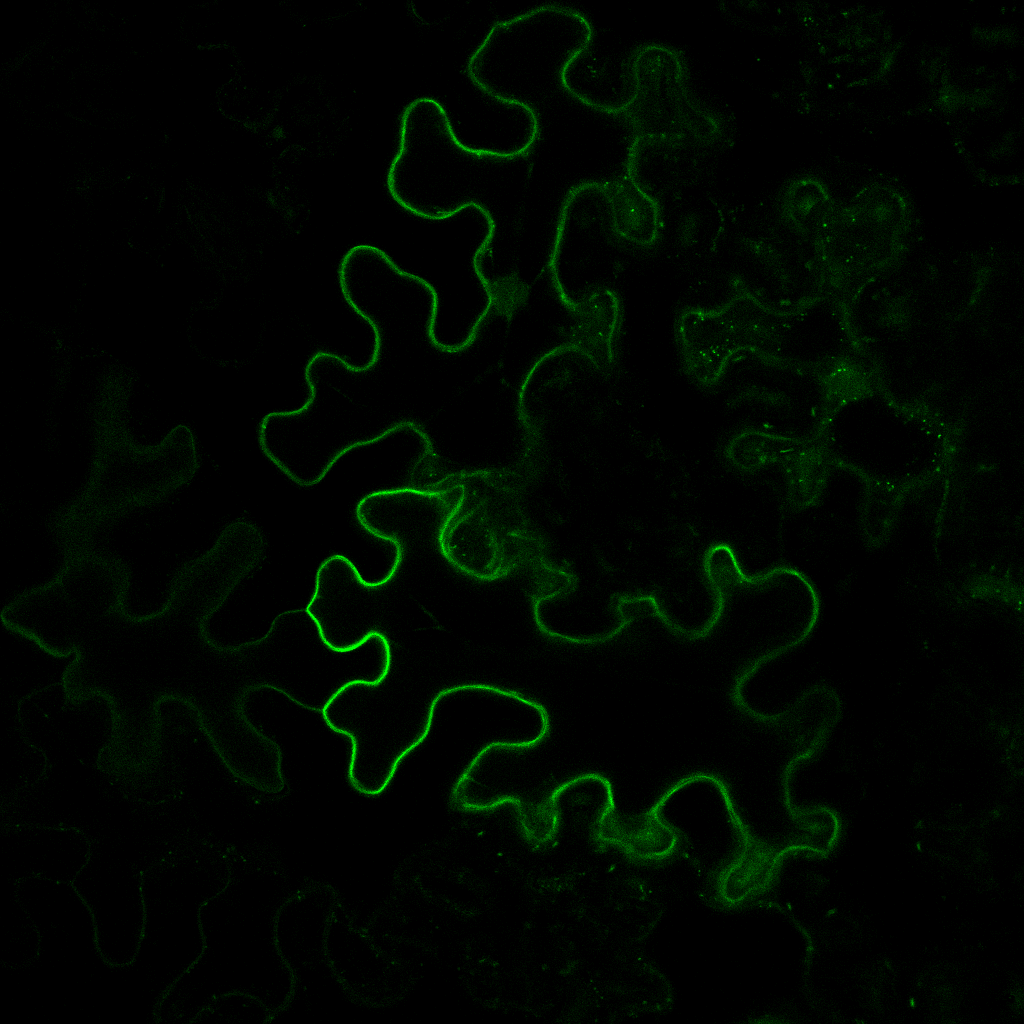

Supplement: Supplementary file 7 — Source data Fig. 5 [file 44318_2025_614_MOESM7_ESM.zip › Fig 5/Fig 5C/3.2_Iti6b YCE _ROL23 YNE/Image001_000_ch00.tif]

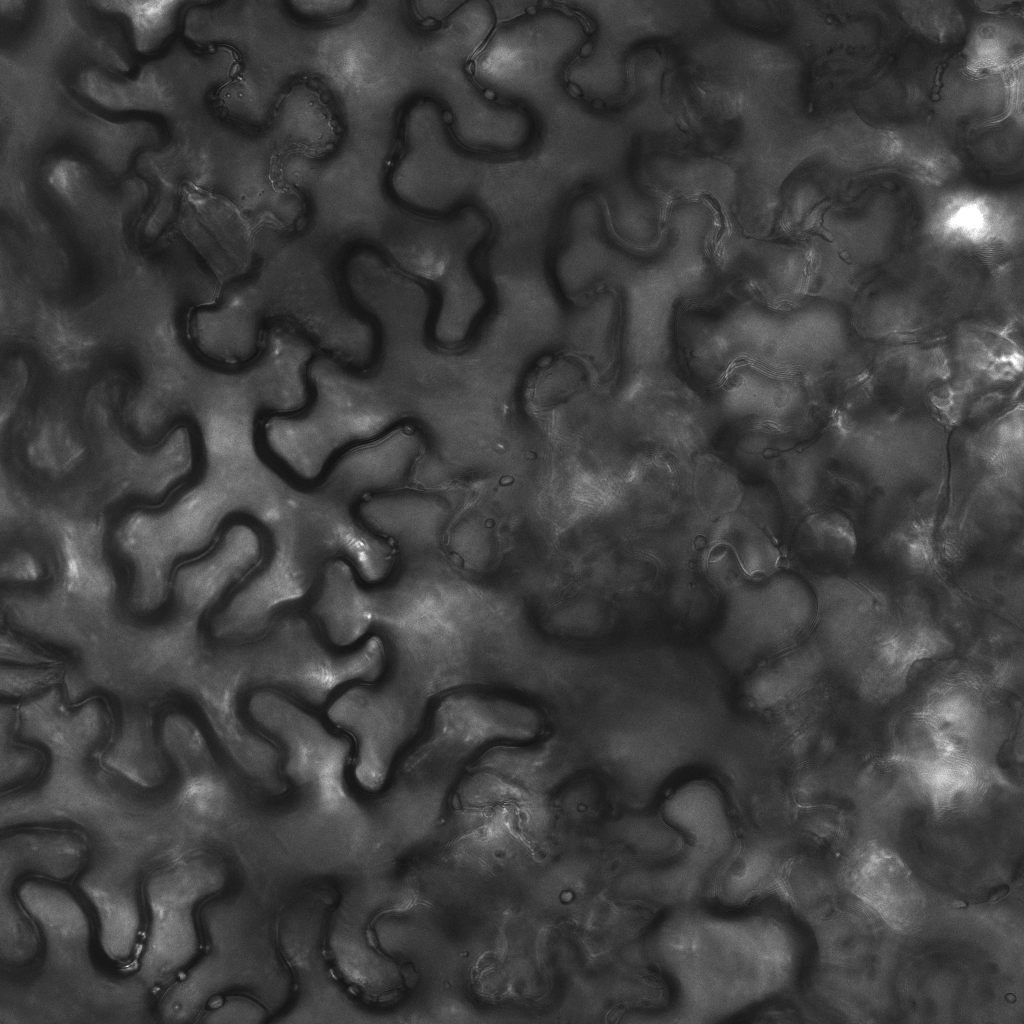

Supplement: Supplementary file 7 — Source data Fig. 5 [file 44318_2025_614_MOESM7_ESM.zip › Fig 5/Fig 5C/3.2_Iti6b YCE _ROL23 YNE/Image001_000_ch01.tif]

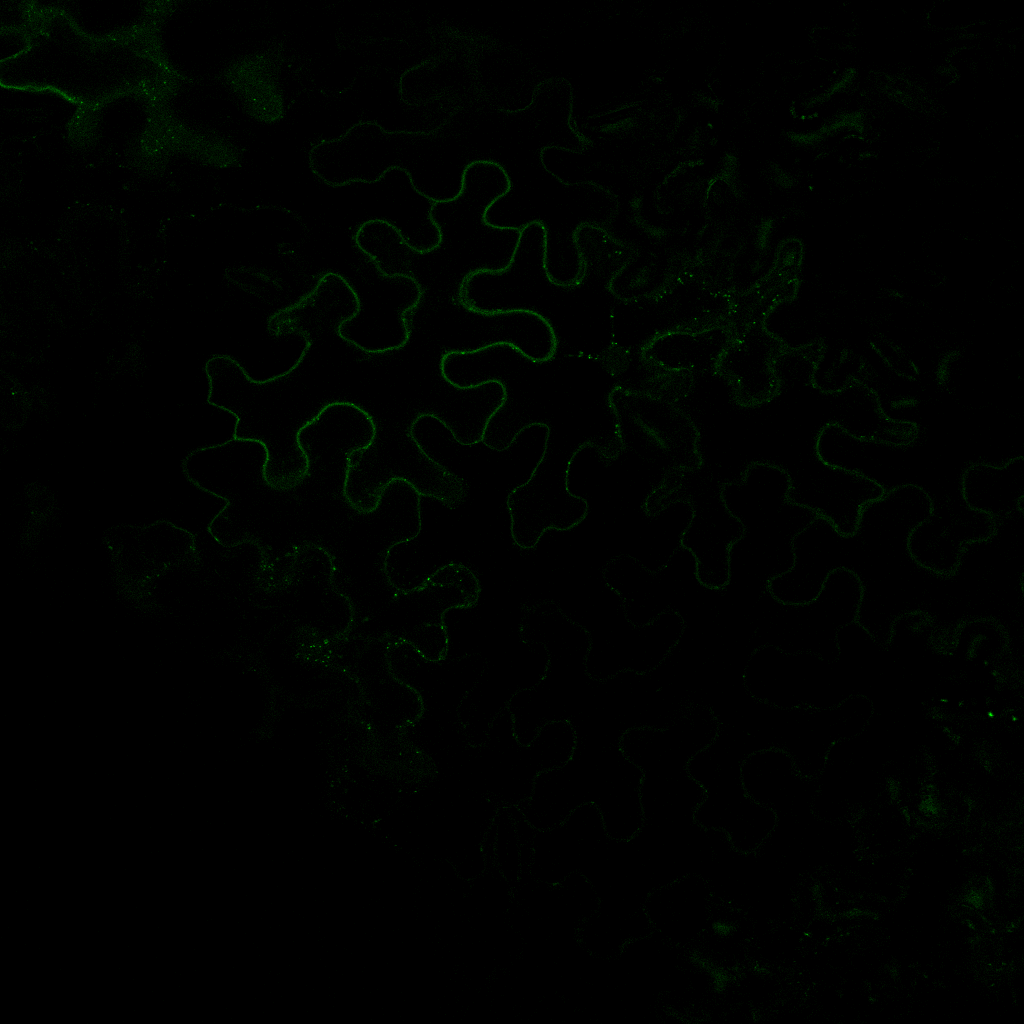

Supplement: Supplementary file 7 — Source data Fig. 5 [file 44318_2025_614_MOESM7_ESM.zip › Fig 5/Fig 5C/3.2_Iti6b YCE _ROL23 YNE/Image001_ch00.tif]

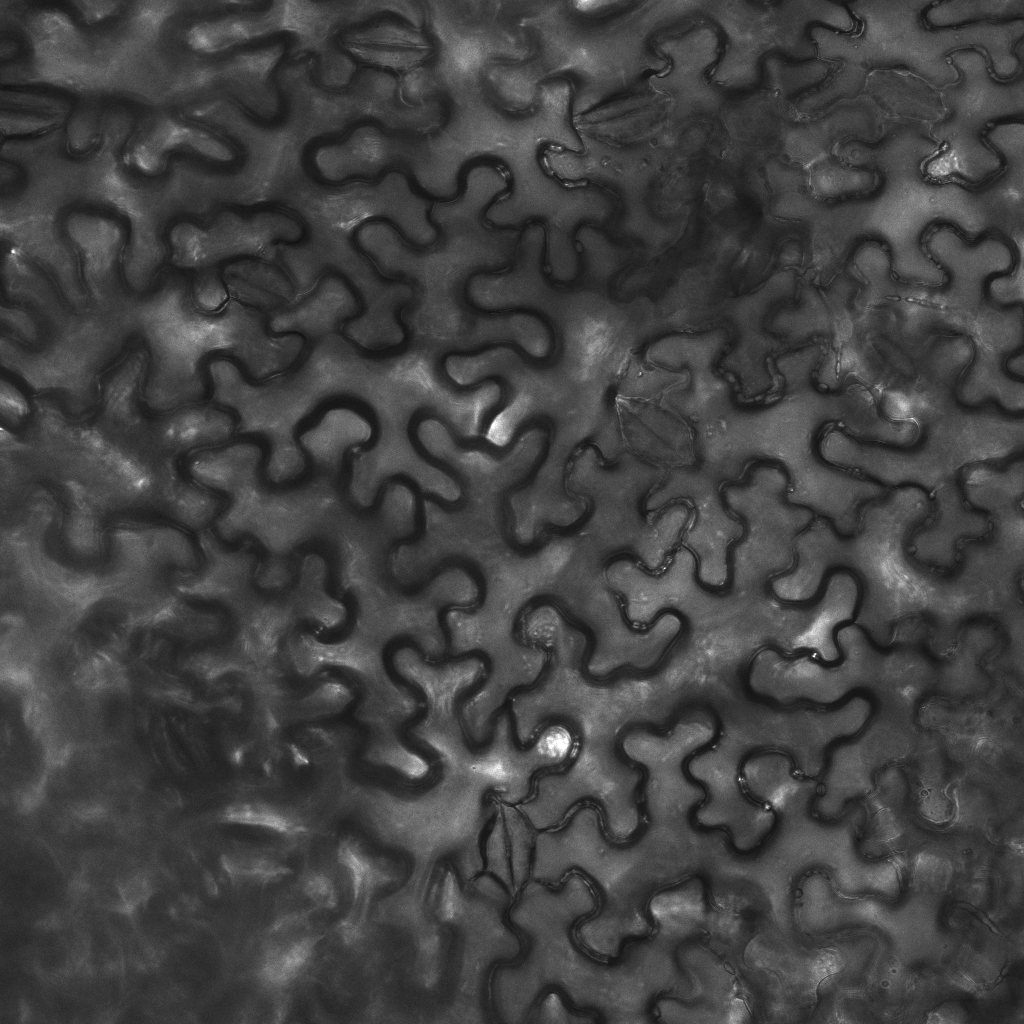

Supplement: Supplementary file 7 — Source data Fig. 5 [file 44318_2025_614_MOESM7_ESM.zip › Fig 5/Fig 5C/3.2_Iti6b YCE _ROL23 YNE/Image001_ch01.tif]

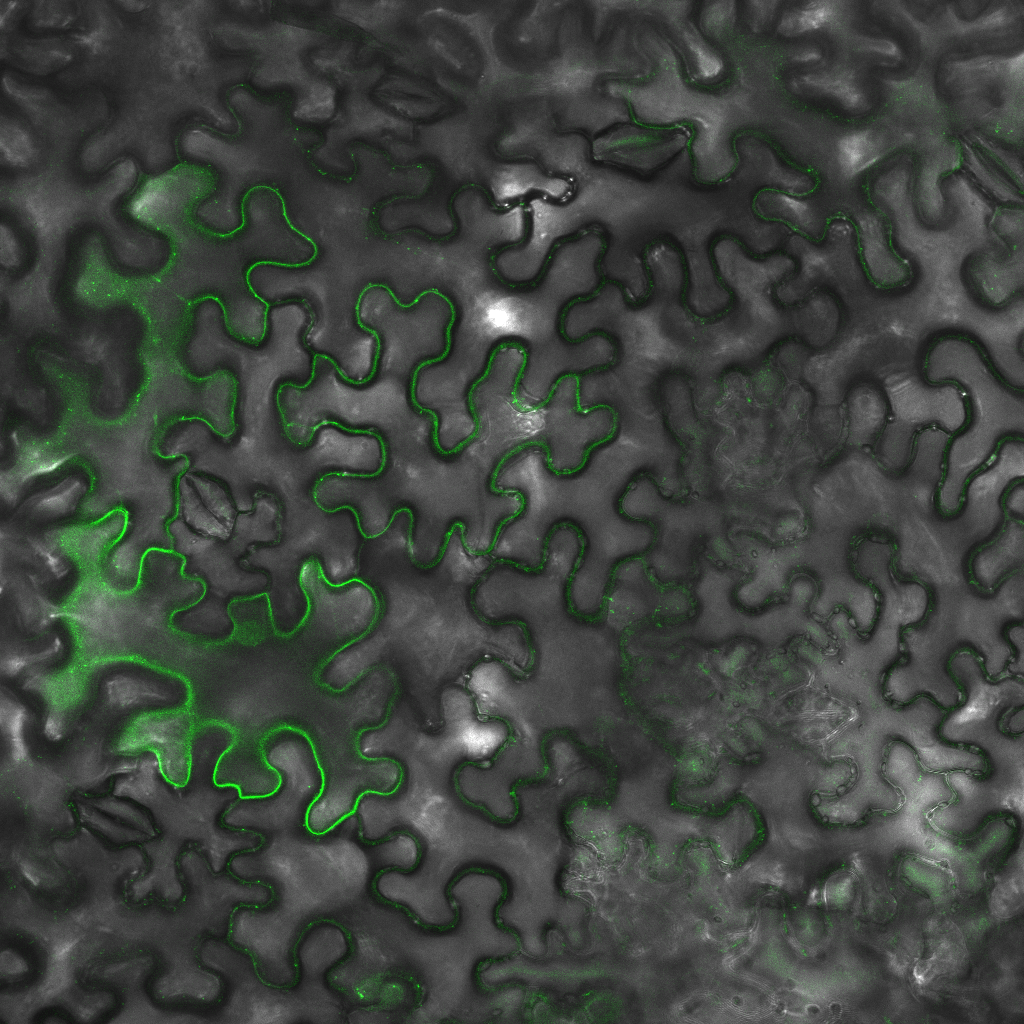

Supplement: Supplementary file 7 — Source data Fig. 5 [file 44318_2025_614_MOESM7_ESM.zip › Fig 5/Fig 5C/3.2_Iti6b YCE _ROL23 YNE/Image003.tif]

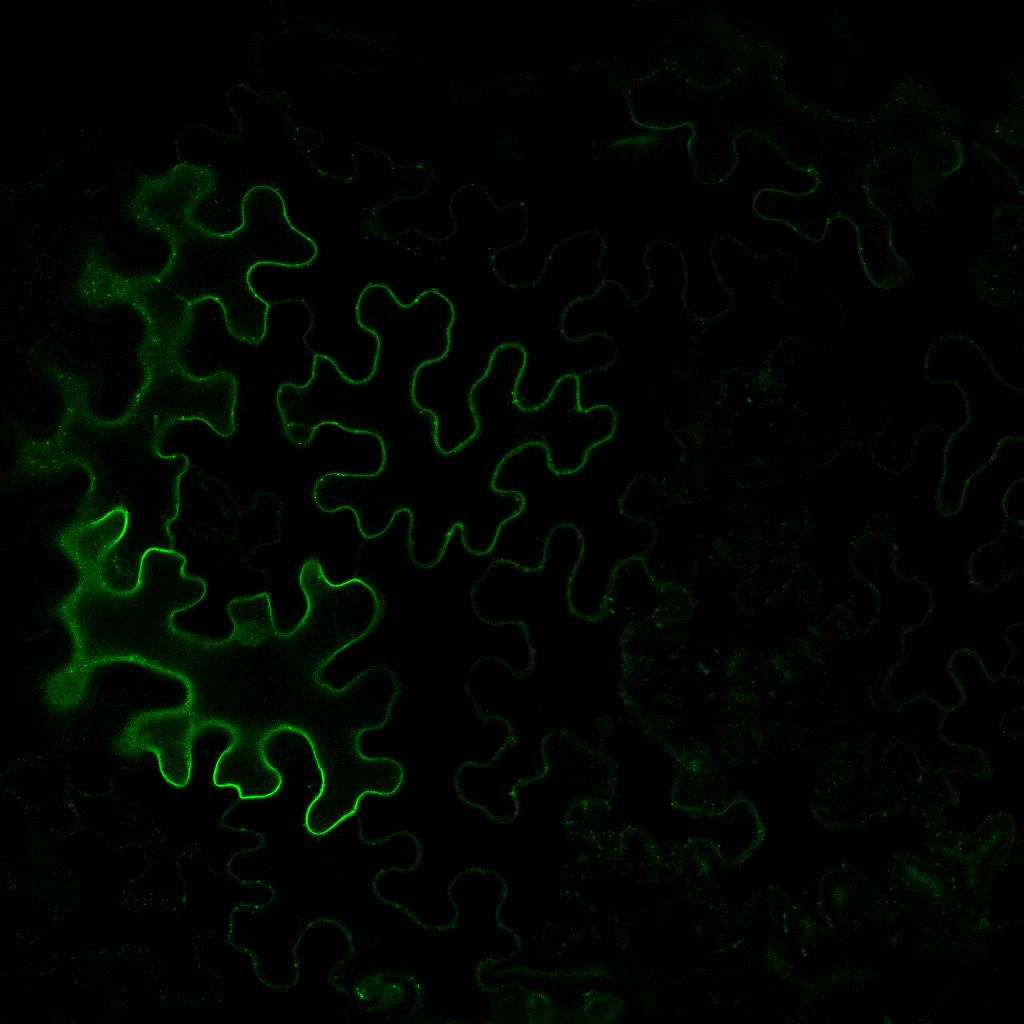

Supplement: Supplementary file 7 — Source data Fig. 5 [file 44318_2025_614_MOESM7_ESM.zip › Fig 5/Fig 5C/3.2_Iti6b YCE _ROL23 YNE/Image003_ch00.tif]

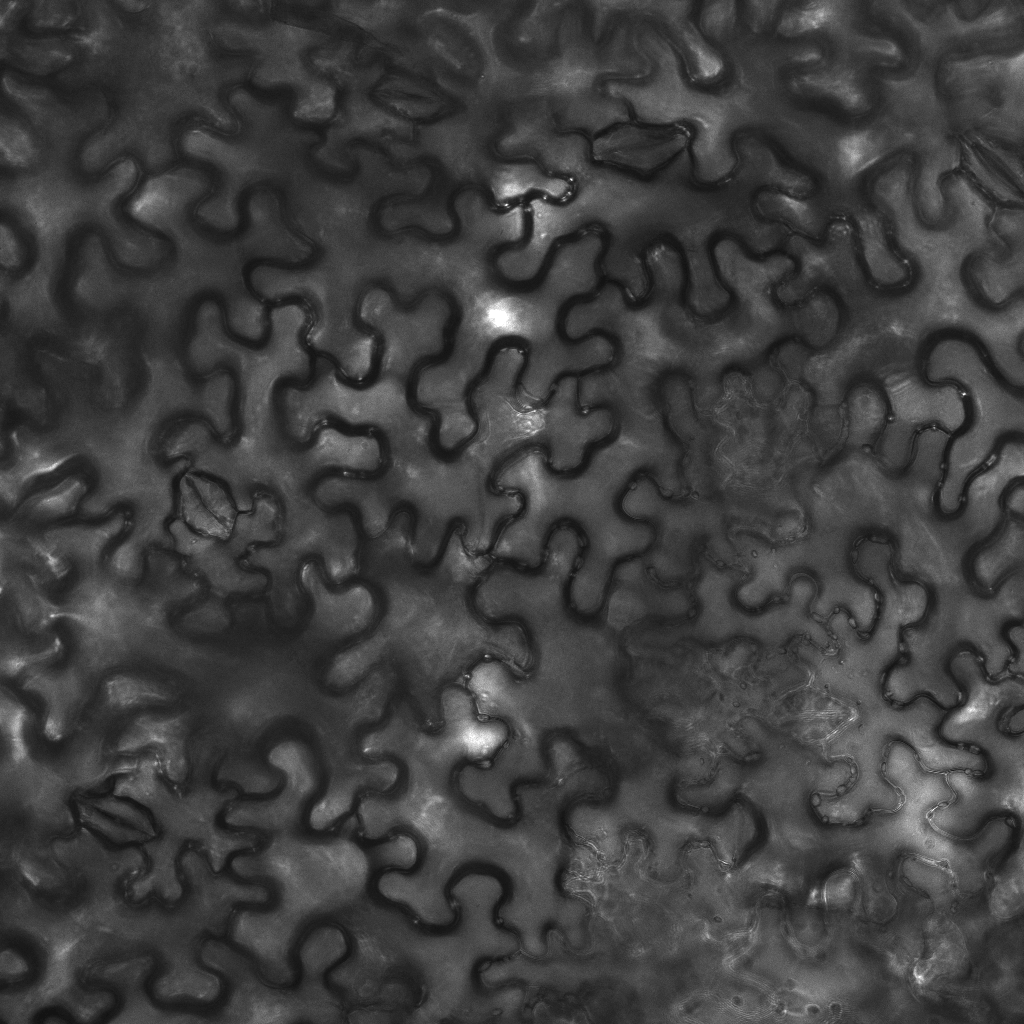

Supplement: Supplementary file 7 — Source data Fig. 5 [file 44318_2025_614_MOESM7_ESM.zip › Fig 5/Fig 5C/3.2_Iti6b YCE _ROL23 YNE/Image003_ch01.tif]

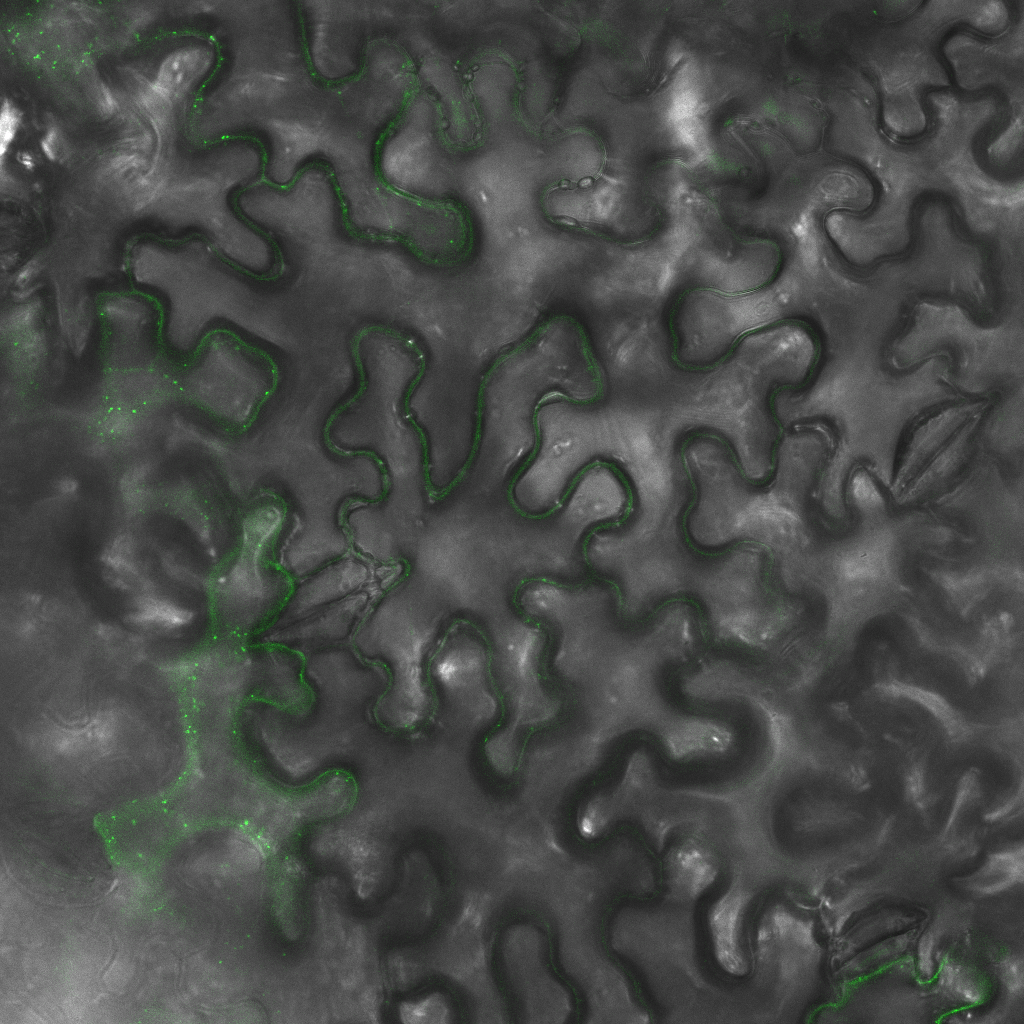

Supplement: Supplementary file 7 — Source data Fig. 5 [file 44318_2025_614_MOESM7_ESM.zip › Fig 5/Fig 5C/3.2_Iti6b YCE _ROL23 YNE/Image005.tif]

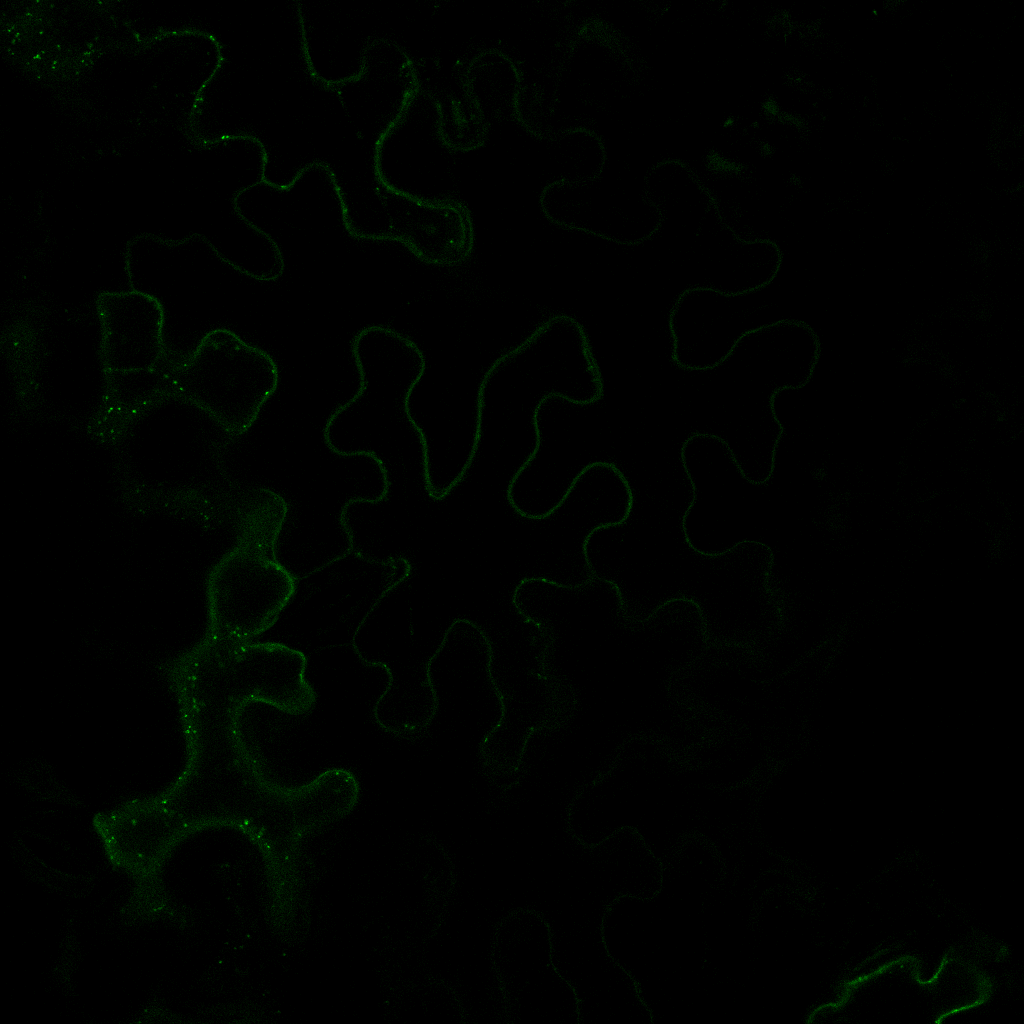

Supplement: Supplementary file 7 — Source data Fig. 5 [file 44318_2025_614_MOESM7_ESM.zip › Fig 5/Fig 5C/3.2_Iti6b YCE _ROL23 YNE/Image005_ch00.tif]

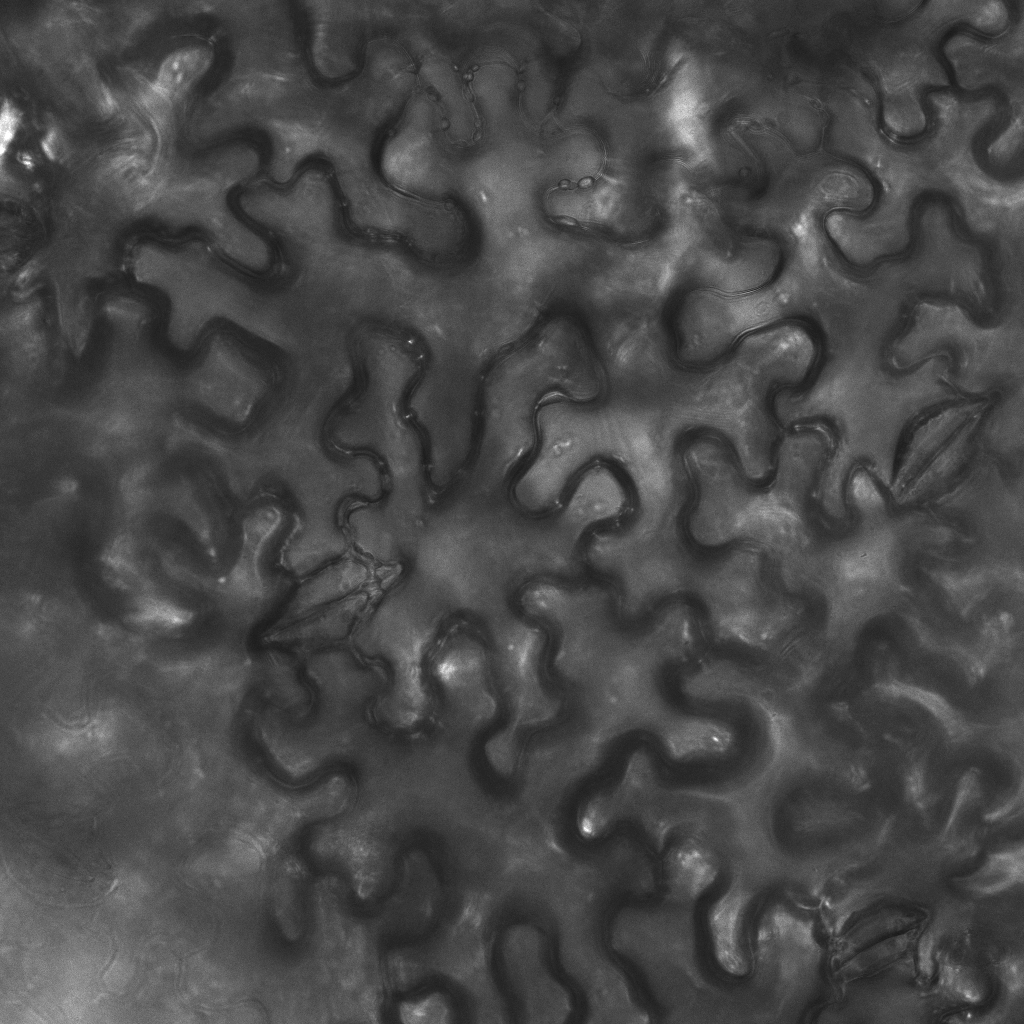

Supplement: Supplementary file 7 — Source data Fig. 5 [file 44318_2025_614_MOESM7_ESM.zip › Fig 5/Fig 5C/3.2_Iti6b YCE _ROL23 YNE/Image005_ch01.tif]

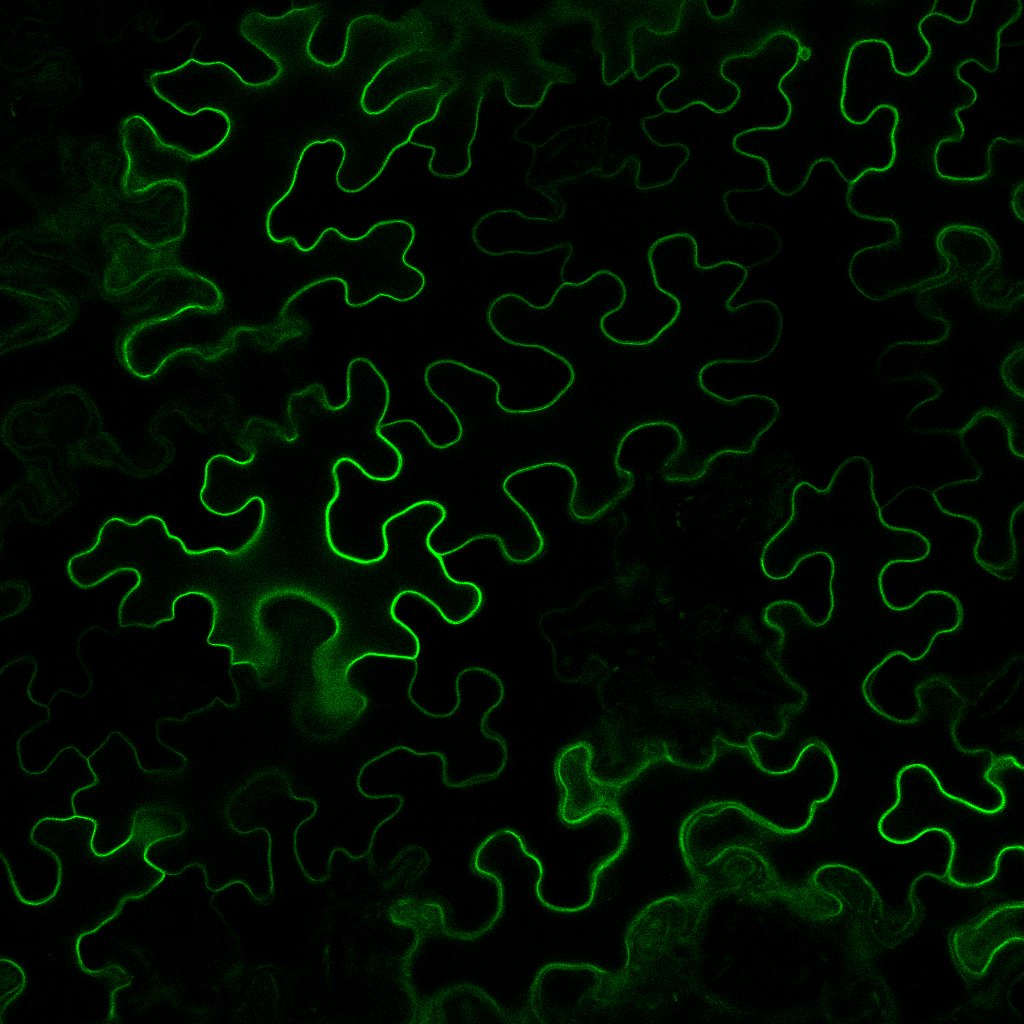

Supplement: Supplementary file 7 — Source data Fig. 5 [file 44318_2025_614_MOESM7_ESM.zip › Fig 5/Fig 5C/6.1_FER YNE +ROL23 YCE/12_ch00.tif]

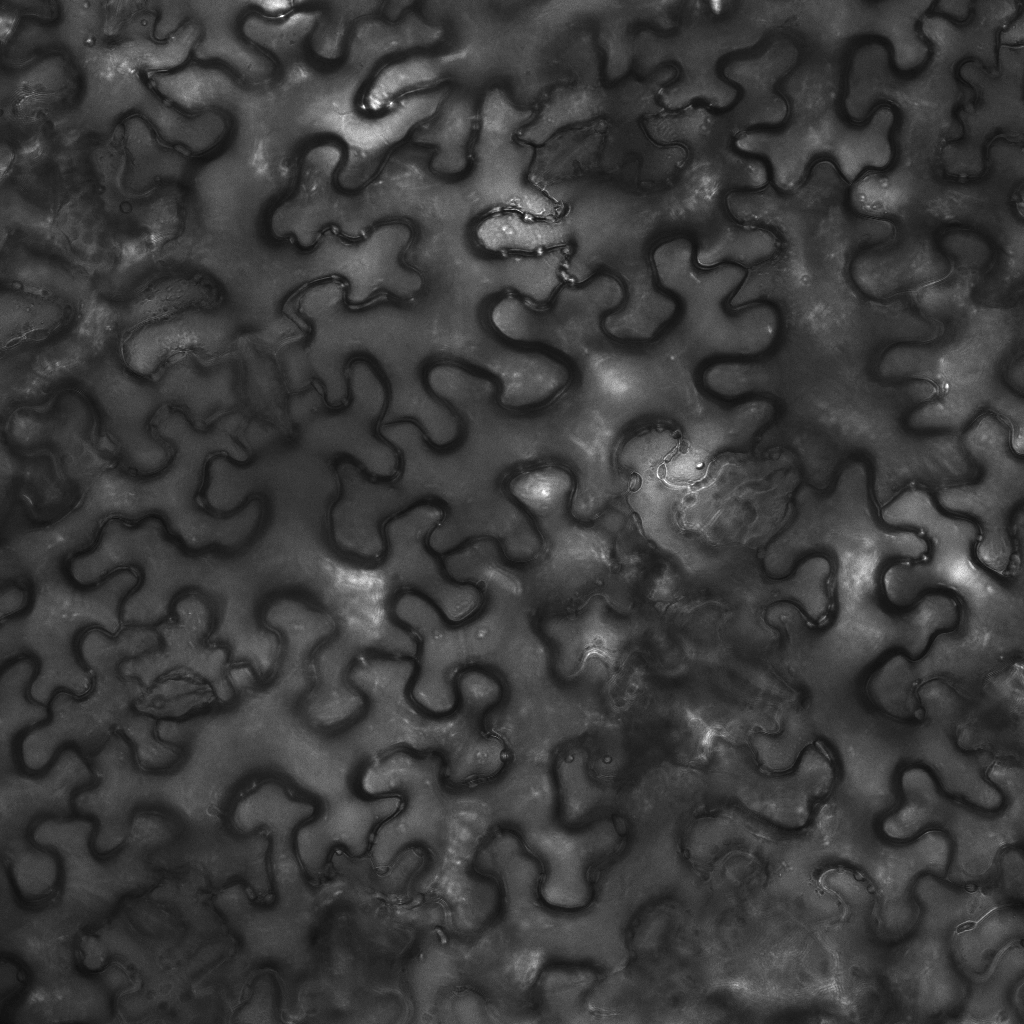

Supplement: Supplementary file 7 — Source data Fig. 5 [file 44318_2025_614_MOESM7_ESM.zip › Fig 5/Fig 5C/6.1_FER YNE +ROL23 YCE/12_ch01.tif]

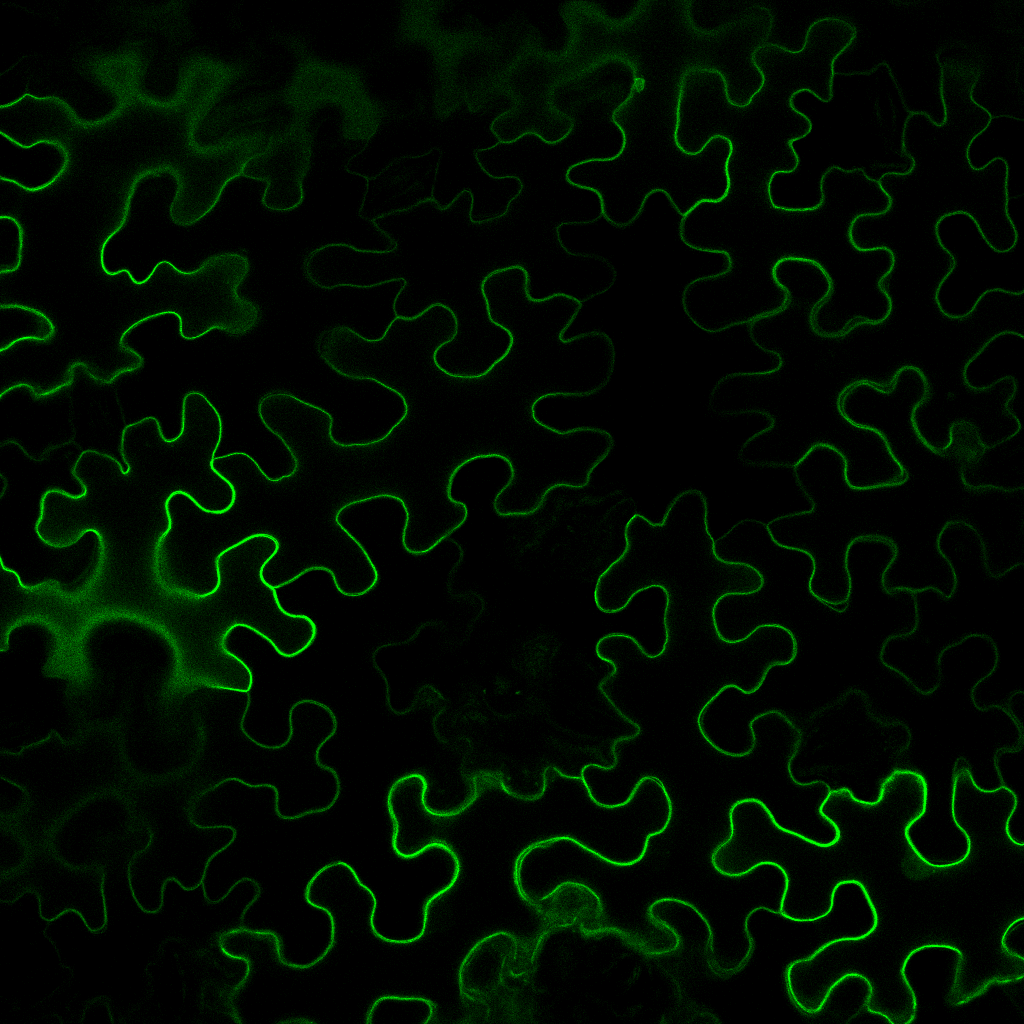

Supplement: Supplementary file 7 — Source data Fig. 5 [file 44318_2025_614_MOESM7_ESM.zip › Fig 5/Fig 5C/6.1_FER YNE +ROL23 YCE/13_ch00.tif]

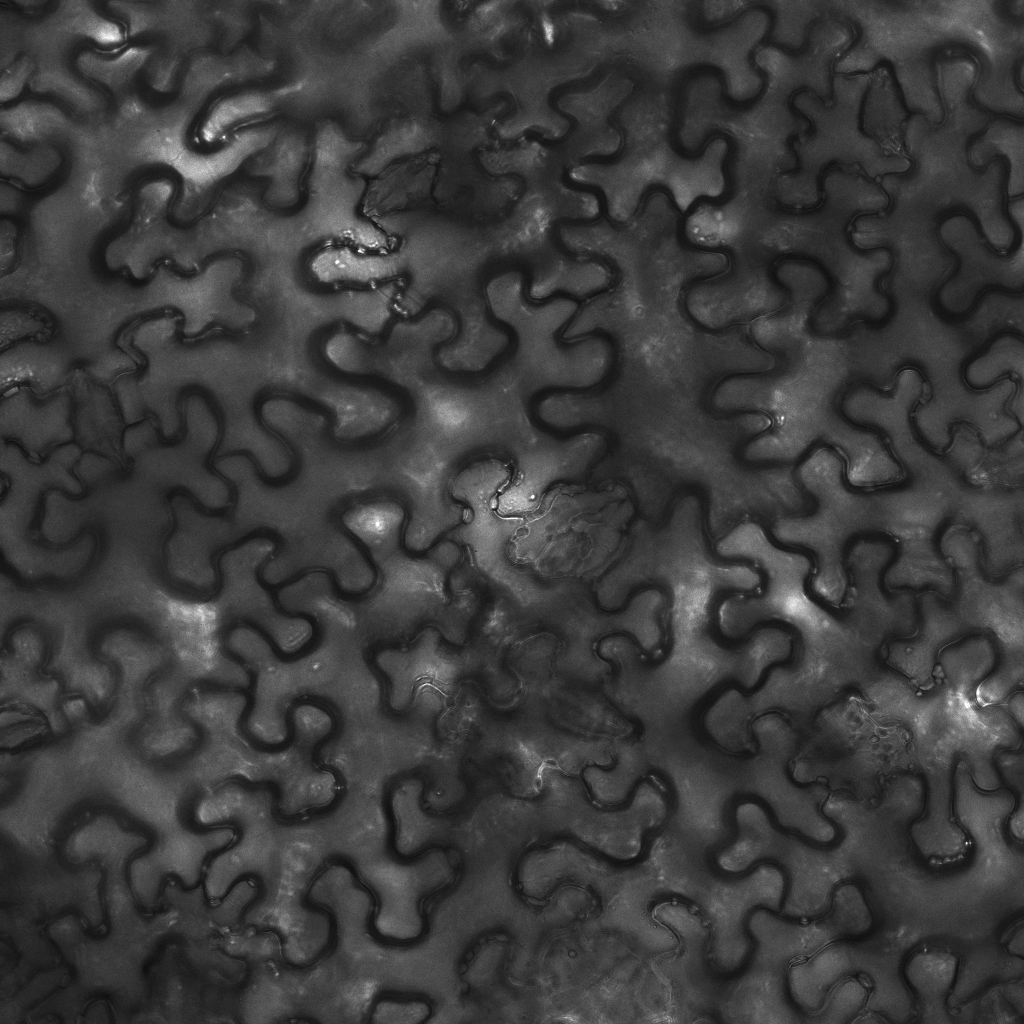

Supplement: Supplementary file 7 — Source data Fig. 5 [file 44318_2025_614_MOESM7_ESM.zip › Fig 5/Fig 5C/6.1_FER YNE +ROL23 YCE/13_ch01.tif]

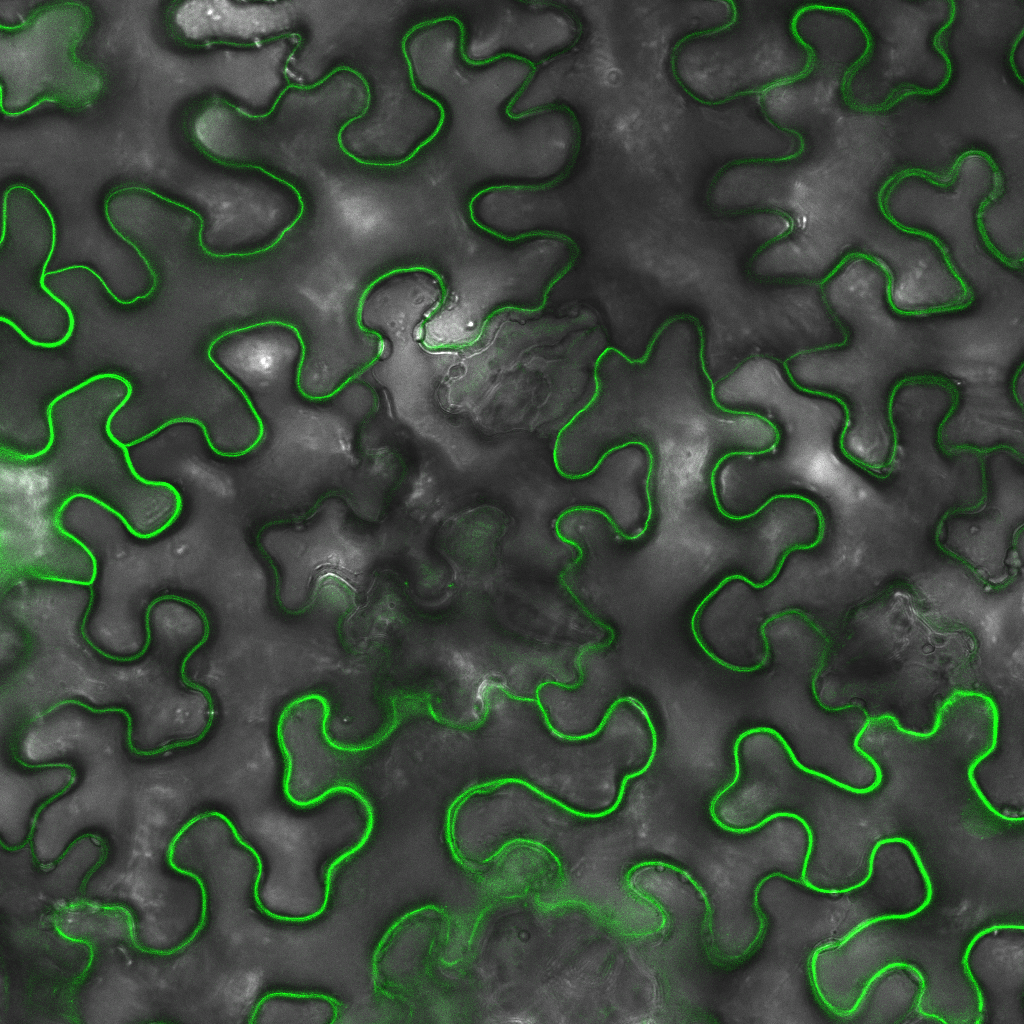

Supplement: Supplementary file 7 — Source data Fig. 5 [file 44318_2025_614_MOESM7_ESM.zip › Fig 5/Fig 5C/6.1_FER YNE +ROL23 YCE/14.tif]

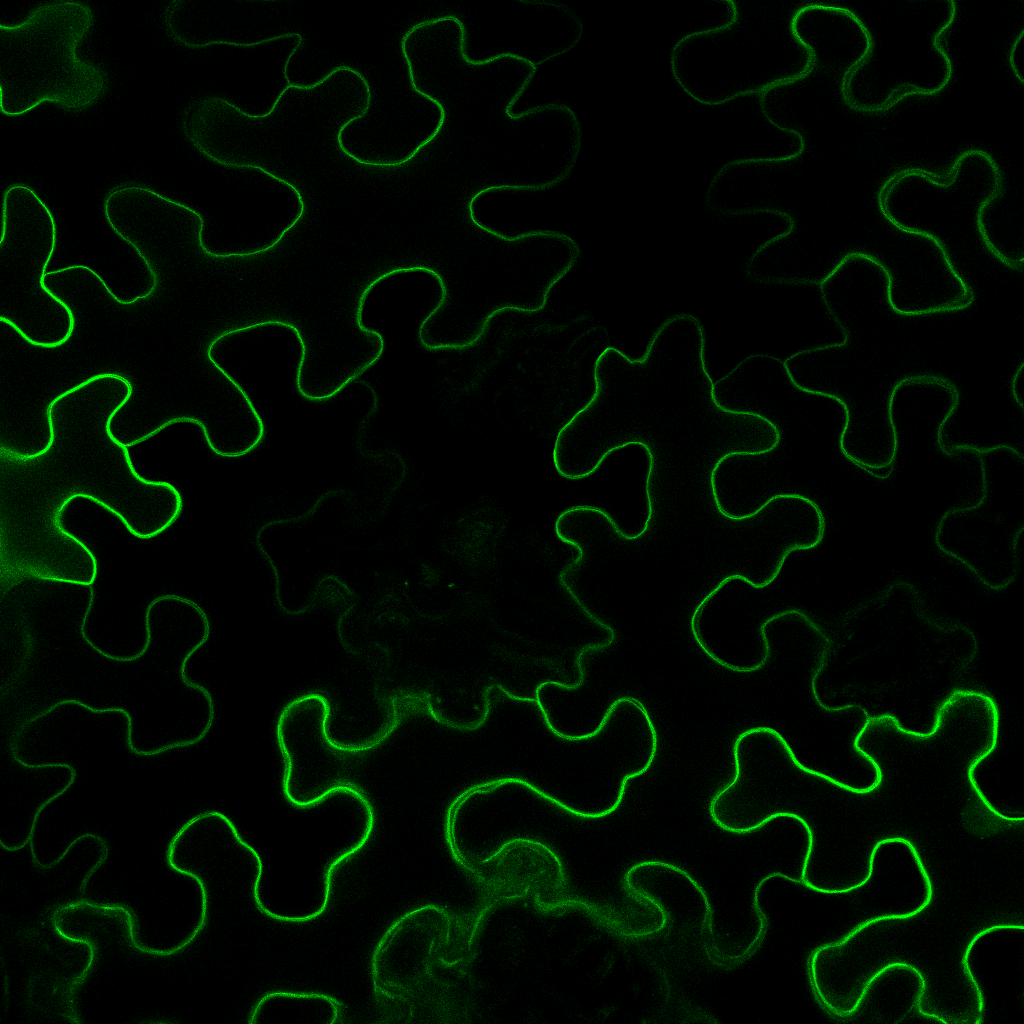

Supplement: Supplementary file 7 — Source data Fig. 5 [file 44318_2025_614_MOESM7_ESM.zip › Fig 5/Fig 5C/6.1_FER YNE +ROL23 YCE/14_ch00.tif]

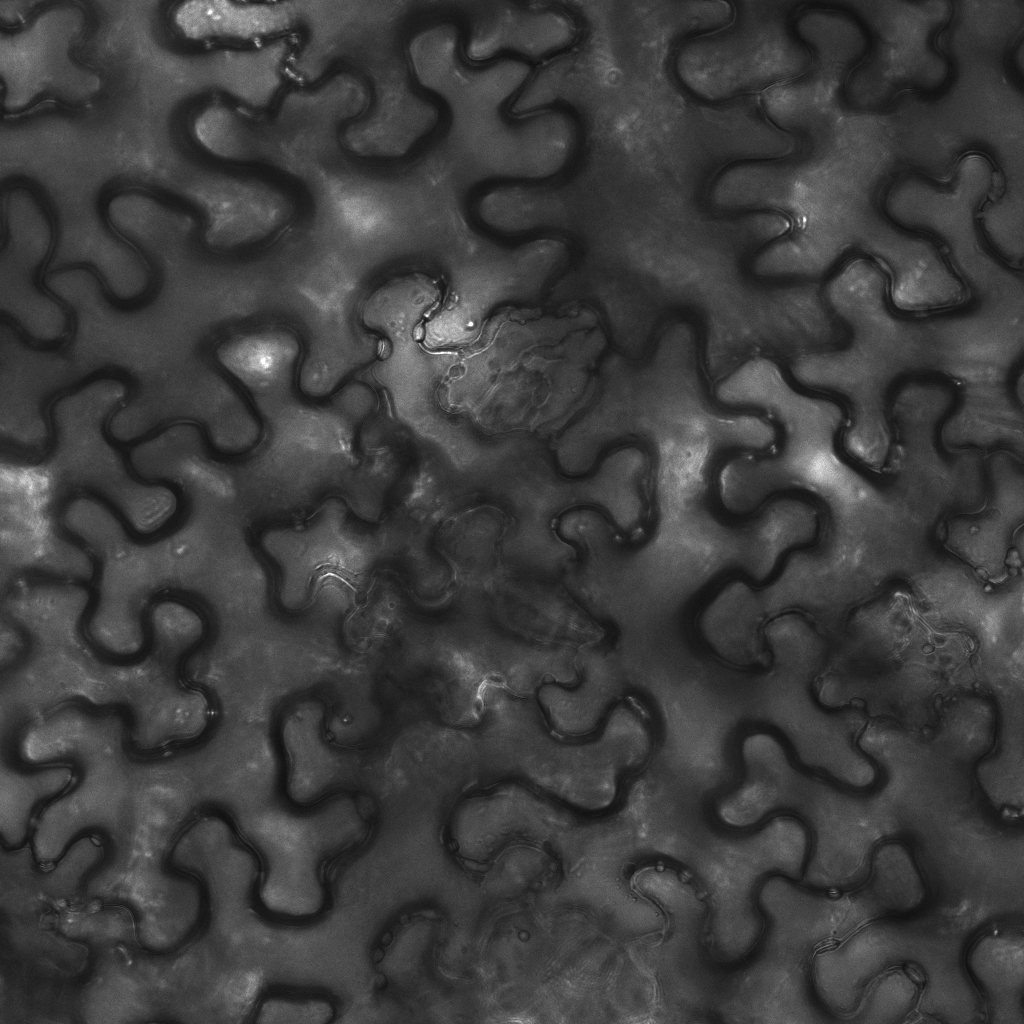

Supplement: Supplementary file 7 — Source data Fig. 5 [file 44318_2025_614_MOESM7_ESM.zip › Fig 5/Fig 5C/6.1_FER YNE +ROL23 YCE/14_ch01.tif]

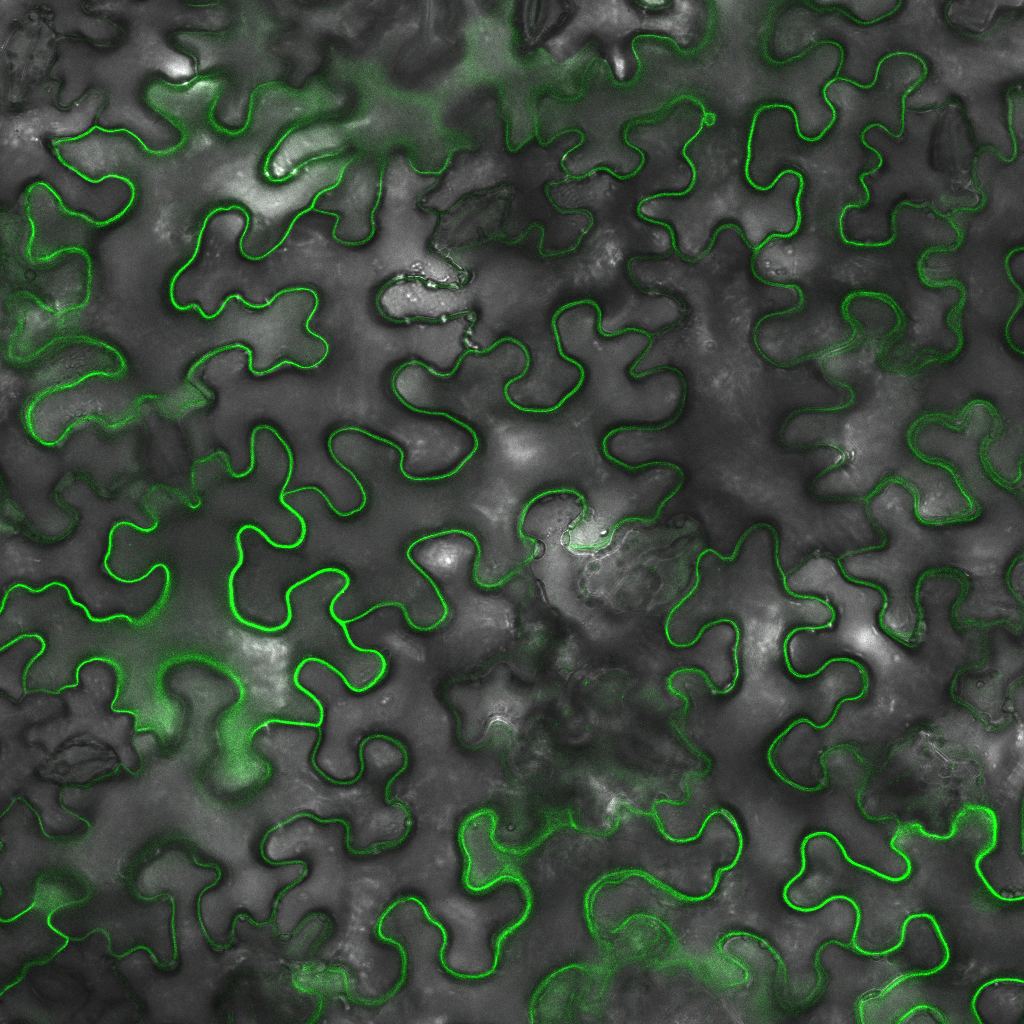

Supplement: Supplementary file 7 — Source data Fig. 5 [file 44318_2025_614_MOESM7_ESM.zip › Fig 5/Fig 5C/6.1_FER YNE +ROL23 YCE/15.tif]

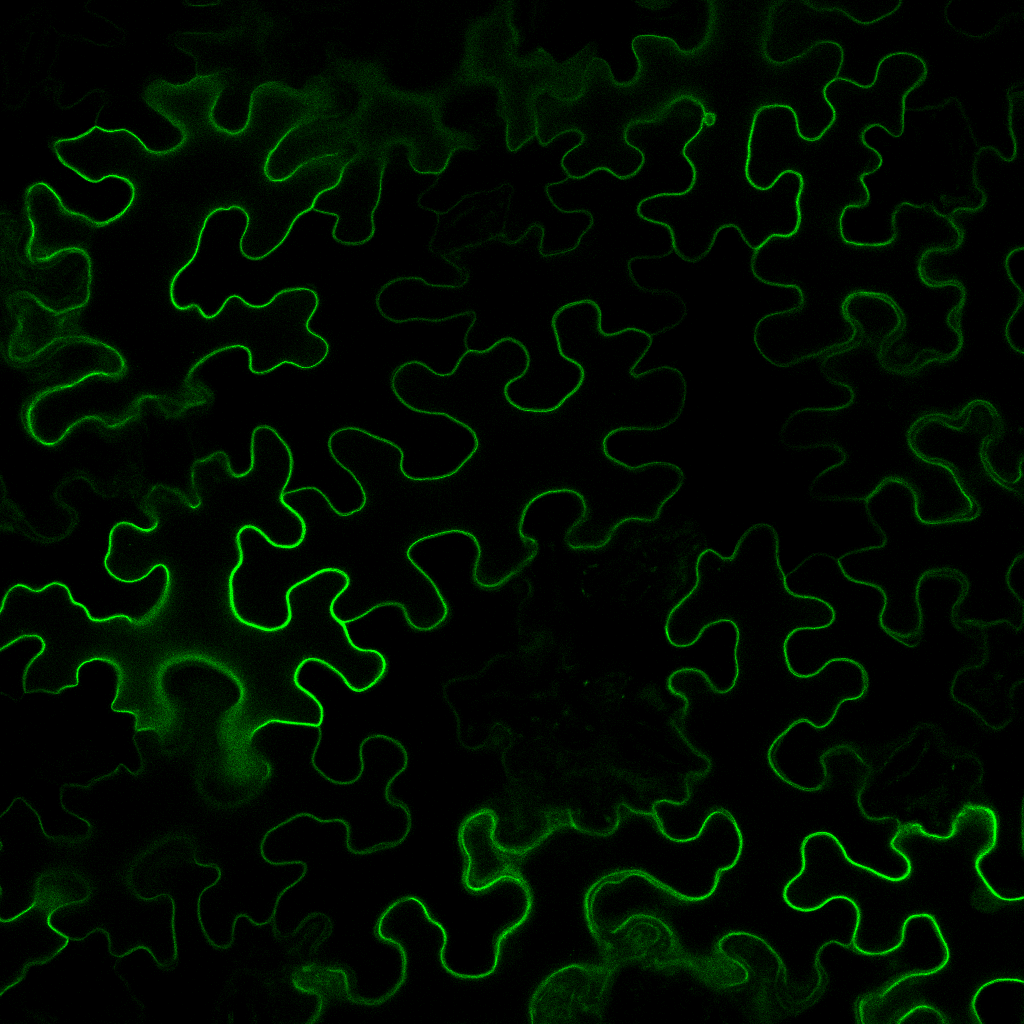

Supplement: Supplementary file 7 — Source data Fig. 5 [file 44318_2025_614_MOESM7_ESM.zip › Fig 5/Fig 5C/6.1_FER YNE +ROL23 YCE/15_ch00.tif]

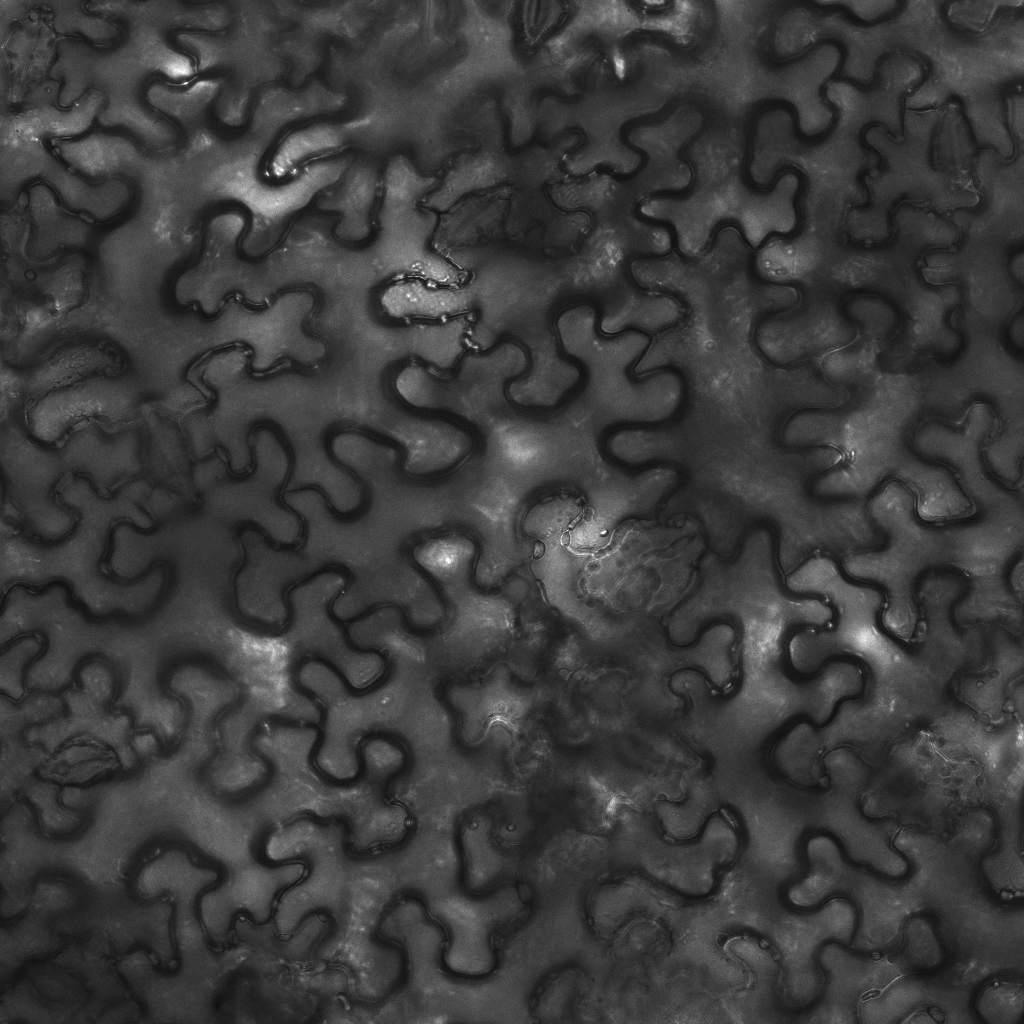

Supplement: Supplementary file 7 — Source data Fig. 5 [file 44318_2025_614_MOESM7_ESM.zip › Fig 5/Fig 5C/6.1_FER YNE +ROL23 YCE/15_ch01.tif]

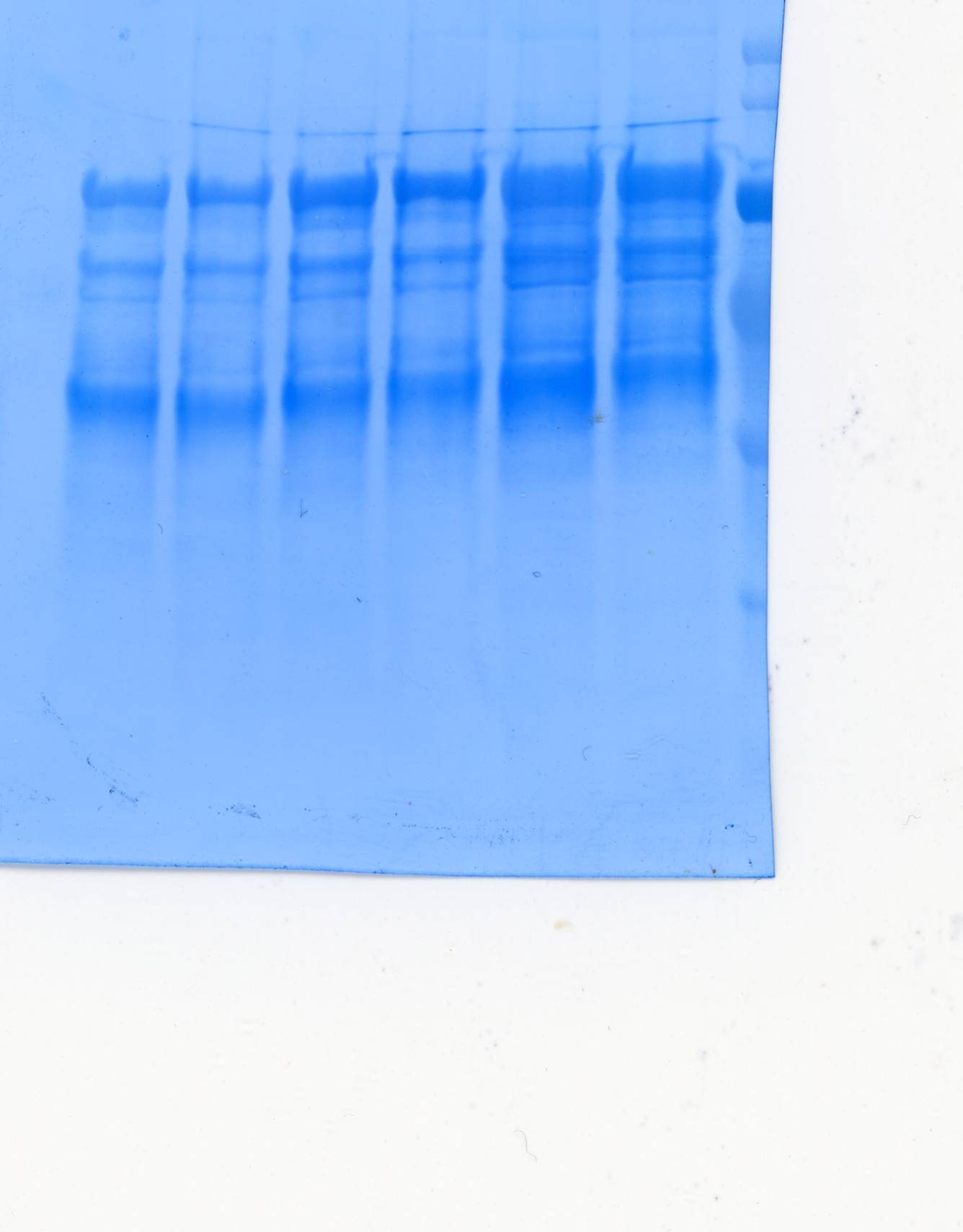

Supplement: Supplementary file 8 — Source data Fig. 6 [file 44318_2025_614_MOESM8_ESM.zip › Fig 6/Fig 6A/Fig 6A CBB staining.tif]

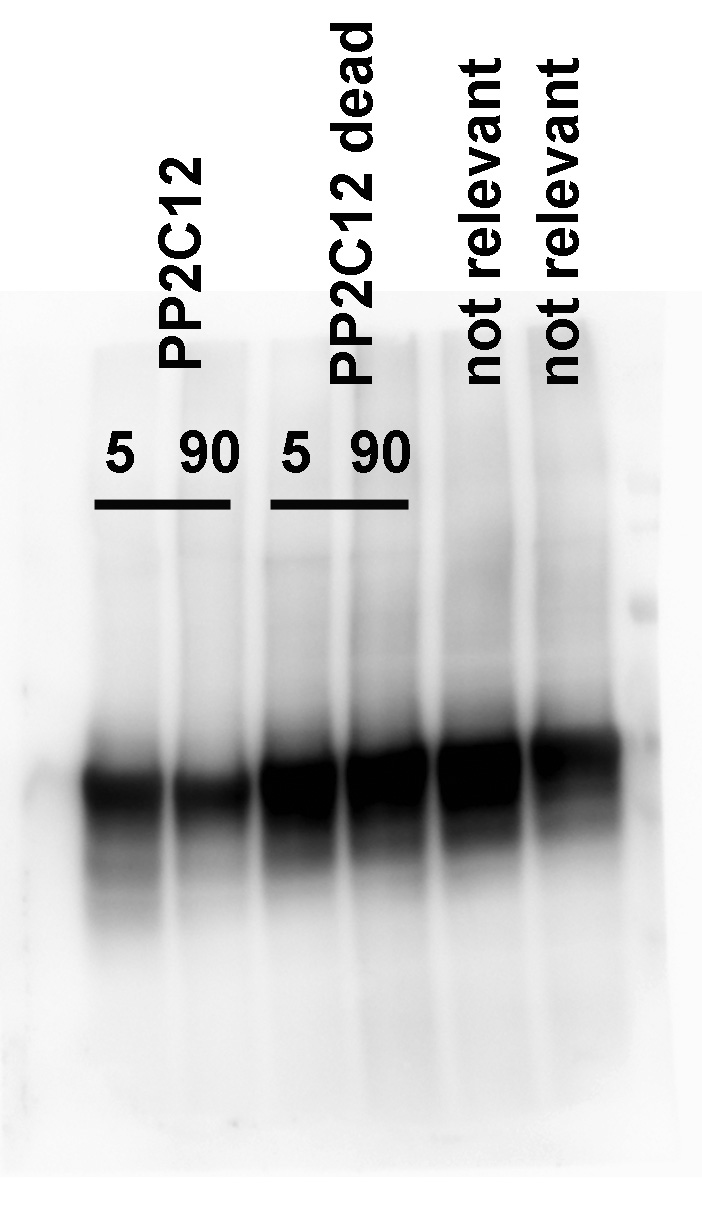

Supplement: Supplementary file 8 — Source data Fig. 6 [file 44318_2025_614_MOESM8_ESM.zip › Fig 6/Fig 6A/Fig 6A immunoblot.jpg]

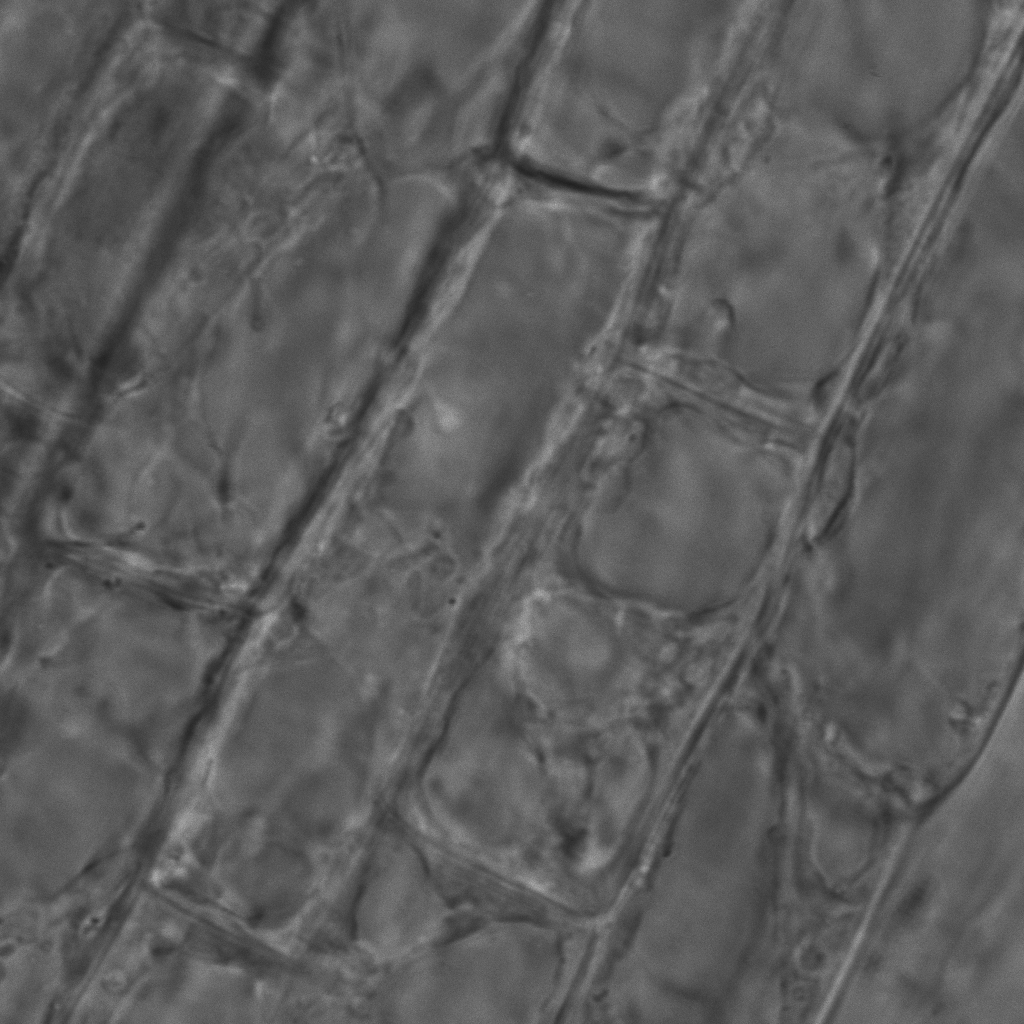

Supplement: Supplementary file 9 — Source data Fig. 8 [file 44318_2025_614_MOESM9_ESM.zip › Fig 8/Fig 8A/Fig 8A fer-4 FER-GFP bright.tif]

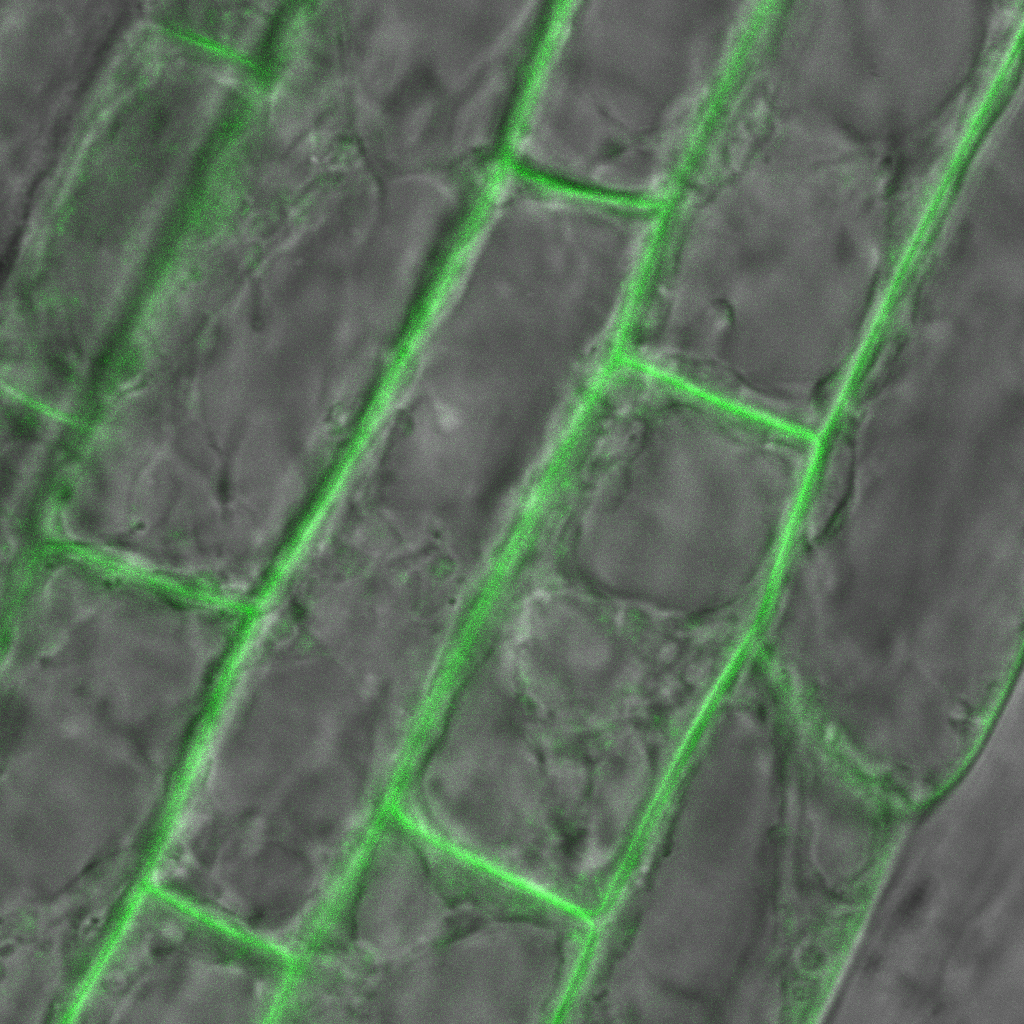

Supplement: Supplementary file 9 — Source data Fig. 8 [file 44318_2025_614_MOESM9_ESM.zip › Fig 8/Fig 8A/Fig 8A fer-4 FER-GFP fluor.tif]
