# Supplementary figures and images for: The Arabidopsis phosphatase PP2C12 negatively regulates LRX-RALF-FER-mediated cell wall integrity sensing (part 2 of 2)
Source: EMBO J. 2025 Nov 17;45(1):243–60. doi: 10.1038/s44318-025-00614-x (PMC12759080; doi:10.1038/s44318-025-00614-x)

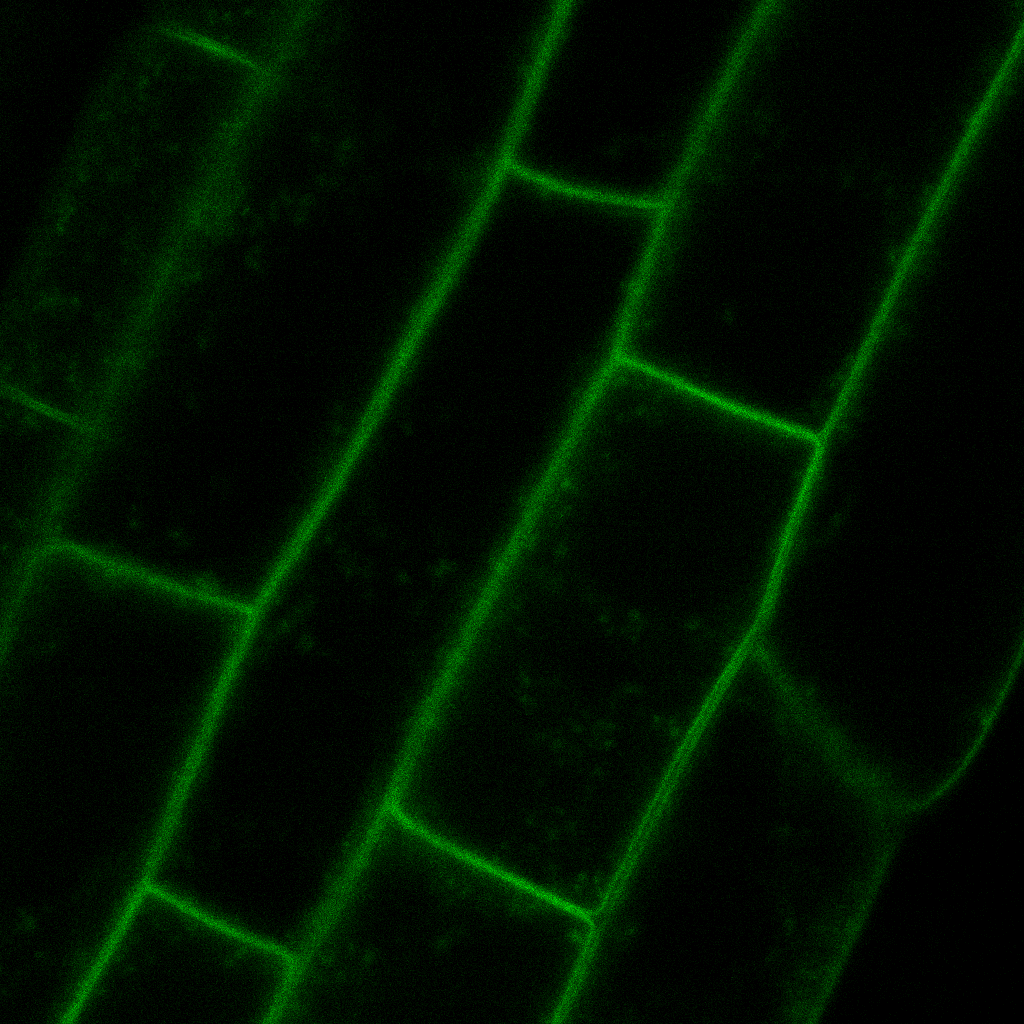

Supplement: Supplementary file 9 — Source data Fig. 8 [file 44318_2025_614_MOESM9_ESM.zip › Fig 8/Fig 8A/Fig 8A fer-4 FER-GFP merge.tif]

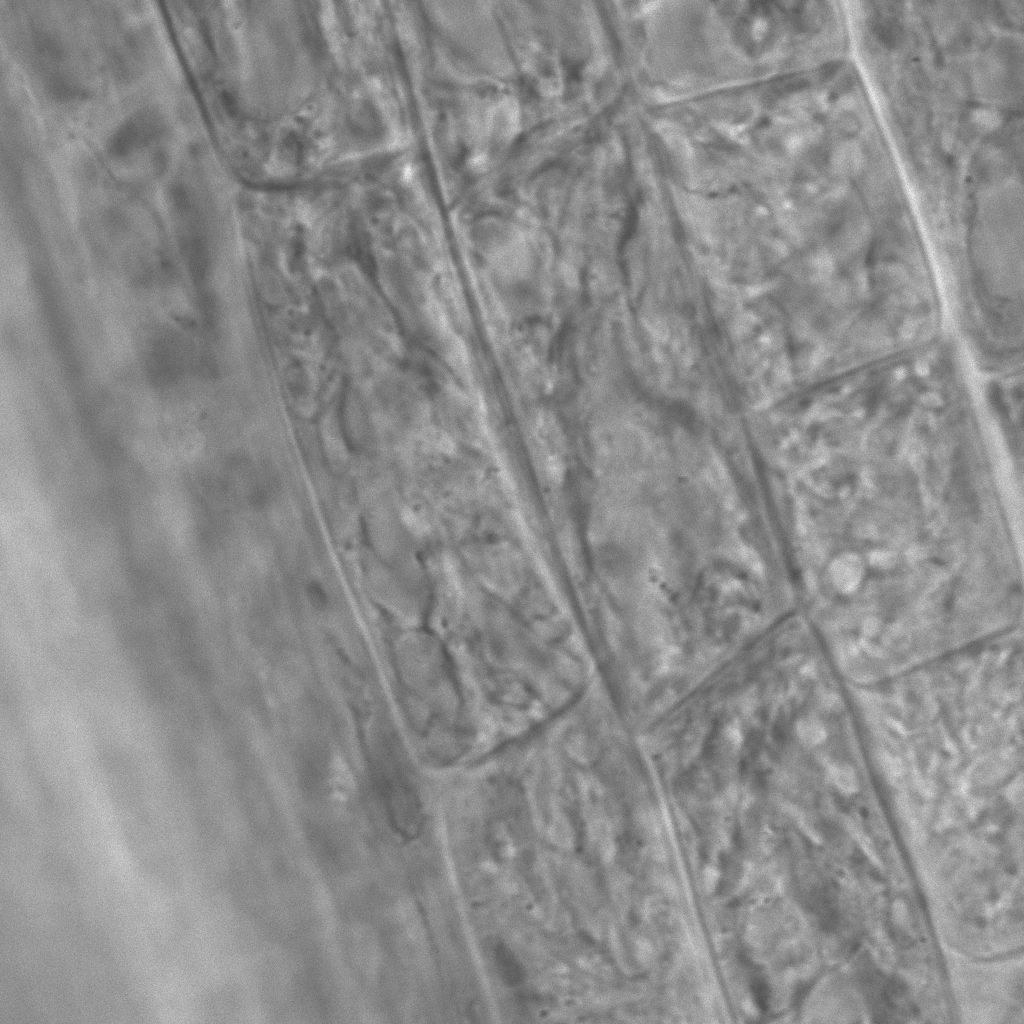

Supplement: Supplementary file 9 — Source data Fig. 8 [file 44318_2025_614_MOESM9_ESM.zip › Fig 8/Fig 8A/Fig 8A fer-4 FER-GFP triple bright.tif]

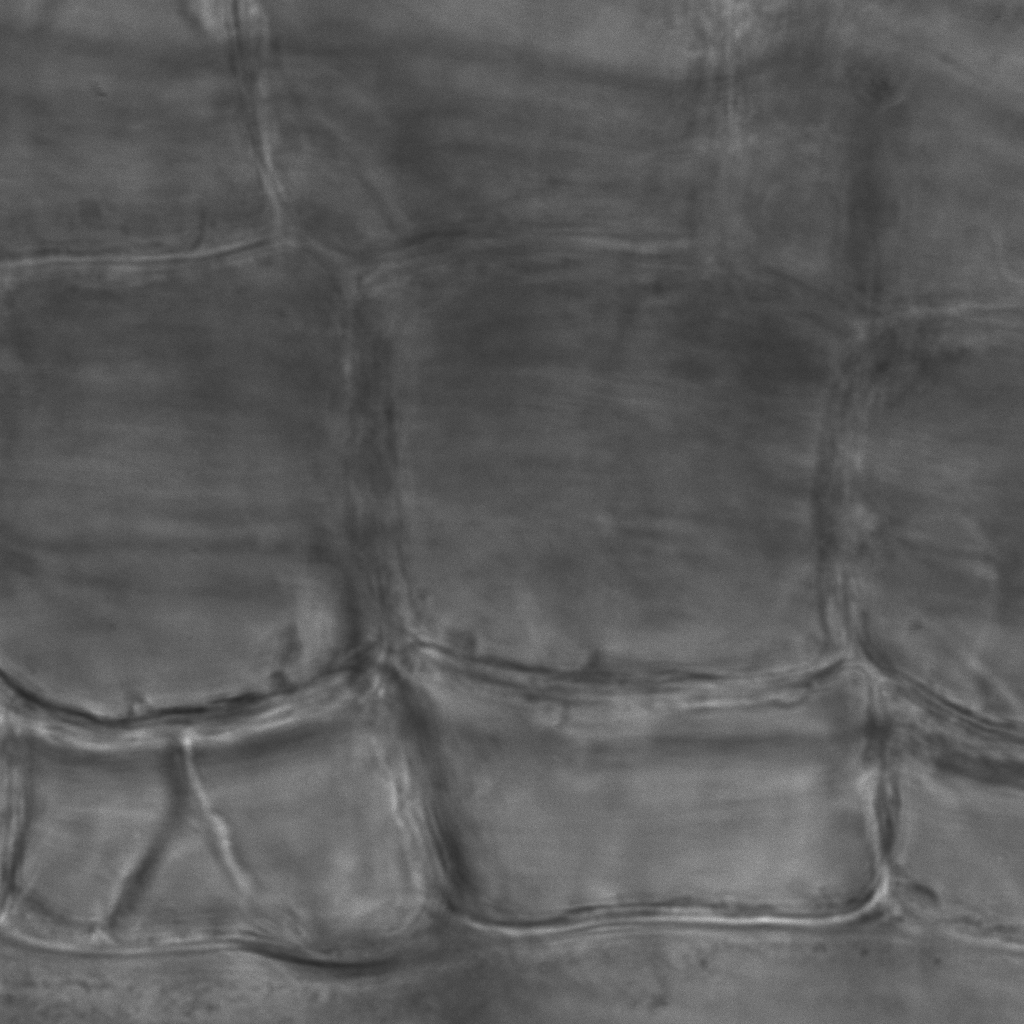

Supplement: Supplementary file 9 — Source data Fig. 8 [file 44318_2025_614_MOESM9_ESM.zip › Fig 8/Fig 8A/Fig 8A fer-4 FER-GFP triple EGCG bright.tif]

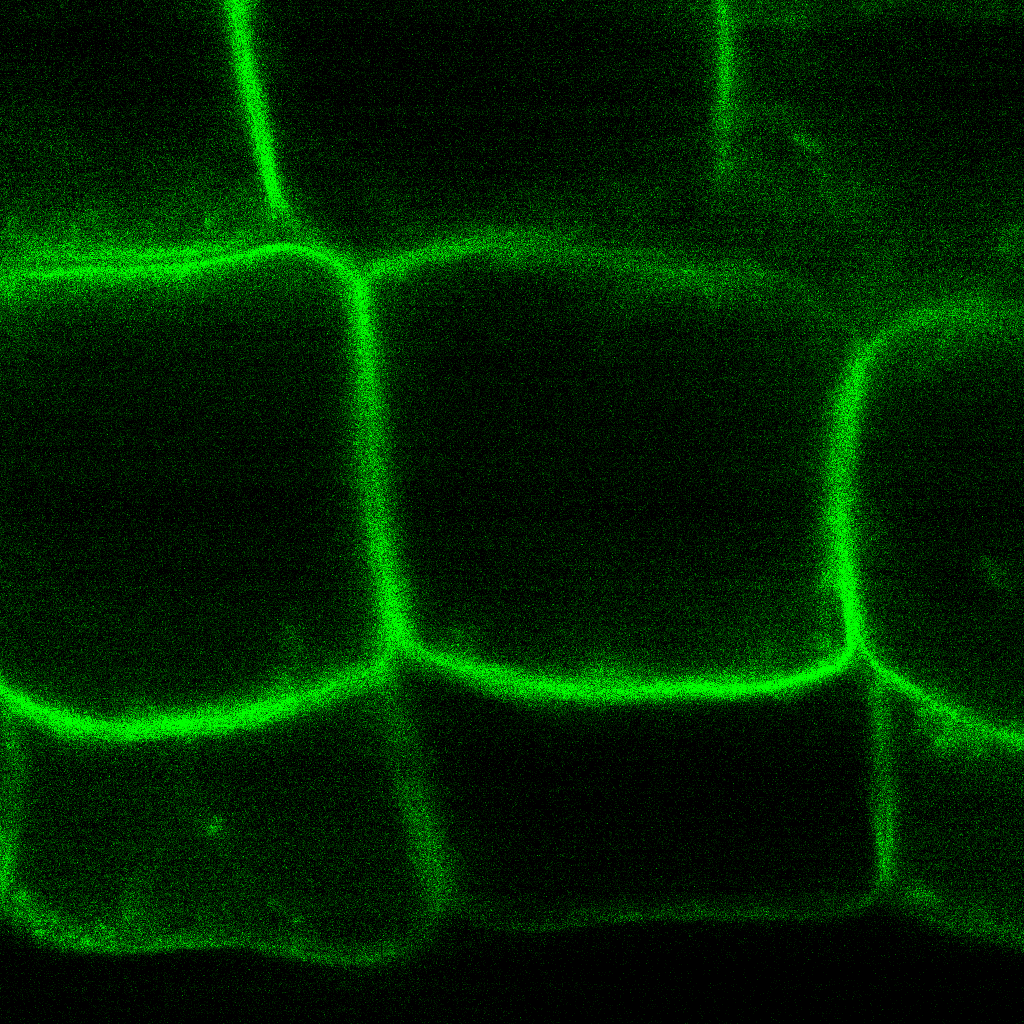

Supplement: Supplementary file 9 — Source data Fig. 8 [file 44318_2025_614_MOESM9_ESM.zip › Fig 8/Fig 8A/Fig 8A fer-4 FER-GFP triple EGCG fluor.tif]

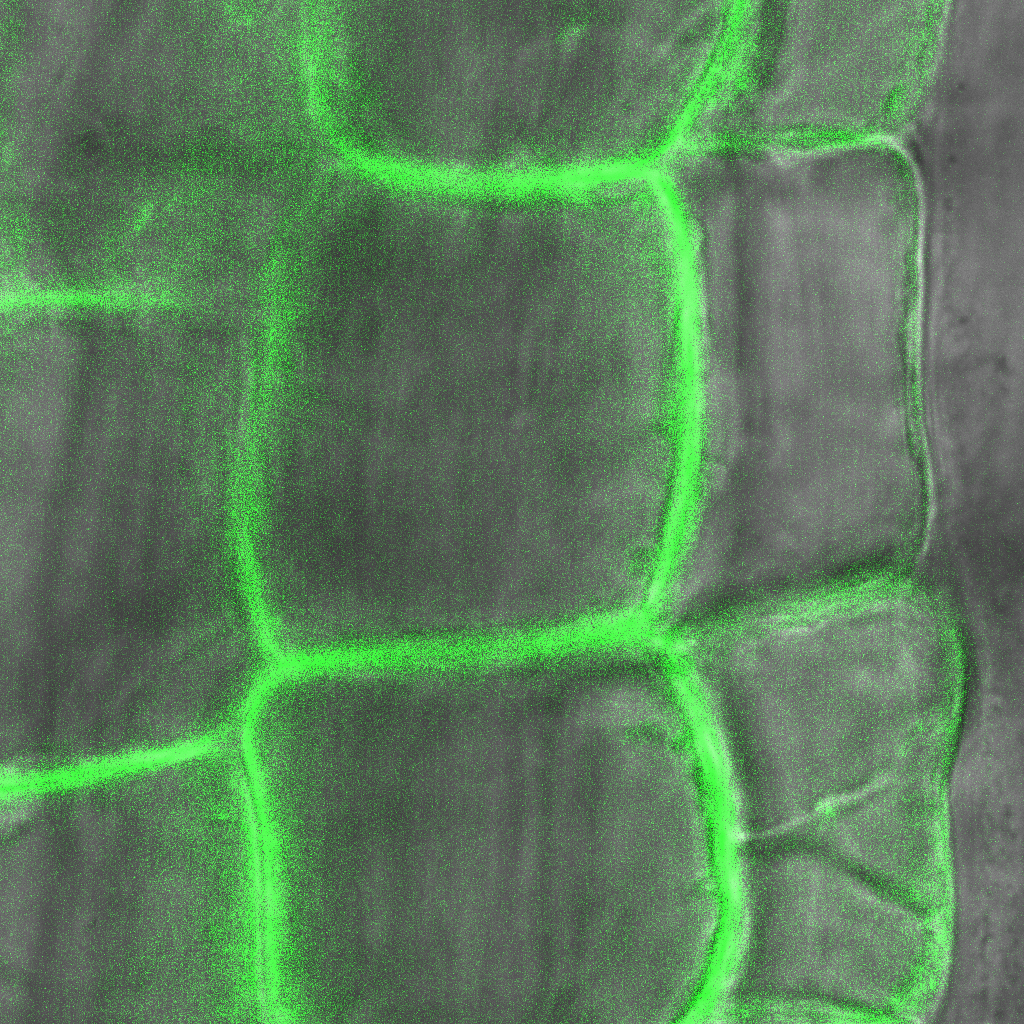

Supplement: Supplementary file 9 — Source data Fig. 8 [file 44318_2025_614_MOESM9_ESM.zip › Fig 8/Fig 8A/Fig 8A fer-4 FER-GFP triple EGCG merge.tif]

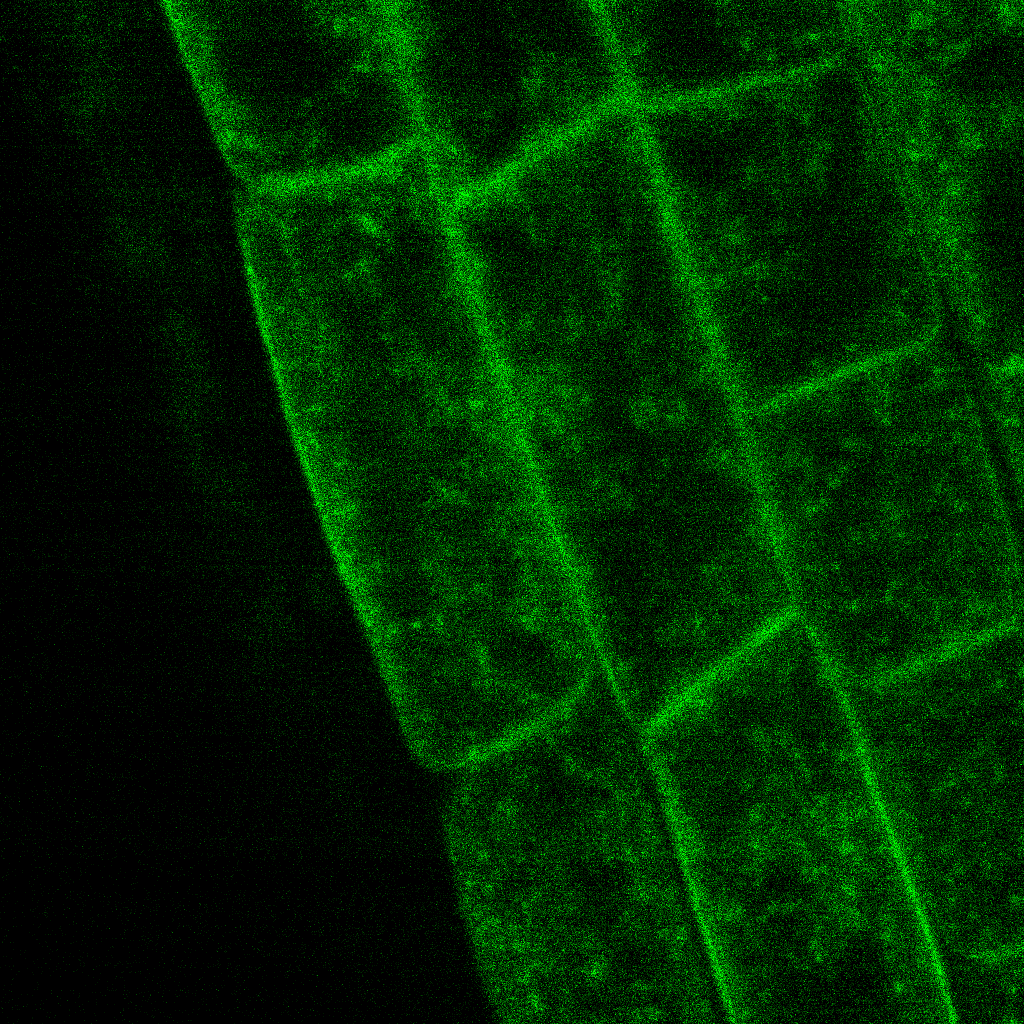

Supplement: Supplementary file 9 — Source data Fig. 8 [file 44318_2025_614_MOESM9_ESM.zip › Fig 8/Fig 8A/Fig 8A fer-4 FER-GFP triple fluor.tif]

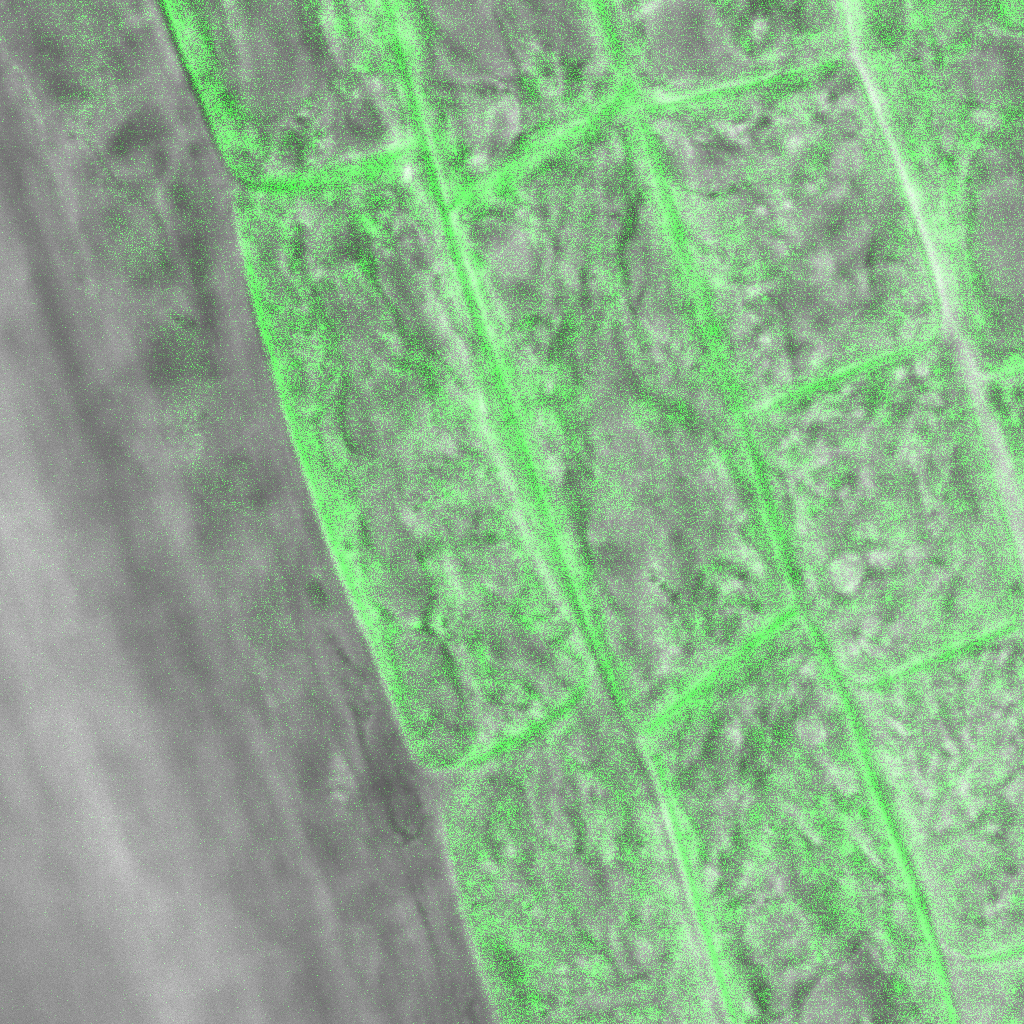

Supplement: Supplementary file 9 — Source data Fig. 8 [file 44318_2025_614_MOESM9_ESM.zip › Fig 8/Fig 8A/Fig 8A fer-4 FER-GFP triple merge.tif]

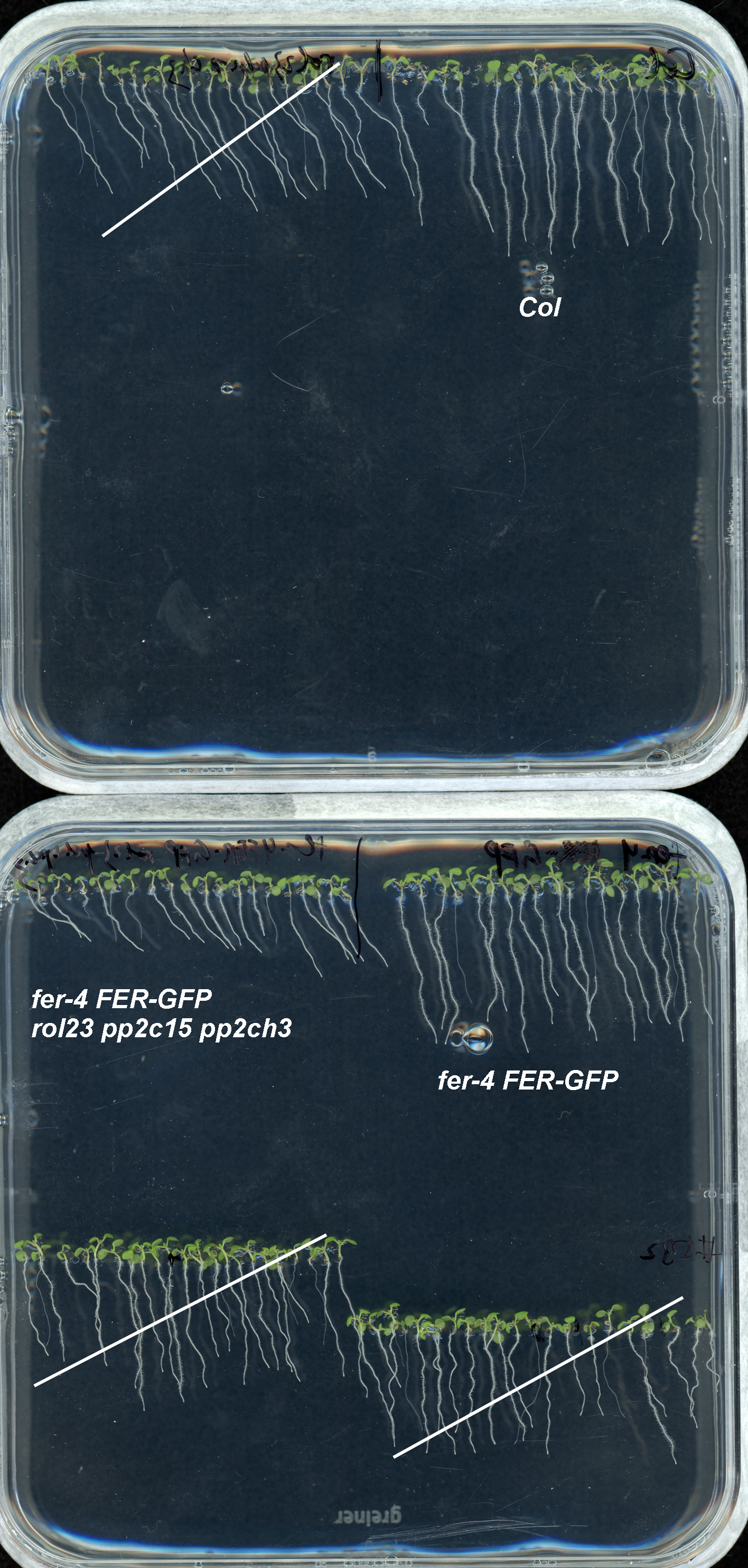

Supplement: Supplementary file 10 — Source data Fig. 9 [file 44318_2025_614_MOESM10_ESM.zip › Fig 9/Fig. 9A/Col fer4 compl triple.tif]

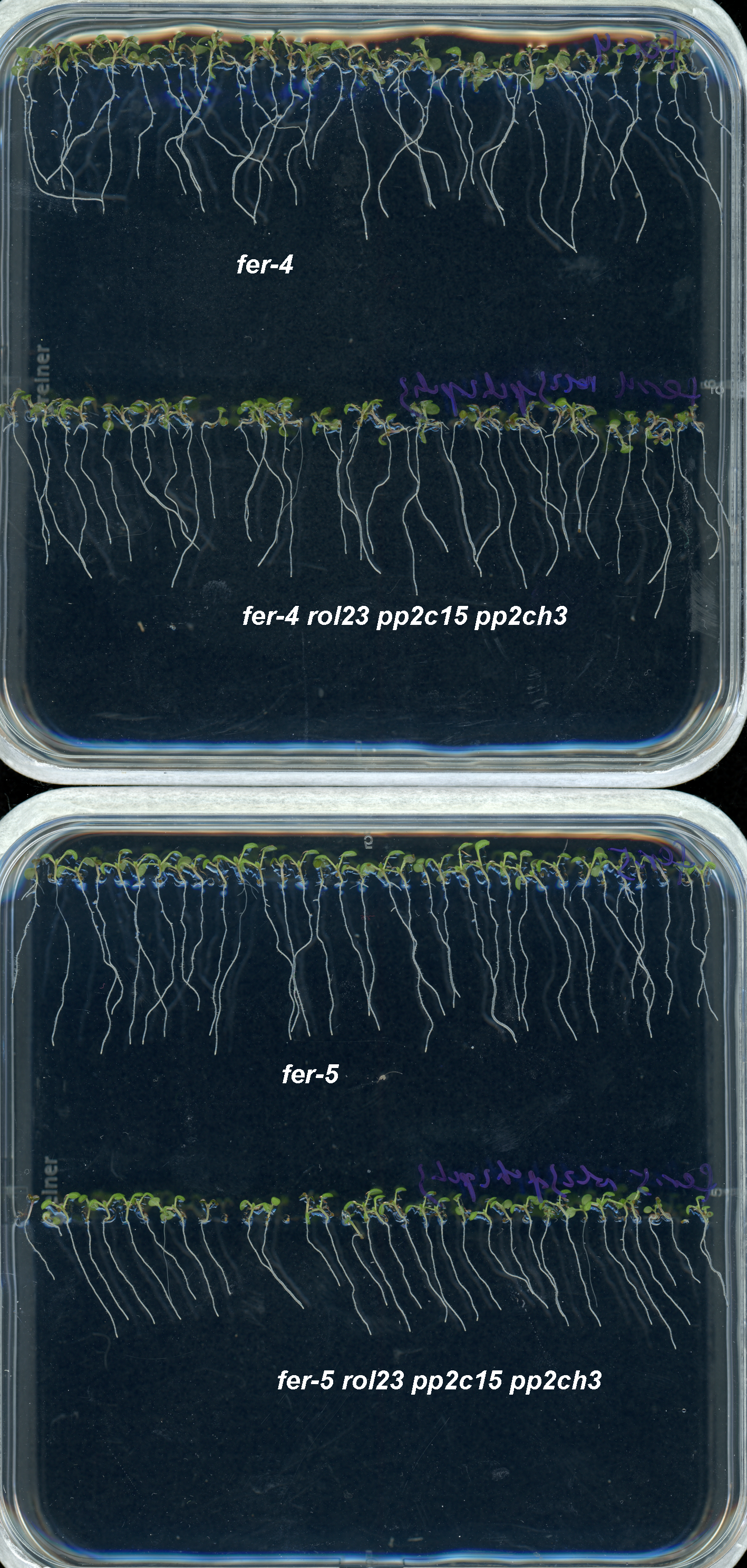

Supplement: Supplementary file 10 — Source data Fig. 9 [file 44318_2025_614_MOESM10_ESM.zip › Fig 9/Fig. 9A/fer4 fer5 triple.tif]

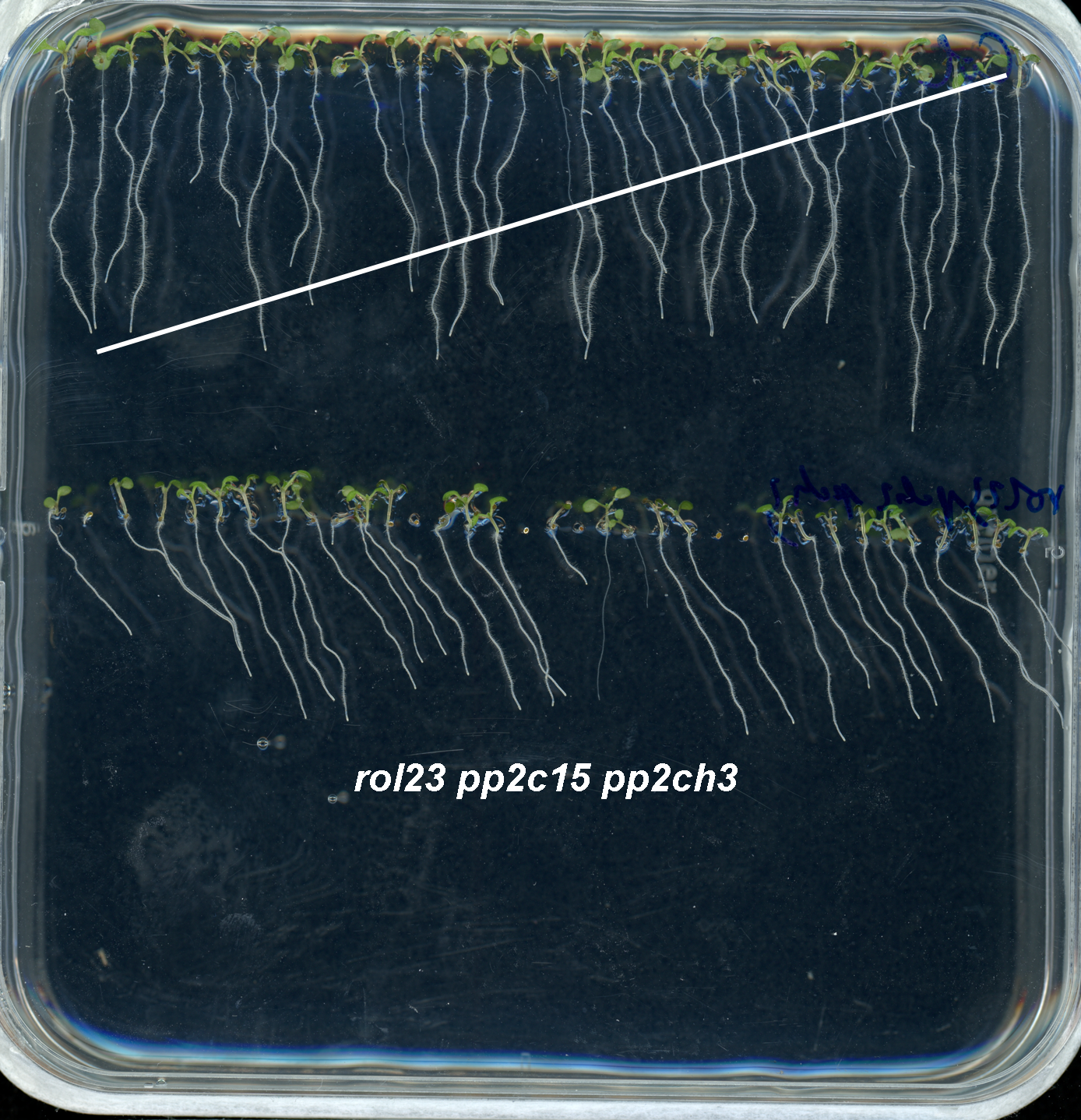

Supplement: Supplementary file 10 — Source data Fig. 9 [file 44318_2025_614_MOESM10_ESM.zip › Fig 9/Fig. 9A/pp2c triple.tif]

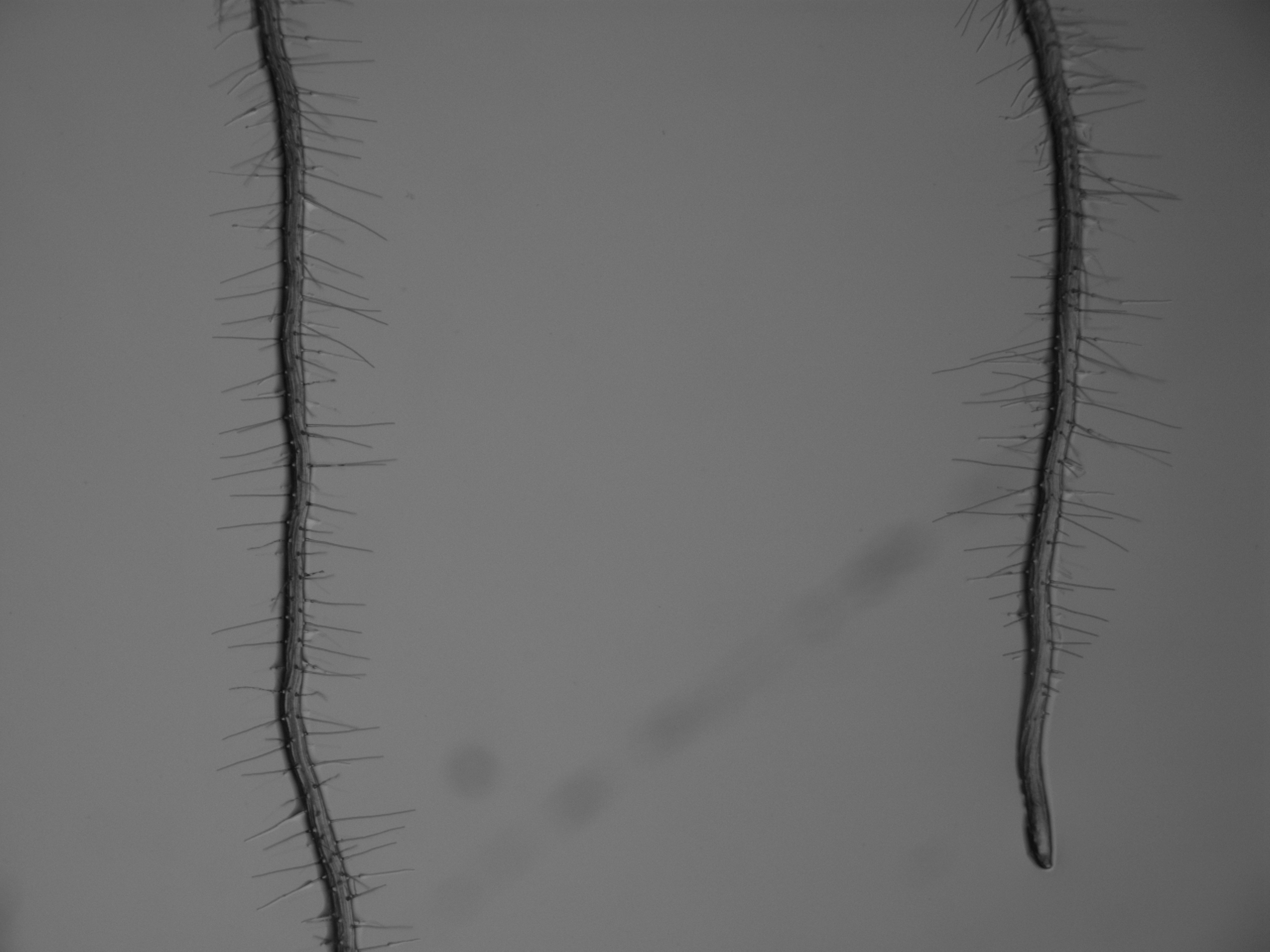

Supplement: Supplementary file 11 — Appendix Figure S1 Source Data [file 44318_2025_614_MOESM11_ESM.zip › Appendix Fig S1/Fig S1E/Col_1.tif]

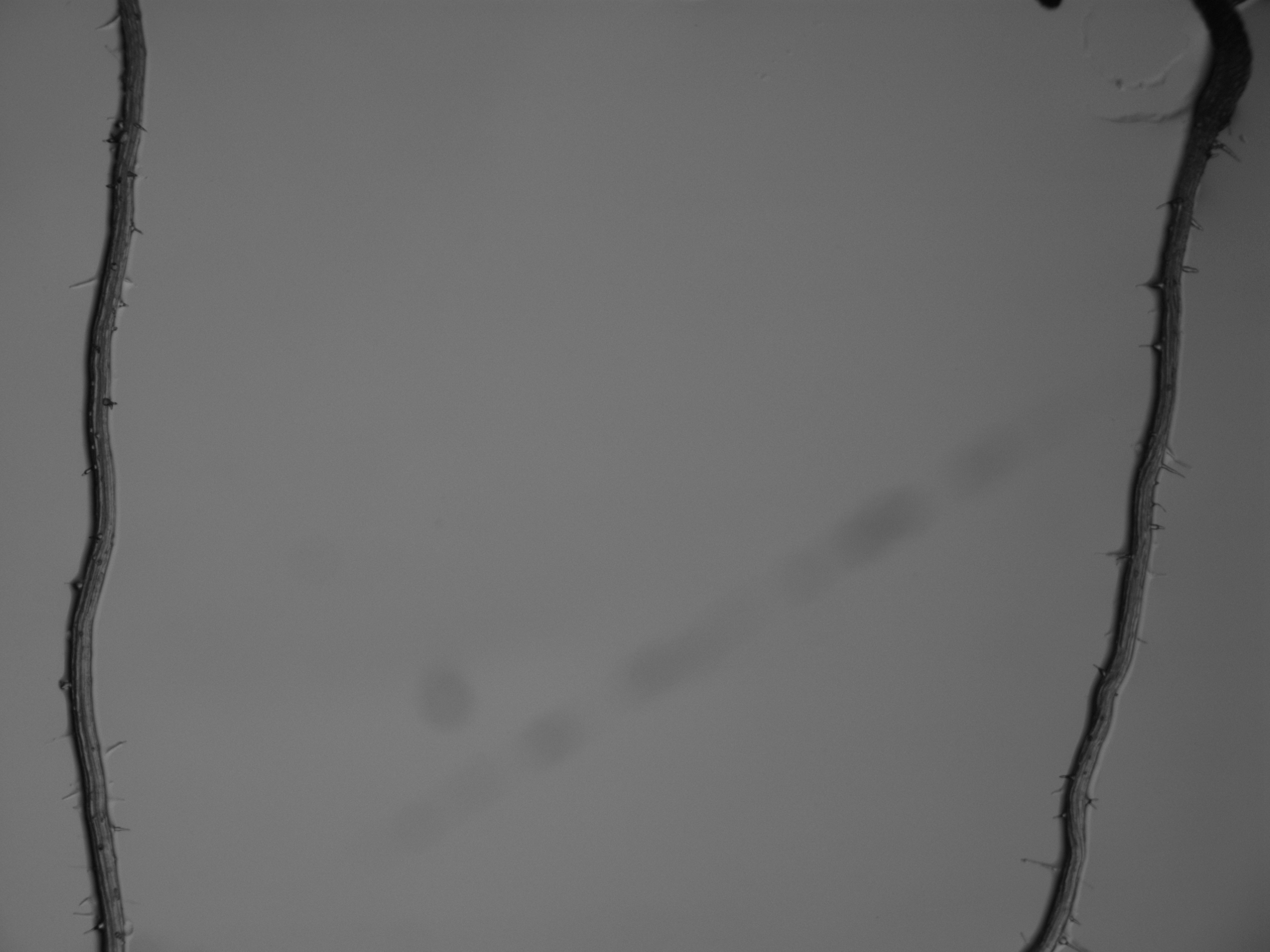

Supplement: Supplementary file 11 — Appendix Figure S1 Source Data [file 44318_2025_614_MOESM11_ESM.zip › Appendix Fig S1/Fig S1E/lrx1lrx2_3.tif]

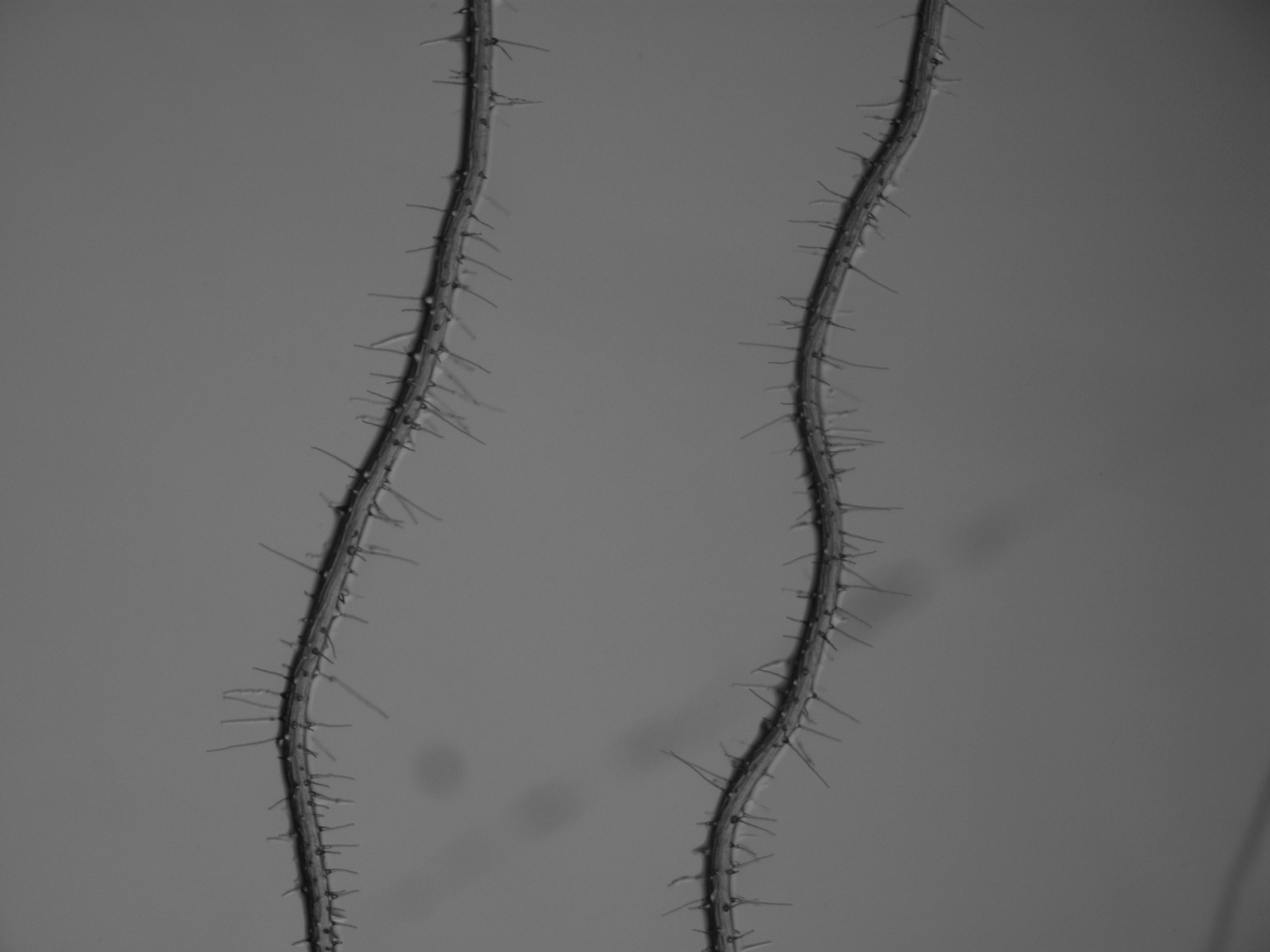

Supplement: Supplementary file 11 — Appendix Figure S1 Source Data [file 44318_2025_614_MOESM11_ESM.zip › Appendix Fig S1/Fig S1E/lrx1lrx2pp2c12_2.tif]

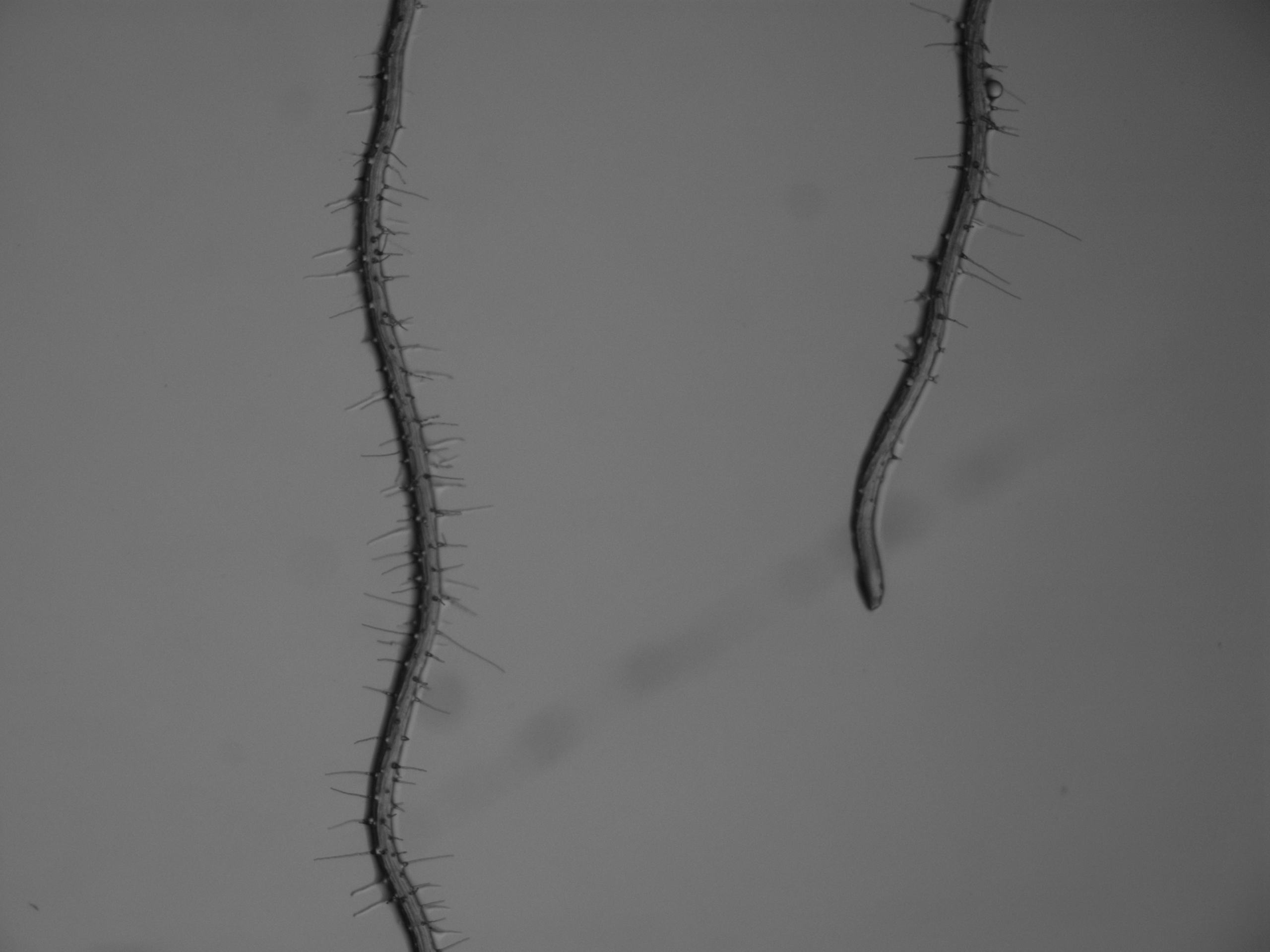

Supplement: Supplementary file 11 — Appendix Figure S1 Source Data [file 44318_2025_614_MOESM11_ESM.zip › Appendix Fig S1/Fig S1E/lrx1lrx2rol23_2.tif]

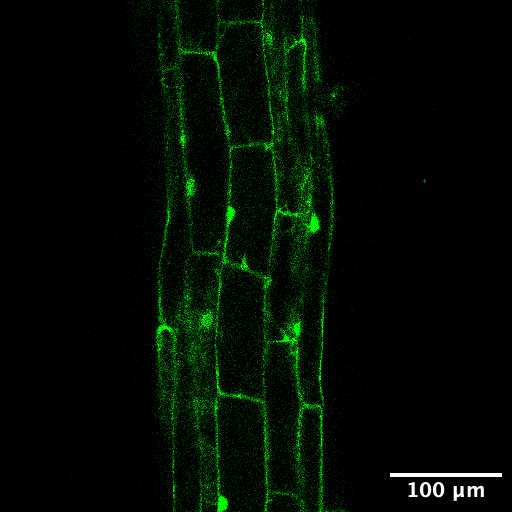

Supplement: Supplementary file 11 — Appendix Figure S1 Source Data [file 44318_2025_614_MOESM11_ESM.zip › Appendix Fig S1/Fig S1F/5.1_dC T2.1_10% smartG, 50% pinhole, 1.7 zoom, 8 accum, 10% laser_ch00.png]

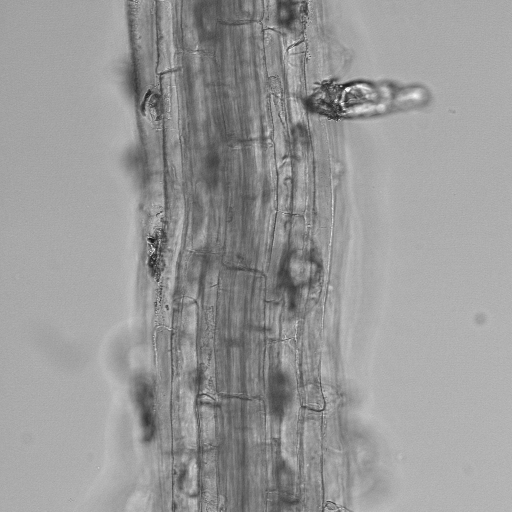

Supplement: Supplementary file 11 — Appendix Figure S1 Source Data [file 44318_2025_614_MOESM11_ESM.zip › Appendix Fig S1/Fig S1F/5.1_dC T2.1_10% smartG, 50% pinhole, 1.7 zoom, 8 accum, 10% laser_ch01.tif]

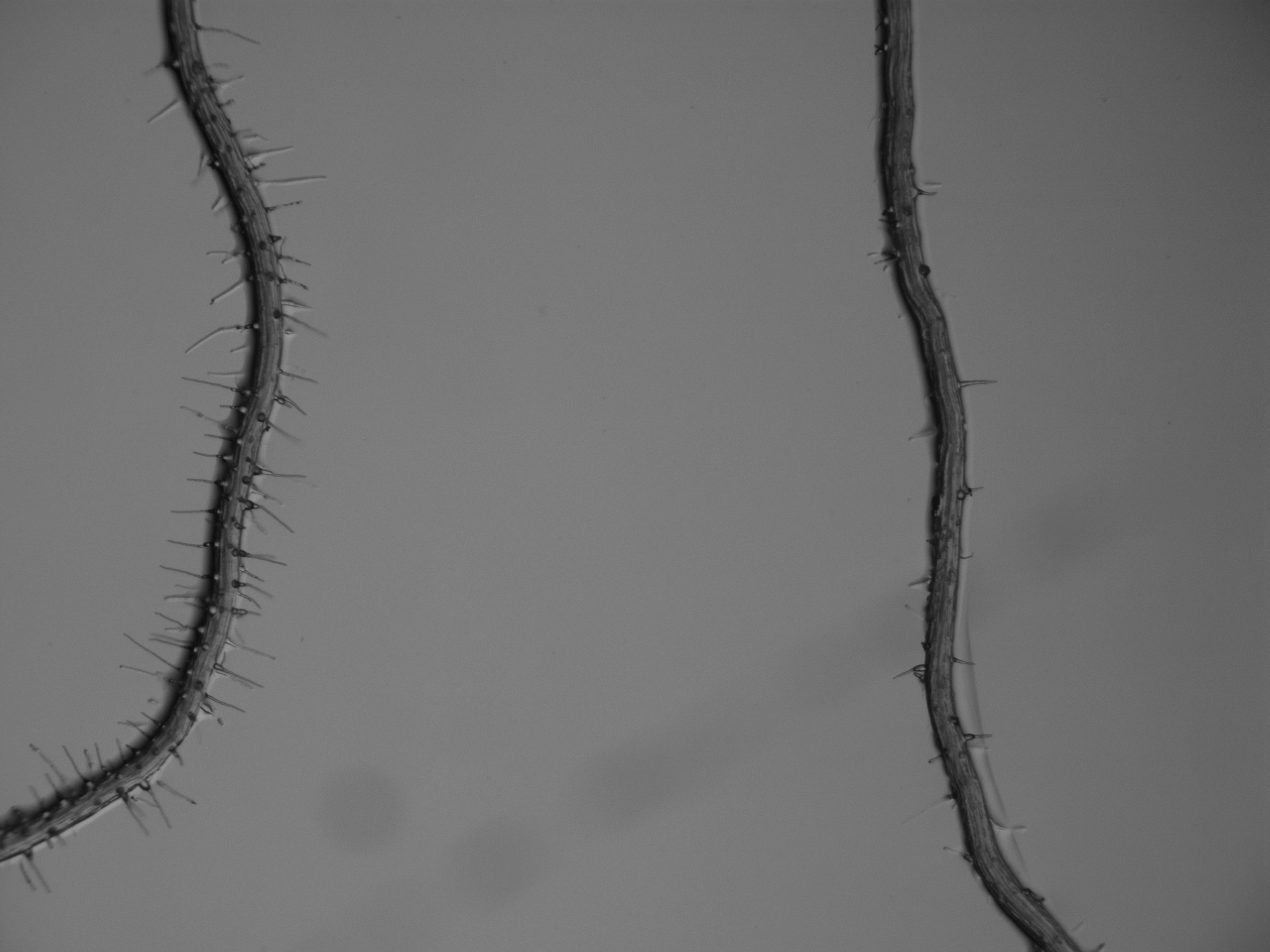

Supplement: Supplementary file 11 — Appendix Figure S1 Source Data [file 44318_2025_614_MOESM11_ESM.zip › Appendix Fig S1/Fig S1F/lrx12rol23NEST2_236_3.tif]

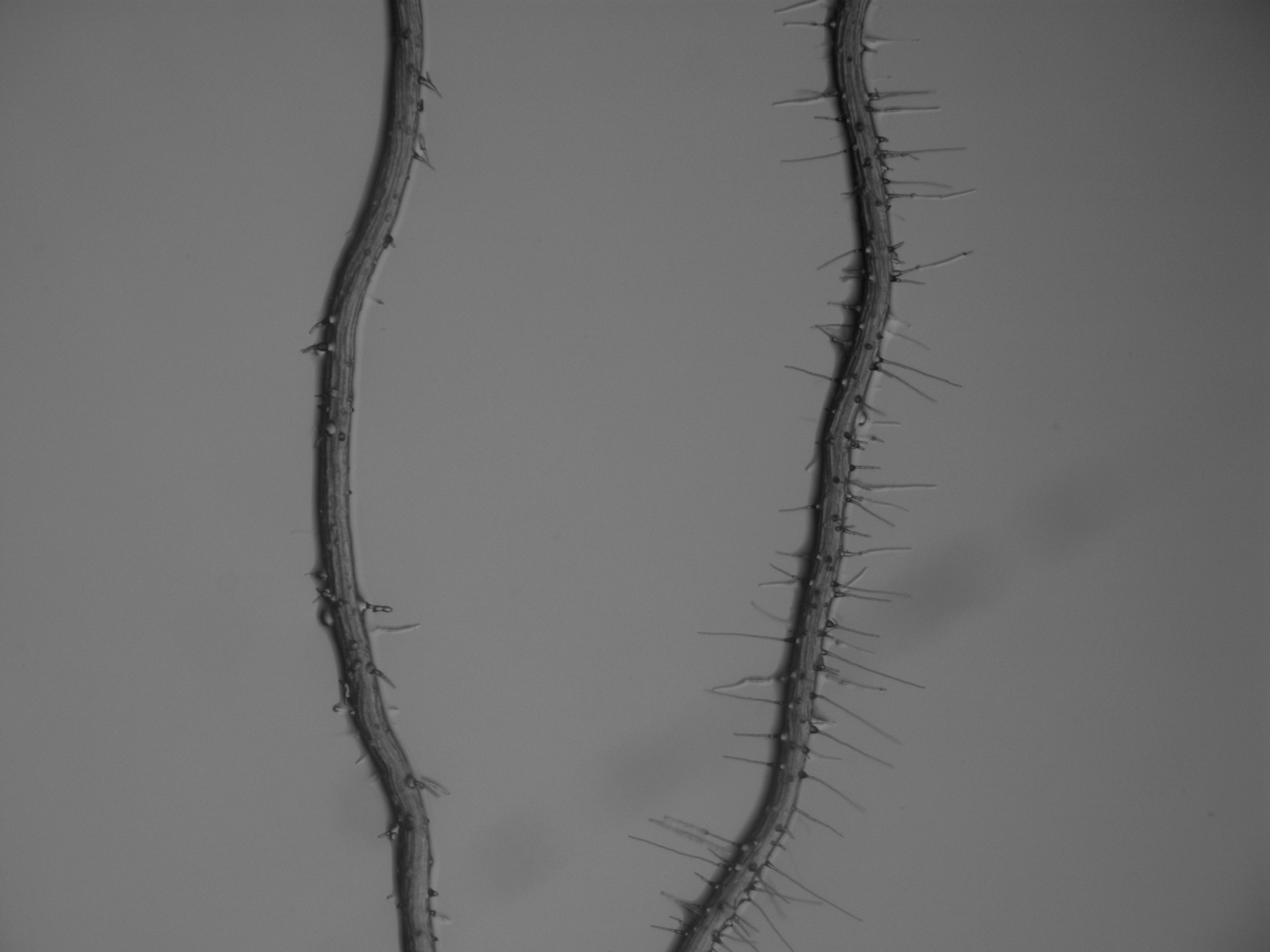

Supplement: Supplementary file 11 — Appendix Figure S1 Source Data [file 44318_2025_614_MOESM11_ESM.zip › Appendix Fig S1/Fig S1F/lrx12rol23nesT2_239_1.tif]

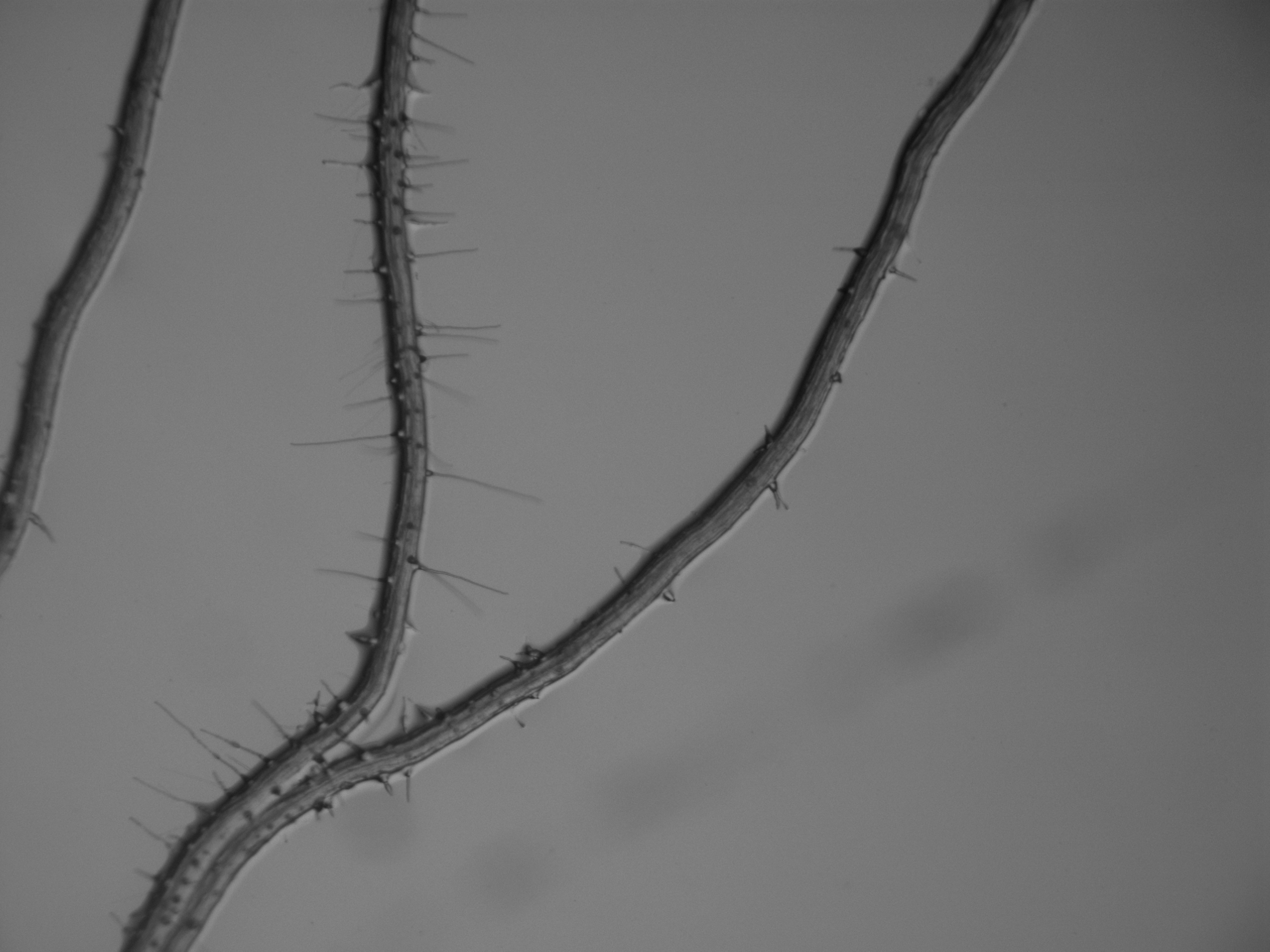

Supplement: Supplementary file 11 — Appendix Figure S1 Source Data [file 44318_2025_614_MOESM11_ESM.zip › Appendix Fig S1/Fig S1F/lrx12rol23PP2C12_1.tif]

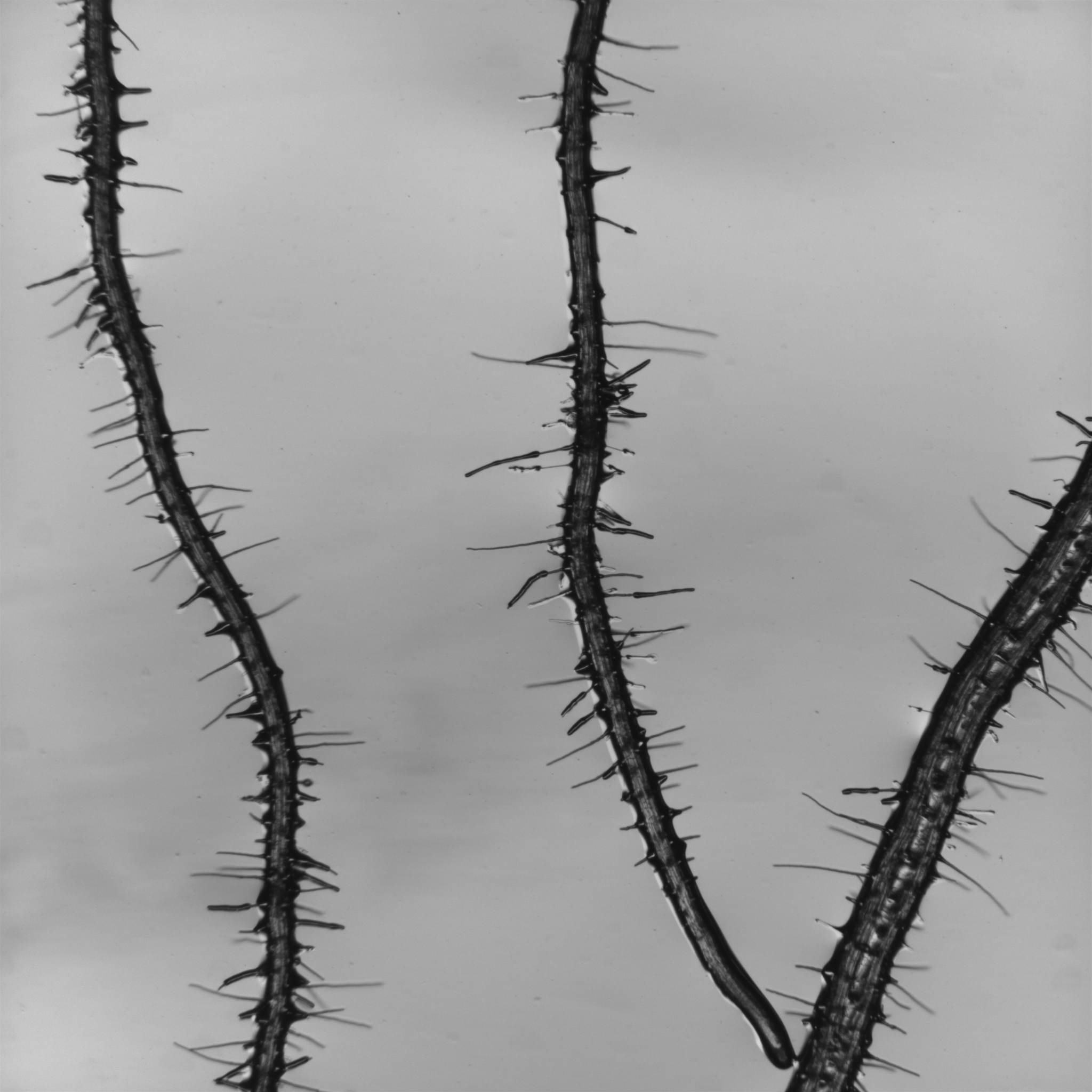

Supplement: Supplementary file 12 — Appendix Figure S2 Source Data [file 44318_2025_614_MOESM12_ESM.zip › Appendix Fig S2/PP2C12_bright_2.tif]

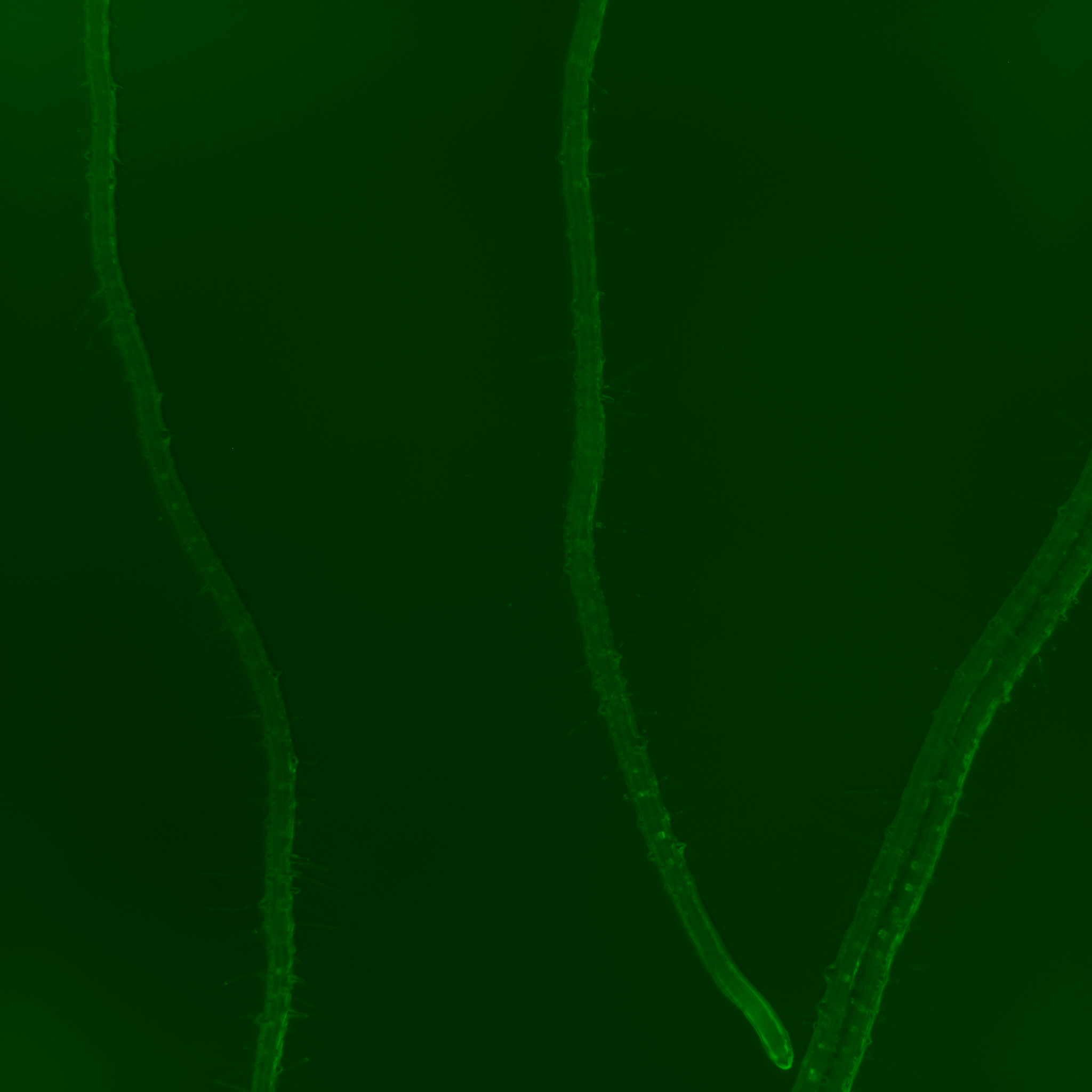

Supplement: Supplementary file 12 — Appendix Figure S2 Source Data [file 44318_2025_614_MOESM12_ESM.zip › Appendix Fig S2/PP2C12_GFP_2.tif]

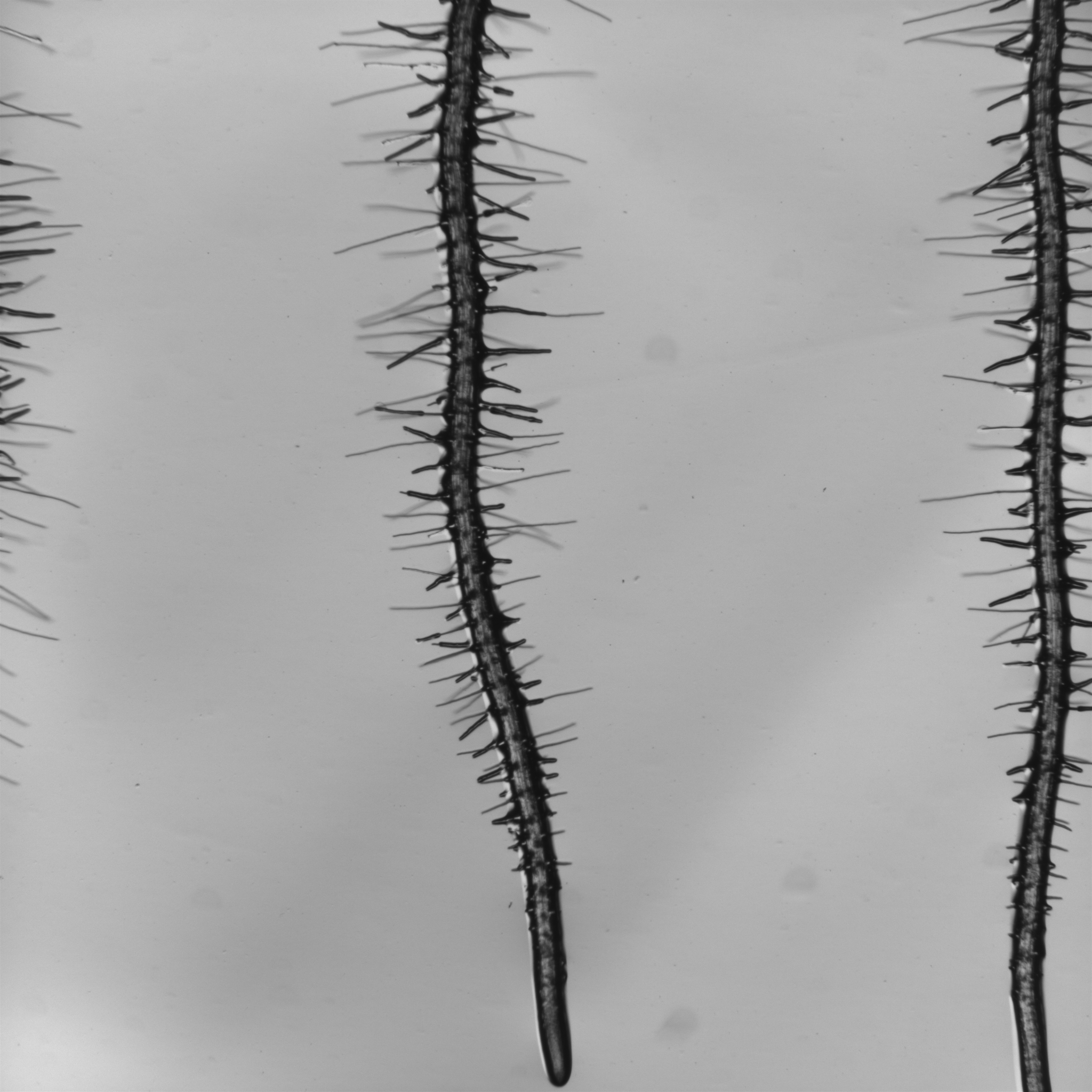

Supplement: Supplementary file 12 — Appendix Figure S2 Source Data [file 44318_2025_614_MOESM12_ESM.zip › Appendix Fig S2/PP2C35 bright_1.tif]

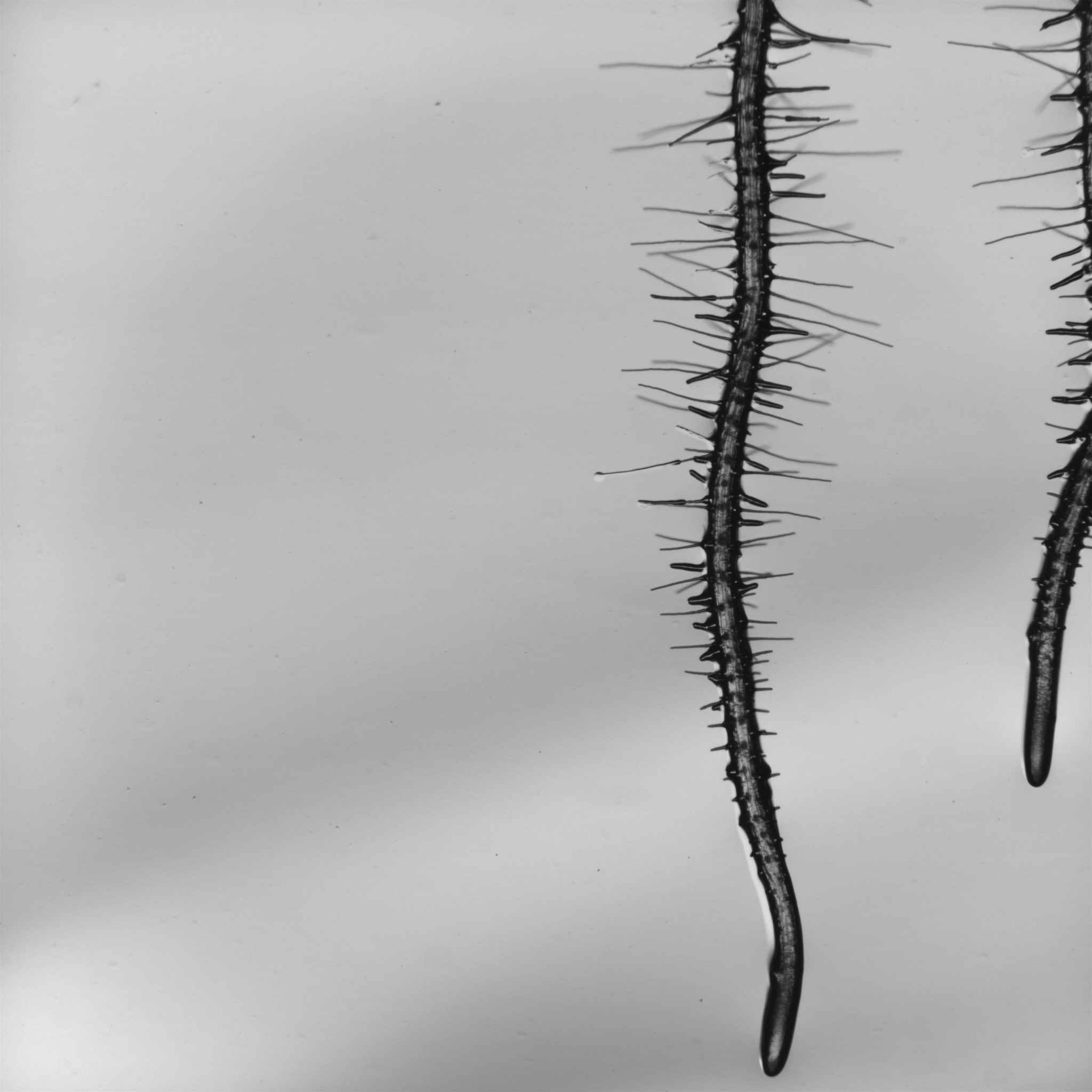

Supplement: Supplementary file 12 — Appendix Figure S2 Source Data [file 44318_2025_614_MOESM12_ESM.zip › Appendix Fig S2/PP2C35 bright_2.tif]

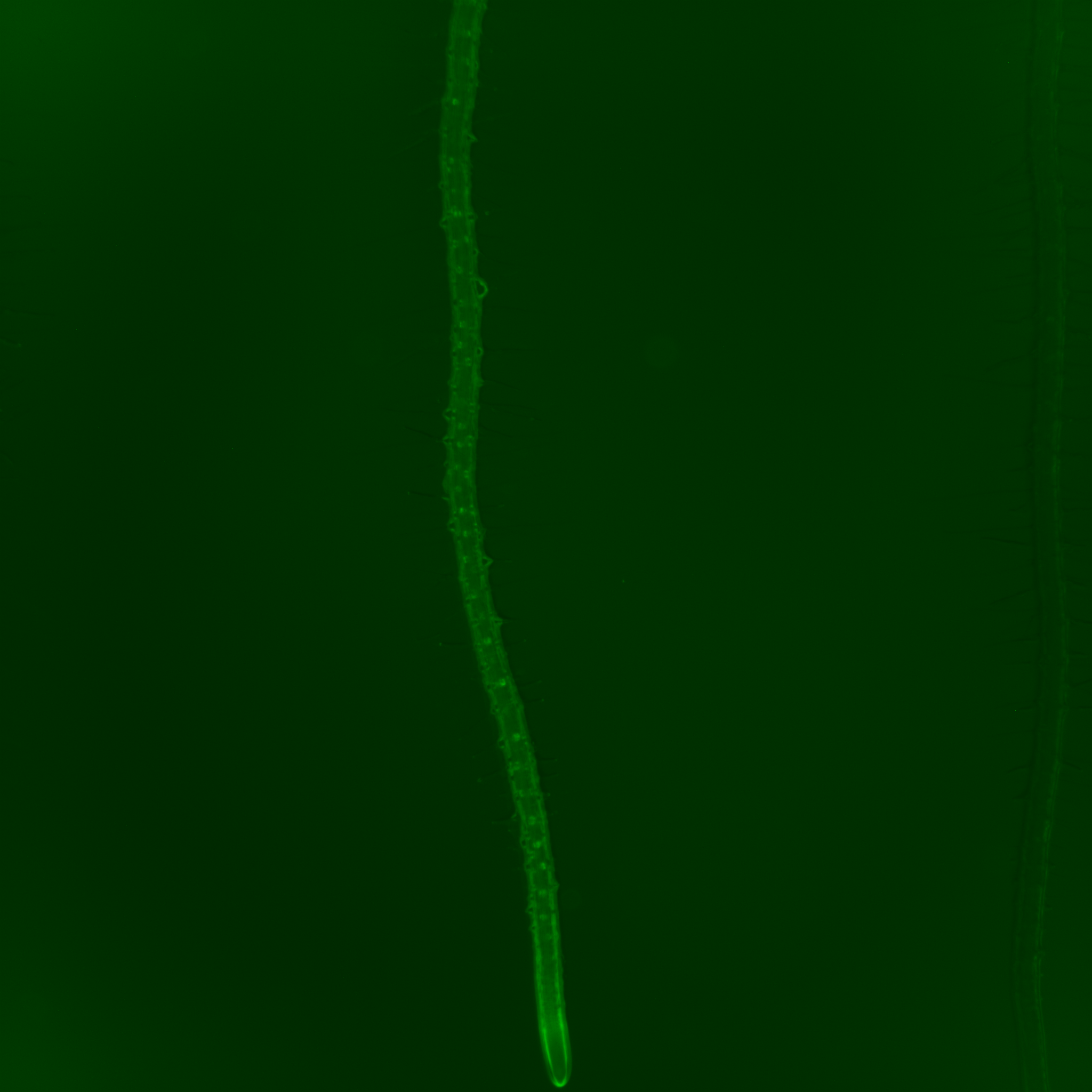

Supplement: Supplementary file 12 — Appendix Figure S2 Source Data [file 44318_2025_614_MOESM12_ESM.zip › Appendix Fig S2/PP2C35 GFP_1.tif]

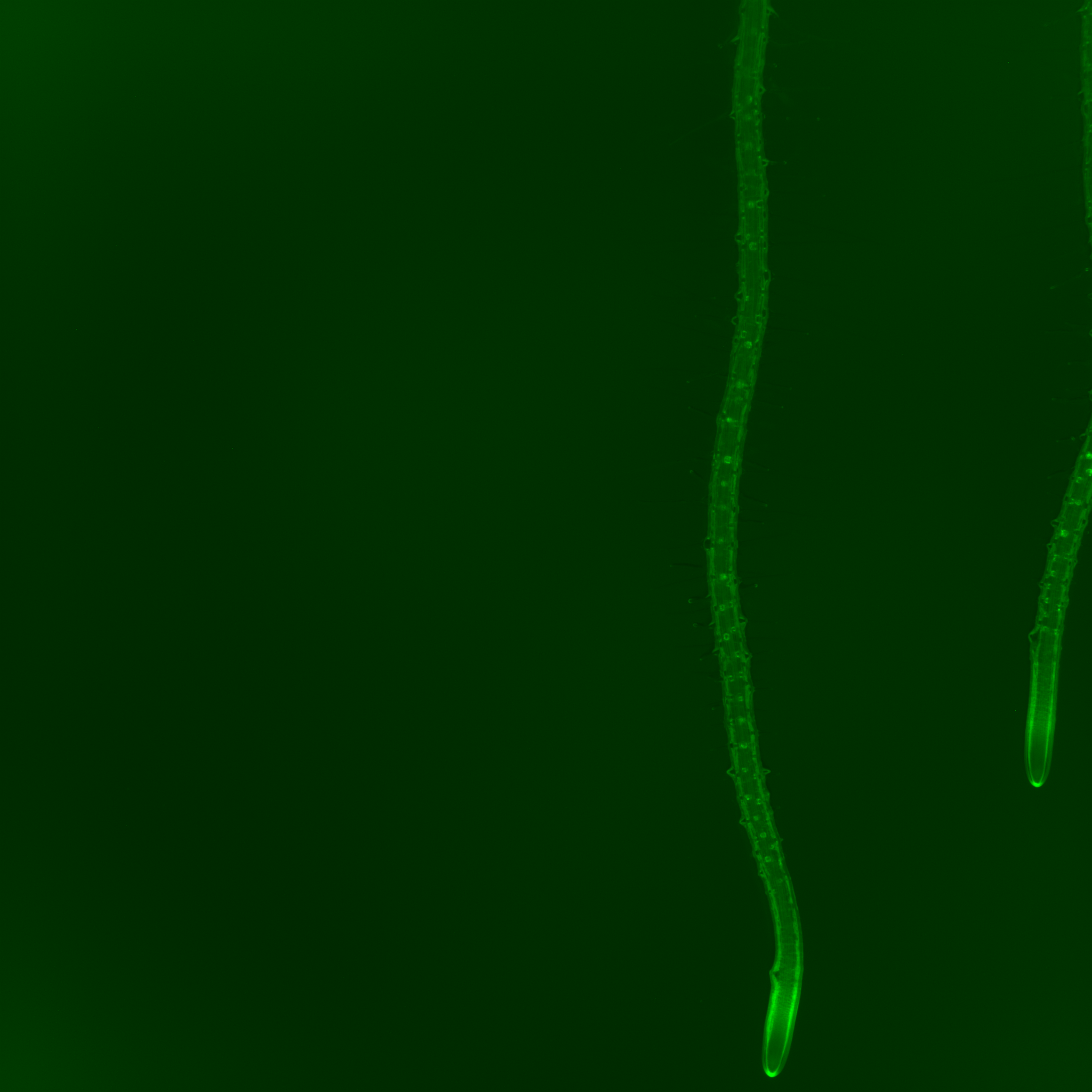

Supplement: Supplementary file 12 — Appendix Figure S2 Source Data [file 44318_2025_614_MOESM12_ESM.zip › Appendix Fig S2/PP2C35 GFP_2.tif]

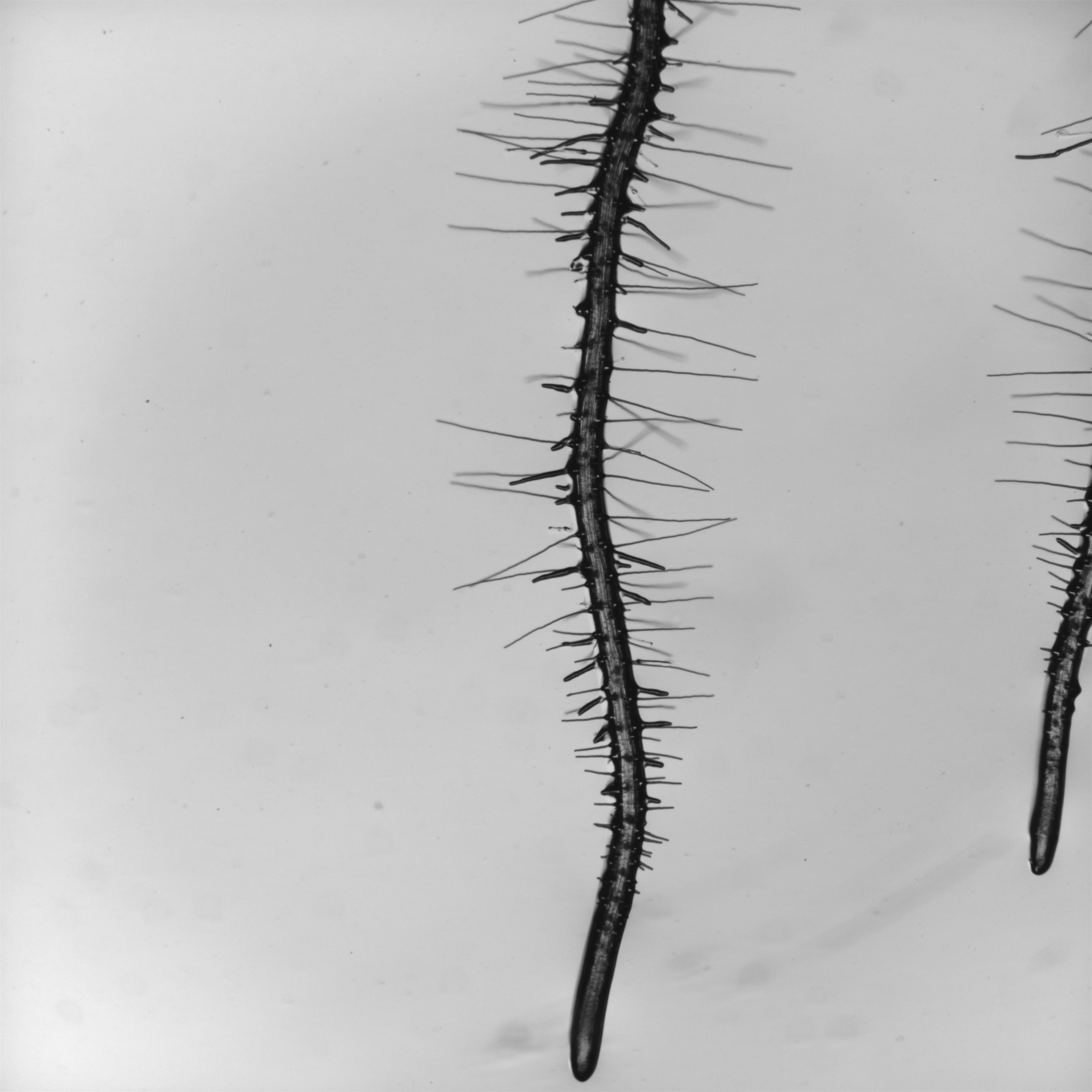

Supplement: Supplementary file 12 — Appendix Figure S2 Source Data [file 44318_2025_614_MOESM12_ESM.zip › Appendix Fig S2/PP2C38 bright.tif]

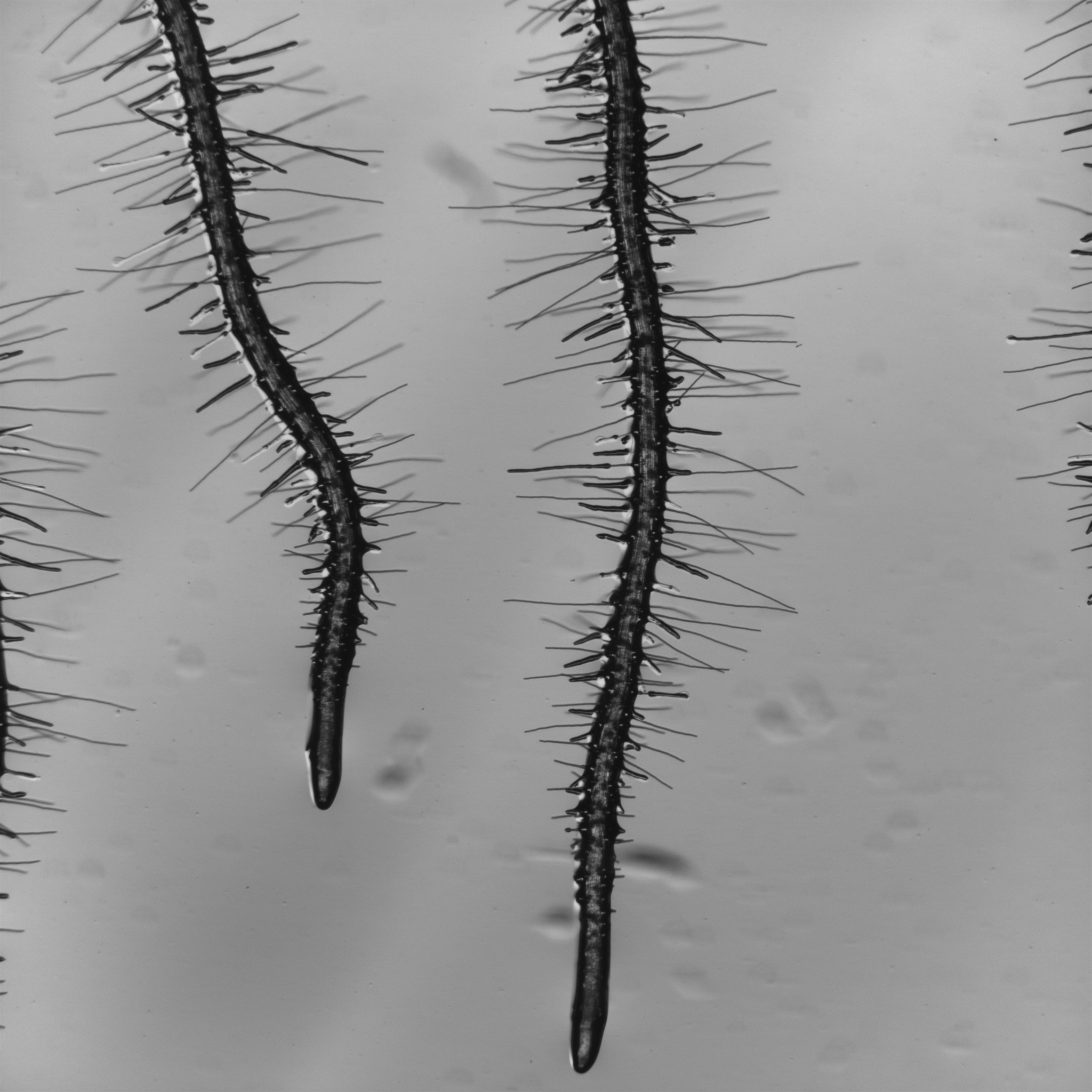

Supplement: Supplementary file 12 — Appendix Figure S2 Source Data [file 44318_2025_614_MOESM12_ESM.zip › Appendix Fig S2/PP2C38 bright_2.tif]

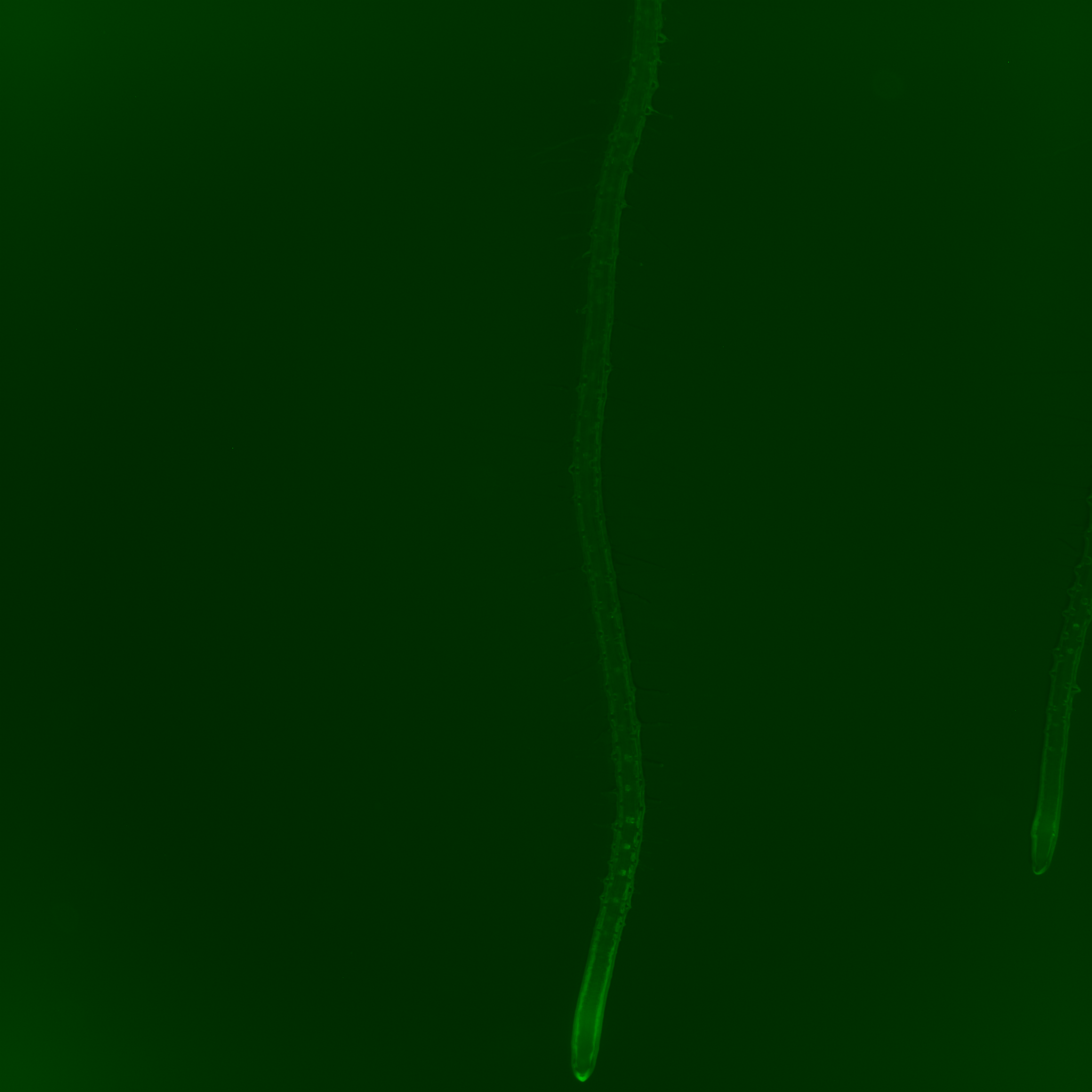

Supplement: Supplementary file 12 — Appendix Figure S2 Source Data [file 44318_2025_614_MOESM12_ESM.zip › Appendix Fig S2/PP2C38 GFP.tif]

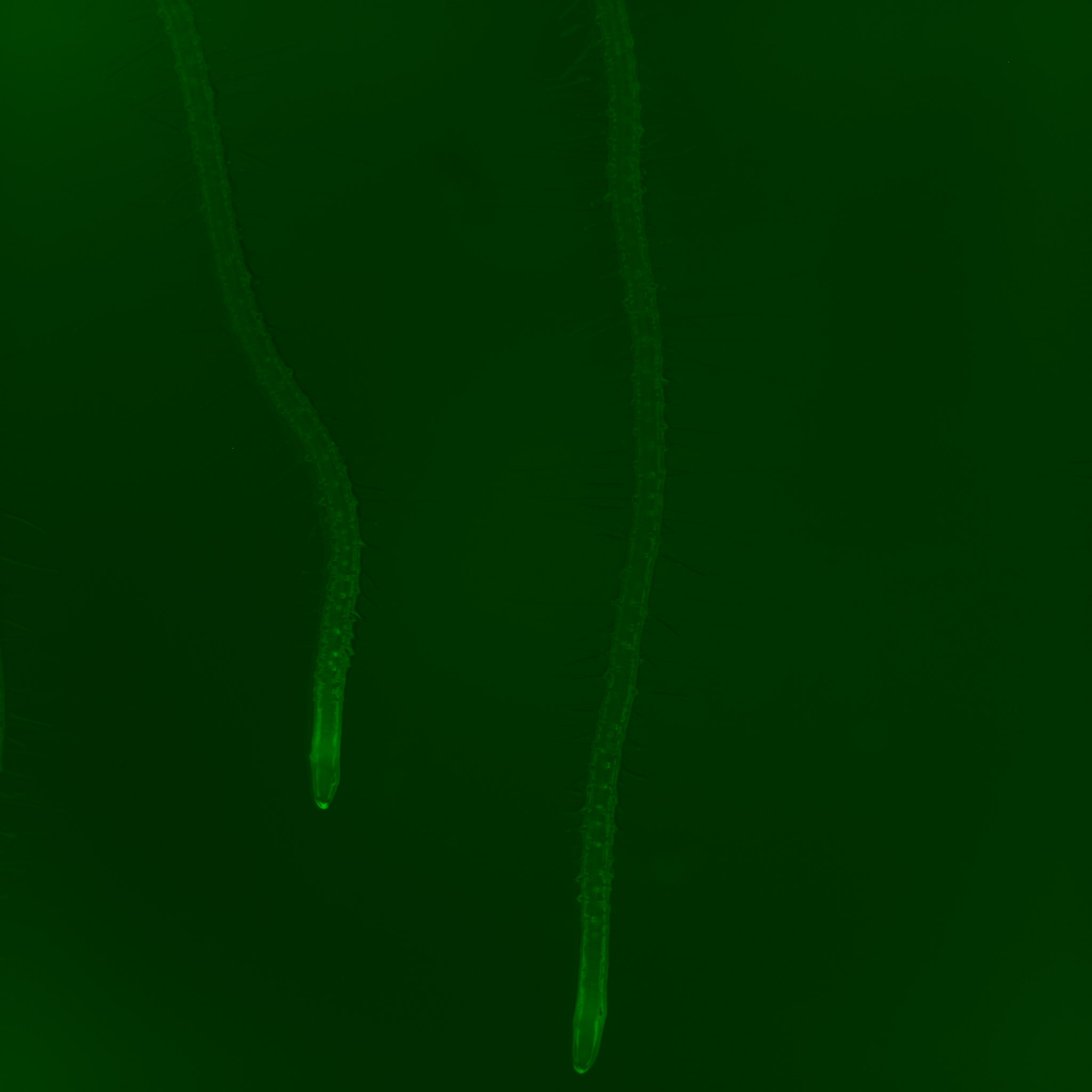

Supplement: Supplementary file 12 — Appendix Figure S2 Source Data [file 44318_2025_614_MOESM12_ESM.zip › Appendix Fig S2/PP2C38 GFP_2.tif]

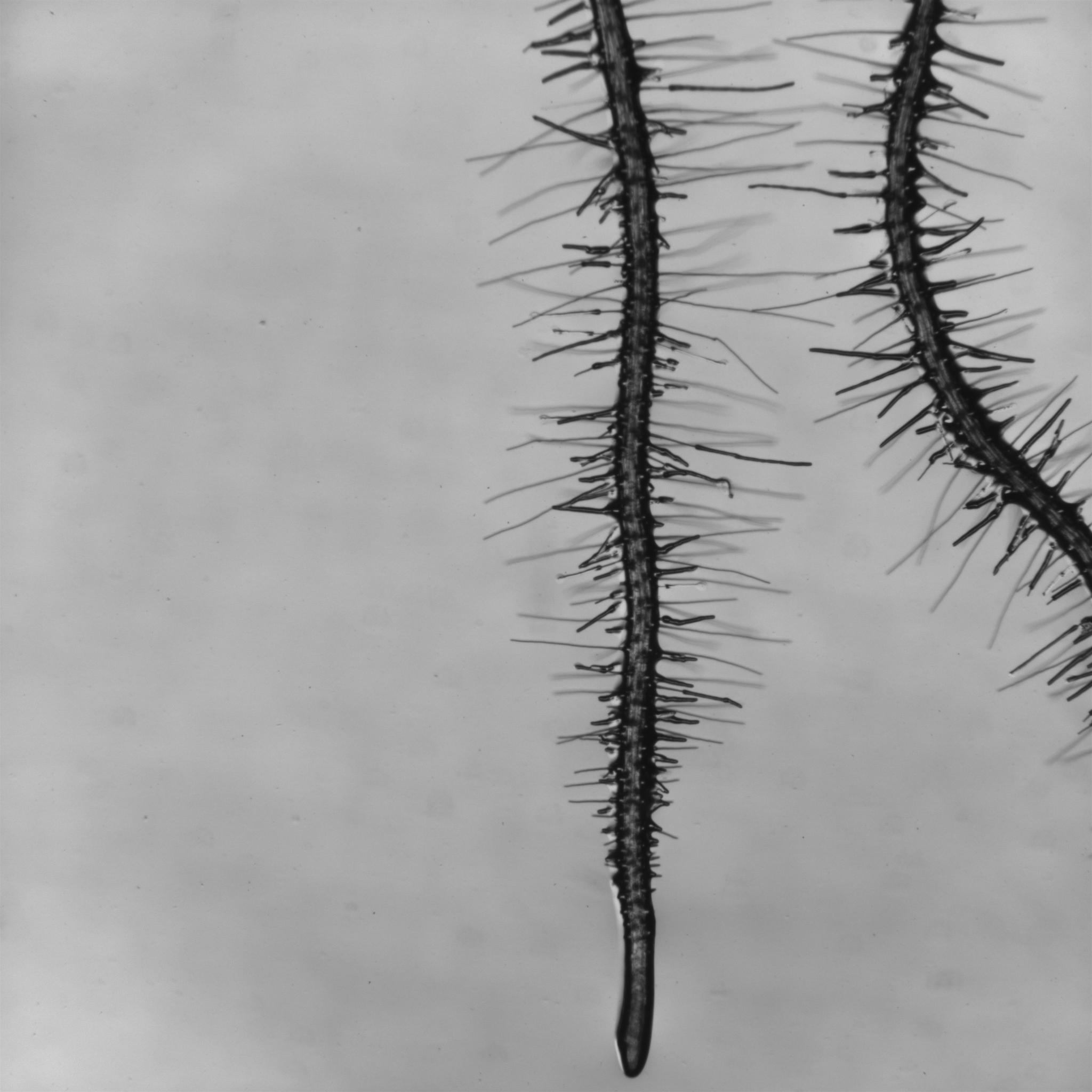

Supplement: Supplementary file 12 — Appendix Figure S2 Source Data [file 44318_2025_614_MOESM12_ESM.zip › Appendix Fig S2/PP2C52 bright_1.tif]

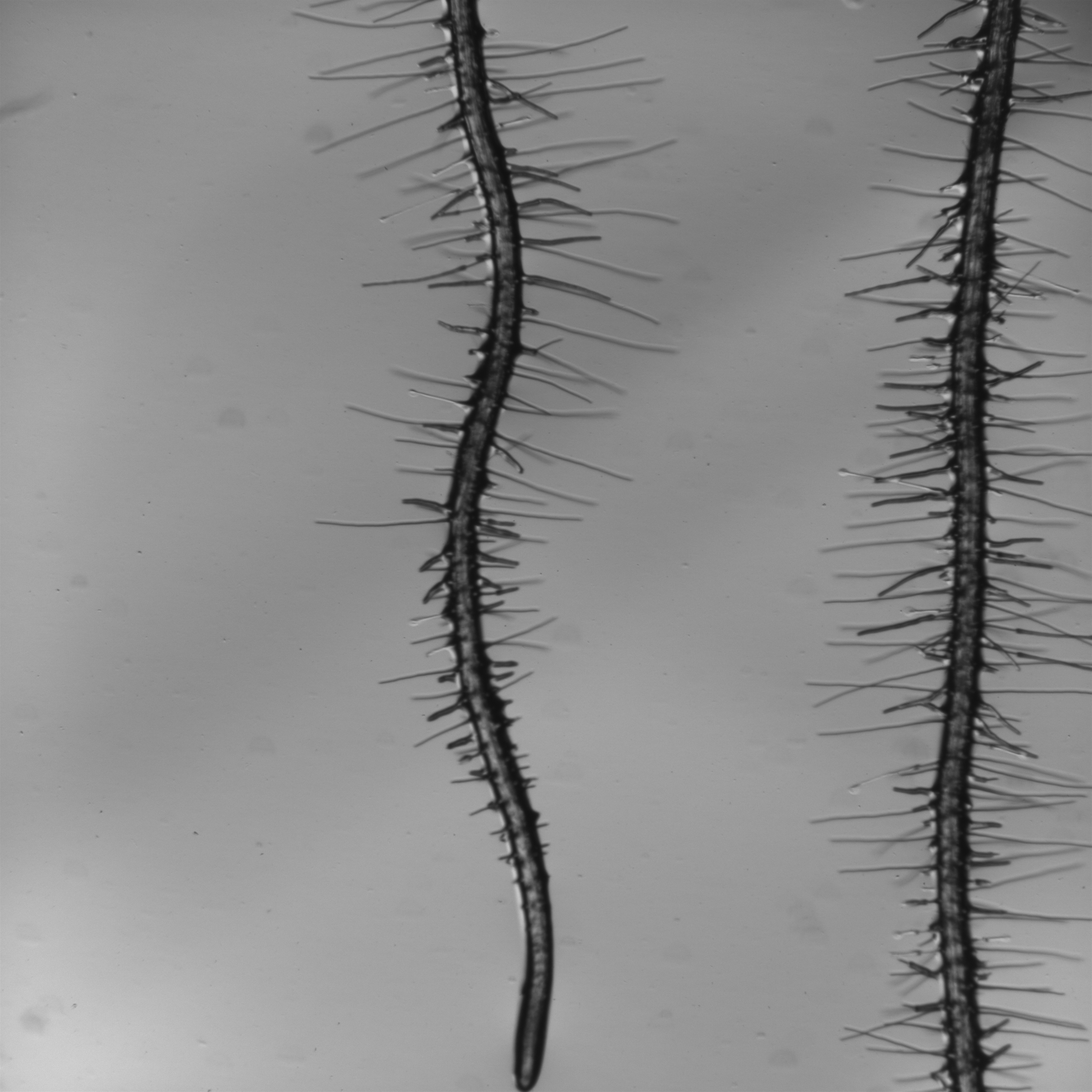

Supplement: Supplementary file 12 — Appendix Figure S2 Source Data [file 44318_2025_614_MOESM12_ESM.zip › Appendix Fig S2/PP2C52 bright_2.tif]

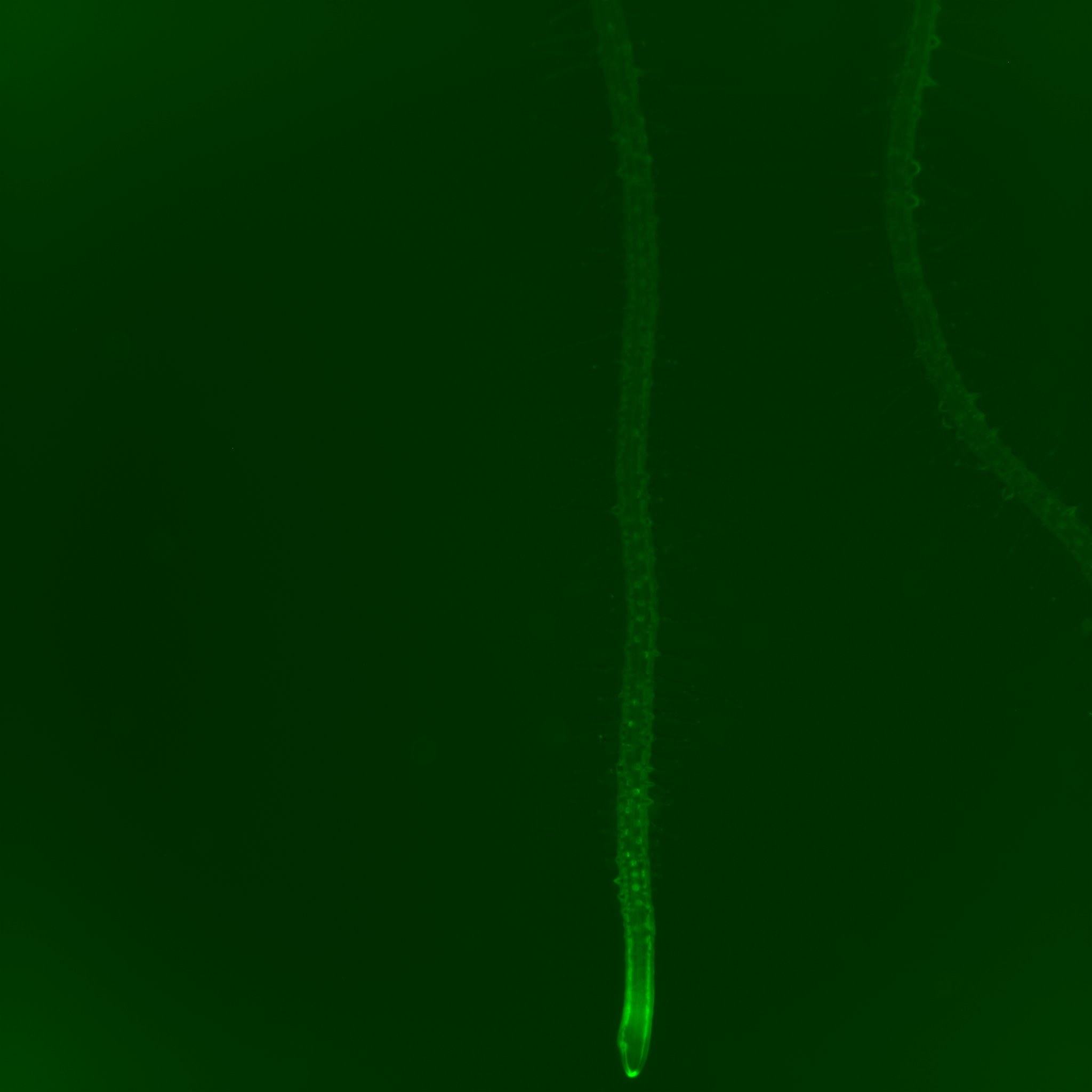

Supplement: Supplementary file 12 — Appendix Figure S2 Source Data [file 44318_2025_614_MOESM12_ESM.zip › Appendix Fig S2/PP2C52 GFP_1.tif]

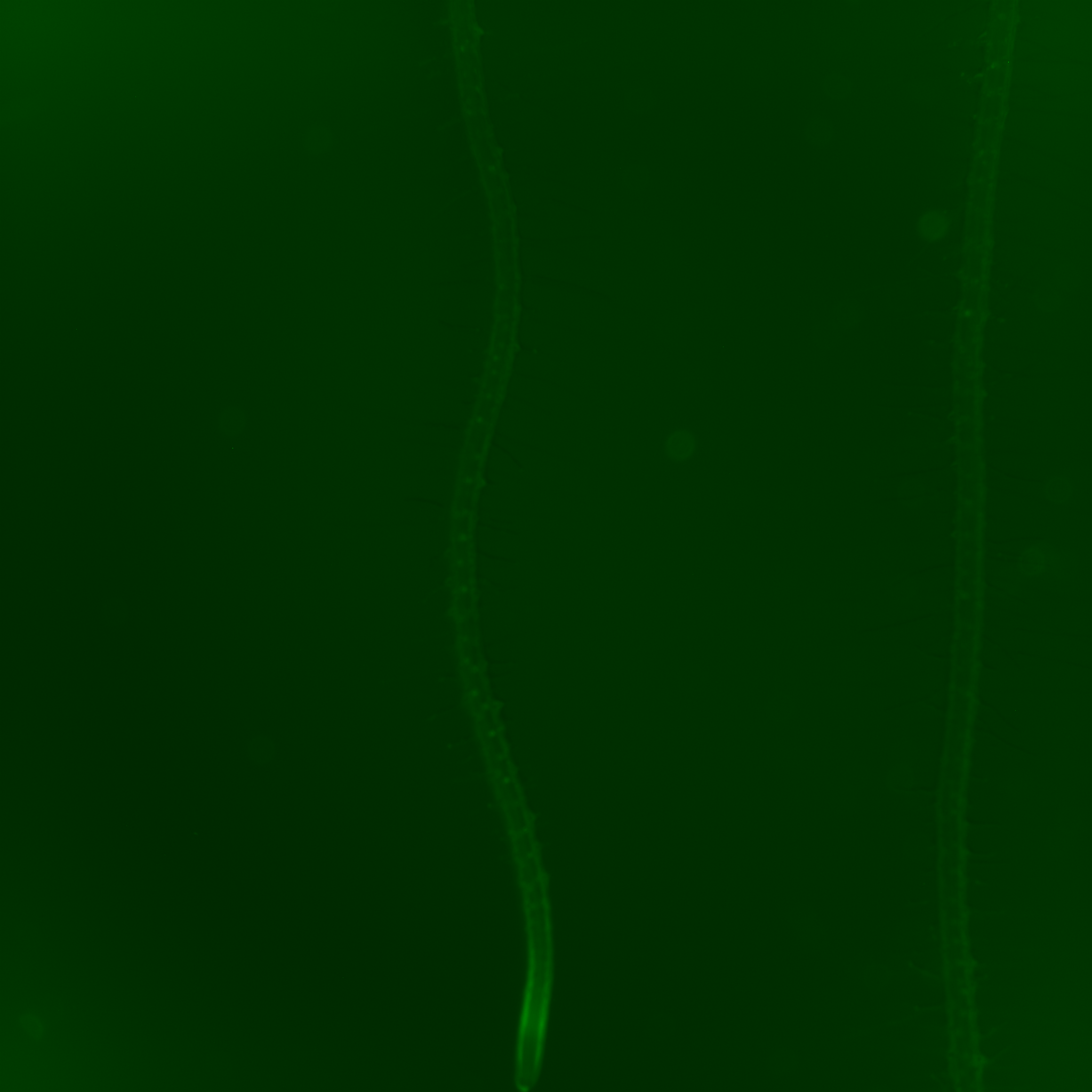

Supplement: Supplementary file 12 — Appendix Figure S2 Source Data [file 44318_2025_614_MOESM12_ESM.zip › Appendix Fig S2/PP2C52 GFP_2.tif]

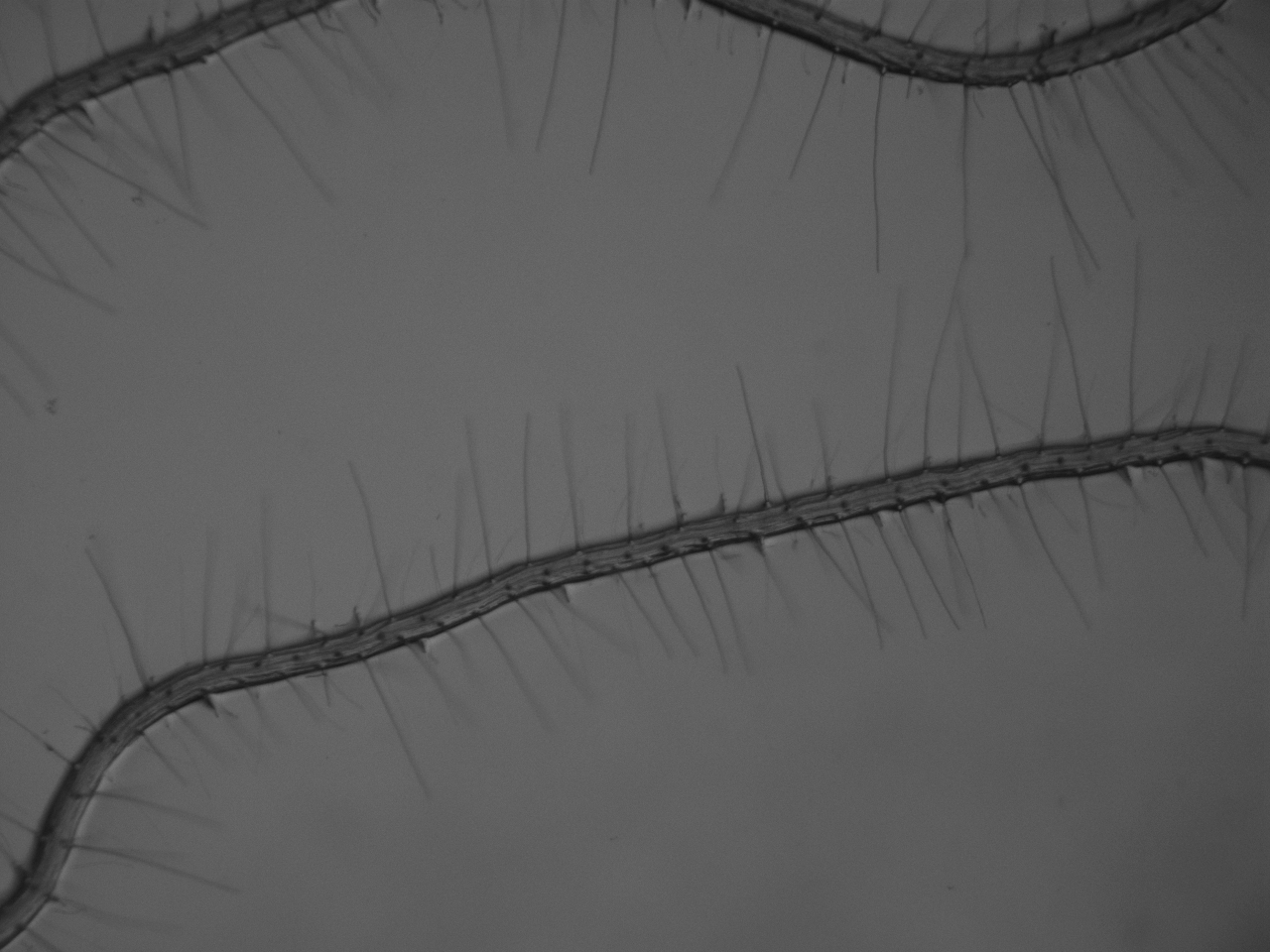

Supplement: Supplementary file 13 — Appendix Figure S3 Source Data [file 44318_2025_614_MOESM13_ESM.zip › Appendix Fig S3/Fig S3E/panel E Col.tif]

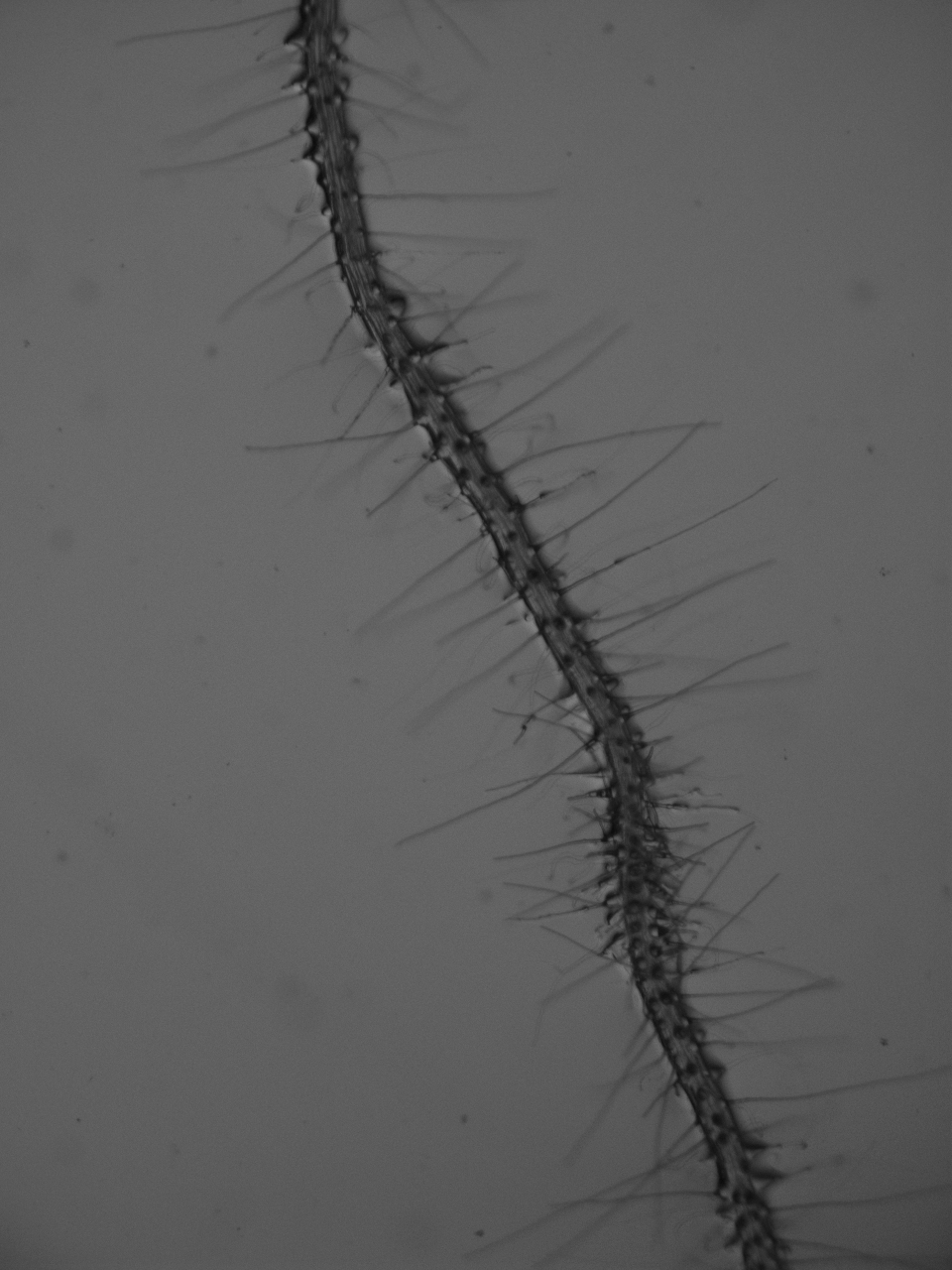

Supplement: Supplementary file 13 — Appendix Figure S3 Source Data [file 44318_2025_614_MOESM13_ESM.zip › Appendix Fig S3/Fig S3E/panel E T2_1.tif]

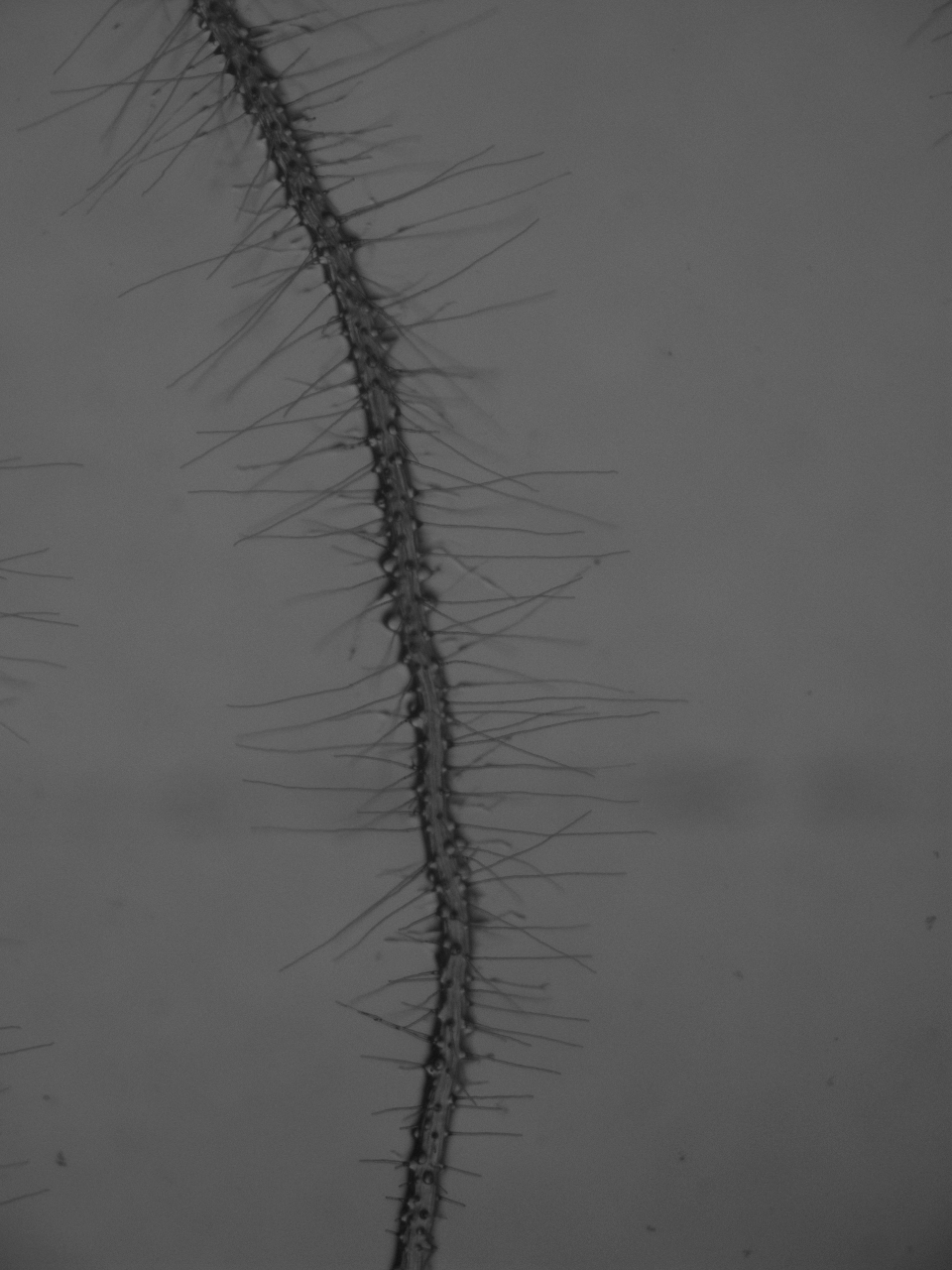

Supplement: Supplementary file 13 — Appendix Figure S3 Source Data [file 44318_2025_614_MOESM13_ESM.zip › Appendix Fig S3/Fig S3E/panel E T2_2.tif]

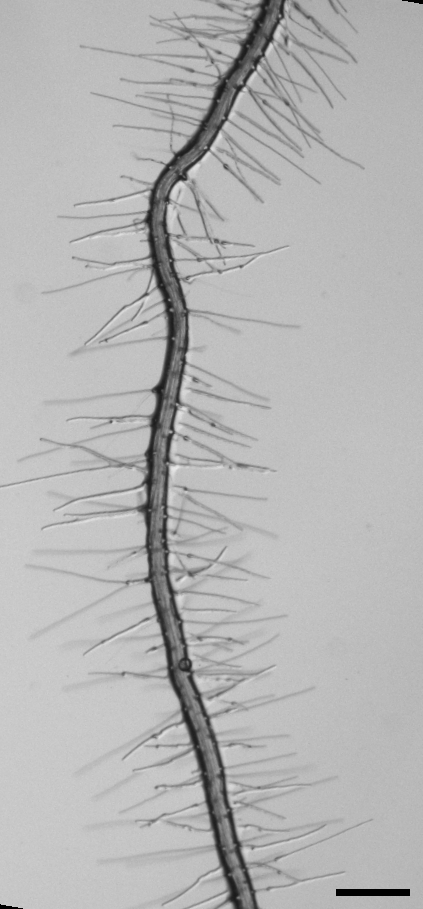

Supplement: Supplementary file 13 — Appendix Figure S3 Source Data [file 44318_2025_614_MOESM13_ESM.zip › Appendix Fig S3/Fig. S3A/Col.png]

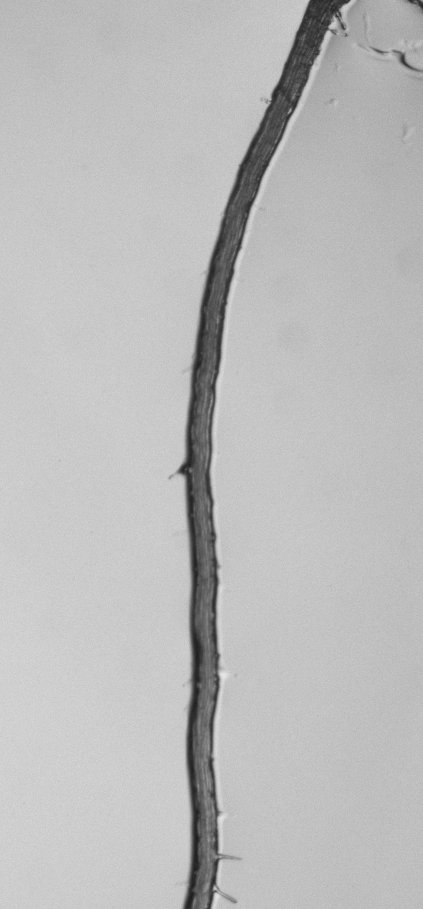

Supplement: Supplementary file 13 — Appendix Figure S3 Source Data [file 44318_2025_614_MOESM13_ESM.zip › Appendix Fig S3/Fig. S3A/fer-4 rol23 pch2 pch3.tif]

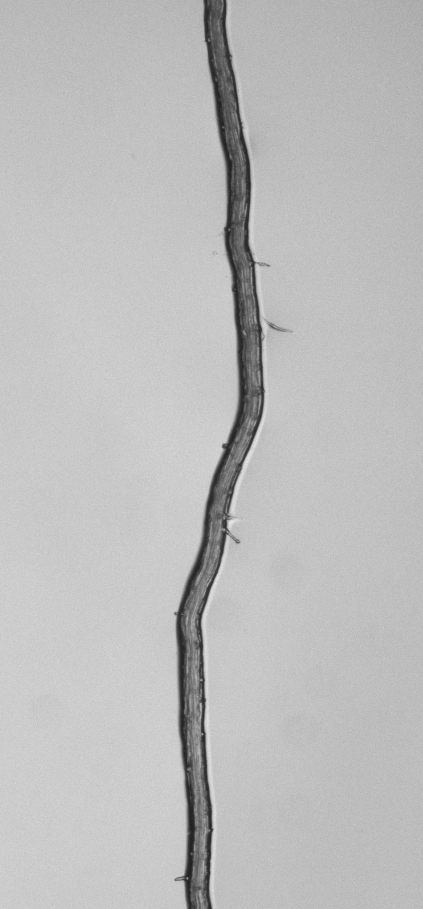

Supplement: Supplementary file 13 — Appendix Figure S3 Source Data [file 44318_2025_614_MOESM13_ESM.zip › Appendix Fig S3/Fig. S3A/fer-4 rol23.tif]

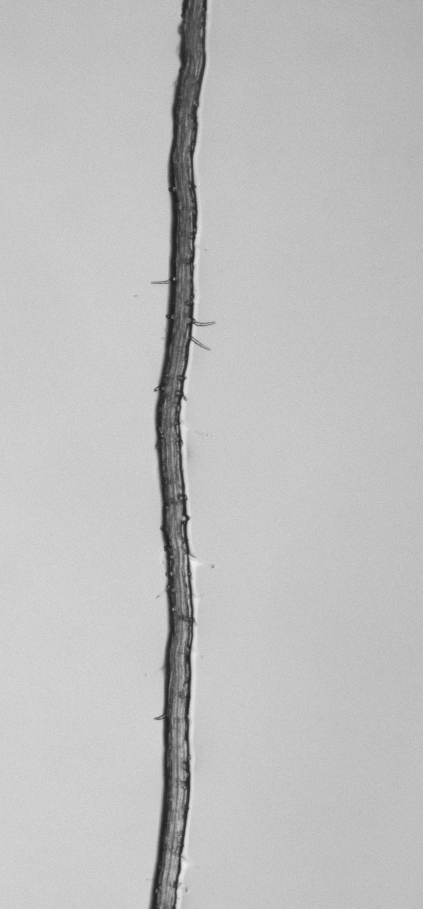

Supplement: Supplementary file 13 — Appendix Figure S3 Source Data [file 44318_2025_614_MOESM13_ESM.zip › Appendix Fig S3/Fig. S3A/fer-4.tif]

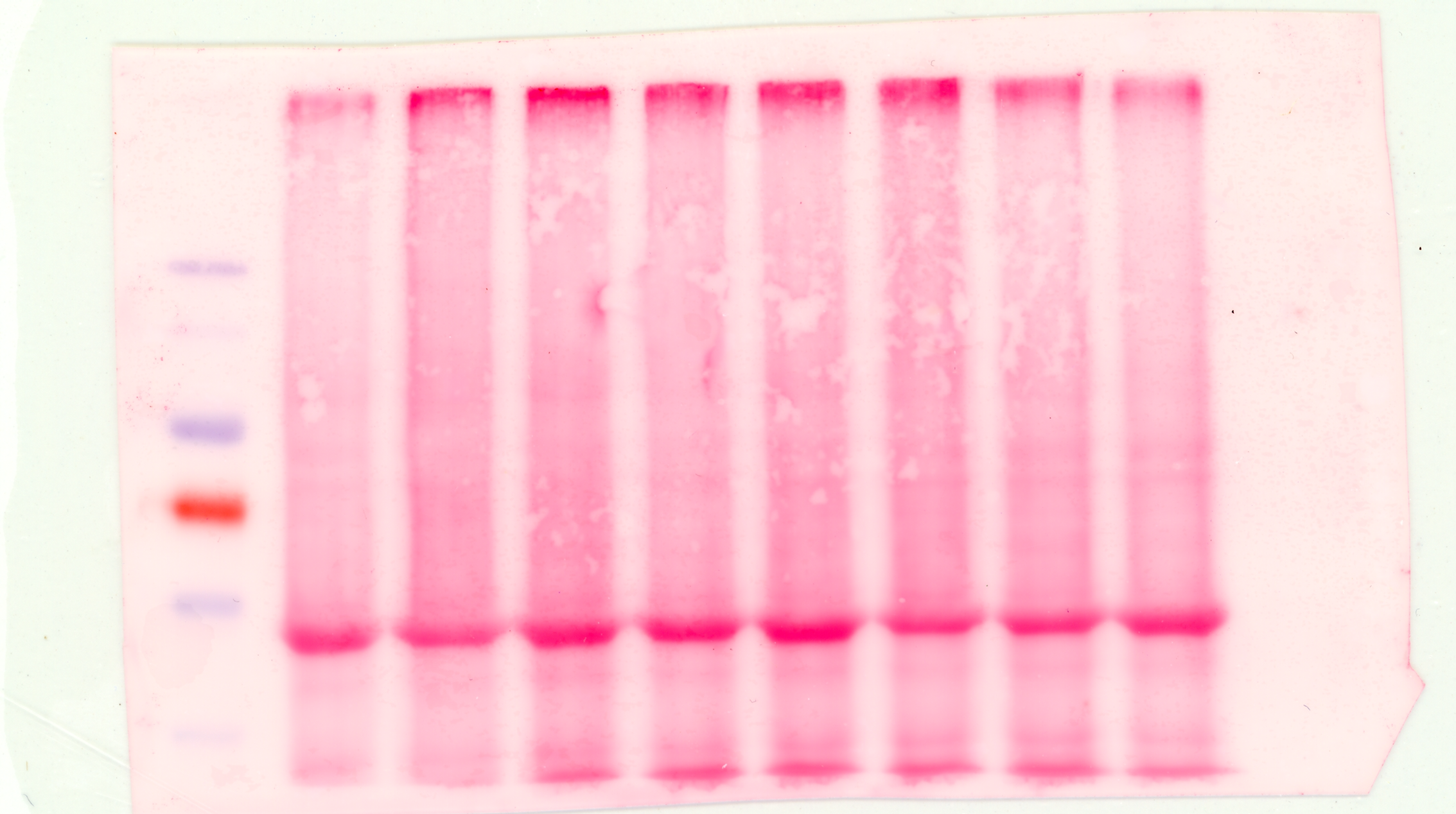

Supplement: Supplementary file 13 — Appendix Figure S3 Source Data [file 44318_2025_614_MOESM13_ESM.zip › Appendix Fig S3/Fig. S3D/Ponceu004.tif]

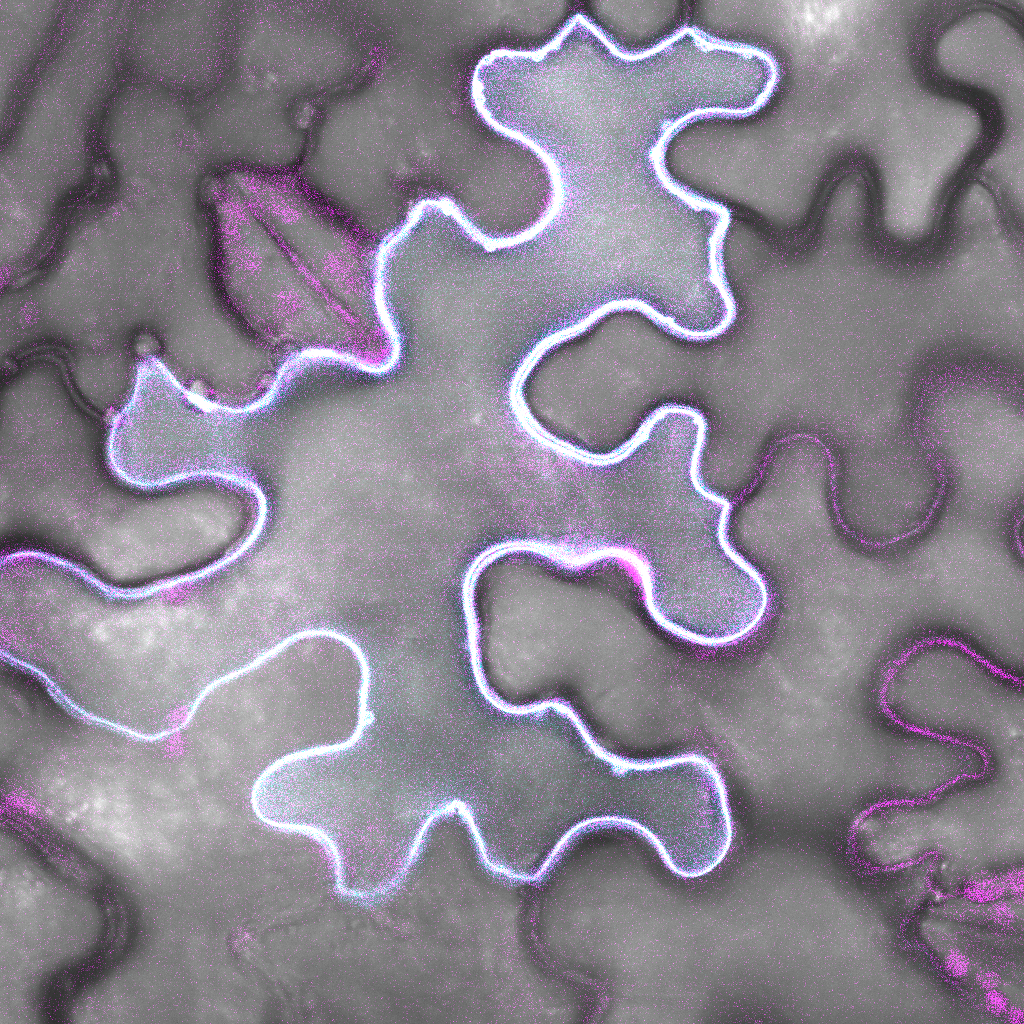

Supplement: Supplementary file 14 — Appendix Figure S4 Source Data [file 44318_2025_614_MOESM14_ESM.zip › Appendix Fig S4/Suppl Fig S4B/FERC + PP2C12N merge.tif]

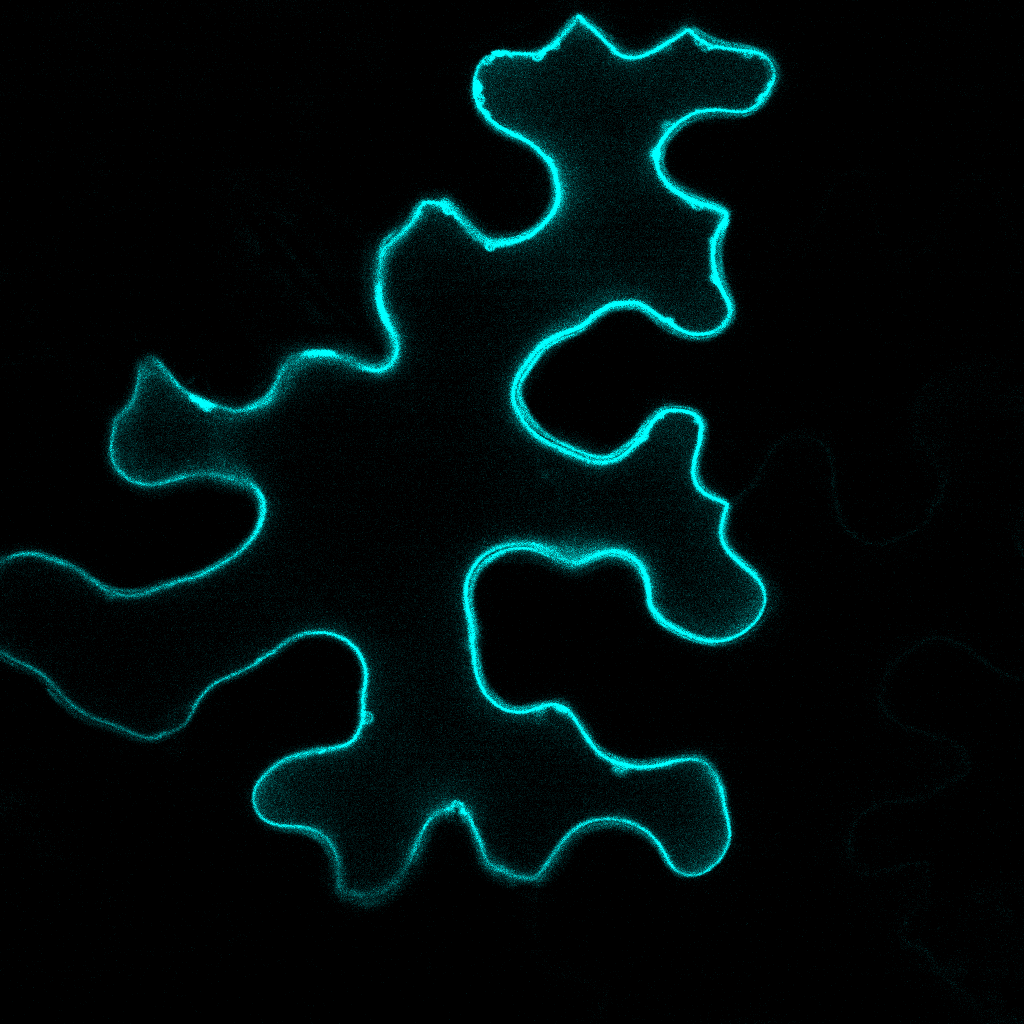

Supplement: Supplementary file 14 — Appendix Figure S4 Source Data [file 44318_2025_614_MOESM14_ESM.zip › Appendix Fig S4/Suppl Fig S4B/FERC + PP2C12N mVENUS.tif]

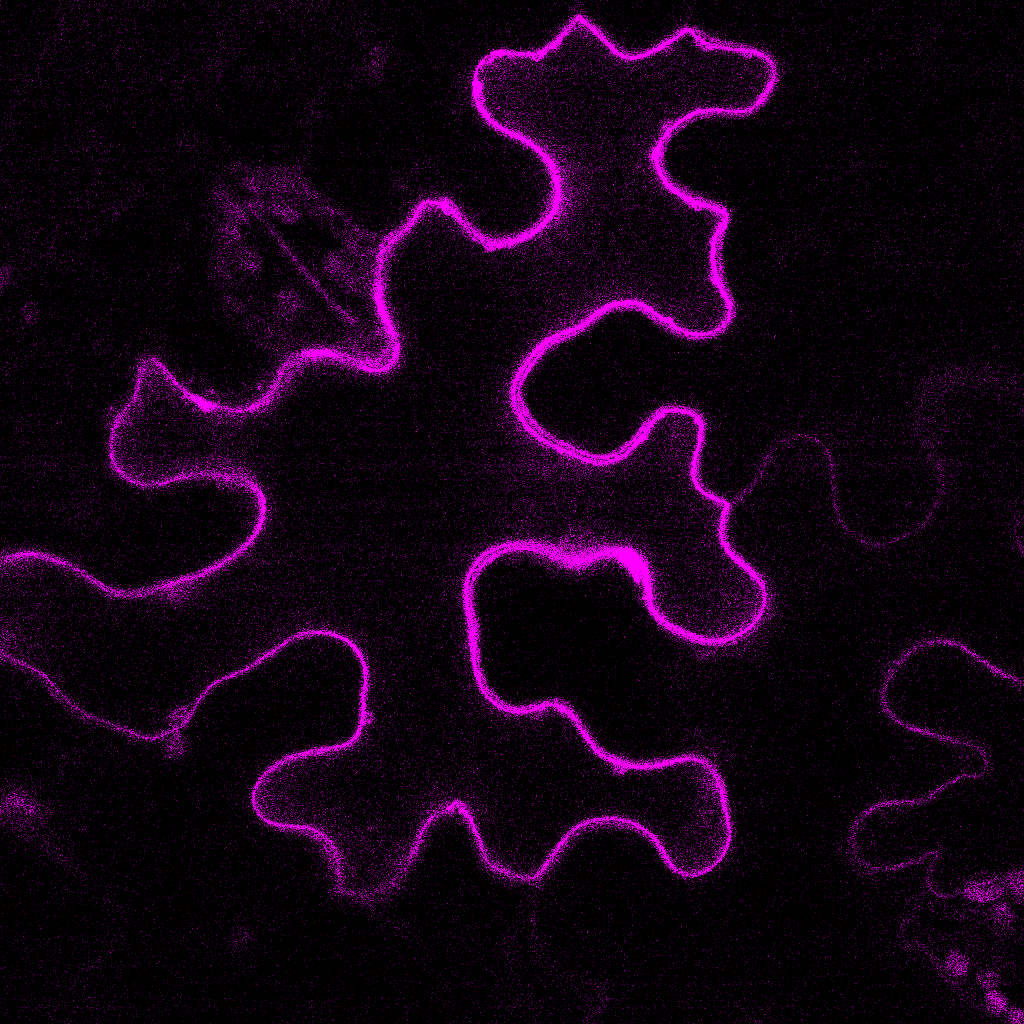

Supplement: Supplementary file 14 — Appendix Figure S4 Source Data [file 44318_2025_614_MOESM14_ESM.zip › Appendix Fig S4/Suppl Fig S4B/FERC + PP2C12N PM-mRFP.tif]

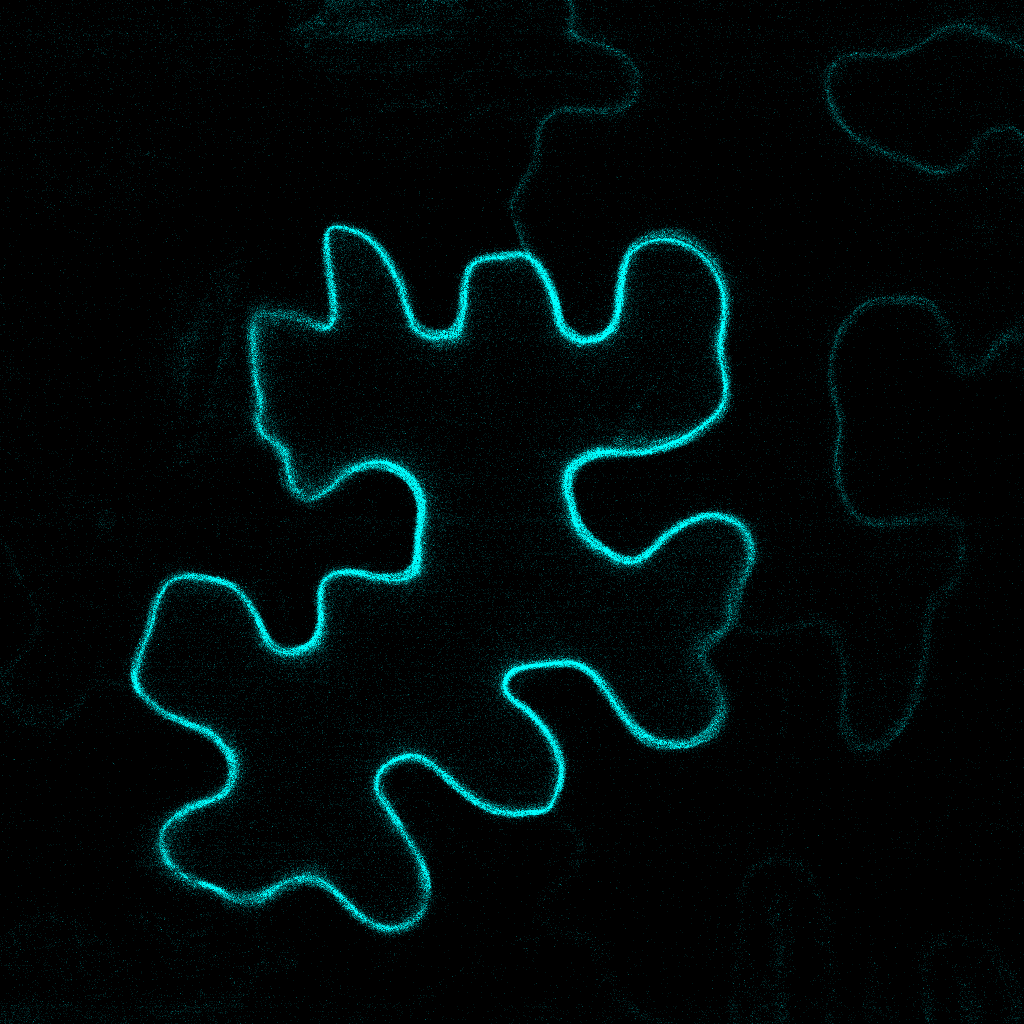

Supplement: Supplementary file 14 — Appendix Figure S4 Source Data [file 44318_2025_614_MOESM14_ESM.zip › Appendix Fig S4/Suppl Fig S4B/FERN + PP2C12C merge mVENUS.tif]

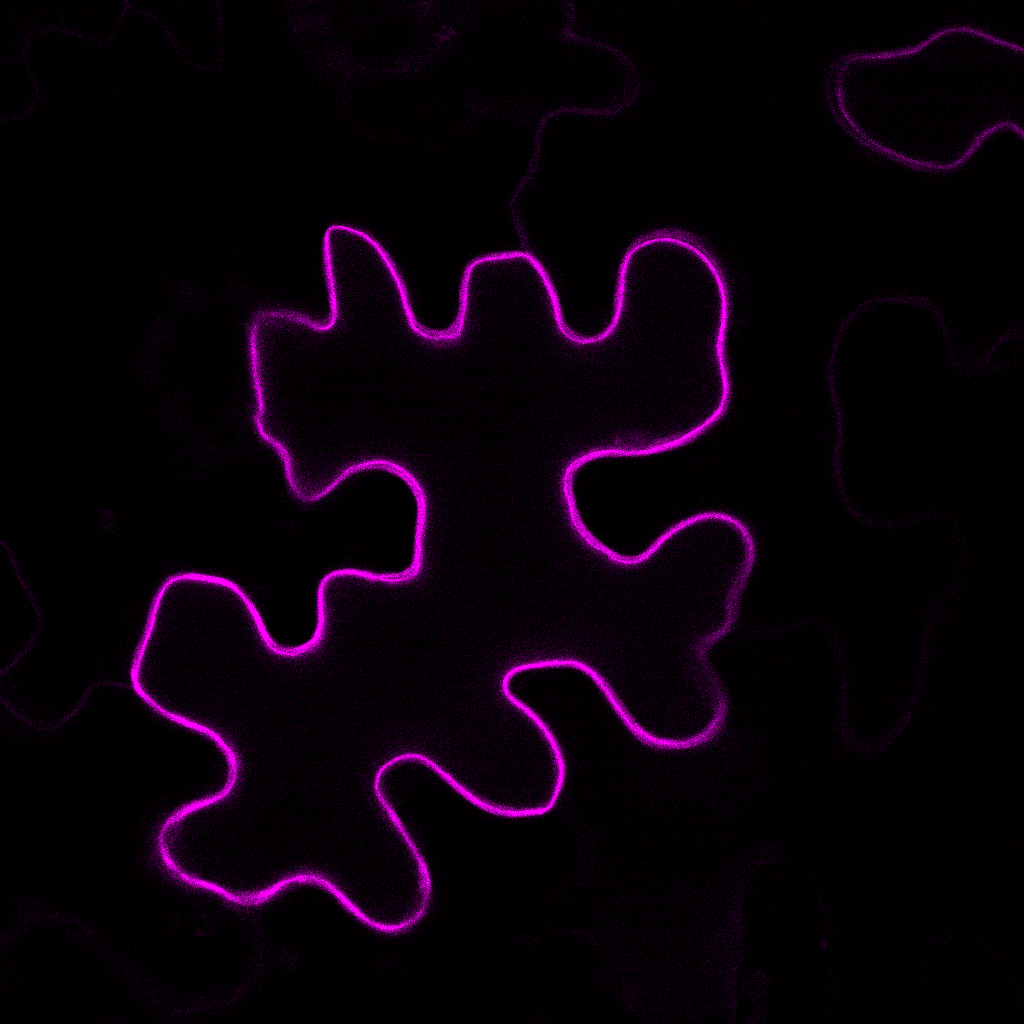

Supplement: Supplementary file 14 — Appendix Figure S4 Source Data [file 44318_2025_614_MOESM14_ESM.zip › Appendix Fig S4/Suppl Fig S4B/FERN + PP2C12C merge PM-mRFP.tif]

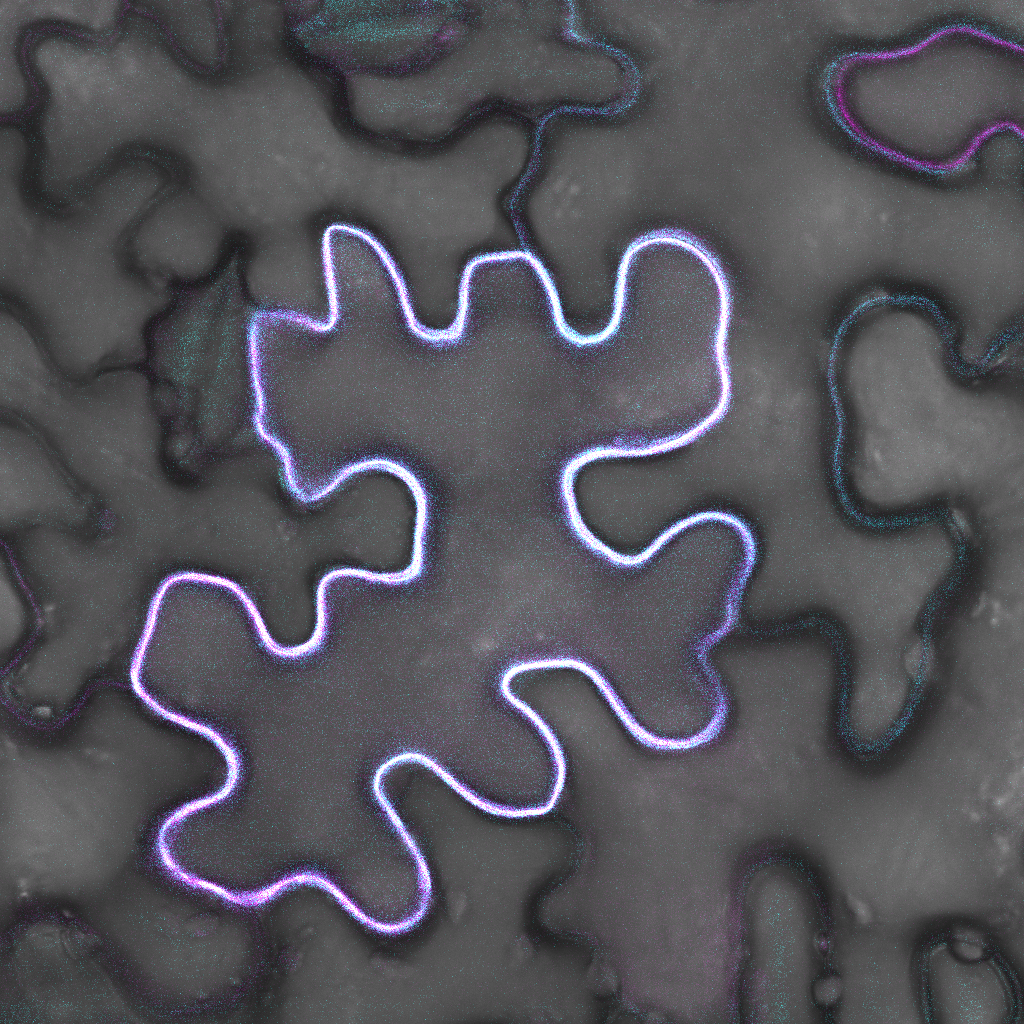

Supplement: Supplementary file 14 — Appendix Figure S4 Source Data [file 44318_2025_614_MOESM14_ESM.zip › Appendix Fig S4/Suppl Fig S4B/FERN + PP2C12C merge.tif]

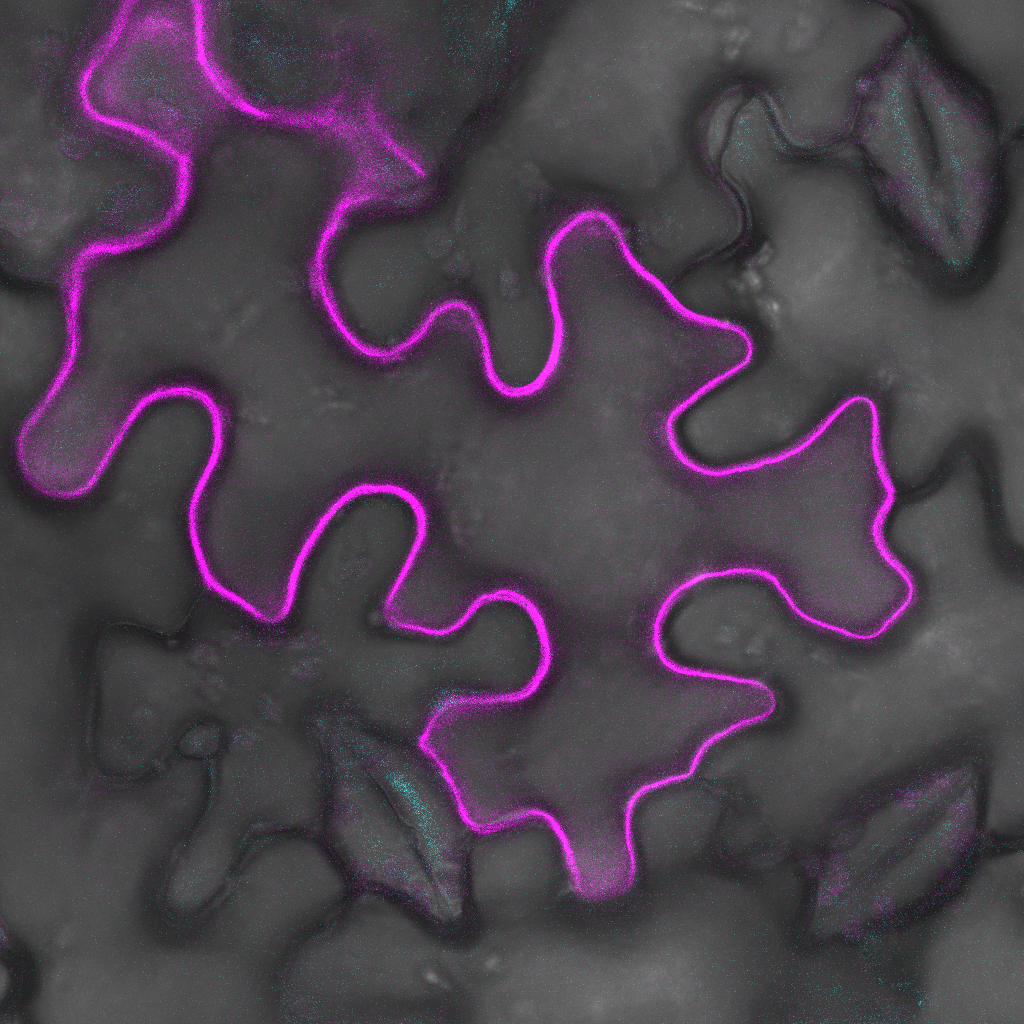

Supplement: Supplementary file 14 — Appendix Figure S4 Source Data [file 44318_2025_614_MOESM14_ESM.zip › Appendix Fig S4/Suppl Fig S4B/Lti6bC + PP2C12N merge.tif]

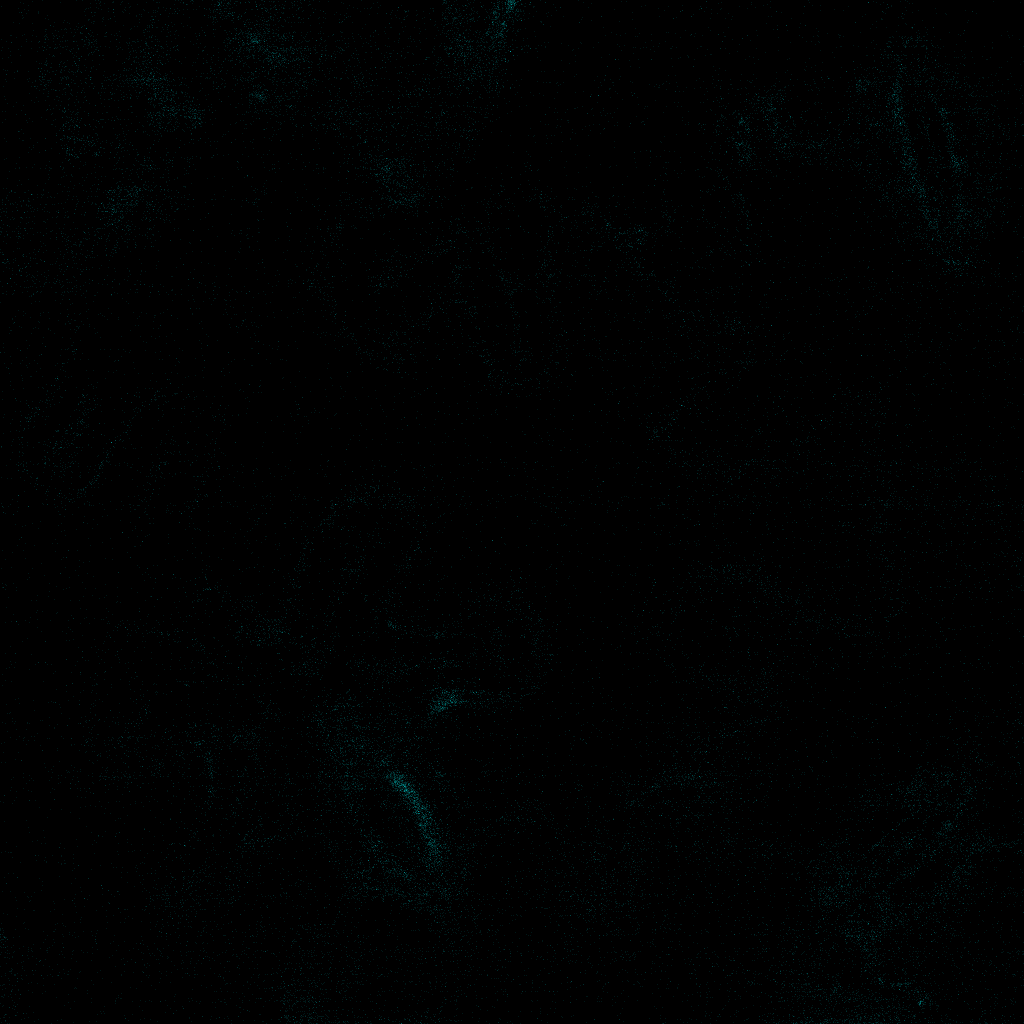

Supplement: Supplementary file 14 — Appendix Figure S4 Source Data [file 44318_2025_614_MOESM14_ESM.zip › Appendix Fig S4/Suppl Fig S4B/Lti6bC + PP2C12N mVenus.tif]

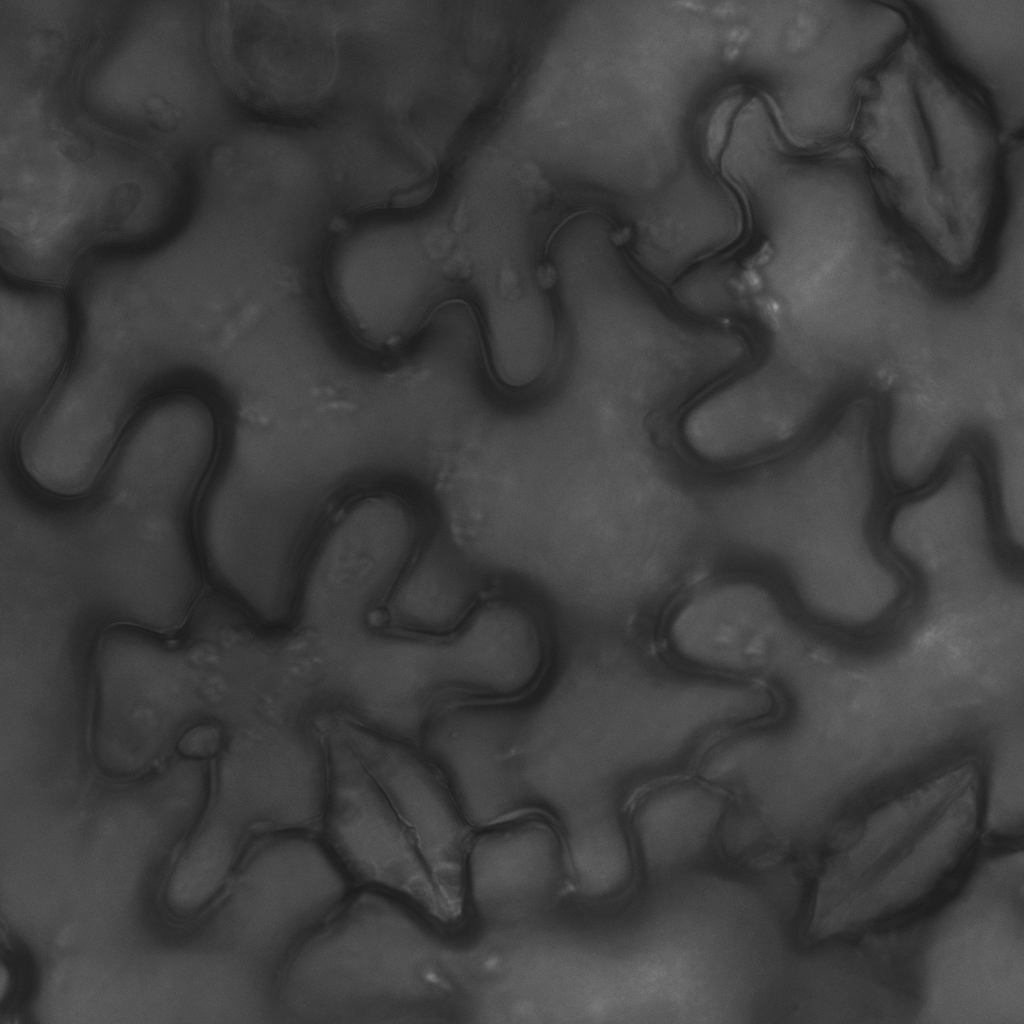

Supplement: Supplementary file 14 — Appendix Figure S4 Source Data [file 44318_2025_614_MOESM14_ESM.zip › Appendix Fig S4/Suppl Fig S4B/Lti6bC + PP2C12N PM-mRFP.tif]

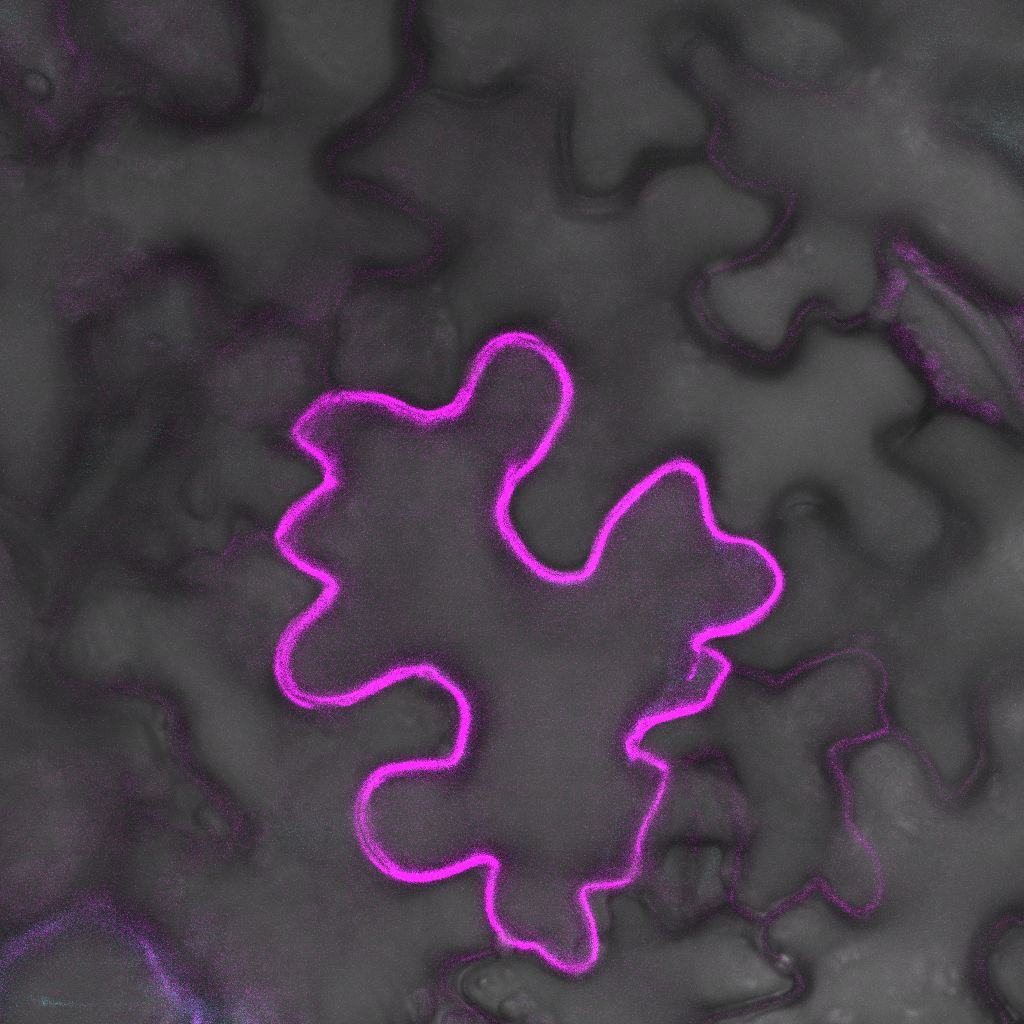

Supplement: Supplementary file 14 — Appendix Figure S4 Source Data [file 44318_2025_614_MOESM14_ESM.zip › Appendix Fig S4/Suppl Fig S4B/Lti6bN + PP2C12C merge.tif]

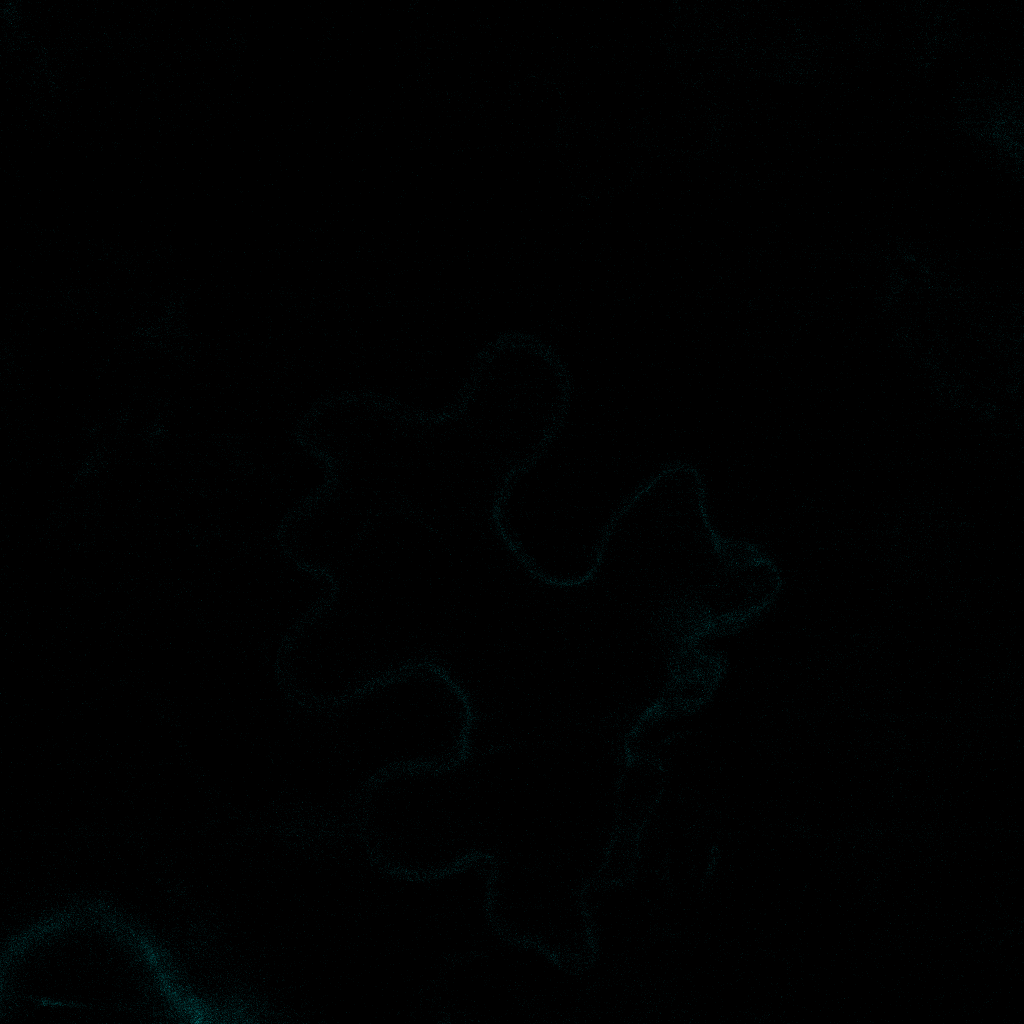

Supplement: Supplementary file 14 — Appendix Figure S4 Source Data [file 44318_2025_614_MOESM14_ESM.zip › Appendix Fig S4/Suppl Fig S4B/Lti6bN + PP2C12C mVENUS.tif]

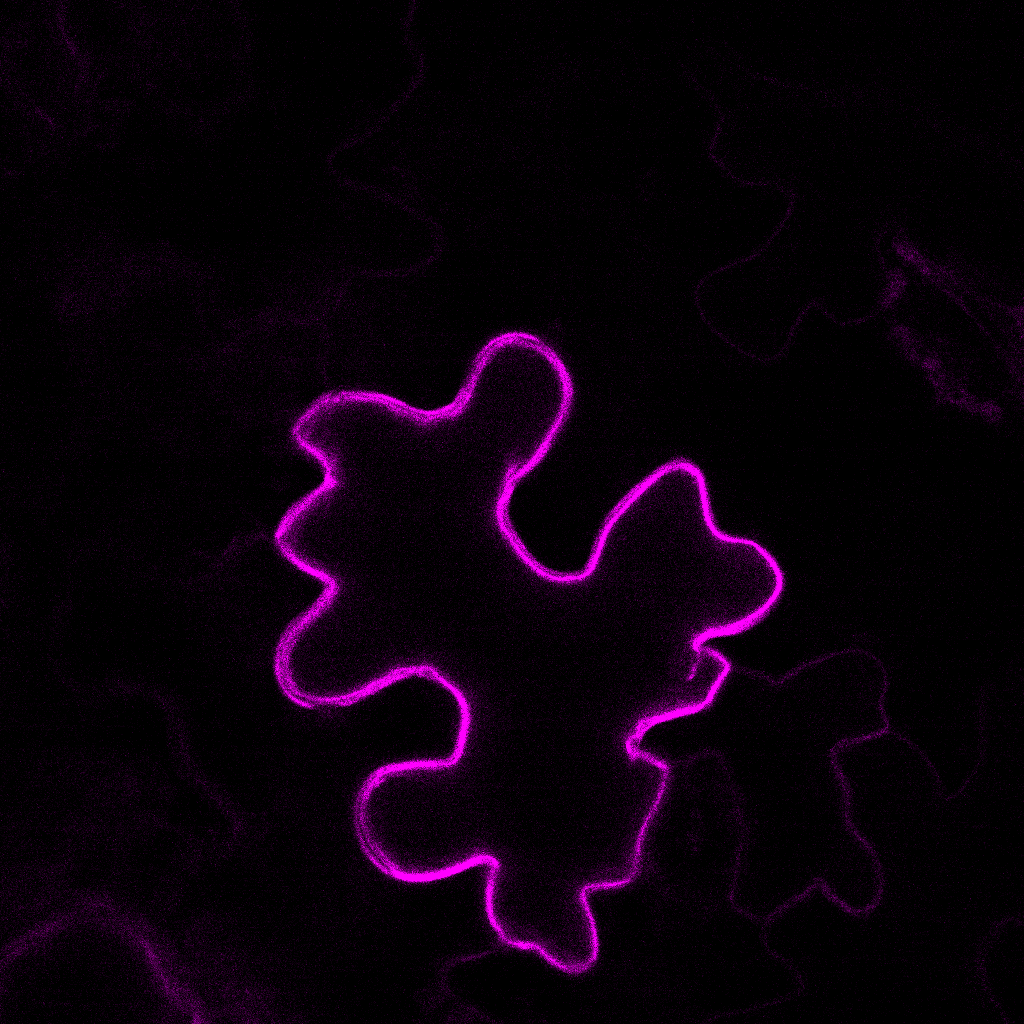

Supplement: Supplementary file 14 — Appendix Figure S4 Source Data [file 44318_2025_614_MOESM14_ESM.zip › Appendix Fig S4/Suppl Fig S4B/Lti6bN + PP2C12C PM-mRFP.tif]

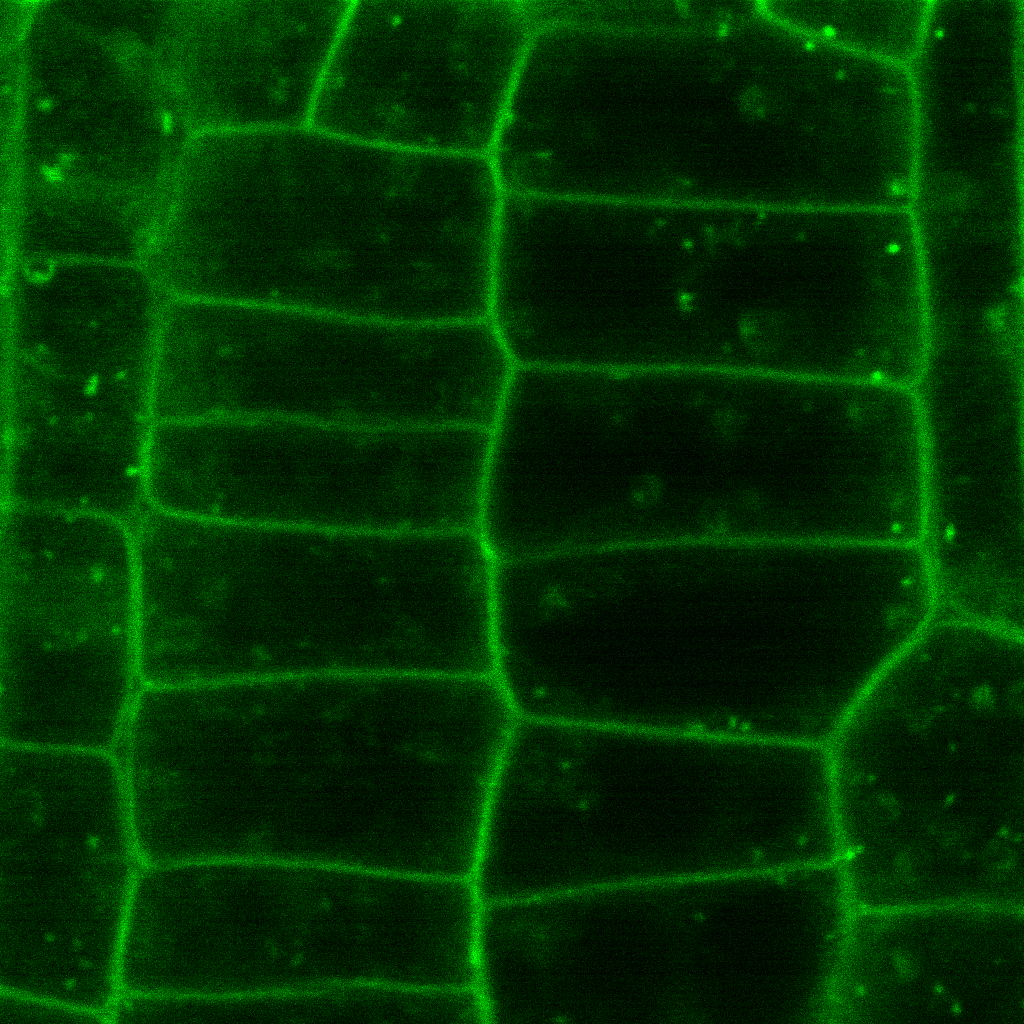

Supplement: Supplementary file 16 — Appendix Figure S6 Source Data [file 44318_2025_614_MOESM16_ESM.zip › Appendix Fig S6/fer-4 FER-GFP mock/Image001_ch00.tif]

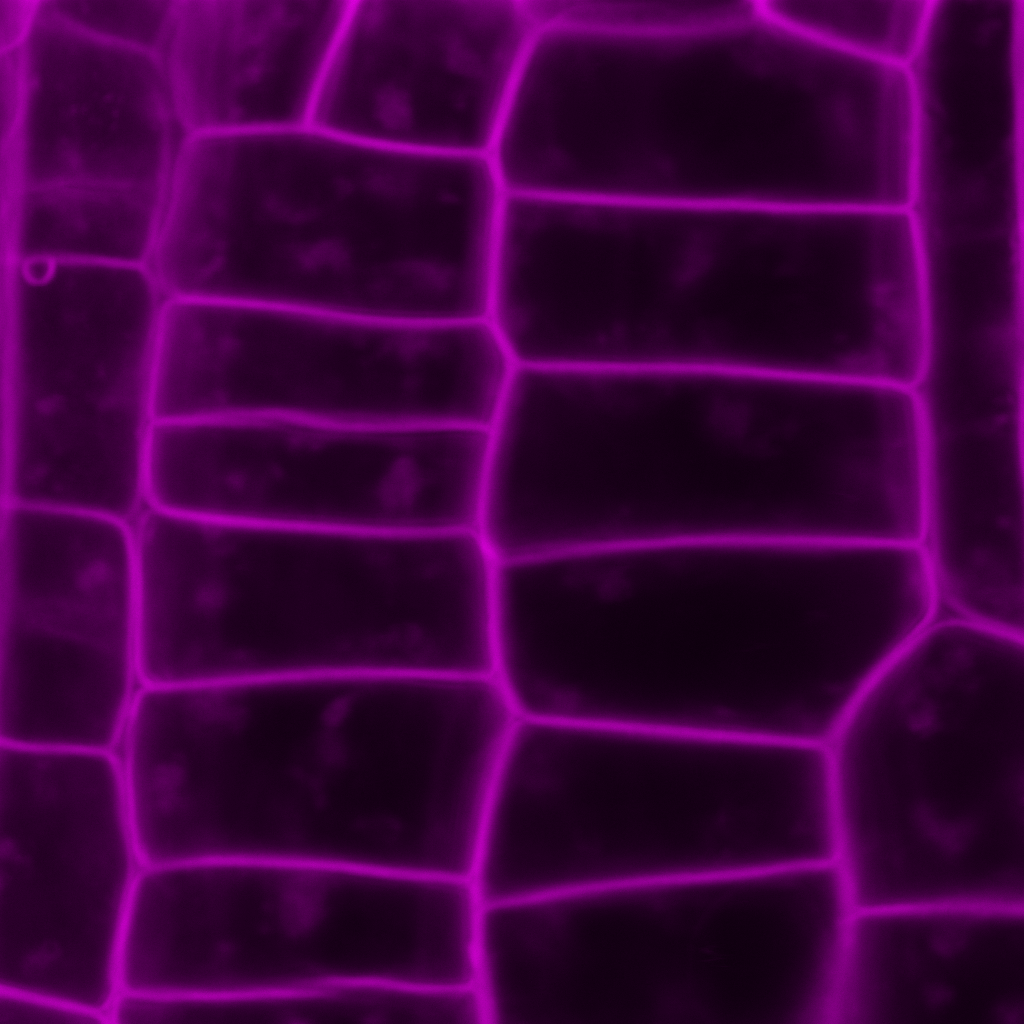

Supplement: Supplementary file 16 — Appendix Figure S6 Source Data [file 44318_2025_614_MOESM16_ESM.zip › Appendix Fig S6/fer-4 FER-GFP mock/Image001_ch01.tif]

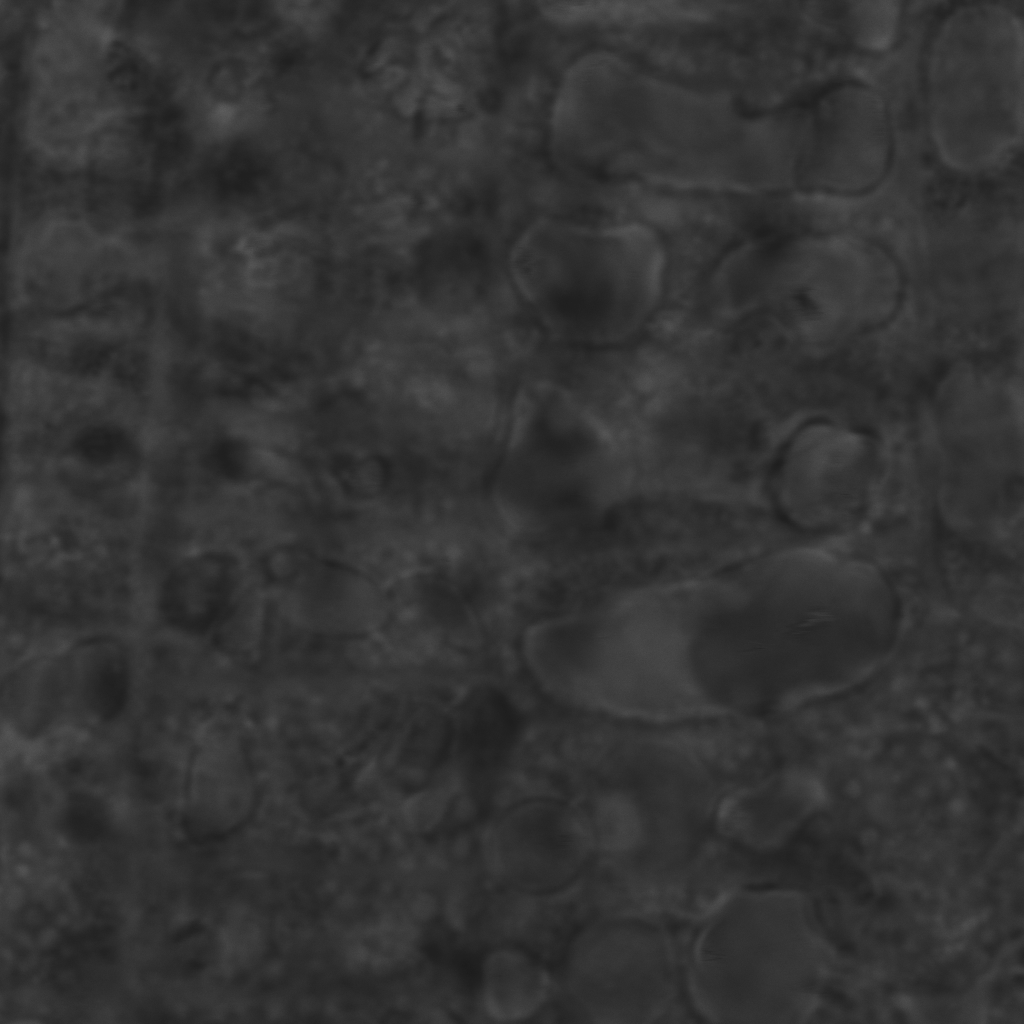

Supplement: Supplementary file 16 — Appendix Figure S6 Source Data [file 44318_2025_614_MOESM16_ESM.zip › Appendix Fig S6/fer-4 FER-GFP mock/Image001_ch02.tif]

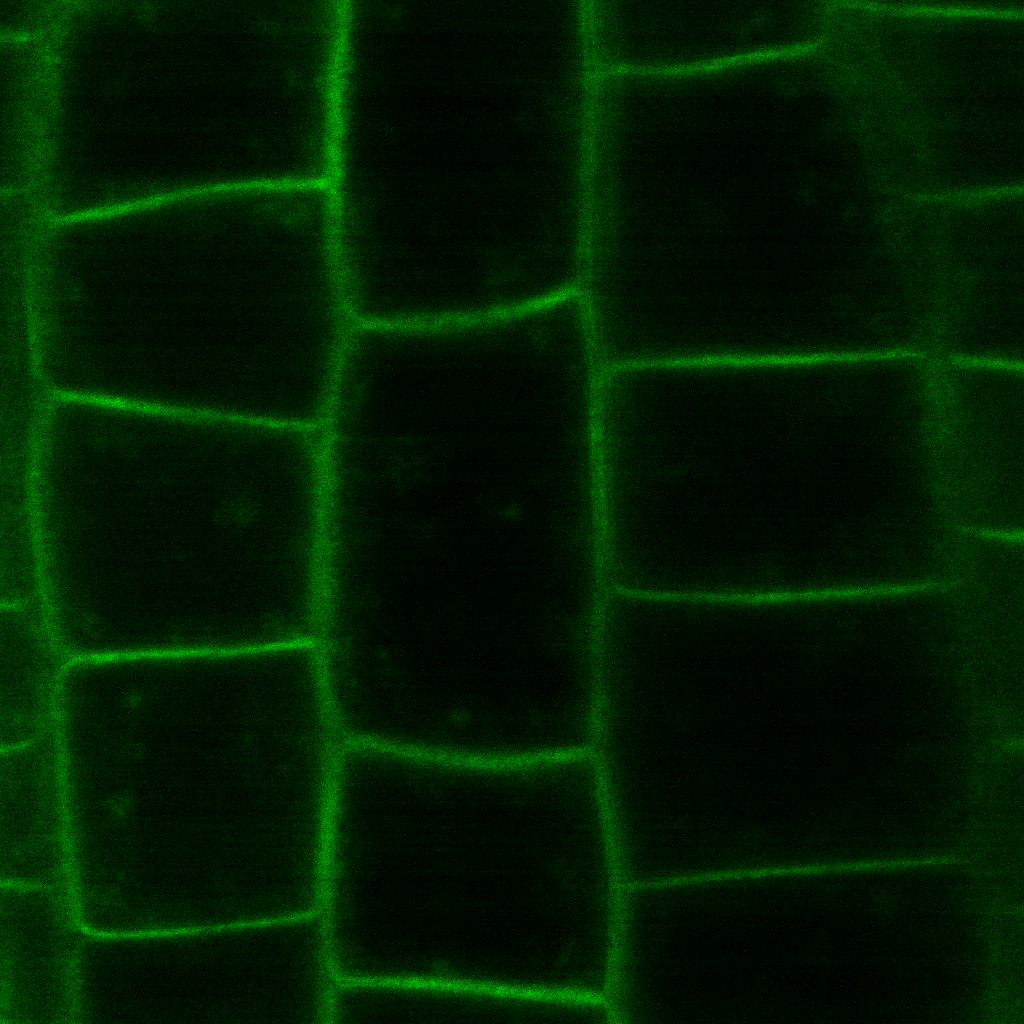

Supplement: Supplementary file 16 — Appendix Figure S6 Source Data [file 44318_2025_614_MOESM16_ESM.zip › Appendix Fig S6/fer-4 FER-GFP RALF1/Image012_ch00.tif]

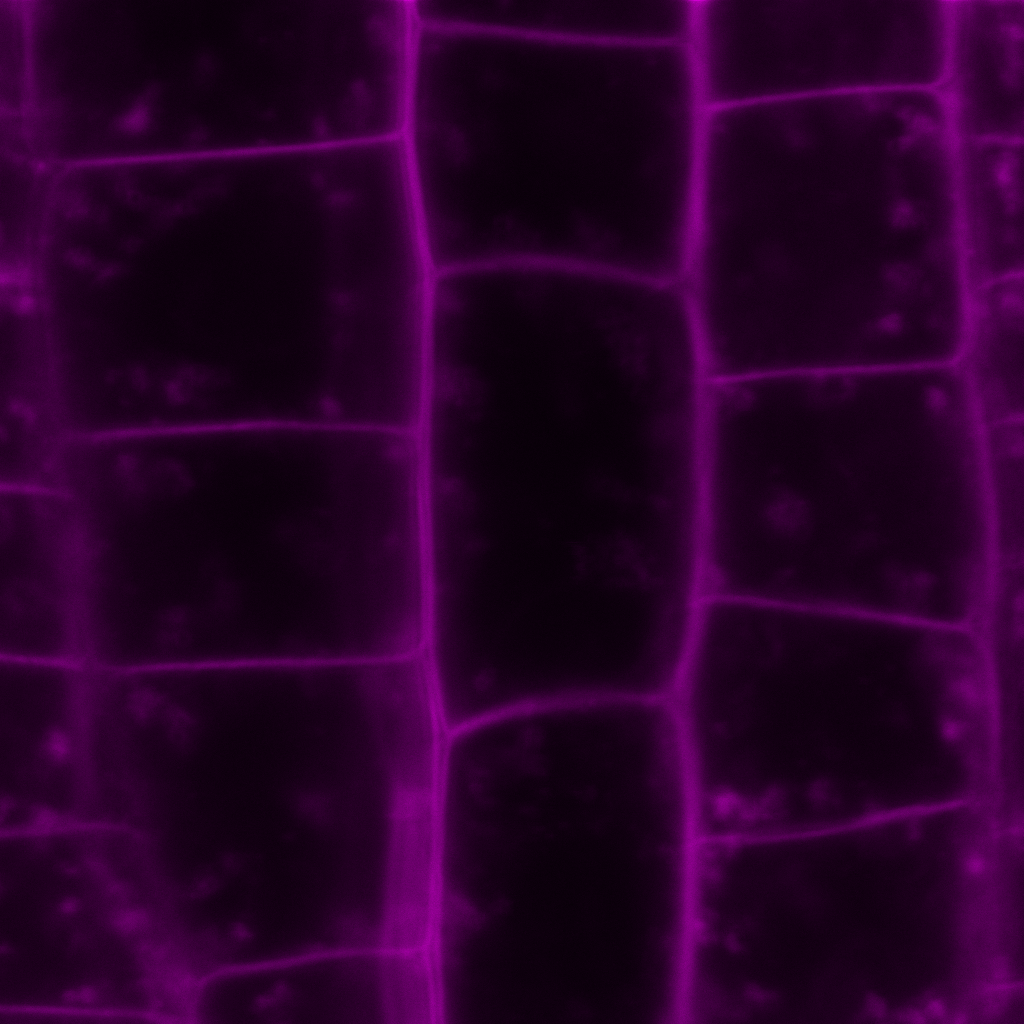

Supplement: Supplementary file 16 — Appendix Figure S6 Source Data [file 44318_2025_614_MOESM16_ESM.zip › Appendix Fig S6/fer-4 FER-GFP RALF1/Image012_ch01.tif]

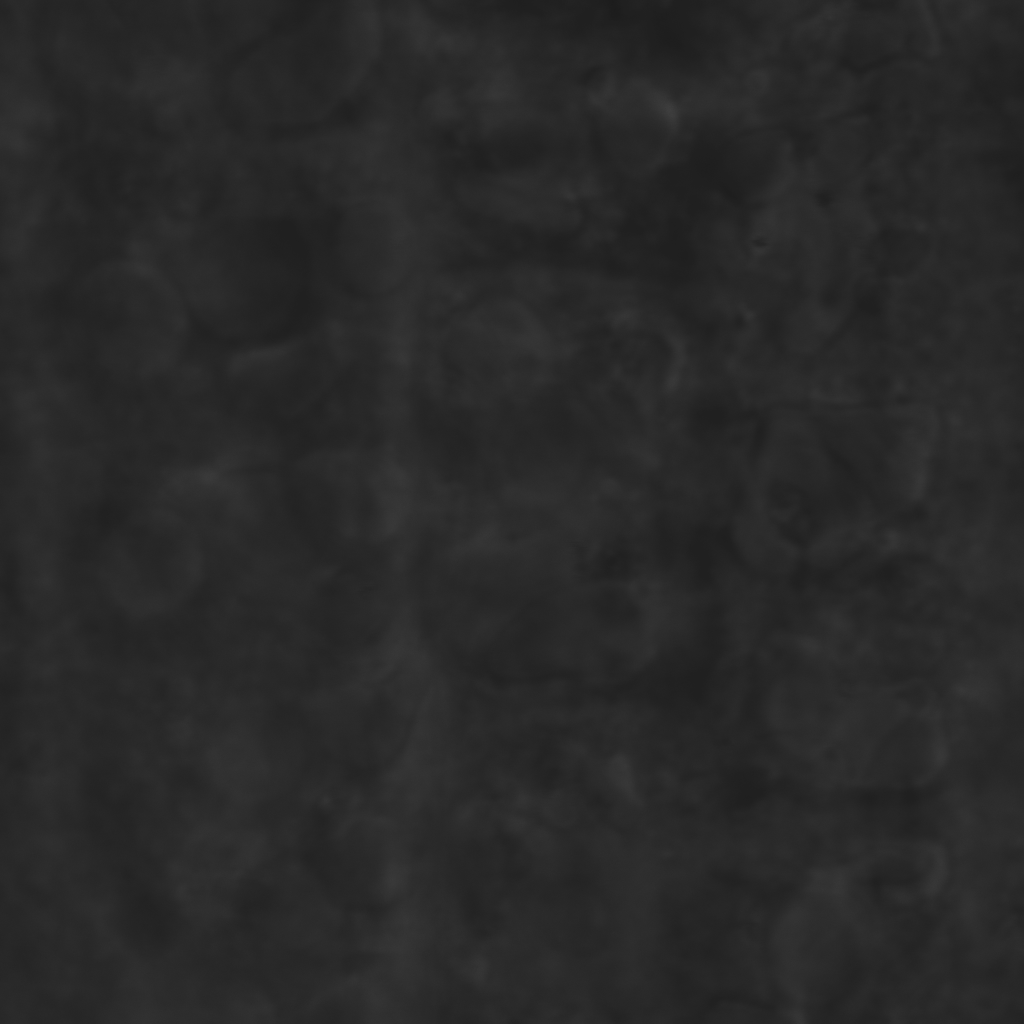

Supplement: Supplementary file 16 — Appendix Figure S6 Source Data [file 44318_2025_614_MOESM16_ESM.zip › Appendix Fig S6/fer-4 FER-GFP RALF1/Image012_ch02.tif]

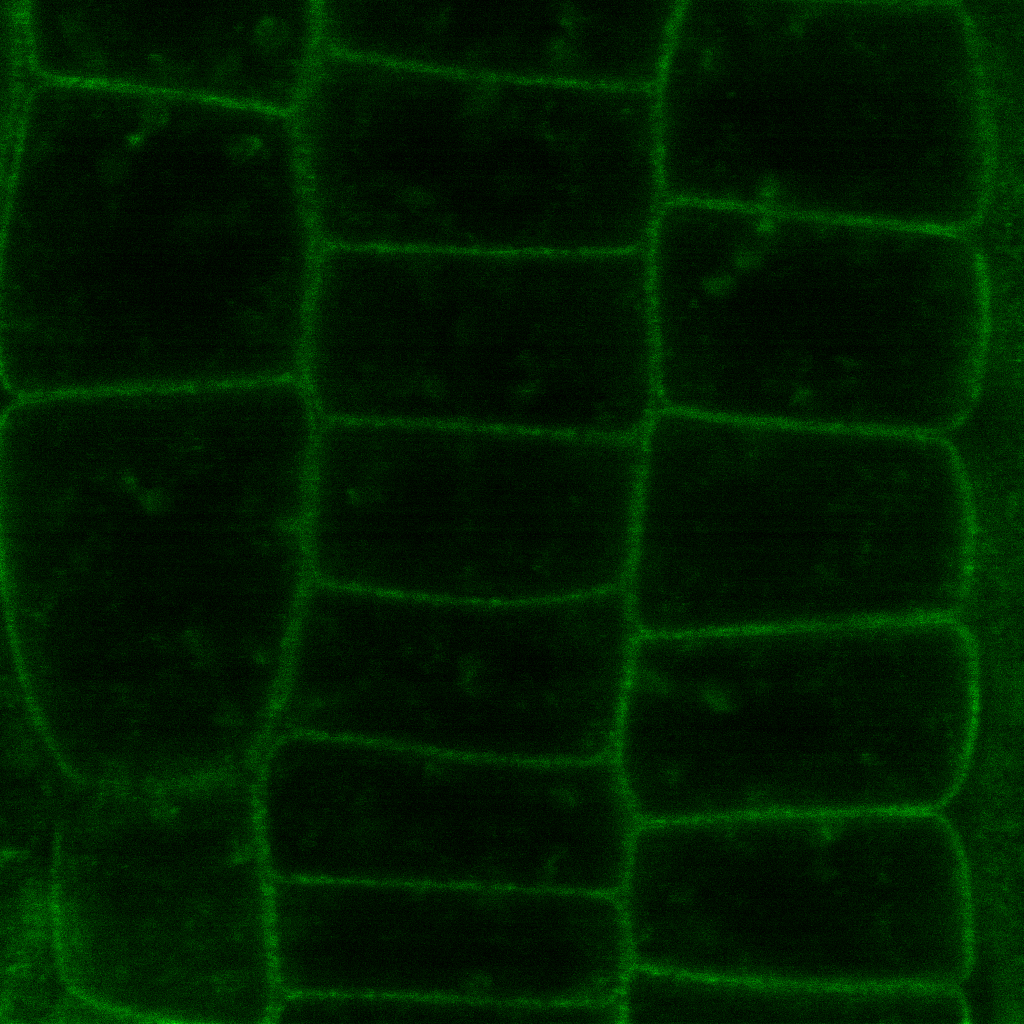

Supplement: Supplementary file 16 — Appendix Figure S6 Source Data [file 44318_2025_614_MOESM16_ESM.zip › Appendix Fig S6/fer-4 pp2ch triple FER-GFP mock/Image012_ch00.tif]

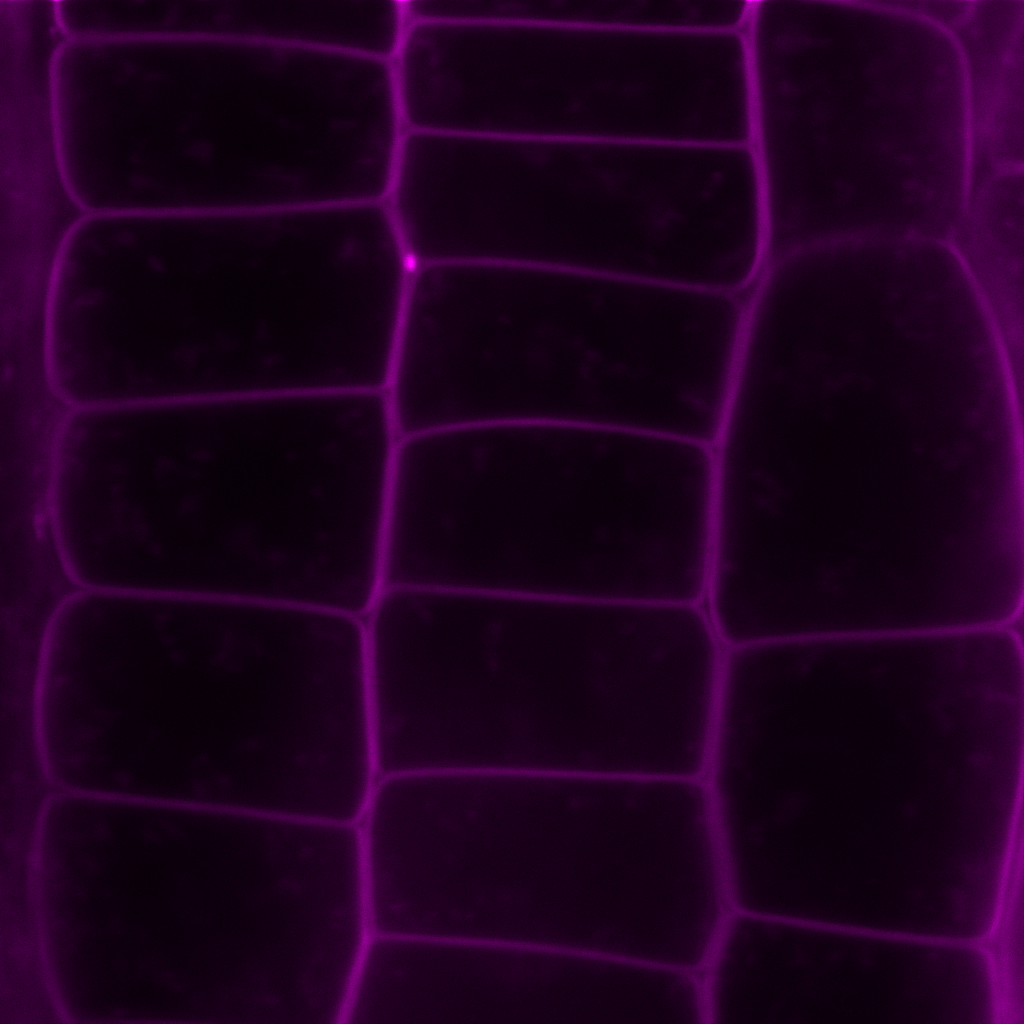

Supplement: Supplementary file 16 — Appendix Figure S6 Source Data [file 44318_2025_614_MOESM16_ESM.zip › Appendix Fig S6/fer-4 pp2ch triple FER-GFP mock/Image012_ch01.tif]

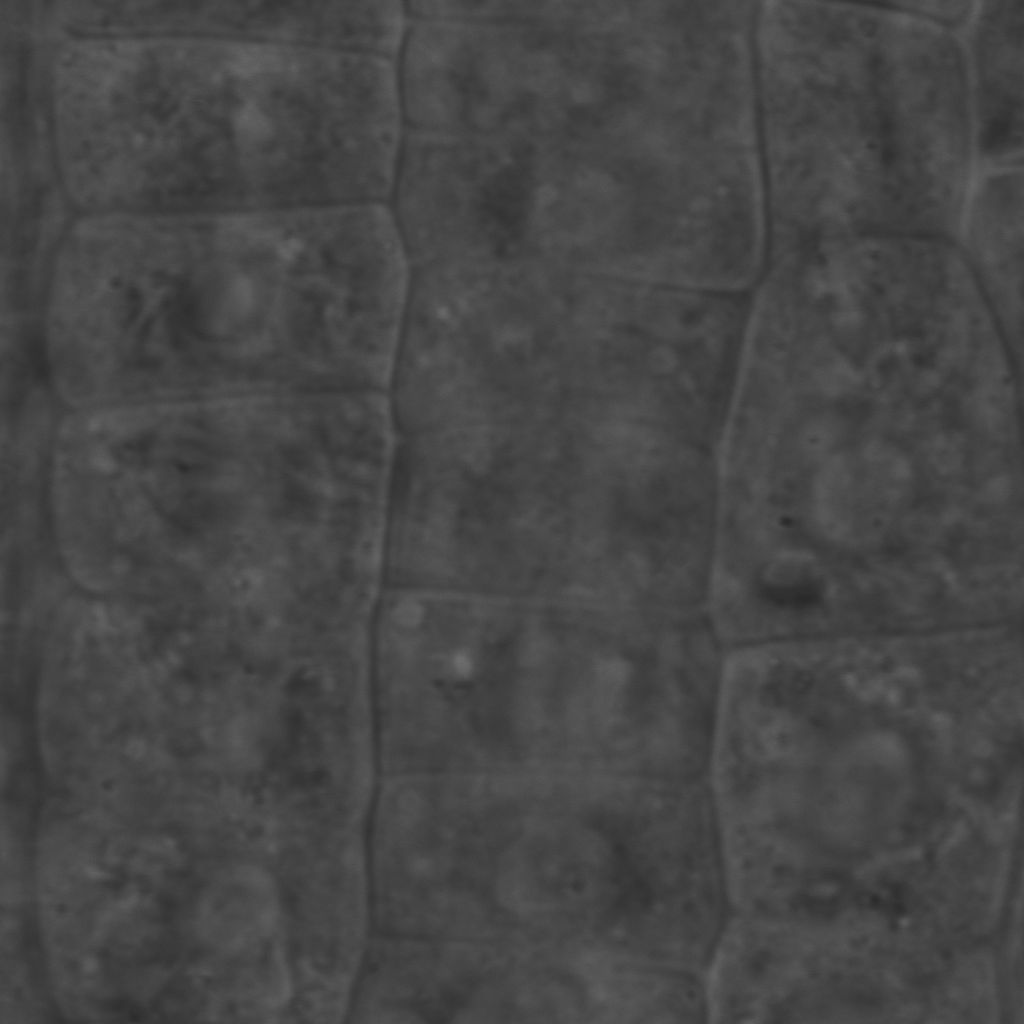

Supplement: Supplementary file 16 — Appendix Figure S6 Source Data [file 44318_2025_614_MOESM16_ESM.zip › Appendix Fig S6/fer-4 pp2ch triple FER-GFP mock/Image012_ch02.tif]

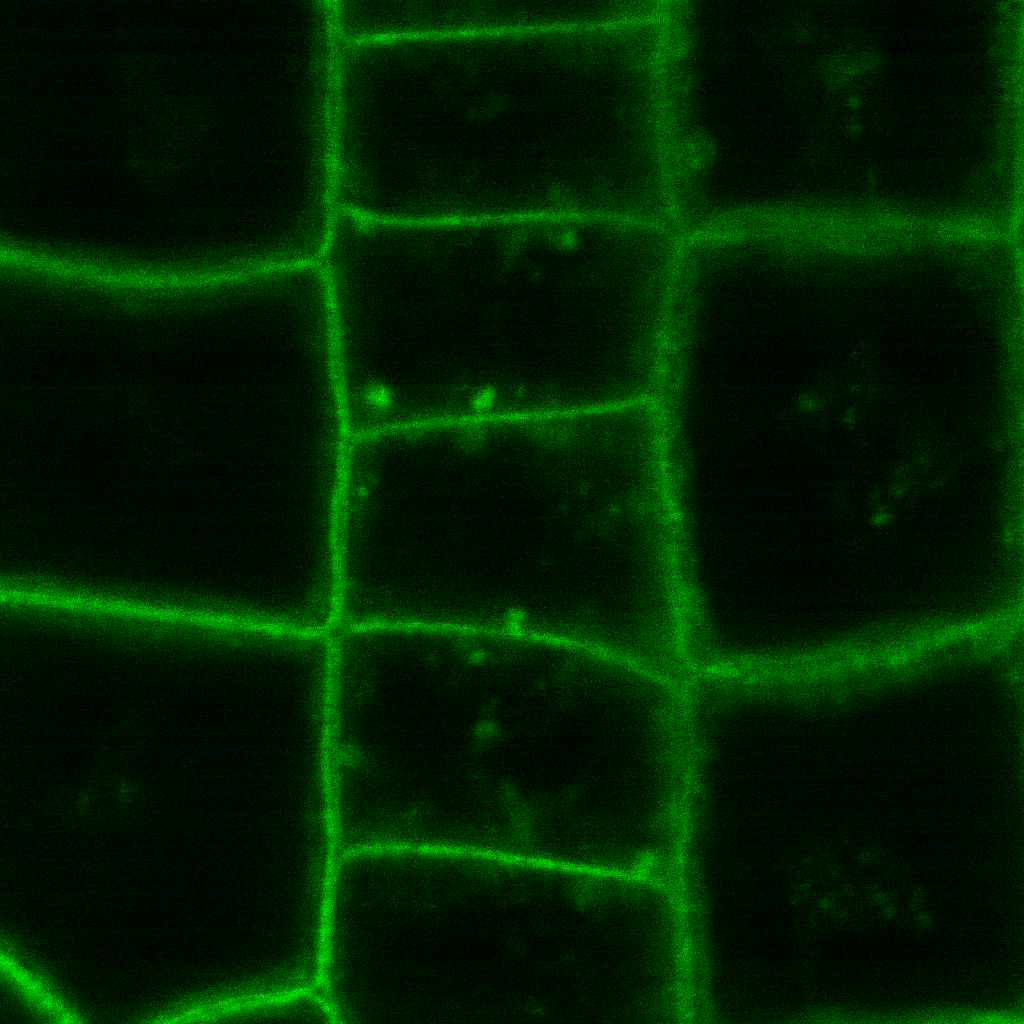

Supplement: Supplementary file 16 — Appendix Figure S6 Source Data [file 44318_2025_614_MOESM16_ESM.zip › Appendix Fig S6/fer-4 pp2ch triple FER-GFP RALF1/Image008_ch00.tif]

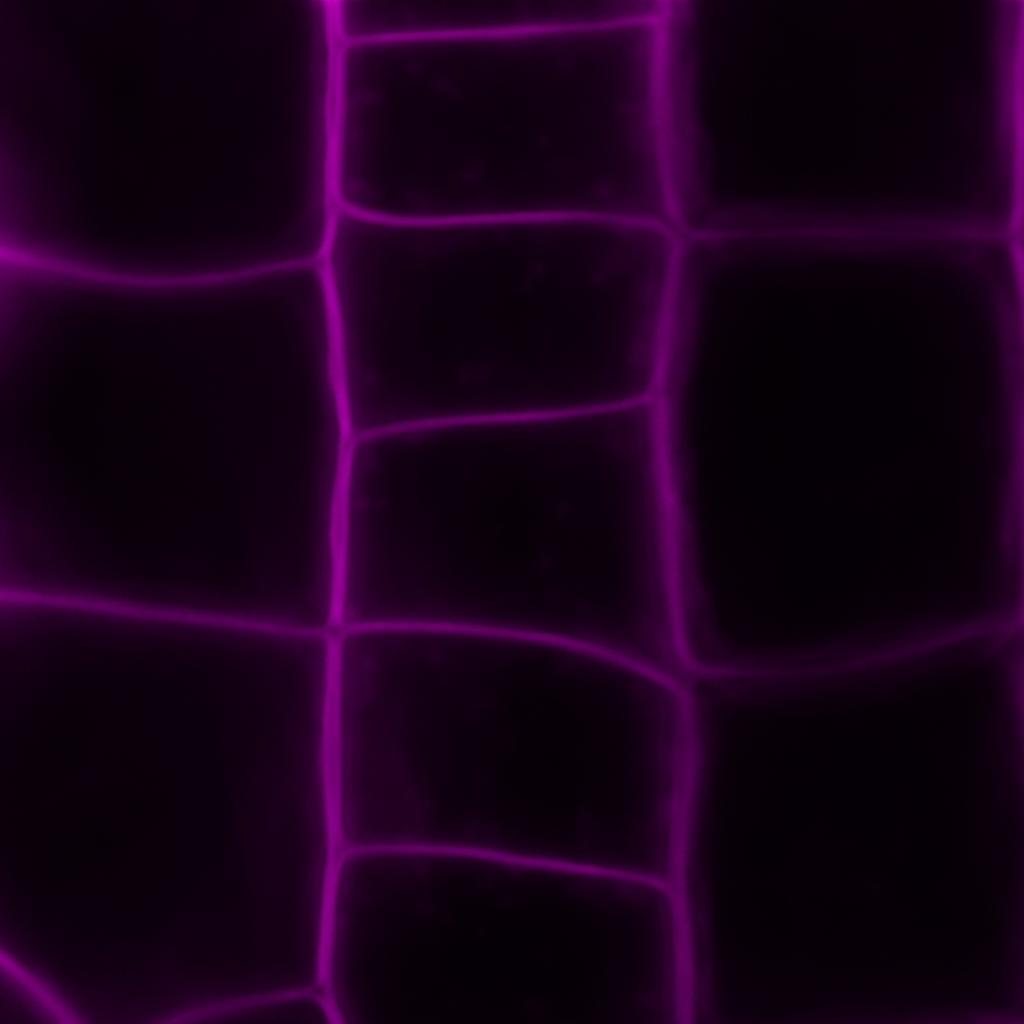

Supplement: Supplementary file 16 — Appendix Figure S6 Source Data [file 44318_2025_614_MOESM16_ESM.zip › Appendix Fig S6/fer-4 pp2ch triple FER-GFP RALF1/Image008_ch01.tif]

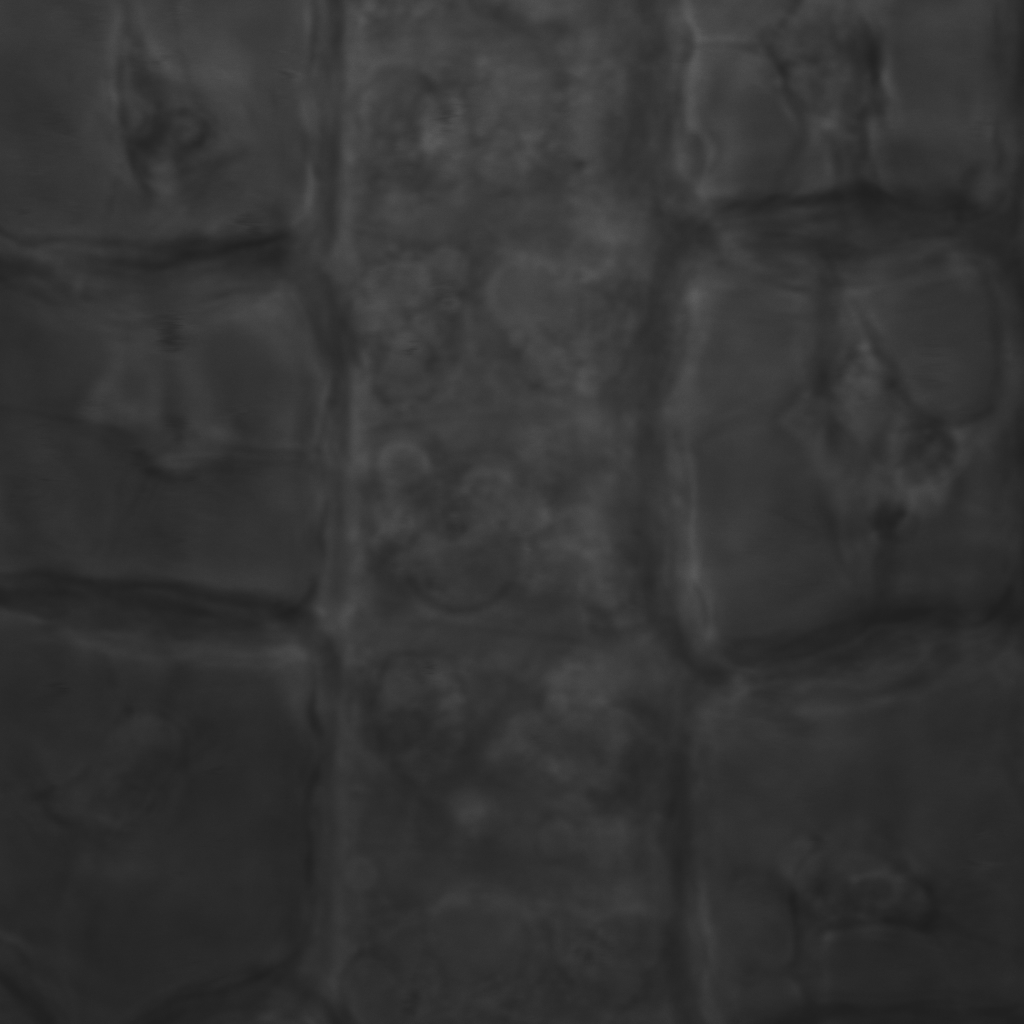

Supplement: Supplementary file 16 — Appendix Figure S6 Source Data [file 44318_2025_614_MOESM16_ESM.zip › Appendix Fig S6/fer-4 pp2ch triple FER-GFP RALF1/Image008_ch02.tif]
