# Supplementary material for: The Arabidopsis phosphatase PP2C12 negatively regulates LRX-RALF-FER-mediated cell wall integrity sensing
Source: EMBO J. 2025 Nov 17;45(1):243–60. doi: 10.1038/s44318-025-00614-x (PMC12759080; doi:10.1038/s44318-025-00614-x)
Supplement: Supplementary file 10 — Source data Fig. 9 [file 44318_2025_614_MOESM10_ESM.zip › Fig 9/Fig. 9B/README Figure 9.docx]

README Figure 9

The raw data used to produce the panel B is based on the seedlings shown in panel A.
