# Supplementary material for: The Arabidopsis phosphatase PP2C12 negatively regulates LRX-RALF-FER-mediated cell wall integrity sensing
Source: EMBO J. 2025 Nov 17;45(1):243–60. doi: 10.1038/s44318-025-00614-x (PMC12759080; doi:10.1038/s44318-025-00614-x)
Supplement: Supplementary file 11 — Appendix Figure S1 Source Data [file 44318_2025_614_MOESM11_ESM.zip › Appendix Fig S1/Fig S1F/readme.docx]

Fig. S1F

The originals of the bright field images of NES and nes were not found. They were done the same way and the same magnification as the one of PP2C12-GFP that is shown.
