# Supplementary material for: The Arabidopsis phosphatase PP2C12 negatively regulates LRX-RALF-FER-mediated cell wall integrity sensing
Source: EMBO J. 2025 Nov 17;45(1):243–60. doi: 10.1038/s44318-025-00614-x (PMC12759080; doi:10.1038/s44318-025-00614-x)
Supplement: Supplementary file 15 — Appendix Figure S5 Source Data [file 44318_2025_614_MOESM15_ESM.zip › Appendix Fig S5/README Appendix Fig S5.docx]

# README Appendix Fig S5

There is no «original» version of these graphs. They are based on the proteomic data shown in Appendix Table S1.
