# Supplementary material for: The Arabidopsis phosphatase PP2C12 negatively regulates LRX-RALF-FER-mediated cell wall integrity sensing
Source: EMBO J. 2025 Nov 17;45(1):243–60. doi: 10.1038/s44318-025-00614-x (PMC12759080; doi:10.1038/s44318-025-00614-x)
Supplement: Supplementary file 17 — Appendix Figure S7 Source Data [file 44318_2025_614_MOESM17_ESM.zip › Appendix Fig S7/README Suppl Fig S7.docx]

README Suppl Fig S7

The raw data are the pictures (scan of plates with seedlings) shown in the figure
